# The contrasting reactivity of *trans*- *vs. cis*-azobenzenes (ArN=NAr) with benzynes†

**DOI:** 10.1039/d3sc02253f

**Published:** 2023-06-06

**Authors:** Dorian S. Sneddon, Thomas R. Hoye

**Affiliations:** a Department of Chemistry, University of Minnesota 207 Pleasant St. SE Minneapolis MN 55455 USA hoye@umn.edu

## Abstract

We report here a study that has revealed two distinct modes of reactivity of azobenzene derivatives (ArN=NAr) with benzynes, depending on whether the aryne reacts with a *trans*- or a *cis*-azobenzene geometric isomer. Under thermal conditions, *trans*-azobenzenes engage benzyne *via* an initial [2 + 2] trapping event, a process analogous to known reactions of benzynes with diarylimines (ArC=NAr). This is followed by an electrocyclic ring opening/closing sequence to furnish dihydrophenazine derivatives, subjects of contemporary interest in other fields (*e.g.*, electronic and photonic materials). In contrast, when the benzyne is attacked by a *cis*-azobenzene, formation of aminocarbazole derivatives occurs *via* an alternative, net (3 + 2) pathway. We have explored these complementary orthogonal processes both experimentally and computationally.

## Abstract 2

*E*- *vs. Z*-azobenzenes engage benzynes in complementary fashion, producing either dihydrophenazine or *N*-aminocarbazole skeletons, respectively. DFT and experimental mechanistic studies shed light on why.

## Graphical abstract

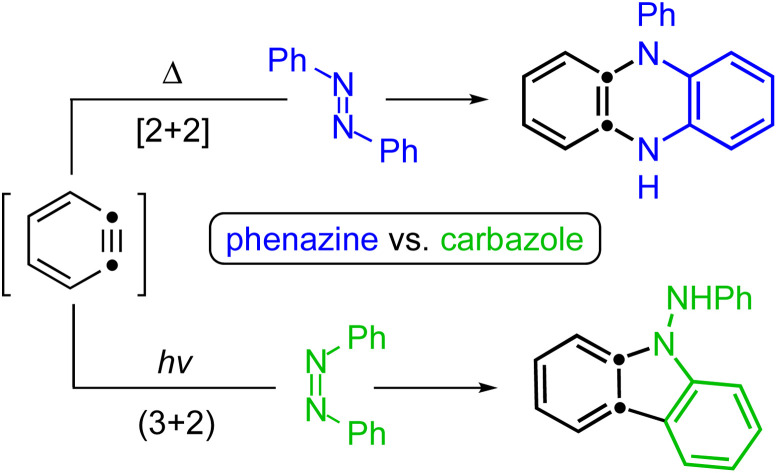

## 1 Introduction

Benzynes (and arynes more generally) are remarkably versatile reactive intermediates, especially with respect to the manifold classes of trapping agents with which they will engage.^1^ Azoarenes (ArN=NAr) comprise a class of compounds of long-standing interest.^2^ We can locate only a single report of reactions of azo compounds with benzynes.^3^ In that work products containing an *N*-aminocarbazole skeleton were produced. We describe here a detailed investigation into the complementary modes of reactivity observed between benzynes and *trans*- *vs. cis*-azobenzenes.

**Fig. 1:**
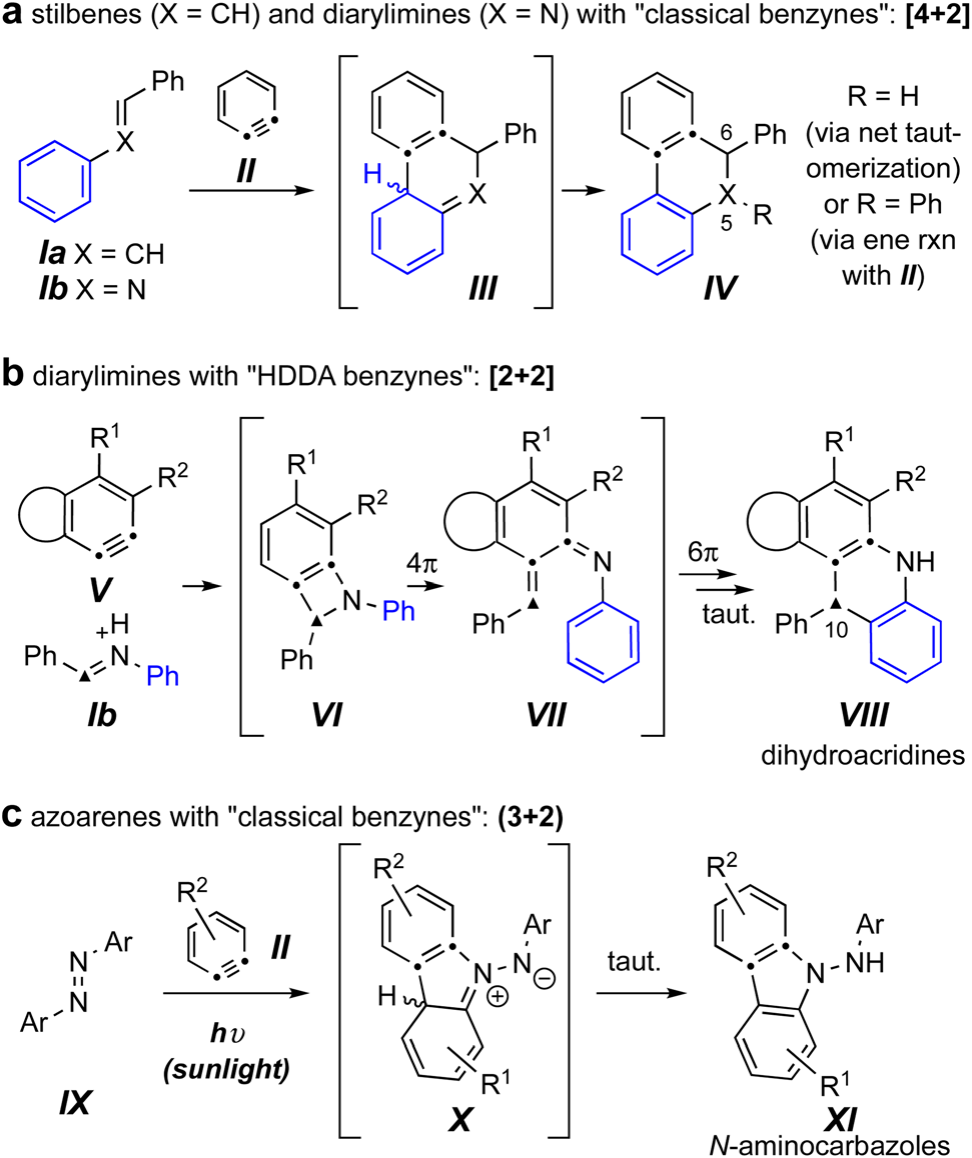
(a) Known reactions of classical benzynes with stilbenes and imines. (b) Known reactions of imines with HDDA-benzynes.^8^ (c) Known photo-stimulated reactions of azoarenes with benzynes.^3^

Reactions of benzynes with the related trapping agents stilbene^4,5^ (Ia) and its mono aza-analog *N*-benzylideneaniline^6,7^ (Ib) are known (Fig. 1a). With classically generated benzynes [*e.g.*, *o*-benzyne (II)] these proceed by either exclusive or predominant formation of initial [4 + 2] cycloadducts (*cf.*III), which then tautomerize to products IV. In contrast, imines such as Ib engage the more complex and, necessarily, sterically hindered hexadehydro-Diels–Alder (HDDA) benzynes V, to give dihydroacridines VIII arising from an initial [2 + 2] event (*cf.*VI) and ensuing electrocyclic ring-opening (*cf*. VII) and -closing processes (Fig. 1b).^8^

The sole example we can locate of diaza-analogs of stilbenes – *i.e.*, azoarenes (IX) – engaging *o*-benzynes was recently described by Wang, Li, and coworkers and proceeded *via* a net (3 + 2) cycloaddition under sunlight (Fig. 1c).^3^ This produced the *N*-aminocarbazole derivatives XI, a process suggested to take place *via* zwitterionic intermediates X. The authors noted that in the absence of sunlight the same “reaction did not proceed.”^3^ This led us to question which of the three distinct modes of reaction shown in Fig. 1 would ensue when an azobenzene engaged a (more hindered) “HDDA benzyne.” Would an overall [4 + 2] pathway lead to a dihydrocinnoline skeleton (a 5,6-diaza-analog of IV); would an initial [2 + 2] pathway produce a dihydrophenazine (a 10-aza analog of VIII); or would the reaction mirror the case of the photochemically driven process to give *N*-aminocarbazoles analogous to XI as observed by Li *et al.*?^3^

## 2 Results and discussion

**Fig. 2:**
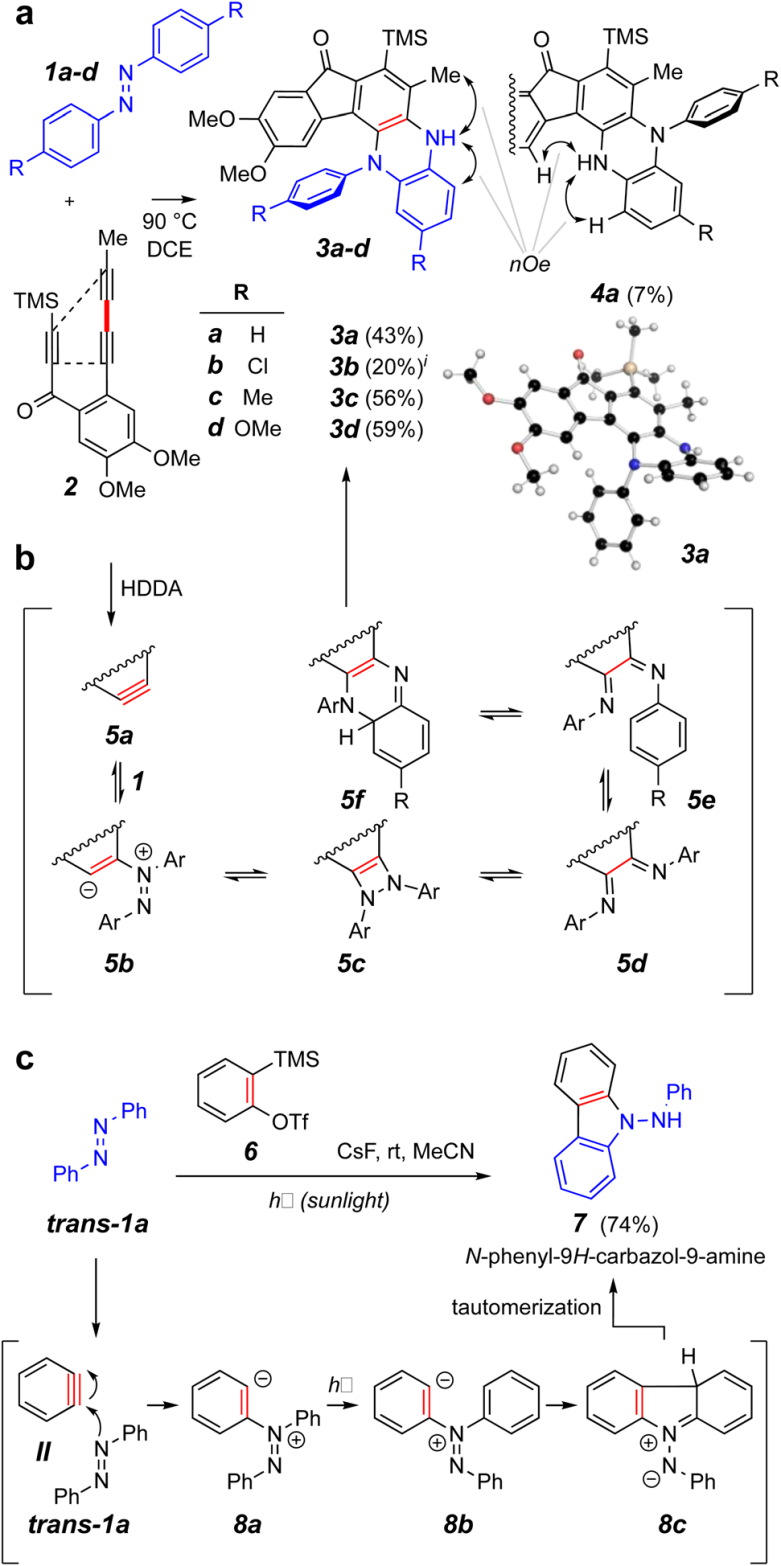
(a) Reaction of *trans*-azobenzene derivatives 1a–d with the benzyne produced by heating triyne 2 to give dihydrophenazines 3 and 4. (b) Possible intermediates on the reaction coordinate leading to the dihydrophenazines. (c) Contrasting mode of reactivity when *o*-benzyne (II) engages azobenzene (*trans*-1a) during exposure to sunlight.^3^^*i*^A small amount of the analog of isomer 4a was observed in the chromatographed sample of this product (see ESI†).

We elected to start our investigations by using a prototypical thermal HDDA substrate. In one of our earliest experiments (Fig. 2a), *trans*-azobenzene (*trans*-1a = IX, 10 equiv.) and the triyne 2 were heated together in a solution of 1,2-dichloroethane (DCE) at 90 °C for 15 h. This is a temperature at which 2 will undergo a HDDA cycloisomerization^9^ to give the corresponding benzyne (*cf.*5a, Fig. 2b). This experiment produced the monoarylated dihydrophenazine 3a along with a small amount of its regioisomeric product 4a in modest yields. The structures of these isomers were definitively distinguished by the complementary, indicated nOe interactions involving the NH proton in each. This structure assignment was further substantiated by an X-ray diffraction analysis of the major isomer 3a. Notably, the pathway leading to the phenazine skeleton in 3a is new and distinct from that observed in the reported photo-induced reactions leading to aminocarbazole derivatives (Fig. 1c). Likewise, the additional azobenzene derivatives 1b–d gave the dihydrophenazines 3b–d as the major if not solely observed products.

The formation of products having the phenazine skeleton from the reaction of an aryne and an azobenzene is novel. Phenazines and dihydrophenazines are of considerable current interest.^10^ For example, they are important in the arenas of optical sensing, electrochemistry, and organic electronics and photonics.^11^ In the optical sensing realm, these can show vibration-induced emission (VIE), allowing them to exhibit multicolor emission. Additionally, certain phenazines exhibit thermally activated delayed fluorescence (TADF), making them attractive organic light-emitting diode (OLED) candidates. Phenazine derivatives may additionally act as ion battery cathode materials. They can also be incorporated into various organic frameworks for use as both electronic and photonic materials. Also, phenazines and dihydrophenazines are commonly found as secondary metabolites, some of which also have interesting biological properties.^12,13^

**Fig. 3:**
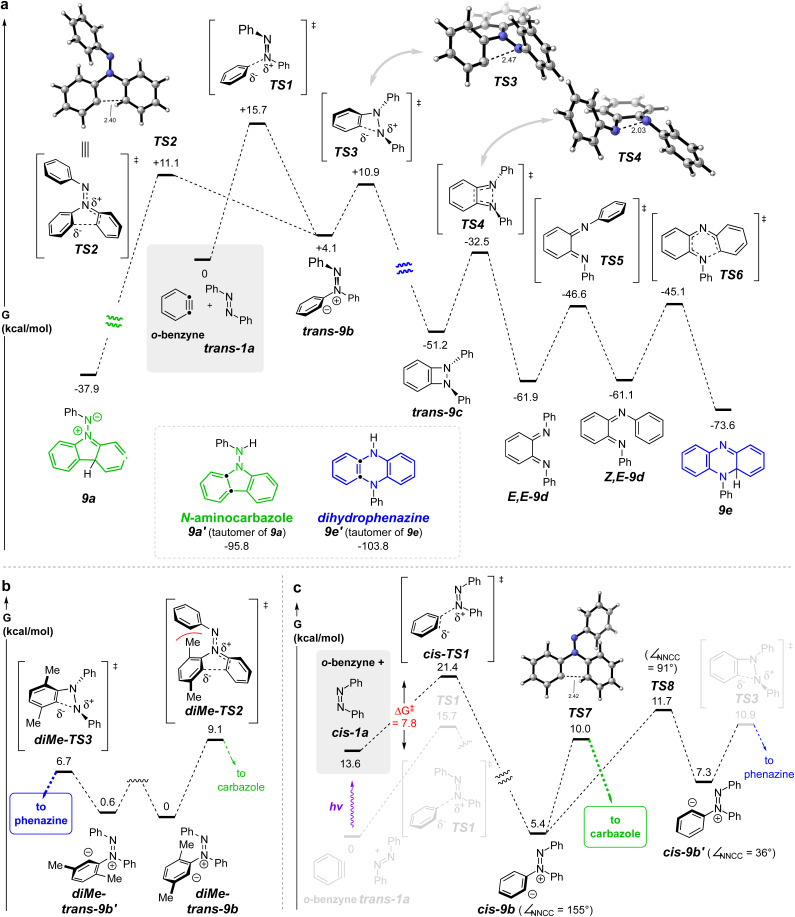
(a) DFT^*i*^ potential energy surface for the reaction of *o*-benzyne with *trans*-azobenzene (*trans*-1a) (gray box) leads to both phenazine and carbazole skeletons with essentially the same activation barrier (*cf*. TS2*vs.*TS3). (b) The more hindered 3,6-dimethylbenzyne, a better mimic of an HDDA-benzyne, shows a significant preference for the phenazine-forming pathway (*cf*. diMe-TS3*vs.*diMe-TS2). (c) *cis*-Azobenzene (*cis*-1a) gives *cis*-9b, the precursor to the carbazole product, *via* a lower activation barrier (7.8 kcal mol^−1^) than that of *trans*-1a to TS1 (15.7 kcal mol^−1^), the forerunner to the phenazine product; *cis*-9b proceeds to the aminocarbazole product *via*TS7 faster than isomerizing to *cis*-9b′, which could have led to the dihydrophenazine product *via*TS3. ^*i*^SMD(benzene)/MN15/6-311++G(d,p).

A mechanistic possibility for the outcome of the thermal reactions with *trans*-azobenzenes leading to the phenazine skeleton is suggested by structures 5a–f (Fig. 2b). This is discussed in greater detail in conjunction with the DFT studies shown in Fig. 3. A contrasting mechanistic rationale for the aminocarbazole formation seen in the work of Wang *et al.* is shown in Fig. 2c. They proposed that the generation of *o*-benzyne (II) by the Kobayashi method in the presence of *trans*-azobenzene (*trans*-1a) gave rise by nucleophilic attack to the zwitterion 8a and that this species absorbed a photon that resulted in isomerization to its geometric isomer 8b. This then cyclized to form the new five-membered ring present in the species 8c (aza-Nazarov-like), a tautomer of the more stable, aromatized aminocarbazole 7.

We turned to DFT studies to parse out some of the energetic details associated with these contrasting modes of reaction. A full potential energy surface (PES) for the pathway leading to the dihydrophenazine is shown in Fig. 3a. *trans*-Azobenzene (*trans*-1a) engages *o*-benzyne *via*TS1 to form zwitterion *trans*-9b (=8a, Fig. 2c). We computed this zwitterion to undergo transformation to either the benzodiazetidine *trans*-9c*via*TS3 or to the zwitterion 9a (=8c, Fig. 2c) *via*TS2. The very similar energies of activation for these competing processes is inconsistent with the fact that we did not observe any products with the aminocarbazole skeleton in the HDDA reactions described in Fig. 2a, an important point to which we return in the following paragraph. The benzodiazetidine *trans*-9c is a rare type of intermediate. The only compound we can find containing a benzodiazetidine (7,8-diazabicyclo[4.2.0]octa-1(6),2,4-triene) substructure is that of the parent, fully unsubstituted molecule, formed in an argon matrix and characterized by its infrared spectroscopic features.^14,15^ This transient species rapidly ring-opened to the bis-imino-*o*-benzoquinone. Likewise, *trans*-9c was computed to isomerize to the bis-imine *E*,*E*-9d through a conrotatory electrocyclic ring opening *via*TS4. *E*/*Z*-Isomerization *via*TS5 (N-inversion) give the diiminoquinone *Z*,*E*-9d, which is poised to undergo 6π-electrocyclization *via*TS6 to furnish the penultimate intermediate 9e. Notably, all of the activation barriers (*via*TS1–TS6) on this computed potential energy surface are <20 kcal mol^−1^.

Recall that the barrier for the cyclization of *trans*-9b to a five-membered ring (aza-Nazarov) enroute to 9a was essentially identical with that leading to the diazetidine *trans*-9c. Why, therefore, did we not see aminocarbazole formation in the reactions of the HDDA-benzyne with *trans*-azobenzenes 1a–d? The HDDA benzyne bears substituents on carbons 3 and 6 flanking the sp-hybridized benzyne carbons. To assess the impact of this type of substitution, we computed the analogous key intermediates for reaction between *trans*-1a and, now, 3,6-dimethyl-*o*-benzyne (Fig. 3b). A striking preference (ΔΔ*G*^‡^ = 2.4 kcal mol^−1^) for cyclization to the benzodiazetidine was seen. This difference is attributable to the unfavorable steric interaction portrayed in diMe-TS2 when compared to that in diMe-TS2. The explicit precursors to these two transition structures are the conformers diMe-*trans*-9b and diMe-*trans*-9b′, respectively; related conformers will play a role in later discussion of formation of aminocarbazole products.

The formation of dihydrophenazine derivatives by this unprecedented process is appealing, and we were encouraged to explore potential improvements in the efficiency of the transformation. In turn, this led us to consider modifications that might improve this process. The barrier of 15.7 kcal mol^−1^ for *trans*-1a to traverse TS1 is higher than a number of other additions to arynes by species containing nucleophilic nitrogen atoms.^16–18^ We speculated that *cis*-azobenzene (*cis*-1a), having easier steric access to each azo nitrogen atom as well as greater negative charge character at each of its nitrogens [*e.g.*, atomic polar tensor = −0.167 *vs.* +0.008 for *cis*- *vs. trans*-azobenzene, respectively (APT in Gaussian)] could serve as a more competent nucleophile in this process. *cis*-Azoarenes have been reported to engage in net-cycloaddition reactions faster than their *trans*-counterparts.^19–23^

The computed PES for the reaction between *cis*-1a and *o*-benzyne is shown in Fig. 3c. Significantly, the activation barrier passing through *cis*-TS1 enroute (to *cis*-9b) is computed to be nearly 8 kcal mol^−1^ lower than that for *trans*-1a to proceed through TS1 (to *trans*-9b): *i.e.*, 7.8 *vs.* 15.7 kcal mol^−1^. This led us to first investigate whether we could use photons to produce a sufficiently high proportion of *cis*-1a to allow the overall energetically favored process to be the capture of the benzyne by the *cis*-azobenzene. (Later we return to discuss the remainder of the computed energetics shown in Fig. 3c proceeding onward from *cis*-9b).

**Fig. 4:**
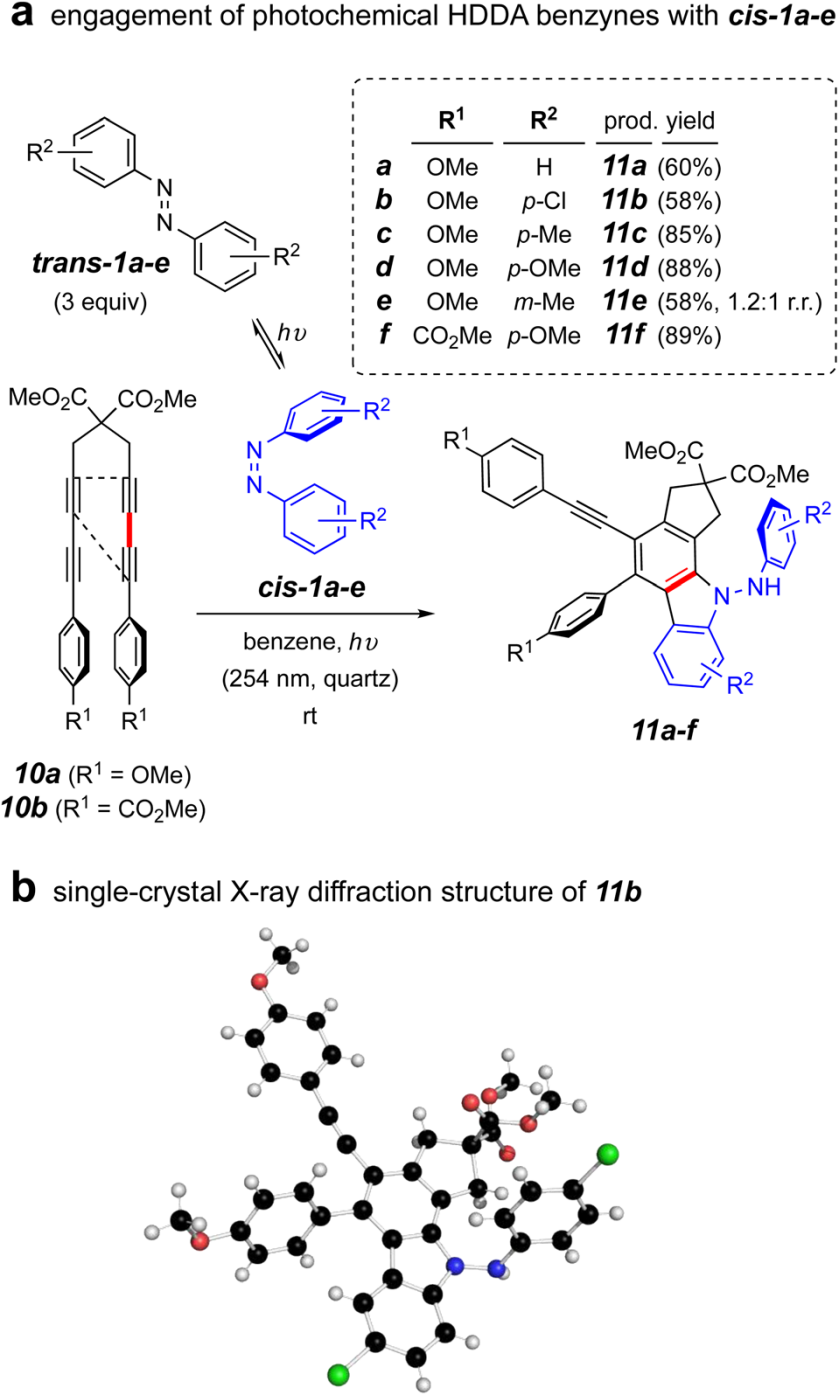
(a) Reaction of photochemical HDDA precursors 10a–b with a photo-generated mixture of *trans*- and *cis*-1a–f under continuous irradiation; (b) single-crystal X-ray diffraction structure of 11b.

To address this question, we carried out the first experiment shown in Fig. 4a. This involved use of the tetrayne 10a, an HDDA substrate known to efficiently undergo photo-induced cycloisomerization to its corresponding HDDA-benzyne.^24^ This allowed both of the benzyne and *cis*-azobenzene reactants to be formed at ambient temperature when irradiated at 254 nm. We pre-irradiated *trans*-1a in benzene in a quartz vessel in a Rayonet reactor for 1 h. Tetrayne 10a was introduced and irradiation continued overnight. This resulted in a single isolable product that, to our surprise proved not to be the expected dihydrophenazine analog of 3a. Instead, this was deduced to be the aminocarbazole compound 11a. This process was shown to be general, proceeding also starting with the azobenzenes *trans*-1b–e. Those with more electron rich aryl substituents resulted in higher product yields. The ester-substituted tetrayne 10b also showed very efficient conversion to product 11f. The structure assignments of this series of products was reflected in their parallel NMR spectral properties, as seen from extensive interpretation of both 1D as well as 2D ^1^H and ^13^C spectra. In addition, an X-ray diffraction structure of the analog 11b confirmed the assignments of these aminocarbazole-containing compounds (Fig. 4b).

**Fig. 5:**
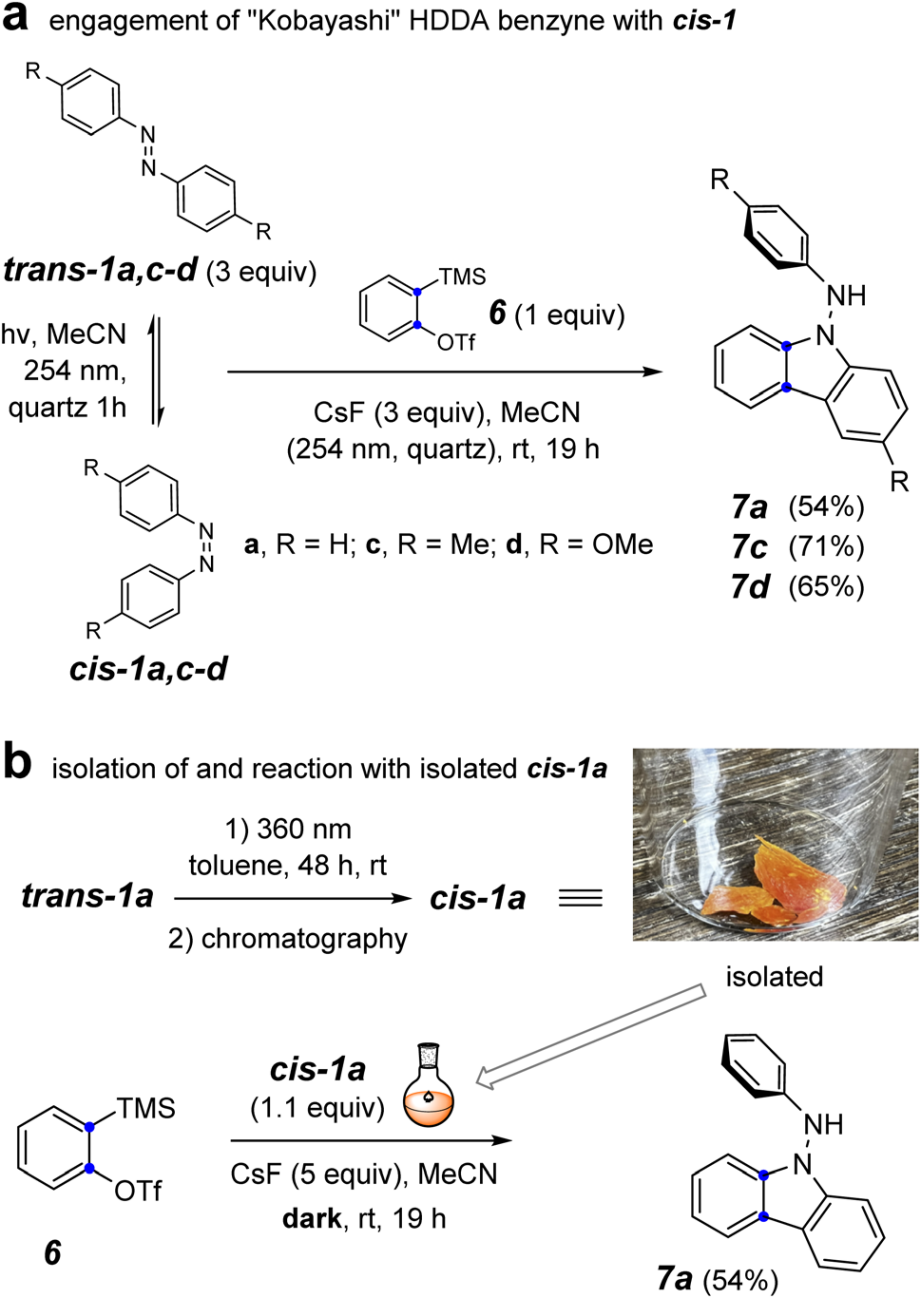
Reaction of *o*-benzyne with (a) a photo-generated mixture of trans- and *cis*-1a,c–d under continuous irradiation and (b) isolated *cis*-1a in the dark.

To probe the involvement of the *cis*-azobenzene in benzyne trapping more explicitly, we explored the trapping of a “Kobayashi-generated benzyne”^25,26^ as presented in Fig. 5. When *o*-benzyne was generated from 2-trimethylsilylphenyl triflate (6) starting with 3 equivalents of a preirradiated solution of a *trans*-azobenzene derivative and under further continuous irradiation, the photochemically established amount of the *cis*-azobenzene *cis*-1a, *cis*-1c, or *cis*-1d proceeded to give the respective aminocarbazole derivative 7a, 7c, or 7d in good yield (Fig. 5a). As further evidence that the *cis*-azobenzene geometric isomer is the key reactive intermediate effecting each of these overall transformations, *cis*-1a was purified by flash chromatography and used soon thereafter as a single isomer to trap *o*-benzyne (Fig. 5b). In an experiment employing only 1.1 equivalent of pure *cis*-1a relative to the Kobayashi precursor 6, the aminocarbazole 7a was obtained in similar yield to that arising from the photostationary mixture of the azobenzenes. Finally, generating *o*-benzyne in the presence of pure *trans*-1a (1.1 equivalent) but in the absence of irradiation gave no discernible amount of the aminocarbazole derivative 7a. The yield of this reaction was not as high as for the more hindered HDDA benzynes, perhaps because of some competing secondary reaction between the NH_2_ group in 7a and additional *o*-benzyne. Collectively, the computational and experimental results presented in Fig. 3–5 indicate that (i) the *cis*-azobenzene geometric isomer is a more reactive trap for a benzyne, (ii) *cis*-azobenzene is the key intermediate responsible for launching the transformation leading to aminocarbazole derivatives, and (iii) the *trans*-azobenzene geometric isomer is uniquely responsible for furnishing dihydrophenazines in the case of HDDA benzynes. In view of these results, we suggest that the process leading to aminocarbazole formation is better viewed as arising from the innate reactivity of *cis*-azobenzene itself (*cf*. Fig. 3c) rather than one in which intermediate *trans*-9b is photoisomerized to *cis*-9b (*cf.*8a to 8b, Fig. 2c).^3^

Having clearly demonstrated experimentally that the *cis*-azobenzene isomer is responsible for redirecting the mode of reaction with benzynes to produce aminocarbazoles rather than dihydrophenazines, we further explored the behavior of the zwitterion *cis*-9b by DFT (Fig. 3c). This species can either undergo aza-Nazarov cyclization directly to the carbazole skeleton *via*TS7 or undergo an aryl rocking motion (TS8) to access an alternative rotamer of the zwitterion, namely *cis*-9b′. While *cis*-9b′ could geometrically access TS3, thereby intersecting with the phenazine pathway, we found, perhaps somewhat surprisingly, that the rotational barrier to produce *cis*-9b′*via*TS8 was higher than proceeding to the aminocarbazole *via*TS7 by 1.7 kcal mol^−1^. We attribute the relatively high barrier of aryl rotation to the penalty of breaking conjugation of the 1,2-diaza-1,3-butadiene system in the nearly orthogonal transition state geometry (*cf.* dihedral angle values in *cis*-9b and *cis*-9b′*vs.*TS8). Taken at face value, these energetics suggest that *ca.* 95% of *cis*-9b should undergo aza-Nazarov cyclization to the carbazole rather than isomerizing to *cis*-9b′ and furnishing phenazine products.

Likewise, we explored by DFT (see Fig. S7 in the ESI† for details) whether *cis*-9b could isomerize directly to *trans*-9b (and then on to the phenazine) by the net isomerization of the N–N double bond geometry. This could occur either (i) by linearization of the N=NPh geometry (*i.e.*, inversion *via* an sp-hybridized nitrogen, *cf.*TS5, Fig. 3a) or (ii) by out-of-plane rotation about the N=N bond. A TS for the former process was located and it showed a free energy >20 kcal mol^−1^ higher than that of TS7. Attempts to find a TS for the latter geometric change instead optimized directly to TS3. These results suggest that it is unlikely for *cis*-9b to give rise to *trans*-9b, thereby precluding a second pathway by which the zwitterion *cis*-9b could give rise to a product having a phenazine skeleton.

To gain evidence addressing the later stages of the thermal mechanism involving the interesting electrocyclic processes emanating from the benzodiazetidine *trans*-9c (identified by the computations summarized in Fig. 3a), we synthesized the phenanthrene–diiminoquinone 12. In particular, we thought that the thermal behavior of this compound, an analog of species *E*,*E*-9d in the computational study, would shed light on the remaining steps in the mechanism (*i.e.*, isomerization to *Z*,*E*-9d and its electrocyclic closure to the 4*a*,5-dihydrophenazine 9e). The first hint of some unusual behavior of species 12 arose while attempting its preparation following a literature protocol for TiCl_4_-promoted condensation of 9,10-phenanthrenequinone with anilines.^27,28^ In our hands this repeatedly gave a complex array of products from which the major isolable product was, surprisingly, the diamine 13. Once in hand, this could be smoothly oxidized to 12 using MnO_2_, although its disproportionation lability upon further handling was soon recognized.

**Fig. 6:**
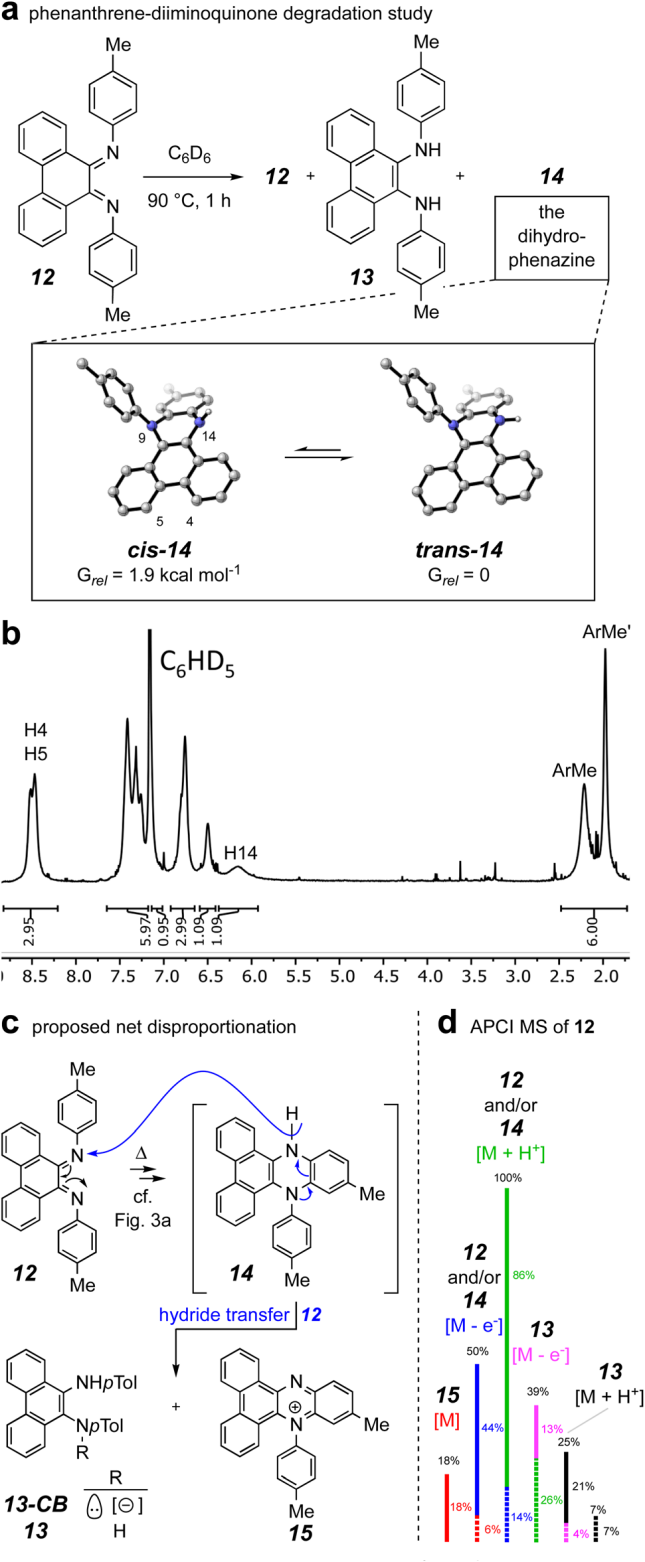
Disproportionation processes emanating from the diiminoquinone 12 to produce the diamine 13 and the phenazinium ion 15*via* the dihydrophenazine 14. (a) The reaction taken to partial conversion from which 14 was isolated along with a coeluting mixture of starting material (12) and 13. (b) ^1^H NMR spectrum of 14. (c) A mechanistic rationale for the overall redox-neutral transformation. (d) Illustration of the mixture of species/ions formed while recording an APCI mass spectrum of an isolated sample of the diiminoquinone 12.

When 12 was warmed in deuterobenzene (90 °C), its transformation could be monitored by ^1^H NMR spectroscopy. An experiment that was terminated after 1 h (*ca.* 67% conversion of the starting 12) resulted in generation of a mixture of the diamine 13 and dihydrophenazine 14 (Fig. 6a) in addition to the unreacted 12. Chromatographic purification provided a pure sample of the dihydrophenazine 14, which proved to be even more labile than its isomer 12. The ^1^H NMR spectrum of 14 (Fig. 6b) contained broad resonances; in retrospect these were also seen in the spectrum of the reaction mixture itself (see Fig. S4†). We identified by DFT two nearly equienergetic, diastereomeric, *cis*- and *trans*-conformers of 14 that differ only in the relative orientation of the hydrogen atom and lone pair of electrons on N14. The NMR spectrum of a *N*5,*N*10-diarylated analog having the same dibenzodihydrophenazine skeleton as 14 showed similar broadening.^29^ This spectroscopic phenomenon, along with the lability we observed for 14, could explain why past researchers have not recognized that *N*,*N*-diaryl-9,10-diiminophenanthrenequinones such as 12 undergo a facile 6π-electrocyclization.^27^

The presence of the diamine 13 arising from the condensation reaction meant to provide 12 (see ESI† for details) and the degradation behavior of 14 were initially puzzling; they implied that redox events were occurring. The only material present in the crude mixture that would serve as a reasonable reductant was the dihydrophenazine 14. Thus, we envisaged (Fig. 6c) an overall net disproportionation sequence in which formation of 14 (*cf.* mechanism delineated in Fig. 3a) sets up reduction of a second equivalent of 12*via* hydride transfer to give the phenazinium cation 15 and the conjugate base of 13 (13-CB) as its counter anion. A stylized APCI mass spectrum of a sample of purified diiminoquinone 12, ionized by thermal activation, is shown in Fig. 6d. Evidence for all of species 12–15 can be seen in the data. Taking into account the proportion of natural abundance ^13^C isotopologues for each member of this array of C_28_ species (dashed lines) allows deconvolution of the entire set of ion intensities as indicated by the color coding. Additionally, reverse-phase LCMS analysis of the crude product mixture also showed evidence for the presence of the phenazinium ion 15 (along with 12 and/or 14 and 13; see Fig. S5†). Taken together, these results support the mechanism in which a diiminoquinone intermediate (*cf.E*,*E*-9d and *Z*,*E*-9d) undergoes electrocyclization (and tautomerization) to furnish the dihydrophenazine core.

## 3 Conclusions

Herein, we have demonstrated the contrasting reactivity of benzynes with *trans*- *vs. cis*-azobenzene derivatives. Our observations indicate that when *trans*-azobenzene is employed as a trap for a benzyne, it reacts in a formal [2 + 2] cycloaddition, followed by electrocyclic ring opening to form a diiminoquinone intermediate. This can then undergo *E*/*Z* isomerization and electrocyclic ring closure to furnish dihydrophenazines after a final tautomerization (*cf.*Fig. 3a and b). We then demonstrated, both computationally (*cf.*Fig. 3c) and experimentally (*cf.*Fig. 4), that *cis*-azobenzene is a more competent trap for the benzyne, triggering an alternative reaction process – one that proceeds *via* a formal (3 + 2) cycloaddition event to furnish *N*-aminocarbazole derivatives. A key mechanistic experiment showed that the divergent reaction pathways emanate from the specific geometry of the initial azobenzene derivative. *cis*-Azobenzene was isolated chromatographically and reacted with *o*-benzyne in the dark to afford the aminocarbazole derivative as the only isolable product (*cf.*Fig. 5b).

Additional mechanistic experiments were carried out to rationalize the later stages of the [2 + 2] cascade leading to dihydrophenazines (*cf.*Fig. 3a). The phenanthrene diiminoquinone 12 was synthesized and used to probe the key electrocyclization event furnishing the dihydrophenazine skeleton. Upon heating, a novel net disproportionation process was discovered that led in part to the dihydrophenazine derivative 14, thereby lending support to our mechanistic rationale for the [2 + 2] reaction cascade between arynes and *trans*-azobenzene (*cf.*Fig. 6). Overall, our data demonstrate two non-convergent reaction pathways, each predetermined by the *trans*- *vs. cis*- geometry of the azobenzene used to engage the benzyne.

## 4 Experimental

### 4.1. General procedure for thermal synthesis of phenazine derivatives from HDDA-generated benzynes and azobenzene derivatives

The polyyne precursor (1 equiv.) and the azobenzene derivative (10 equiv.) were combined in a screw-capped culture tube. 1,2-Dichloroethane was added to bring the solution to an initial concentration of the polyyne of 0.01 M. The resulting solution was placed in an oil bath maintained at 90 °C for *ca.* 16 h. Subsequently, the reaction mixture was passed through a silica gel plug and eluted with EtOAc. The volatiles were removed under reduced pressure, and the crude residue was purified using MPLC with the elution solvent mixture indicated for each compound.

### 4.2. General procedure for photochemical synthesis of aminocarbazole derivatives from HDDA-generated benzynes and azobenzene derivatives

The azobenzene derivative (3 equiv.) was placed in a quartz glass tube and dissolved in *ca.* 1 mL of benzene. The resulting solution was irradiated at ∼254 nm at ambient temperature in a Rayonet reactor fitted with quartz glass mercury vapor lamps. After *ca.* 1 h, the polyyne precursor (20 mg, 1 equiv.) was added. This reaction mixture was then irradiated overnight under the same conditions. The reaction mixture was passed through a silica gel plug and eluted with EtOAc. The solvent was removed under reduced pressure, and the crude material was purified using MPLC with the elution solvent mixture indicated for each compound.

### 4.3. General procedure for photochemical synthesis of aminocarbazole derivatives from Kobayashi generated benzynes and azobenzene derivatives

The azobenzene derivative (3 equiv.) was placed in a quartz glass tube and dissolved in *ca.* 2 mL of acetonitrile. The resulting solution was irradiated at 254 nm at ambient temperature in a Rayonet reactor fitted with quartz glass mercury vapor lamps. After *ca.* 1 h, the *o*-trimethylsilylphenyl triflate (“Kobayashi precursor,” 50 mg, 1 equiv.) and cesium fluoride (3 equiv.) were added. The final reaction mixture was then irradiated overnight while being stirred (magnetically) under the same photochemical conditions. The acetonitrile was evaporated under reduced pressure and the residue was partitioned between DCM and water. The organic layer was washed with brine, dried over Na_2_SO_4_, filtered, and concentrated. The residue was purified using MPLC with the elution solvent mixture indicated for each compound.

## Data availability

The data upon which the discussion and conclusions in this manuscript are based can be found in the ESI file.†

## Author contributions

D. S. S. performed all the experimental and computational work; both authors interpreted the data and co-wrote the manuscript.

## Conflicts of interest

There are no conflicts to declare.

## Supplementary Material

SC-014-D3SC02253F-s001

SC-014-D3SC02253F-s002
