# Supplementary material for: The contrasting reactivity of trans- vs. cis-azobenzenes (ArN[double bond, length as m-dash]NAr) with benzynes
Source: Chem Sci. 2023 Jun 6;14(24):6730–7. doi: 10.1039/d3sc02253f (PMC10284128; doi:10.1039/d3sc02253f)
Supplement: SC-014-D3SC02253F-s001 [file SC-014-D3SC02253F-s001.pdf]

Electronic Supplementary Material (ESI) for Chemical Science.
This journal is © The Royal Society of Chemistry 2023

Electronic Supporting Information for

The Contrasting Reactivity of *trans*- vs. *cis*-Azobenzenes (ArN=NAr) with Benzynes

Dorian S. Sneddon and Thomas R. Hoye*

Department of Chemistry, University of Minnesota, 207 Pleasant St. SE, Minneapolis, Minnesota 55455

Email: hoye@umn.edu

Table of Contents

I. General Experimental Protocols S3

II. Setup Used for Irradiation Experiments S4

III. General Experimental Procedures S5

IV. Experimental Procedures and Characterization Data for All New Compounds S6–S23

3a and **4a** S6–S7

3b S8

3c S9

3d S10

11a S11

11b S12

11c S13

11d S14

11e S15–S16

11f S17

***cis*-1a** S18

7a S19–S21

7c S22

7d S23

12–15 .... S24–S29

V. Variable Temperature NMR Analysis of the Carbazole 11d S30

VI. Discussion of Computational Results S31–S91

VII. X-ray Data for 3a and 11b S92–S97

VIII. References for Supporting Information S98–S99

IX.Copies of 1D and 2D NMR Spectra S100–S190

3a ^{1}H/^{13}C/nOe/COSY/HSQC/HMBC S101–S106

4a $^{1}H/^{13}C$/COSY/NOESY/HSQC/HMBC S107–S112
3b ^{1}H/NOESY/HSQC/HMBC S113–S116
3c $^{1}H/^{13}C$/COSY/NOESY/HSQC/HMBC S117–S122
3d $^{1}H/^{13}C$/COSY/NOESY/HSQC/HMBC S123–S128
11a $^{1}H/^{13}C$/NOESY/COSY/HSQC/HMBC S129–S134
11b $^{1}H/^{13}C$/NOESY/COSY/HSQC/HMBC S135–S140
11c $^{1}H/^{13}C$/NOESY/COSY/HSQC/HMBC S141–S146
11d $^{1}H/^{13}C$/NOESY/COSY/HSQC/HMBC S147–S152
11e $^{1}H/^{13}C$/NOESY/COSY/HSQC/HMBC ($CDCl_3$) S153–S158
11e $^{1}H/^{13}C$/NOESY/COSY/HSQC/HMBC (C_6D_6) S159–S164
11f $^{1}H/^{13}C$/NOESY/COSY/HSQC/HMBC S165–S170
***cis*-1a** $^{1}H/^{13}C$ S171–S172
7a $^{1}H/^{13}C$/NOESY/COSY/HSQC/HMBC S173–S178
7c $^{1}H/^{13}C$ S179–S180
7d $^{1}H/^{13}C$ S181–S182
12 $^{1}H/^{13}C$ ($CDCl_3$) S183–S184
12 ^{1}H (C_6D_6) S185
13 $^{1}H/^{13}C$ ($CDCl_3$) S186–S187
13 ^{1}H (C_6D_6) S188
14 ^{1}H ($CDCl_3$) S189
14 ^{1}H (C_6D_6) S190

I. General Experimental Protocols

"**^{13}C and ^{1}H NMR spectra** were recorded on a Bruker Avance III (HD-500) spectrometer. Chemical shifts for spectra in $CDCl_3$ are referenced to TMS at δ 0.00 ppm. A non-first order multiplet, doublet, or doublet of doublets in a ^{1}H NMR spectrum are denoted as 'nfom', 'nfod', or 'nfodd,' respectively. Multiplets are described by: chemical shift (ppm) [multiplicity, coupling constant(s) in Hz, integral value to the nearest integer, and assignment of the environment within the structure by indicating neighboring atoms or by numbering of the carbon atom to which the proton is attached]. Analysis of coupling constants was done using methods published previously.[1,2] ^{13}C NMR chemical shifts are those measured in the 1D spectrum. Carbon chemical shifts in $CDCl_3$ are referenced to δ 77.16 ppm.

Infrared spectra were taken on a Bruker Alpha II Spectrometer in the attenuated total reflectance (ATR) mode. Absorption maxima are given in cm^{-1}. The samples were prepared as thin films made by evaporation of a DCM solution on a diamond window.

High-resolution **mass spectrometry** (HRMS) was performed in ESI-TOF mode on a Thermo Orbitrap Velos instrument having a mass accuracy of ≤3 ppm. Pierce™ LTQ was used as an external calibrant. The samples were injected directly into the ion source.

Medium pressure liquid **chromatography** (MPLC) was used to purify most new compounds. Hand-packed silica gel columns (Teledyne RediSep Rf Gold®; normal-phase, 20–40 µm, 60 Å pore size) were used. The apparatus was constructed with a HPLC pump (Waters model 510), differential refractive index detector (Waters R401), and UV detector (Gilson 111 UV). Preparative flash chromatography was done on Agela silica gel (230–400 mesh). Thin layer chromatography (TLC) was carried out using silica-gel coated, aluminum-backed plates that were visualized first by UV light and, then, by staining with a solution of $KMnO_4$ and heating.

Reaction temperatures refer to the temperatures of an external heating oil bath or block heater. HDDA reactions, including reactions at temperatures higher than the that of the boiling point of the solvent, were done in a screw-top culture tube that was capped with an inert Teflon®-lined closure."[3]

Melting points were recorded on a Mel-Temp® (Laboratory Devices) apparatus as a range with the first number representing the initial point of liquification (or degradation) of the crystal and the final point being that at which full liquification or degradation was observed. A Bristoline Bristolscope microscope with a polarizing filter was used to assess crystallinity of solid samples.

Poly-yne substrates **2**[4], **10a**[5], and **10b**[6] were synthesized according to reported methods.

II. Setup Used for Irradiation Experiments

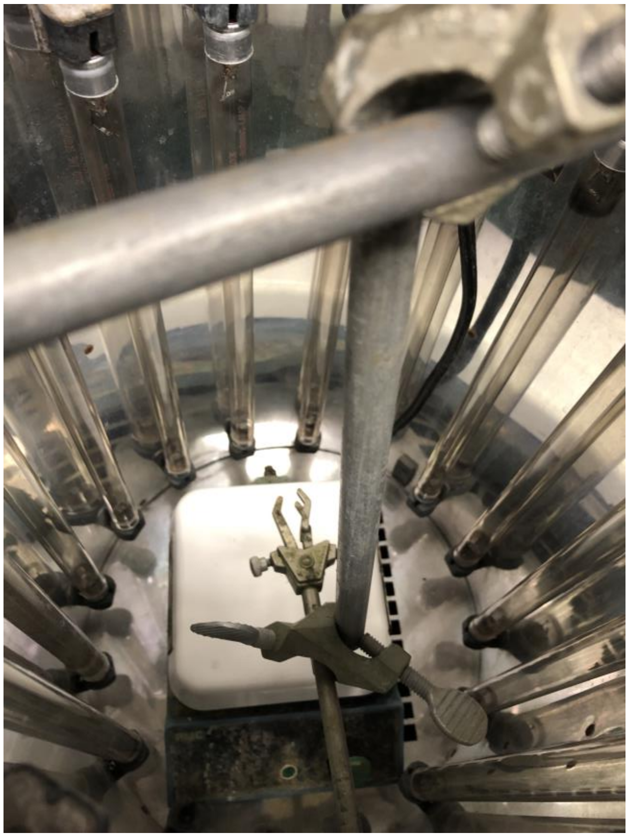

Figure S1. Rayonet chamber fitted with 254 nm quartz glass mercury vapor bulbs and integrated clamp/stir plate (used for mixing of the heterogeneous "Kobayashi" reaction mixtures).

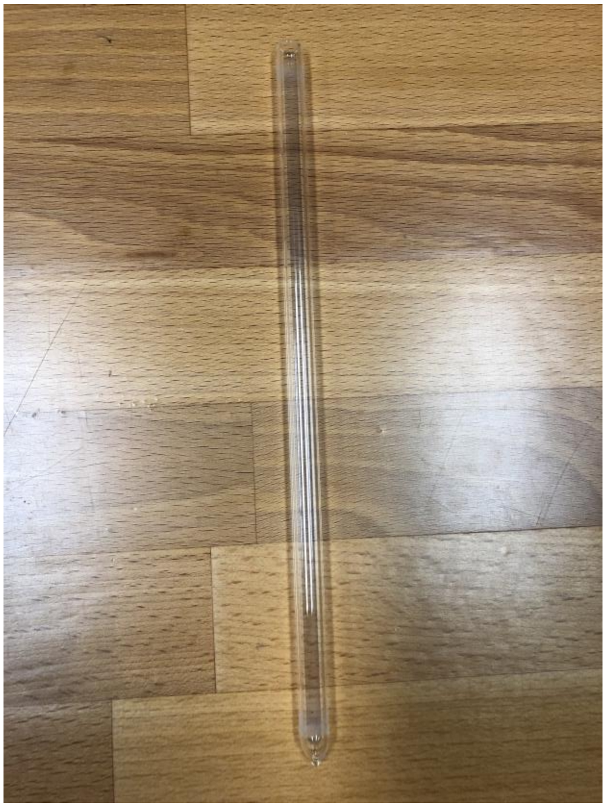

Figure S2. Representative quartz glass tube used for photochemical studies described in this manuscript.

III. Preparation procedures and characterization data for all compounds

A. General procedure for the thermal synthesis of phenazine derivatives from HDDA-generated benzynes and azobenzene derivatives

The polyyne precursor (1 equiv) and the azobenzene derivative (10 equiv) were combined in a screw-capped culture tube. 1,2-Dichloroethane was added to bring the solution to an initial concentration of the polyyne of 0.01 M. The resulting solution was placed in an oil bath maintained at 90 °C for ca. 16 h. Subsequently, the reaction mixture was passed through a silica gel plug and eluted with EtOAc. The volatiles were removed under reduced pressure, and the crude residue was purified using MPLC with the elution solvent mixture indicated for each compound.

B. General procedure for photochemical synthesis of aminocarbazole derivatives from HDDA-generated benzynes and azobenzene derivatives

The azobenzene derivative (3 equiv) was placed in a quartz glass tube and dissolved in ca. 1 mL of benzene. The resulting solution was irradiated at ~254 nm at ambient temperature in a Rayonet reactor fitted with quartz glass mercury vapor lamps. After ca. 1 h, the polyyne precursor (20 mg, 1 equiv) was added. This reaction mixture was then irradiated overnight under the same conditions. The reaction mixture was passed through a silica gel plug and eluted with EtOAc. The solvent was removed under reduced pressure, and the crude material was purified using MPLC with the elution solvent mixture indicated for each compound.

C. General procedure for photochemical synthesis of aminocarbazole derivatives from Kobayashi generated benzynes and azobenzene derivatives

The azobenzene derivative (3 equiv) was placed in a quartz glass tube and dissolved in ca. 2 mL of acetonitrile. The resulting solution was irradiated at 254 nm at ambient temperature in a Rayonet reactor fitted with quartz glass mercury vapor lamps. After ca. 1 h, the *o*-trimethylsilylphenyl triflate ("Kobayashi precursor," 50 mg, 1 equiv) and cesium fluoride (3 equiv) were added. The final reaction mixture was then irradiated overnight while being stirred (magnetically) under the same photochemical conditions. The acetonitrile was evaporated under reduced pressure and the residue was partitioned between DCM and water. The organic layer was washed with brine, dried over Na_2SO_4, filtered, and concentrated. The residue was purified using MPLC with the elution solvent mixture indicated for each compound.

IV. Experimental Procedures and Characterization Data for All New Compounds

10,11-Dimethoxy-6-methyl-13-phenyl-7-(trimethylsilyl)-5,13-dihydro-8H-indeno[1,2-a]phenazin-8-one (3a)
and
10,11-Dimethoxy-6-methyl-5-phenyl-7-(trimethylsilyl)-5,13-dihydro-8H-indeno[1,2-a]phenazin-8-one (4a)

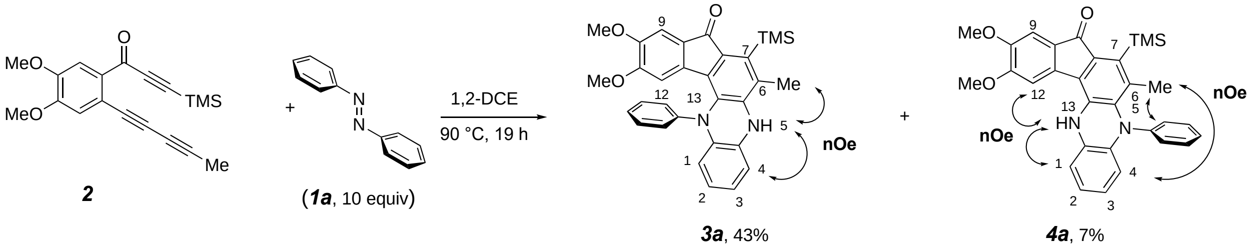

NMR Ratio: 4 : 1

Following general procedure A, 1-(4,5-dimethoxy-2-(penta-1,3-diyn-1-yl)phenyl)-3-(trimethylsilyl)prop-2-yn-1-one (**2**, 29 mg, 0.091 mmol, 1 equiv), (*E*)-1,2-diphenyldiazene (**1a**, 166 mg, 0.910 mmol, 10 equiv), and 1,2-dichloroethane (5 mL) were used to prepare the phenazine derivatives **3a** and **4a**. Purification by MPLC (3:1 hexanes:EtOAc) yielded, in order of elution, the phenazine derivatives **3a** (20 mg, 0.039 mmol, 43%), as an orange crystalline solid, and **4a** (3 mg, 0.006 mmol, 7%), as a dark red crystalline solid. The latter was repurified by HPLC to obtain a sample suitably pure for characterization.

Data for the phenazine derivative 3a, the major isomer:

^{1}H NMR ($CDCl_3$, 500 MHz): δ 7.44 (dd, *J* = 7.7, 1.1 Hz, 1H, *H1*), 7.19 (s, 1H, *H12*), 7.16 (ddd, *J* = 7.7, 7.7, 1.5 Hz, 1H, *H3*), 7.13 (nfom, 2H, PhH_m), 7.11 (s, 1H, *H9*), 7.10 (ddd, *J* = 7.7, 7.7, 1.4 Hz, 1H, *H2*), 7.06 (nfom, 2H, PhH_o), 6.90 (dd, *J* = 7.8, 1.5 Hz, 1H, *H4*), 6.89 (tt, *J* = 7.7, 1.2 Hz, 1H, PhH_p), 6.25 (br s, 1H, N*H*), 3.88 (s, 3H, C10-OCH_3), 3.69 (s, 3H, C11-OCH_3), 2.35 (s, 3H, ArCH_3), and 0.46 [s, 9H, Si(CH_3)$_3$].

^{13}C{^{1}H} NMR ($CDCl_3$, 126 MHz): δ 193.7 (C8), 153.5 (C11), 149.2 (C10), 148.0 (PhC_{ipso}), 145.8 (C5a), 140.7 (C4a), 140.3 (C7), 138.8, 136.6, 134.5, 131.3 (C13a), 129.3 (PhC_m), 127.9, 127.4 (C1), 126.6 (C3), 126.0 (C6), 125.5 (C12c), 122.1 (C2), 121.8 (PhC_p), 116.1 (PhC_o), 115.9 (C4), 107.8 (C12), 106.2 (C9), 56.2, 56.1, 17.4, and 3.1.

based on NOESY
based on HSQC
based on HMBC

HRMS (ESI-TOF) *m/z*: $[M+H^+–H_2]^+$ Calcd for $C_{31}H_{29}N_2O_3Si$ 505.1942; Found 505.1913.

IR (neat): 3369, 3061, 3000, 2943, 2898, 2835, and 1694 cm^{-1}.

mp: 165–170 °C (decomposition prior to melting).

Data for the phenazine derivative 4a, the minor isomer:

^{1}H NMR ($CDCl_3$, 500 MHz): δ 7.42 (dd, *J* = 7.6, 1.1 Hz, 1H, *H4*), 7.23 (s, 1H, *H9*), 7.22–7.18 (nfom, 2H, PhH_m), 7.12 (ddd, *J* = 7.6, 7.6, 1.3 Hz, 1H, *H2*), 7.06 (ddd, *J* = 7.7, 7.7, 1.3 Hz, 1H, *H3*), 7.00–6.96 (nfom, 2H, PhH_o), 6.96 (tt, *J* = 7.3, 1.1 Hz, 1H, PhH_p), 6.88 (s, 1H, *H12*), 6.86 (dd, *J* = 7.7, 1.2 Hz, 1H, *H1)*, 6.19 (br s, 1H, N*H*), 4.04 (s, 3H, C11OCH_3), 3.93 (s, 3H, C10OCH_3), 2.30 (s, 3H, ArCH_3), and 0.40 [s, 9H, Si(CH_3)$_3$].

^{13}C{^{1}H} NMR ($CDCl_3$, 126 MHz): δ 193.6 (C8), 153.9 (C11), 148.9 (C10), 148.4, 141.0 (C6), 139.8 (C13a), 138.7, 138.4, 137.4 (C12a), 135.5 (C7), 134.9 (C5a), 132.2 (C4a), 129.3 (Ph$_m$), 127.4 (C8a), 127.2 (C4), 126.2 (C2), 125.8, 122.3 (C3), 122.1 (Ph$_p$), 117.7 (Ph$_o$), 115.4 (C1), 107.9 (C9), 104.8 (C12), 56.8 (C11OCH_3), 56.4 (C10OCH_3), 19.6 (C6CH_3), and 3.0 (TMS).

based on NOESY
based on HSQC
based on HMBC

HRMS (ESI-TOF) *m/z*: $[M+H^+–H_2]^+$ Calcd for $C_{31}H_{29}N_2O_3Si$ 505.1942; Found 505.1914.

IR (neat): 3402, 3059, 2971, 2940, 2899, 2837, and 1699 cm^{-1}.

mp: 165–170 °C (decomposition prior to melting).

2-Chloro-13-(4-chlorophenyl)-10,11-dimethoxy-6-methyl-7-(trimethylsilyl)-5,13-dihydro-8H-indeno[1,2-a]phenazin-8-one (3b)

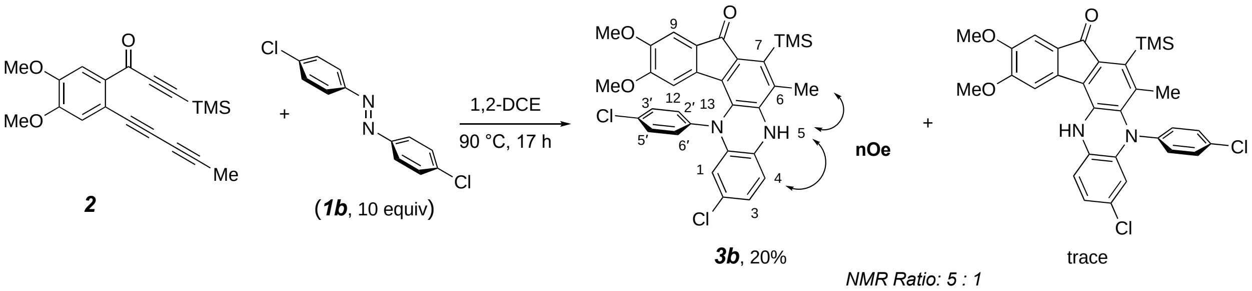

(*E*)-1,2-*bis*(4-Chlorophenyl)diazene (**1b**) was prepared according to a literature procedure.[7] Following general procedure A, 1-(4,5-dimethoxy-2-(penta-1,3-diyn-1-yl)phenyl)-3-(trimethylsilyl)prop-2-yn-1-one (**2**, 20 mg, 0.062 mmol, 1 equiv), (*E*)-1,2-*bis*(4-chlorophenyl)diazene (**1b**, 155 mg, 0.617 mmol, 10 equiv), and 1,2-dichloroethane (5 mL) were used to prepare the phenazine **3b**. Purification by MPLC (3:1 hexanes:EtOAc) yielded coeluting phenazine derivatives (7 mg, 0.01 mmol, 20%). A purer sample of phenazine **3b** for characterization was obtained by HPLC as an orange crystalline solid. [The ^{1}H NMR spectrum of the crude product contains resonances (in an ca. 5 : 1 ratio of the major to minor constituents). The minor set of resonances are suggestive of it being the N5-arylated isomer, although this compound was not obtained following the HPLC purification.]

Data for phenazine derivative 3b, the major regioisomer:

^{1}H NMR ($CDCl_3$, 500 MHz): δ 7.37 (d, *J* = 2.3 Hz, 1H, *H1*), 7.16 (dd, *J* = 8.5, 2.3 Hz, 1H, *H3*), 7.124 (s, 1H, *H9*), 7.116 (s, 1H, *H12*), 7.10 (nfod, J_{app} = 8.4 Hz, 2H, *H3′* and *H5′*), 6.96 (nfod, J_{app} = 9.2 Hz, 2H, *H2′* and *H6′*), 6.85 (d, *J* = 8.4 Hz, 1H, *H4*), 6.24 (br s, 1H, N*H*), 3.89 (s, 3H, OCH_3), 3.74 (s, 3H, OCH_3′), 2.34 (s, 3H, ArCH_3), and 0.46 [s, 9H, Si(CH_3)$_3$].

^{13}C{^{1}H} NMR ($CDCl_3$, 126 MHz): δ 193.1 (C8), 153.2 (C11), 149.3 (C10), 146.3 (PCP-C_{ipso}), 144.6 (C5a), 140.7 (C7), 139.0 (x2, C4a and C12a or C12b), 136.0 (C12a or C12b), 131.7 (C13a), 129.0 (PCP-C_m), 127.0 (PCP-C_p), 126.9 (C1), 126.6 (x2, C2 and C3), 126.1 (C6), 124.8 (C12c), 117.3 (PCP-C_o), 116.5 (C4), 106.8 (x2, C9 and C12), 56.1 (x2, C11-OCH_3 and C10-OCH_3), 17.1 (ArCH_3), and 2.9 [Si(CH_3)$_3$]. (*chemical shifts read from the HSQC and HMBC spectra*; neither *C7a* or *C8a* was definitively identified).

based on NOESY
based on HSQC
based on HMBC

HRMS (ESI-TOF) *m/z*: $[M+H^+–H_2]^+$ Calcd for $C_{31}H_{27}Cl_2N_2O_3Si$ 573.1163; Found 573.1137.

IR (neat): 3362, 3060, 3004, 2942, 2901, 2836, and 1696 cm^{-1}.

mp: 92–95 °C.

10,11-Dimethoxy-2,6-dimethyl-13-(*p*-tolyl)-7-(trimethylsilyl)-5,13-dihydro-8H-indeno[1,2-a]phenazin-8-one (3c)

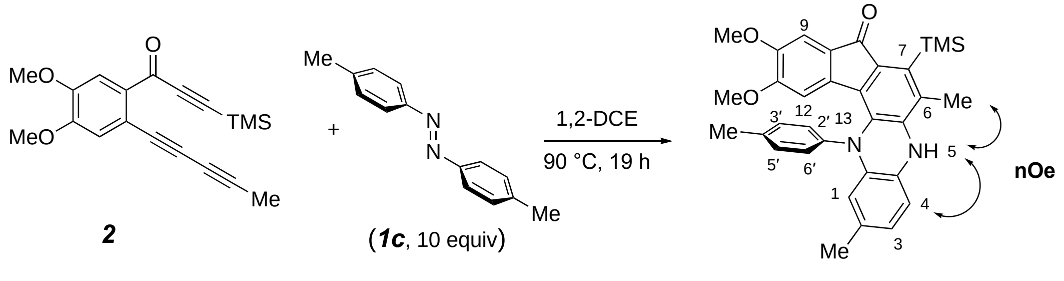

3c, 56%

(*E*)-1,2-Di-*p*-tolyldiazene (**1c**) was prepared according to a literature procedure.[7]
Following general procedure A, 1-(4,5-dimethoxy-2-(penta-1,3-diyn-1-yl)phenyl)-3-(trimethylsilyl)prop-2-yn-1-one (**2**, 20 mg, 0.062 mmol, 1 equiv), (*E*)-1,2-di-*p*-tolyldiazene (**1c**, 129 mg, 0.61 mmol, 10 equiv), and 1,2-dichloroethane (5 mL) were used to prepare the phenazine derivative **3c**. Purification by MPLC (3:1 hexanes:EtOAc) yielded phenazine derivative **3c** (19 mg, 0.035 mmol, 56%) as a red crystalline solid. (The ^{1}H NMR spectrum for this sample contains ca. 6% of resonances that are suggestive of the presence of the N5-arylated isomer.)

Data for the phenazine derivative 3c:

^{1}H NMR ($CDCl_3$, 500 MHz): δ 7.25 (s, 1H, *H12*), 7.21 (d, *J* = 1.9 Hz, 1H, *H1*), 7.10 (s, 1H, *H9*), 6.97 (nfod, J_{app} = 8.8 Hz, 2H, *H2′* and *H6′*), 6.94–6.92 (m, *H3*, 2H, *H3′*, and *H5′*), 6.75 (d, *J* = 8.0 Hz, 1H, *H4*), 6.15 (br s, 1H, N*H*), 3.87 (s, 3H, C10OCH_3), 3.76 (s, 3H, C11OCH_3), 2.38 (s, 3H, C2CH_3), 2.32 (s, 3H, C6CH_3), 2.19 (s, 3H, C4'CH_3), and 0.45 [s, 9H, Si(CH_3)$_3$].

based on NOESY

^{13}C{^{1}H} NMR ($CDCl_3$, 126 MHz): δ 193.7 (C8), 153.4 (C11), 149.1 (C10), 146.3 (C1'), 146.0 (C5a), 140.0 (C7), 138.6, 137.8 (C4a), 136.7 (C12a), 134.2, 131.9 (C2 or C13a), 131.8 (C2 or C13a), 131.5 (C4'), 129.8 (C3' and C5'), 127.9, 127.5 (C1), 126.9 (C3), 126.1, 125.6 (C12c), 117.0 (C2' and C6'), 115.5 (C4), 107.8 (C12), 106.2 (C9), 56.22, 56.21, 21.1 (C2*C*H_3), 20.6 (C4'*C*H_3), 17.4 (C6*C*H_3), and 3.1.

based on NOESY
based on HSQC
based on HMBC

HRMS (ESI-TOF) *m/z*: $[M+H^{+}–H_2]^{+}$ Calcd for $C_{33}H_{33}N_2O_3Si$ 533.2255; Found 533.2244.

IR (neat): 3370, 3001, 2943, 2921, 2900, 2835, and 1691 cm^{-1}.
mp: 160–165 °C (decomposition prior to melting).

2,10,11-Trimethoxy-13-(4-methoxyphenyl)-6-methyl-7-(trimethylsilyl)-5,13-dihydro-8H-indeno[1,2-a]phenazin-8-one (3d)

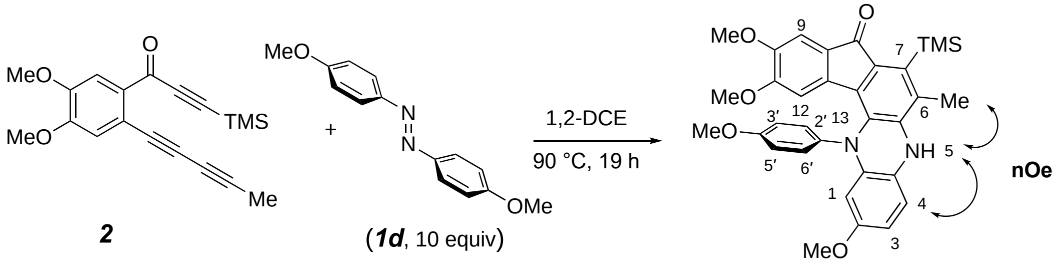

2 (***1d***, 10 equiv) ***3d***, 59%

(*E*)-1,2-*bis*(4-Methoxyphenyl)diazene (**1d**) was prepared according to a literature procedure.[7] Following general procedure A, 1-(4,5-dimethoxy-2-(penta-1,3-diyn-1-yl)phenyl)-3-(trimethylsilyl)prop-2-yn-1-one (**2**, 20 mg, 0.061 mmol, 1 equiv), (*E*)-1,2-*bis*(4-methoxyphenyl)diazene (**1d**, 149 mg, 0.61 mmol, 10 equiv), and 1,2-dichloroethane (5 mL) were used to prepare the phenazine derivative **3d**. Purification by MPLC (2:1 hexanes:EtOAc) yielded phenazine derivative **3d** (20 mg, 0.036 mmol, 59%), which was further purified by HPLC to obtain a sample well suited for characterization, as a red crystalline solid.

Data for the phenazine derivative 3d:

^{1}H NMR ($CDCl_3$, 500 MHz): δ 7.27 (s, 1H, *H12*), 7.09 (s, 1H, *H9*), 7.07 (nfod, J_{app} = 9.1 Hz, 2H, *H2′ and H6′*), 6.99 (d, *J* = 2.7 Hz, 1H, *H1*), 6.76 (d, *J* = 8.6 Hz, 1H, *H4*), 6.71 (nfod, J_{app} = 9.1 Hz, 2H, *H3′ and H5′*), 6.69 (dd, *J* = 8.6, 2.7 Hz, 1H, *H3*), 6.06 (br s, 1H, N*H*), 3.87 (s, 3H, C10OCH_3), 3.83 (s, 3H, C2OCH_3), 3.79 (s, 3H, C11OCH_3), 3.69 (s, 3H, C4'OCH_3), 2.32 (s, 3H, ArCH_3), and 0.45 [s, 9H, Si(CH_3)$_3$].

based on NOESY

^{13}C{^{1}H} NMR ($CDCl_3$, 126 MHz): δ 193.5 (C8), 155.4 (C2), 155.3 (C4'), 153.3 (C11), 149.2 (C10), 145.8 (C5a), 142.6 (C1'), 140.1 (C7), 136.6, 133.8 (C4a), 133.6 (C13a), 128.0, 126.1 (C12c), 125.3 (C6), 124.5, 119.4 (C2' and C6'), 115.7 (C4), 114.5, 114.3 (C3' and C5'), 113.5 (C1), 110.9 (C3), 107.8 (C12), 106.2 (C9), 56.3 (C10O*C*H_3 or C11O*C*H_3), 56.2 (C10O*C*H_3 or C11O*C*H_3), 56.1 (C2O*C*H_3), 55.6 (C4'O*C*H_3), 17.4, and 3.1.

based on NOESY
based on HSQC
based on HMBC

HRMS (ESI-TOF) *m/z*: $[M+H^+–H_2]^+$ Calcd for $C_{33}H_{33}N_2O_5Si$ 565.2153; Found 565.2149.

IR (neat): 3372, 2999, 2947, 2902, 2834, and 1693 cm^{-1}.

mp: 105–108 °C.

Dimethyl 5-(4-methoxyphenyl)-4-((4-methoxyphenyl)ethynyl)-10-(phenylamino)-3,10-dihydrocyclopenta[a]carbazole-2,2(1H)-dicarboxylate (11a)

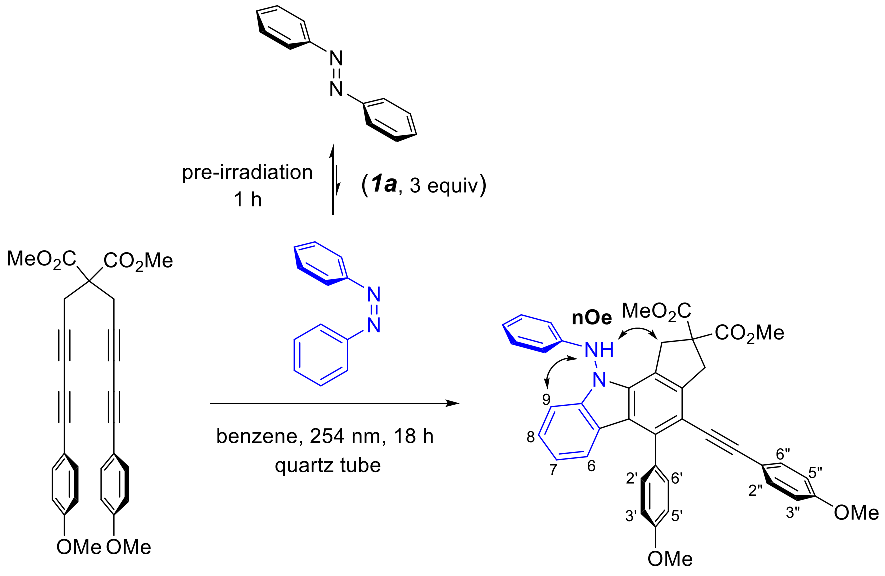

10a ***11a***, 60%

Following general procedure B, dimethyl 2,2-*bis*(5-(4-methoxyphenyl)penta-2,4-diyn-1-yl)malonate (**10a**, 20 mg, 0.043 mmol, 1 equiv), (*E*)-1,2-diphenyldiazene (**1a**, 23 mg, 0.13 mmol, 3 equiv), and benzene (1 mL) were used to prepare the carbazole **11a**. Purification by MPLC (3:1 hexanes:EtOAc) yielded **11a** (17 mg, 0.026 mmol, 60%) as a pale yellow amorphous solid.

Data for the carbazole derivative 11a:

^{1}H NMR ($CDCl_3$, 500 MHz): δ 7.53 (br s, 2H, *H2′* and *H6′*), 7.26 (ddd, *J* = 8.2, 6.9, 1.2 Hz, 1H, *H8*), 7.212 (nfom, 2H, PhH_m), 7.208 (ddd, *J* = 8.2, 1.0, 1.0 Hz, 1H, *H9*), 7.19 (nfod, J_{app} = 8.8 Hz, 2H, *H2″* and *H6″*), 7.13 (ddd, *J* = 8.0, 1.0, 1.0 Hz, 1H, *H6*), 7.10 (nfod, J_{app} = 9.0 Hz, 2H, *H3′* and *H5′*), 6.96 (ddd, *J* = 8.0, 6.9, 1.2 Hz, 1H, *H7*), 6.91 (tt, *J* = 7.3, 1.1 Hz, 1H, PhH_p), 6.79 (nfod, J_{app} = 8.8 Hz, 2H, *H3″* and *H5″*), 6.66 (s, 1H, N*H*), 6.61 (nfod, J_{app} = 7.4 Hz, 2H, PhH_o), 4.16 (br s, 1H, C1H_a), 3.96 (s, 3H, C4′OCH_3), 3.92 (br s, 1H, C3H_a), 3.80 (s, 3H, C4″OCH_3), 3.76 (br s, 1H, C3H_b), and 3.80–3.55 (br m, 7H, COOCH_3, COOCH_3′, C1H_b).

^{13}C{^{1}H} NMR ($CDCl_3$, 126 MHz): δ 172.3 (br s, x2), 159.4, 159.3, 147.2, 141.8, 140.4, 138.7, 136.6, 132.8, 131.7, 131.3, 129.7, 125.9, 122.4, 122.0, 121.4, 120.5, 119.9, 119.5, 116.3, 114.0, 113.8, 112.8, 112.4, 108.9, 94.7, 86.5, 60.3, 55.5, 55.4, 53.2 (x2), 41.3, and 39.0.

HRMS (ESI-TOF) *m/z*: $[M+H^+–H_2]^+$ Calcd for $C_{41}H_{33}N_2O_6$ 649.2333; Found 649.2347.

IR (neat): 3326, 3050, 3001, 2952, 2905, 2836, 1731, and 1171 cm^{-1}.

Dimethyl 7-chloro-10-((4-chlorophenyl)amino)-5-(4-methoxyphenyl)-4-((4-methoxyphenyl)ethynyl)-3,10-dihydrocyclopenta[a]carbazole-2,2(1H)-dicarboxylate (11b)

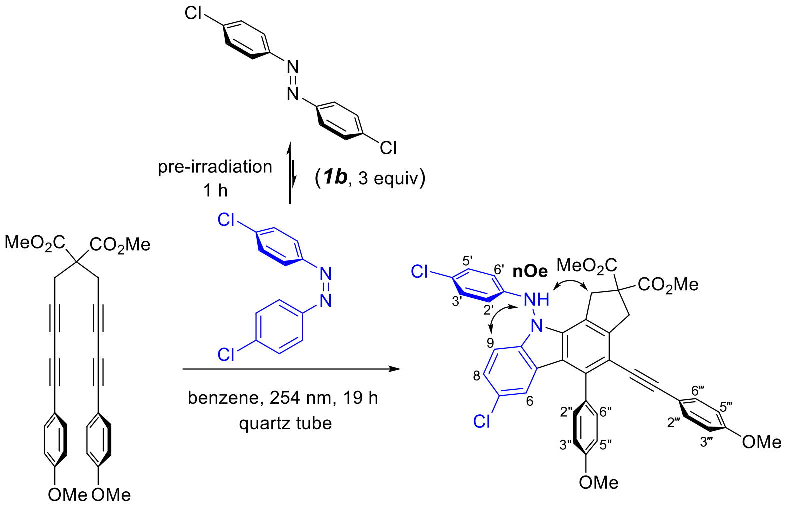

10a ***11b***, 58%

Following general procedure B, dimethyl 2,2-*bis*(5-(4-methoxyphenyl)penta-2,4-diyn-1-yl)malonate (**10a**, 20 mg, 0.043 mmol, 1 equiv), (*E*)-1,2-*bis*(4-chlorophenyl)diazene (**1b**, 32 mg, 0.13 mmol, 3 equiv), and benzene (1 mL) were used to prepare the carbazole **11b**. Purification by MPLC (3:1 hexanes:EtOAc) yielded **11b** (18 mg, 0.025 mmol, 58%) as a pale yellow crystalline solid. See the information on page S93 for a description of the method used to prepare the sample of crystalline material used for the X-ray diffraction analysis that confirmed the structure assignment of **11b**.

Data for the carbazole derivative 11b:

^{1}H NMR ($CDCl_3$, 500 MHz): δ 7.50 (br s, 2H, *H2″* and *H6″*), 7.23 (dd, *J* = 8.6, 2.0 Hz, 1H, *H8*), 7.19 (nfod, J_{app} = 8.8 Hz, 2H, *H2‴* and *H6‴*), 7.17 (nfod, *J* = 8.9 Hz, 2H, *H3′* and *H5′*), 7.123 (nfod, J_{app} = 8.6 Hz, 2H, *H3″* and *H5″*), 7.117 (d, *J* = 8.7, Hz, 1H, *H9*), 7.10 (d, *J* = 1.9 Hz, 1H, *H6*), 6.80 (nfod, J_{app} = 8.9 Hz, 2H, *H3‴* and *H5‴*), 6.72 (s, 1H, N*H*), 6.55 (nfod, J_{app} = 8.9 Hz, 2H, *H2′* and *H6′*), 4.13 (br s, 1H, C1H_a), 3.96 (s, 3H, C4″OCH_3), 3.91 (br s, 1H, C3H_a), 3.85 (br s, 1H, C3H_b), 3.82–3.65 (overlapped m, 7H, COOCH_3, COOCH_3′, and C1H_b), and 3.80 (s, 3H, C4‴OCH_3).

^{13}C{^{1}H} NMR ($CDCl_3$, 126 MHz): δ 172.1 (only seen in HMBC), 159.7, 159.5, 145.5, 142.7, 139.0, 138.6, 136.7, 132.8, 131.1, 130.9, 129.8, 127.0, 126.4, 126.21, 126.18, 123.1, 122.2, 119.9, 118.7, 116.0, 114.8, 114.0, 113.9, 113.2, 109.8, 95.1, 86.1, 60.2, 55.6, 55.5, 53.3 (br, 2x), 41.3, and 38.9. [one carbon resonance was not observed, likely a result of dynamic inversion of N10 (cf. broadening seen in ^{1}H NMR spectrum)]

HRMS (ESI-TOF) *m/z*: $[M+H^+]^+$ Calcd for $C_{41}H_{33}{}^{35}Cl_2N_2O_6$ 719.1711; Found 719.1692.

IR (neat): 3330, 3002, 2953, 2934, 2910, 2837, 1732, and 1171 cm^{-1}.

mp: 137–140 °C.

Dimethyl 5-(4-methoxyphenyl)-4-((4-methoxyphenyl)ethynyl)-7-methyl-10-(*p*-tolylamino)-3,10-dihydrocyclopenta[a]carbazole-2,2(1H)-dicarboxylate (11c)

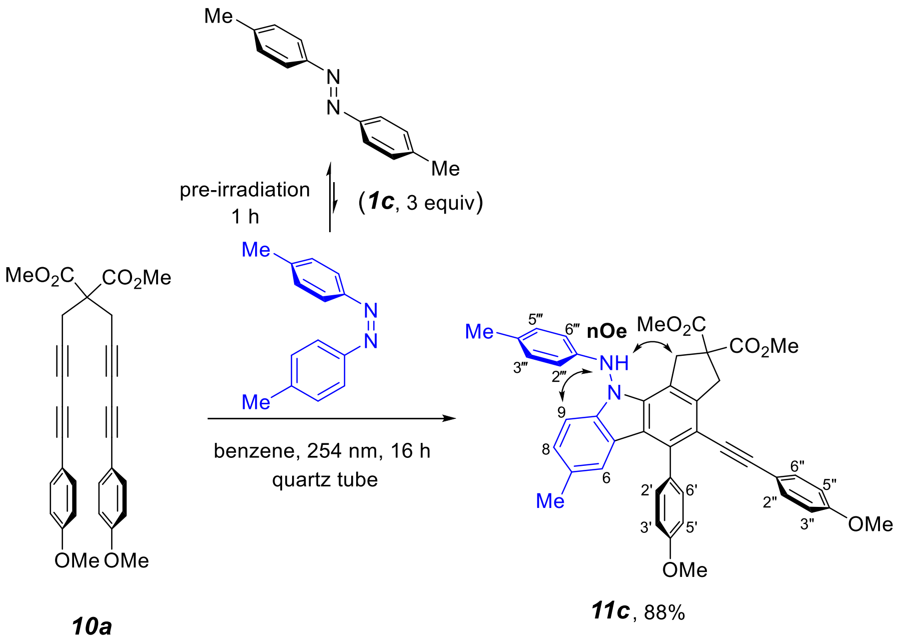

Following general procedure B, dimethyl 2,2-*bis*(5-(4-methoxyphenyl)penta-2,4-diyn-1-yl)malonate (**10a**, 20 mg, 0.043 mmol, 1 equiv), (*E*)-1,2-di-*p*-tolyldiazene (**1c**, 27 mg, 0.13 mmol, 3 equiv), and benzene (1 mL) were used to prepare the carbazole **11c**. Purification by MPLC (3:1 hexanes:EtOAc) yielded **11c** (26 mg, 0.038 mmol, 88%) as a pale yellow amorphous solid.

Data for the carbazole derivative 11c:

^{1}H NMR ($CDCl_3$, 500 MHz): δ 7.53 (br d, *J* = 7.4 Hz, 2H, *H2′* and *H6′*), 7.19 (nfod, J_{app} = 8.8 Hz, 2H, *H2″* and *H6″*), 7.10 (nfod, J_{app} = 8.9 Hz, 2H, *H3′* and *H5′*), 7.09 (dd, *J* = 8.2, 0.8 Hz, 1H, *H9*), 7.06 (dd, *J* = 8.3, 1.5 Hz, 1H, *H8*), 6.99 (nfod, J_{app} = 8.6 Hz, 2H, *H2‴* and *H6‴*), 6.93 (dd, *J* = 1.5, 0.9 Hz, 1H, *H6*), 6.79 (nfod, J_{app} = 8.8 Hz, 2H, *H3″* and *H5″*), 6.55 (br s, 1H, N*H*), 6.51 (nfod, J_{app} = 8.5 Hz, 2H, *H3‴* and *H5‴*), 4.17 (br s,1H, C1H_a), 3.96 (s, 3H, C4′OCH_3), 3.89 (br s, 2H, C3H_a and C3H_b), 3.79 (s, 3H, C4″OCH_3), 3.72 (br s, 7H, C1H_b, COOCH_3, and COOCH_3′), and 2.25 (s, 6H, C8CH_3 and C4‴C*H3*).

^{13}C{^{1}H} NMR ($CDCl_3$, 126 MHz): δ 172 (only discerned as an HMBC correlation), 159.4, 159.3, 145.0, 141.6, 138.62, 138.60, 137.0, 132.7, 131.8, 131.3, 130.6, 130.2, 129.7, 127.1, 122.5, 122.1, 119.9, 119.1, 116.4, 114.0, 113.7, 112.9, 112.1, 108.7, 94.5, 86.7, 60.3, 55.6, 55.4, 53.1 (br, 2x), 41.3, 39.0, 21.7, and 20.7.

HRMS (ESI-TOF) *m/z*: $[M+H^+–H_2]^+$ Calcd for $C_{43}H_{37}N_2O_6$ 677.2646; Found 677.2659.

IR (neat): 3327, 3000, 2952, 2837, 1732, and 1171 cm^{-1}.

Dimethyl 7-methoxy-5-(4-methoxyphenyl)-10-((4-methoxyphenyl)amino)-4-((4-methoxyphenyl)ethynyl)-3,10-dihydrocyclopenta[a]carbazole-2,2(1H)-dicarboxylate (11d)

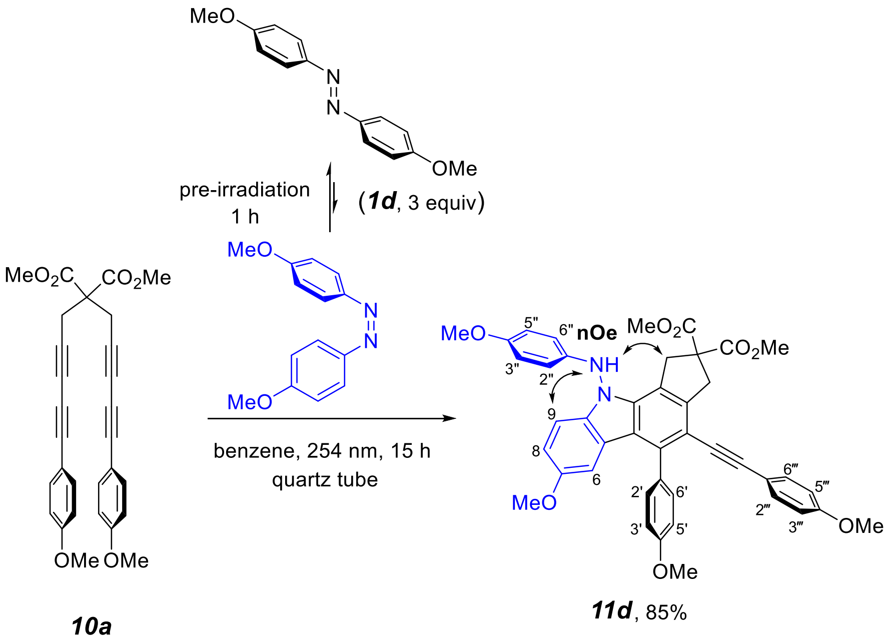

Following general procedure B, dimethyl 2,2-*bis*(5-(4-methoxyphenyl)penta-2,4-diyn-1-yl)malonate (**10a**, 20 mg, 0.043 mmol, 1 equiv), (*E*)-1,2-*bis*(4-methoxyphenyl)diazene (**1d**, 31 mg, 0.13 mmol, 3 equiv), and benzene (1 mL) were used to prepare the carbazole **11d**. Purification by MPLC (1:1 hexanes:EtOAc) yielded **11d** (26 mg, 0.036 mmol, 85%) as a pale yellow amorphous solid.

Data for the carbazole derivative 11d:

^{1}H NMR ($CDCl_3$, 500 MHz): δ 7.53 (nfod, J_{app} = 8.0 Hz, 2H, *H2′* and *H6′*), 7.19 (nfod, J_{app} = 8.9 Hz, 2H, *H2‴* and *H6‴*), 7.10 (nfod, J_{app} = 8.0 Hz, 2H, *H3′* and *H5′*), 7.09 (d, *J* = 8.7 Hz, 1H, *H9*), 6.87 (dd, *J* = 8.8, 2.5 Hz, 1H, *H8*), 6.79 (nfod, J_{app} = 8.8 Hz, 2H, *H3‴* and *H5‴*), 6.76 (nfod, J_{app} = 8.9 Hz, 2H, *H2″* and *H6″*), 6.61 (d, *J* = 2.5 Hz, 2H, *H6*), 6.55 (nfod, J_{app} = 9.0 Hz, 2H, *H3″* and *H5″*), 6.51 (s, 1H, N*H*), 3.93–3.92 (overlapped m, 4H, C4‴OCH_3 and C1H_a), 3.89 (br s, 2H, C3H_a and C3H_b), 3.80–3.79 (overlapped m, 4H, C4″OCH_3 and C2H_b), 3.72 (br s, 9H, C4′OCH_3, COOCH_3, and COOCH_3′), 3.56 (s, 3H, C9OCH_3).

^{13}C{^{1}H} NMR ($CDCl_3$, 126 MHz): δ 172.4 (x2), 159.4, 159.3, 154.6, 154.3, 141.6, 141.1, 138.6, 137.3, 135.2, 132.7, 131.6, 131.4, 122.5, 120.0, 119.0, 116.3, 115.1, 114.6, 114.1, 114.0, 113.7, 111.8, 109.7, 105.6, 94.5, 86.6, 60.2, 55.7, 55.62, 55.60, 55.4, 53.2 (2x), 41.3, and 39.1.

HRMS (ESI-TOF) *m/z*: $[M+H^+–H_2]^+$ Calcd for $C_{43}H_{37}N_2O_8$ 709.2544; Found 709.2560.

IR (neat): 3331, 2999, 2953, 2926, 2853, 2836, 1732, and 1171 cm^{-1}.

Dimethyl 5-(4-methoxyphenyl)-4-((4-methoxyphenyl)ethynyl)-6-methyl-10-(*m*-tolylamino)-3,10-dihydrocyclopenta[a]carbazole-2,2(1H)-dicarboxylate (major) and

Dimethyl 5-(4-methoxyphenyl)-4-((4-methoxyphenyl)ethynyl)-8-methyl-10-(*m*-tolylamino)-3,10-dihydrocyclopenta[a]carbazole-2,2(1H)-dicarboxylate (minor) (11e)

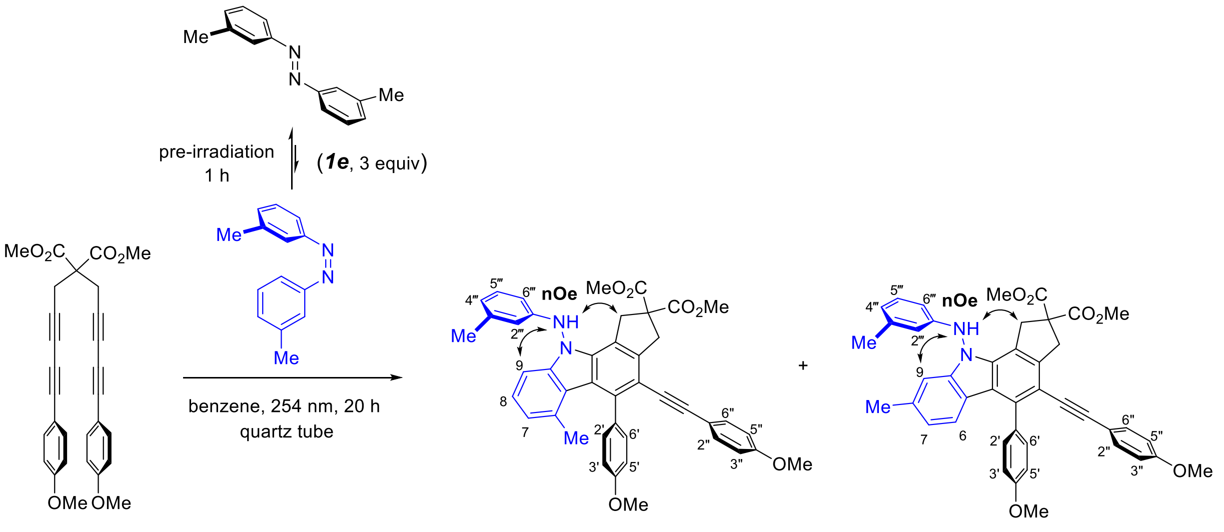

10a ***11e***, 58% (1.2:1 r.r.)

(*E*)-1,2-di-*m*-Tolyldiazene (**1e**) was prepared according to a literature procedure.[7]

Following general procedure B, dimethyl 2,2-*bis*(5-(4-methoxyphenyl)penta-2,4-diyn-1-yl)malonate (**10a**, 20 mg, 0.043 mmol, 1 equiv), (*E*)-1,2-di-*m*-tolyldiazene (**1e**, 27 mg, 0.13 mmol, 3 equiv), and benzene (1 mL) were used to prepare the carbazoles **11e**. Purification by MPLC (3:1 hexanes:EtOAc) yielded a coeluting mixture of the carbazoles **11e** (17 mg, 0.025 mmol, 58%) as a pale yellow amorphous solid. NMR spectral data indicate the sample to be of a 1.2:1 mixture of regioisomers.

The ^{1}H spectrum had a number of overlapping resonances. Copies of the ^{1}H and ^{13}C spectra in $CDCl_3$ are appended, but the line listings and assignments for characterization of these structures are taken from the battery of 1D and 2D spectra recorded in C_6D_6.

NMR spectral data for the major regioisomer:

^{1}H NMR (C_6D_6, 500 MHz): δ 7.60 (dd, *J* = 8.2, 1.8 Hz, 1H, *H2′* or *H6′*), 7.51 (dd, *J* = 8.2, 1.8 Hz, 1H, *H6′* or *H2′*), 7.23 (nfod, J_{app} = 8.9 Hz, 2H, *H2″* and *H6″*), 7.14 (dd, *J* = 8.1, 7.1 Hz, 1H, *H8*), 7.04–6.97 (overlapped with resonances from the minor isomer m, *H9*), 6.94–6.85 (overlapped m's, *H7, H3′*, *H5′*, and *H5‴*), 6.58–6.51 (overlapped m, *H4‴*), 6.53 (nfod, J_{app} = 8.9 Hz, 2H, *H3″* and *H5″*), 6.32 (br dd, *J* = 1.2, 1.2 Hz, 1H, *H2‴*), 6.24 (br ddd, *J* = 8.0, 2.3 Hz, 1H,

H6‴), 5.61 (s, 1H, N*H*), 4.64 (d, *J* = 16.6 Hz, 1H, C1H_a), 4.52–4.31 (overlapped m's, C3H_a and C3H_b), 4.05 (d, *J* = 16.6 Hz, 1H, C1H_b), 3.37 (s, 3H, C4′OCH_3), 3.32 [br s, 3H, (COOCH_3)$_2$], 3.17 (s, 3H, C4″OCH_3), 3.14 (br s, 3H, COOCH_3′), 2.01 (s, 3H, C6CH_3), and 1.93 (s, 3H, C3‴CH_3).

^{13}C{^{1}H} NMR (C_6D_6, 126 MHz): δ 172.2, 160.03, 160.01, 148.0, 142.5, 142.0, 140.1, 140.0, 137.6, 136.2, 135.1, 133.4, 132.2, 130.1, 128.7, 128.3, 125.0, 122.8, 122.5, 122.1, 120.8, 120.7, 117.3, 114.7, 114.6, 113.7, 113.5, 110.3, 107.5, 96.2, 88.4, 61.1, 55.2, 55.0, 52.9 (br, 2x), 42.4, 40.4, 23.5, and 21.8.

NMR spectral data for the minor regioisomer:

^{1}H NMR (C_6D_6, 500 MHz): δ 7.77–7.68 (br m, 2H, *H2′* and *H6′*), 7.53 (d, *J* = 8.1 Hz, 1H, *H6*)[note to self , 7.26 (nfod, J_{app} = 8.7 Hz, 2H, *H2″* and *H6″*), 7.04–6.97 (overlapped m, *H9*, *H3′* and *H5′*), 6.94–6.85 (overlapped m, *H5‴*), 6.79 (dd, 8.1, 1.6 Hz, *H7*), 6.58–6.51 (overlapped m, *H4‴*), 6.56 (nfod, J_{app} = 8.7 Hz, 2H, *H3″* and *H5″*), 6.34 (br ddd, *J* = 2.3, 2.3 Hz, 1H, *H2‴*), 6.25 (br ddd, *J* = 8.1, 2.3 Hz, 1H, *H6‴*), 5.69 (s, 1H, N*H*), 4.57 (d, *J* = 16.5 Hz, 1H, C1H_a), 4.52–4.31 (overlapped m, C3H_a and C3H_b), 3.96 (d, *J* = 16.5 Hz, 1H, C1H_b), 3.38 (s, 3H, C4′OCH_3), 3.32 (br s, 3H, COOCH_3), 3.15 (s, 3H, C4″OCH_3), 3.14 (br s, 3H, COOCH_3′), 2.13 (s, 3H, C8CH_3), and 1.91 (s, 3H, C3‴CH_3).·

^{13}C{^{1}H} NMR (C_6D_6, 126 MHz): δ 172.9, 160.2, 160.0, 148.1, 142.3, 141.8, 140.2, 139.5, 138.2, 136.7, 134.8, 133.5, 132.7, 130.2, 128.7, 128.5, 126.6, 123.1, 122.5, 121.4, 120.9, 120.6, 117.2, 114.8, 114.6, 113.8, 113.4, 110.2, 109.9, 95.9, 87.8, 61.1, 55.1, 55.0, 52.8 (br, 2x), 42.3, 39.9, 22.2, and 21.8.

Data from the sample of both isomers:

HRMS (ESI-TOF) m/z: $[M+H^+–H_2]^+$ Calcd for $C_{43}H_{37}N_2O_6$ 677.2646; Found 677.2661.

IR (neat): 3226, 3037, 3002, 2953, 2838, 1732, and 1172 cm^{-1}.

Dimethyl 7-methoxy-5-(4-(methoxycarbonyl)phenyl)-4-((4-(methoxycarbonyl)phenyl)ethynyl)-10-((4-methoxyphenyl)amino)-3,10-dihydrocyclopenta[a]carbazole-2,2(1H)-dicarboxylate (11f)

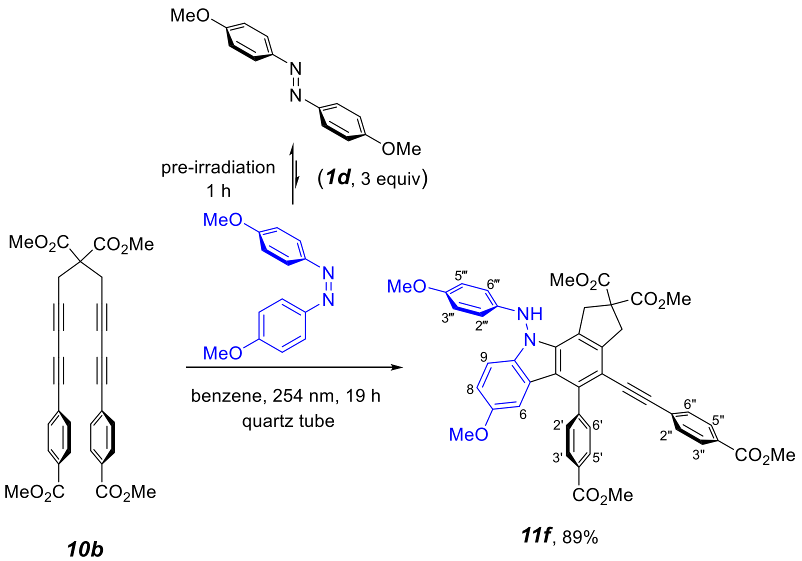

Following general procedure B, dimethyl 2,2-bis(5-(4-(methoxycarbonyl)phenyl)penta-2,4-diyn-1-yl)malonate (**10b**, 20 mg, 0.039 mmol, 1 equiv), (*E*)-1,2-*bis*(4-methoxyphenyl)diazene (**1d**, 28 mg, 0.12 mmol, 3.1 equiv), and benzene (1 mL) were used to prepare the carbazole **11f**. Purification by MPLC (1:1 hexanes:EtOAc) yielded **11f** (27 mg, 0.035 mmol, 89%) as a pale yellow crystalline solid.

Data for the carbazole derivative 11f:

^{1}H NMR ($CDCl_3$, 500 MHz): δ 8.27 (nfod, J_{app} = 8.5 Hz, 2H, *H3′* and *H5′*), 7.91 (nfod, J_{app} = 8.5 Hz, 2H, *H3″* and *H5″*), 7.70 (br d, *J* = 7.9 Hz, 2H, *H2′* and *H6′*), 7.36 (s, 1H, N*H*), 7.22 (nfod, J_{app} = 8.5 Hz, 2H, *H2″* and *H6″*), 7.12 (d, *J* = 8.8 Hz, 1H, *H9*), 6.90 (dd, *J* = 8.8, 2.5 Hz, 1H, *H8*), 6.78 (nfod, *J* = 9.0 Hz, 2H, *H2‴* and *H6‴*), 6.56 (nfod, J_{app} = 9.0 Hz, 2H, *H3‴* and *H5‴*), 6.46 (d, *J* = 2.5 Hz, 1H, *H6*), 4.01 (s, 3H, COOCH_3), 3.95–3.81 (overlapped m, 8H, COOCH_3′, COOCH_3″, C1H_a, C3H_a), 3.77–3.72 (overlapped m, 8H, COOCH_3‴, C4‴OCH_3, C1H_b, C3H_b), and 3.51 (s, 3H, C8OCH_3).

^{13}C{^{1}H} NMR ($CDCl_3$, 126 MHz): δ 172.2, 172.1, 167.2, 166.8, 154.8, 154.5, 144.1, 142.1, 140.9, 138.3, 137.8, 135.2, 131.1, 130.5, 129.8, 129.7, 129.6, 129.1, 128.5, 122.0, 121.0, 118.4, 115.12, 115.06, 114.2, 110.4, 110.0, 105.5, 94.3, 90.7, 60.2, 55.74, 55.66, 53.4, 53.3, 52.4, 52.3, 41.2, and 39.1.

HRMS (ESI-TOF) *m/z*: $[M+H^+–H_2]^+$ Calcd for $C_{45}H_{37}N_2O_{10}$ 765.2443; Found 765.2417.

IR (neat): 3325, 2998, 2951, 2904, 2834, 1718, and 1172 cm^{-1}.

mp: 120–122 °C.

(*Z*)-1,2-Diphenyldiazene (*cis*-1a)

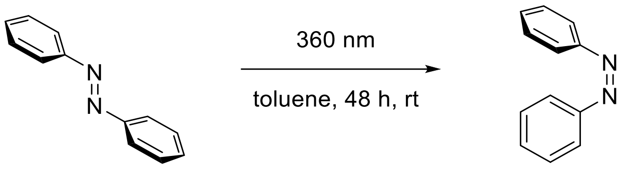

trans-1a ***cis-1a***, 5.6% (quant. b.r.s.m.)

Commercial azobenzene (***trans*-1a**, 5.01 g, 27.5 mmol, 1 equiv) was dissolved in toluene (20 mL) in a 250 mL round-bottom flask. The flask was placed in a Rayonet reactor fitted with 360 nm black lamps and irradiated for 48 h. During the following manipulations, care was taken to minimize exposure to ambient laboratory light. The reaction mixture was loaded directly to a silica flash column and chromatographed (9:1 hexanes:EtOAc) to yield two red oils, which, once concentrated, solidified when stored in a refrigerator to form orange crystalline solids. The first compound to elute was the recovered starting material, (*E*)-1,2-diphenyldiazene (***trans*-1a**, 4.73 g, 26.0 mmol), and the second was (*Z*)-1,2-diphenyldiazene (***cis*-1a**, 0.28 g, 1.5 mmol, 5.6%). The proton and carbon NMR spectroscopic properties for ***cis*-1a** matched those reported (surprisingly, only one time that we were able to find) in the literature.[8]

Data for (*Z*)-1,2-diphenyldiazene *cis*-1a:

^{1}H NMR ($CDCl_3$, 500 MHz): δ 7.25 (nfom, 4H, PhH_m), 7.14 (tt, J = 7.0, 1.2 Hz, 2H, PhH_p), and 6.84 (nfod, J_{app} = 8.4 Hz, 4H, PhH_o).

^{13}C{^{1}H} NMR ($CDCl_3$, 126 MHz): δ 153.6, 128.8, 127.5, and 120.7.

HRMS (ESI-TOF) *m/z*: $[M+H^+]^+$ Calcd for $C_{12}H_{11}N_2^+$ 183.0917; Found 183.0914.

IR (neat): 3057, 1477, 1445, 1265, 1069, 926, 776, 758, 735, 691, 593, and 493 cm^{-1}.

N-Phenyl-9H-carbazol-9-amine (7a)

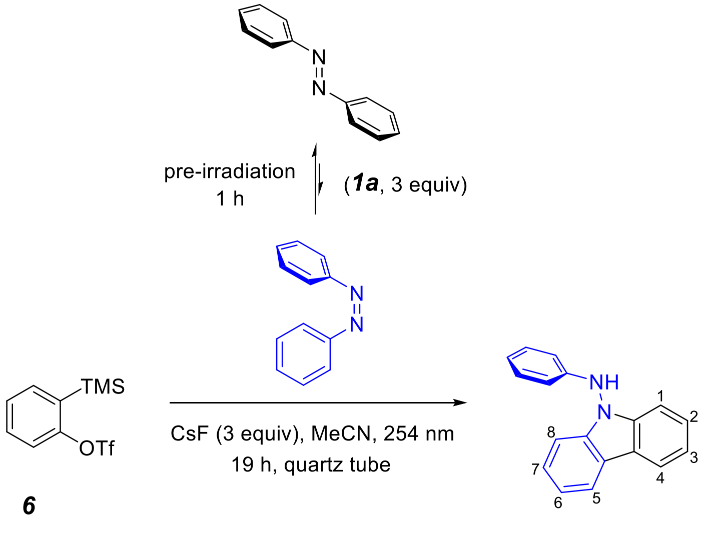

Following general procedure C, (2-trimethylsilyl)phenyl triflate (**6**, 50 mg, 0.17 mmol, 1 equiv), (*E*)-1,2-diphenyldiazene (**1a**, 92 mg, 0.51 mmol, 3 equiv), CsF (54 mg, 0.50 mmol, 3 equiv), and MeCN (2 mL) were used to prepare the carbazole **7a**. Purification by MPLC (9:1 hexanes:EtOAc) yielded **7a** (23 mg, 0.091 mmol, 54%) as a white crystalline solid.

Data for the carbazole derivative 7a:

^{1}H NMR ($CDCl_3$, 500 MHz): δ 8.11 (ddd, *J* = 7.8, 1.0, 1.0 Hz, 2H, *H4* and *H5*), 7.41 (dd, *J* = 8.1, 6.7, 1.1 Hz, 2H, *H2* and *H7*), 7.38 (ddd, *J* = 8.0, 1.5, 0.7 Hz, 2H, *H1* and *H8*), 7.28 (ddd, *J* = 7.9, 6.7, 1.5 Hz, 2H, *H3* and *H6*), 7.18 (nfom, 2H, PhH_m,), 6.89 (tt, *J* = 7.4, 1.1 Hz, 1H, PhH_p), 6.61 (nfom, 2H, PhH_o), and 6.58 (s, 1H, N*H*).

^{13}C{^{1}H} NMR ($CDCl_3$, 126 MHz): δ 146.7, 140.4, 129.6, 126.3, 121.5, 121.2, 120.5, 120.4, 112.9, and 109.2.

HRMS (ESI-TOF) *m/z*: $[M+H^+]^+$ Calcd for $C_{18}H_{15}N_2$ 259.1230; Found 259.1218.

IR (neat): 3318, 3048, and 3021 cm^{-1}.

mp: 163–164 °C (lit. mp 170–171 °C).[9]

1 mmol scale reaction

To demonstrate the scalability of the "Kobayashi" method, general procedure C was followed with slight modifications: (*E*)-1,2-diphenyldiazene (**1a**, 549 mg, 3.01 mmol, 3 equiv) was dissolved in ca. 10 mL MeCN in a quartz glass vessel and pre-irradiated with 254 nm light for 12 h. ^{1}H NMR analysis of an aliquot suggest an ca. 1:2 mixture of the *Z*- to *E*-azobenzenes. (2-Trimethylsilyl)phenyl triflate (**6**, 300 mg, 1.01 mmol, 1 equiv) and CsF (326 mg, 3.01 mmol, 3

equiv) and a magnetic stir bar were added. The resulting suspension was then stirred under 254 nm irradiation for 32 h. The reaction mixture was concentrated in vacuo and partitioned between DCM and water. The organic layer was washed with brine, dried over Na_2SO_4, filtered, and concentrated. The residue was purified by flash column chromatography on silica gel (19:1 hexanes:EtOAc). This afforded *N*-phenyl-9H-carbazol-9-amine (**7a**, 159 mg, 0.617 mmol, 61.4%) as an off-white crystalline solid that matched the spectral and physical properties described for the product of the small-scale reaction. This sample was recrystallized and its melting point compared to that in the literature. The proton NMR spectrum given in the ESI is from the recrystallized sample.

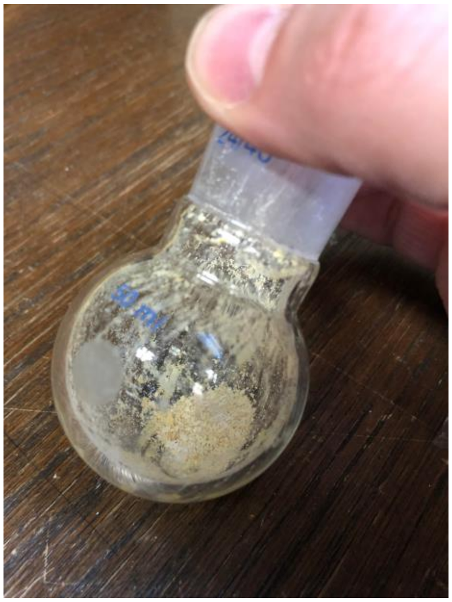

Figure S3. The solid sample of **7a** obtained from reaction scale-up. (This photograph was taken by Dorian Sneddon, one of the authors of this manuscript.)

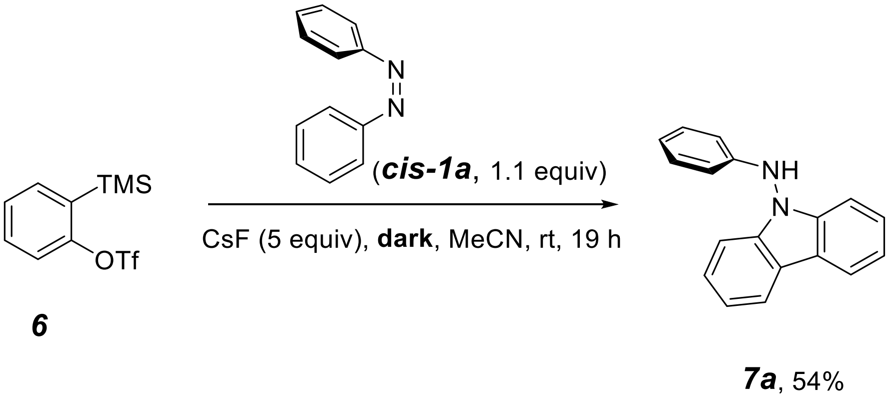

An alternative procedure was carried out in which pure *cis*-azobenzene (***cis*-1a**) was allowed to react with the Kobayashi benzyne precursor in a stoichiometric ratio of 1.1:1.0. (2-Trimethylsilyl)phenyl triflate (**6**, 50 mg, 0.17 mmol, 1 equiv) was dissolved in 2 mL of MeCN in a screw-capped culture tube. (*Z*)-1,2-Diphenyldiazene (***cis*-1a**, 34 mg, 0.19 mmol, 1.1 equiv) and

CsF (91 mg, 0.84 mmol, 5 equiv) were added, and the culture tube was covered with aluminum foil. This mixture was allowed to stir magnetically overnight. The acetonitrile was evaporated under reduced pressure and the residue was partitioned between DCM and water. The organic layer was washed with brine, dried over Na_2SO_4, filtered, and concentrated. The residue was purified using MPLC (9:1 Hex:EtOAc), which yielded *N*-phenyl-9H-carbazol-9-amine (**7a**, 24 mg, 0.094 mmol, 56%) as a white crystalline solid that matched the spectral and physical properties described above.

3-Methyl-*N*-(*p*-tolyl)-9H-carbazol-9-amine (7c)

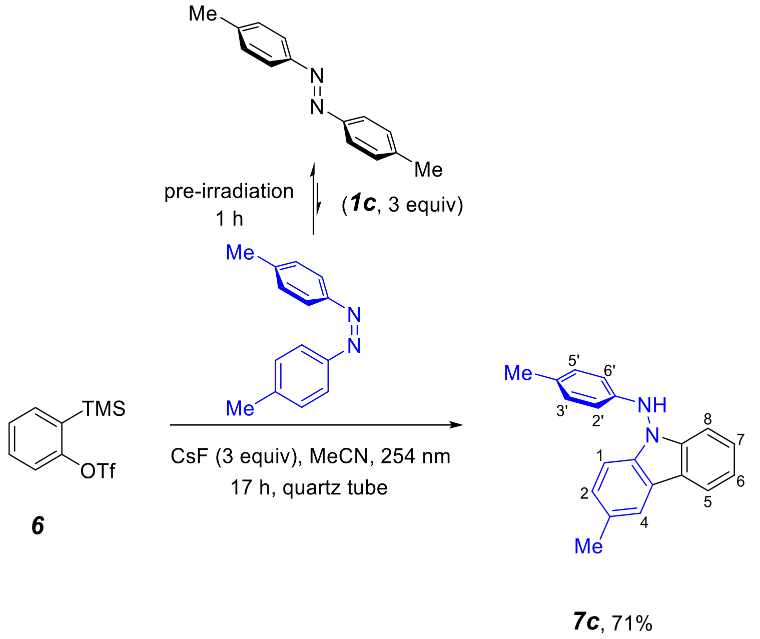

Following general procedure C, (2-trimethylsilyl)phenyl triflate (**6**, 50 mg, 0.168 mmol, 1 equiv), (*E*)-1,2-di-*p*-tolyldiazene (**1c**, 106 mg, 0.504 mmol, 3 equiv), CsF (54 mg, 0.50 mmol, 3 equiv), and MeCN (2 mL) were used to prepare the carbazole **7c**. Purification by MPLC (9:1 hexanes:EtOAc) yielded **7c** (34 mg, 0.12 mmol, 71%) as a pale yellow crystalline solid.

Data for the carbazole derivative 7c:

^{1}H NMR ($CDCl_3$, 500 MHz): δ 8.06 (ddd, *J* = 7.7, 0.9, 0.9 Hz, 1H, *H5*), 7.89 (ddq, *J* = 1.6, 0.8, 0.8 Hz, 1H, *H4*), 7.39 (d, *J* = 8.1, 6.4, 1.2 Hz, 1H, *H7*), 7.36 (dd, *J* = 8.1, 1.9, 0.8 Hz, 1H, *H8*), 7.27 (dd, *J* = ~8, 0.8 Hz, 1H, *H1*), 7.24 (ddd, *J* = 8.0, 6.4, 1.9 Hz, 1H, *H6*), 7.22 (ddq, *J* = 8.2, 1.4, 0.7 Hz, 1H, *H2*), 6.98 (nfod, J_{app} = 8.0 Hz, 2H, *H3′* and *H5′*), 6.51 (nfod, J_{app} = 8.5 Hz, 2H, *H2′* and *H6′*), 6.50 (s, 1H, N*H*), 2.53 (dd, *J* = 0.8, 0.8 Hz, 3H, C3CH_3), and 2.24 (t, *J* = 0.8 Hz, 3H, C4’CH_3).

^{13}C{^{1}H} NMR ($CDCl_3$, 126 MHz): δ 144.5, 140.7, 138.7, 130.5, 130.1, 129.6, 127.6, 126.1, 121.6, 121.3, 120.5, 120.4, 120.0, 113.0, 109.2, 108.9, 21.6, and 20.7.

HRMS (ESI-TOF) *m/z*: $[M+H^+]^+$ Calcd for $C_{20}H_{19}N_2$ 287.1543; Found 287.1530.

IR (neat): 3320, 3047, 3022, 2918, and 2862 cm^{-1}.

mp: 173–175 °C (lit. mp 167–168 °C).[9]

3-Methoxy-*N*-(4-methoxyphenyl)-9H-carbazol-9-amine (7d)

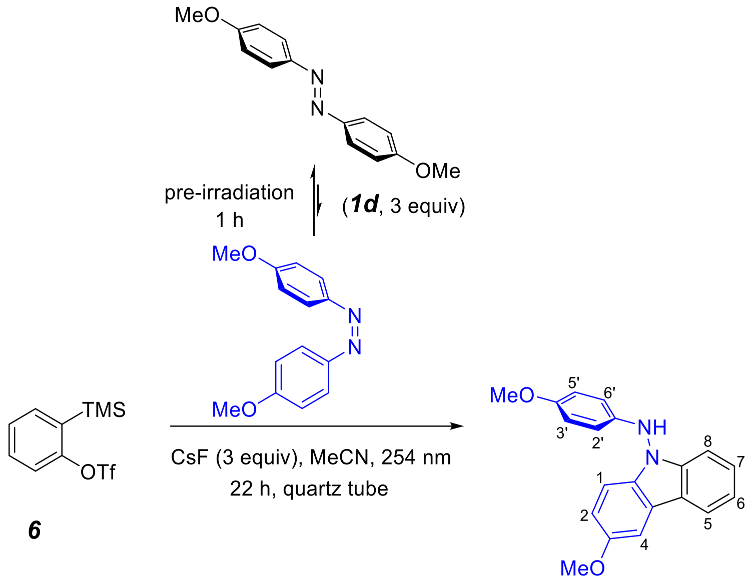

7d, 65%

Following general procedure C, (2-trimethylsilyl)phenyl triflate (**6**, 50 mg, 0.168 mmol, 1 equiv), (*E*)-1,2-*bis*(4-methoxyphenyl)diazene (**1d**, 122 mg, 0.504 mmol, 3 equiv), CsF (54 mg, 0.50 mmol, 3 equiv), and MeCN (2 mL) were used to prepare the carbazole **7d**. Purification by MPLC (9:1 hexanes:EtOAc) yielded **7d** (35 mg, 0.11 mmol, 65%) as a pale yellow crystalline solid.

Data for the carbazole derivative 7d:

^{1}H NMR ($CDCl_3$, 500 MHz): δ 8.04 (ddd, *J* = 7.8, 1.0, 1.0 Hz, 1H, *H5*), 7.56 (d, *J* = 2.4 Hz, 1H, *H4*), 7.39 (ddd, *J* = 8.2, 6.9, 1.2 Hz, 1H, *H7*), 7.35 (ddd, *J* = 8.2, 1.1, 1.1 Hz, 1H, *H8*), 7.25 (d, *J* = 8.7 Hz, 1H, *H1*), 7.23 (ddd, *J* = 7.9, 6.9, 1.2 Hz, 1H, *H6*), 7.03 (dd, *J* = 8.7, 2.4 Hz, 1H, *H2*), 6.73 (nfod, J_{app} = 9.0 Hz, 2H, *H2′* and *H6′*), 6.53 (nfod, J_{app} = 9.0 Hz, 2H, *H3′* and *H5′*), 6.37 (s, 1H, N*H*), 3.92 (s, 3H, OCH_3), and 3.71 (s, 3H, OCH_3′).

^{13}C{^{1}H} NMR ($CDCl_3$, 126 MHz): δ 154.6, 154.5, 141.1, 140.6, 135.3, 126.2, 121.9, 121.2, 120.4, 119.8, 115.2, 115.0, 114.2, 110.0, 109.3, 103.7, 56.2, and 55.8.

HRMS (ESI-TOF) *m/z*: $[M+H^+–H_2]^+$ Calcd for $C_{20}H_{17}N_2O_2$ 317.1285; Found 317.1270.

IR (neat): 3311, 3053, 2996, 2935, 2905, and 2832 cm^{-1}.

mp: 159–160 °C (lit. mp 159–161 °C).[9]

Syntheses of 12, 13, and 14 and mass spec generation of the phenazinium ion 15

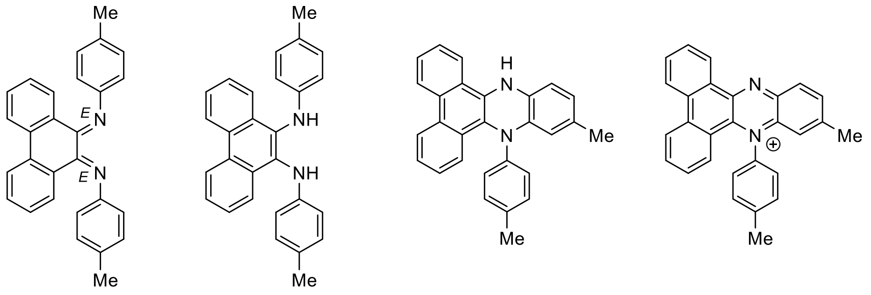

12 ***13*** ***14*** ***15***

<u>Mechanistic Experiments Modeling the Key Electrocyclization Process using the Phenanthrene Diiminoquinone Model System **12** (which disproportionates to **13** and **14**)</u>

To gain further support for the key electrocyclization event that is proposed to account for the dihydrophenazine core, we attempted to synthesize the phenanthrene diiminoquinone **12** following a reported $TiCl_4$-promoted method[10] for the synthesis of the unsubstituted analogue (which used aniline rather than *p*-toluidine). The analog with a *p*-tolyl functionality was chosen here to simplify identification of the eventual dihydrophenazine by way of NMR spectral analysis. To our surprise, we observed, instead, formation of the reduced diamine **13** in modest yield as the major product of this $TiCl_4$ promoted condensation instead of the expected diimine.[10] The proton and carbon NMR spectra of the diamine **13** in $CDCl_3$ matched those reported in the literature, prepared there by a different method.[11] (We have located one report that hints at the formation of an analogous diamine as the major product formed by the $TiCl_4$ protocol.[12]) We then observed that subsequent treatment of **13** with 10 equivalents of MnO_2 in DCM for ca. 10 minutes quickly oxidized diamine **13** to the desired diimine **12**. The only reported proton NMR spectral data available for a compound having the constitution of **12** are of an isomer that most likely had the *Z*,*Z*-configuration (the corresponding diphenyl analog, prepared in that study by the same method as the ditolyl, was shown to be the *Z,Z*-isomer by X-ray diffraction analysis.[13]) We followed a literature procedure that described reaction of 9,10-phenanthrenequinone with aniline.[10a] We used *p*-toluidine as the amine and, instead of recrystallizing the product, used flash chromatography as the purification method.

Preparation of *N*^{9},*N*^{10}-di-*p*-tolylphenanthrene-9,10-diamine (13) [from condensation of 9,10-phenanthrenequinone and *p*-toluidine and accompanying redox events]

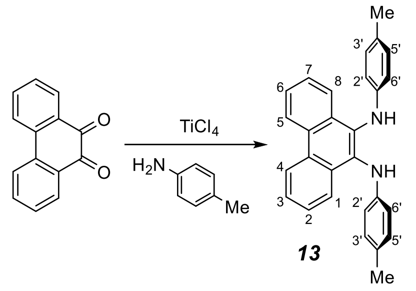

9,10-Phenanthrenequinone (0.500 g, 2.41 mmol, 1 equiv) and *p*-toluidine (1.54 g, 14.4 mmol, 6 equiv) were added to a flame-dried 50 mL round-bottom flask and suspended in 15 mL of dry toluene. The flask was placed under dry nitrogen and stirred while $TiCl_4$ (0.25 mL, 2.3 mmol, 1 equiv) was added dropwise at ambient temperature. The reaction mixture quickly became black and viscous. Reaction progress was monitored by TLC and, after 16 hours, the reaction was quenched by addition of an equal volume of water. No evidence of the diimine **12** was detected. An additional 50 mL of toluene was added, and the organic layer was washed with 50 mL of additional water. The organic layer was dried over Na_2SO_4, filtered, and concentrated to give a tarry black crude material. This was taken up in 15 mL of toluene and

loaded onto a longer than typical silica flash column (l = ca. 15") because **13** elutes very closely with the excess *p*-toluidine (R_f = 0.3 vs. 0.25 in 9:1 Hex:EtOAc, respectively.) The column was eluted with 9:1 Hex:EtOAc to give the sample of **13** (0.184 g, 0.476 mmol, 19.8%) as brown crystals, whose proton and carbon NMR spectra matched those reported in the literature.[11]

^{1}H NMR ($CDCl_3$, 500 MHz): δ 8.73 (dd, *J* = 8.4, 1.2 Hz, 2H, *H4* and *H5*), 8.00 (dd, *J* = 8.3, 1.4 Hz, 2H, *H1* and *H8*), 7.62 (ddd, *J* = 8.4, 6.9, 1.4 Hz, 2H, *H3* and *H6*), 7.51 (ddd, *J* = 8.2, 6.9, 1.2 Hz, 2H, *H2* and *H7*), 7.94 (nfod, J_{app} = 8.6 Hz, 4H, *H3′* and *H5′*), 6.55 (nfod, J_{app} = 8.4 Hz, 4H, *H2′* and *H6′*), 5.78 (s, 2H, N*H*), and 2.24 (s, 6H, CH_3).

^{13}C{^{1}H} NMR ($CDCl_3$, 126 MHz): δ 144.4, 131.3, 130.2, 130.02, 129.97, 129.0, 127.0, 126.3, 125.1, 123.0, 115.5, and 20.6.

HRMS (ESI-TOF) *m/z*: $[M+H^+]^+$ Calcd for $C_{28}H_{22}N_2$ 387.1856; Found 387.1851.

IR (neat): 3370, 3021, 2920, 2860, 1612, 1511, 1334, 1246, 812, 751, and 726 cm^{-1}.

mp: 184–186 °C.

(9*E*,10*E*)-*N*^{9},*N*^{10}-Di-*p*-tolylphenanthrene-9,10-diimine (12) by oxidation of 13

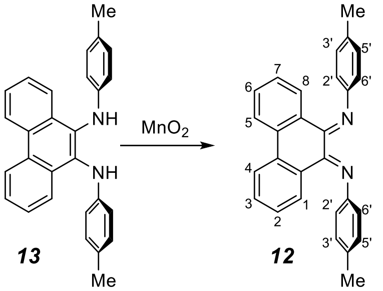

13 *12*

N^9,*N*^{10}-Di-*p*-tolylphenanthrene-9,10-diamine (**13**, 150 mg, 0.388 mmol, 1 equiv) was added to a 50 mL round-bottom flask and dissolved in 20 mL of DCM. A magnetic stir bar and MnO_2 (337 mg, 3.88 mmol, 10 equiv) were added. The suspension was stirred at room temperature for ten minutes, at which point full consumption of **13** was observed by TLC. The MnO_2 was removed by filtration and the solvent was evaporated from the filtrate. The crude material was then purified by MPLC (9:1 Hex:EtOAc) to afford **12** (73 mg, 0.476 mmol, 49%) as a red crystalline solid. [It is of note that care must be taken when isolating this material, as it will decompose (see the following experimental procedure) over time in solution, and, especially when heated: *e.g.,* in a heating bath during rotary evaporation. This phenomenon was observed by TLC and NMR spectroscopy.]

^{1}H NMR ($CDCl_3$, 500 MHz): δ 8.08 (dd, *J* =7.7, 1.5 Hz, 2H, *H4* and *H5* or *H1* and *H8*), 7.92 (dd, *J* =7.9, 1.2 Hz, 2H, *H1* and *H8* or *H4* and *H5*), 7.59 (ddd, *J* = 7.8, 7.4, 1.4 Hz, 2H, *H2* and *H7* or *H3* and *H6*), 7.47 (ddd, *J* = 7.5, 7.5, 1.2 Hz, 2H, *H3* and *H6* or *H2* and *H7*), 6.82 (nfodd, J_{app} = 8.5, 0.8 Hz, 4H, *H3′* and *H5′*), 6.20 (nfod, J_{app} = 8.3 Hz, 4H, *H2′* and *H6′*), and 2.24 (t, *J* = 0.8 Hz, 6H, CH_3).

^{13}C{^{1}H} NMR ($CDCl_3$, 126 MHz): δ 159.8, 147.0, 135.7, 134.7, 134.3, 131.6, 129.3, 129.0, 126.5, 123.7, 119.8, and 21.1.

HRMS (ESI-TOF) *m/z*: $[M+H^+]^+$ Calcd for $C_{28}H_{24}N_2$ 389.2012; Found 389.2009.

IR (neat): 3021, 2919, 2862, 1595, 1502, 1449, 813, 753, 724, and 546 cm^{-1}.

mp: 120–125 °C (with decomposition).

Thermal disproportionation of 11 to 12 and (the metastable) 11-methyl-9-(*p*-tolyl)-9,14-dihydrodibenzo[*a*,*c*]phenazine (14)

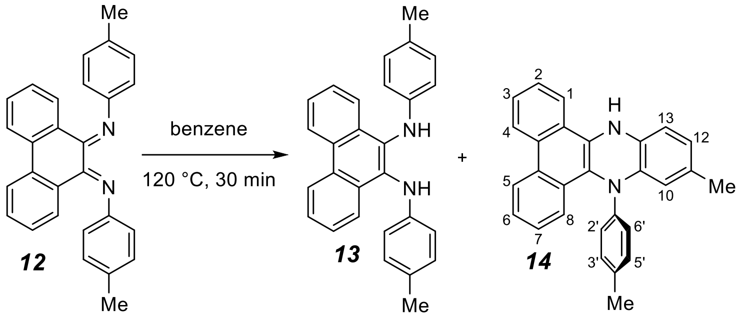

A sample of **12** (ca. 10 mg) was dissolved in C_6D_6 (1 mL) and heated to 90 °C for 1 h in a screw-capped culture tube. The crude proton NMR spectrum was recorded (Figure S6), and the presence of a mixture of diimine **12**, diamine **13**, and dihydrophenazine **14** was observed. The dihydrophenazine **14** was isolated by MPLC (9:1 Hex:EtOAc), and its proton NMR spectrum was also recorded in deuterobenzene. Severe line-broadening was observed in the proton spectrum, likely as a result of the interconversion of diastereomers resulting from N-H pyramidalization.

This transformation was repeated and allowed to reach full consumption of the starting material:

(9*E*,10*E*)-N^9,N^{10}-Di-*p*-tolylphenanthrene-9,10-diimine (**12**, 50 mg, 0.13 mmol, 1 equiv) was added to a screw-capped culture tube and dissolved in 2 mL of benzene. The solution was warmed in a block heater to 120 °C. After 30 minutes, full consumption of the starting material was apparent by TLC. The crude mixture was loaded directly onto a silica MPLC column (eluant of 9:1 Hex:EtOAc) to afford, in order of elution, **13** (13 mg, 0.034 mmol, 26%) as a brown crystalline solid with spectral and physical properties matching those described above and **14** (29 mg, 0.476 mmol, 58%). Upon initial concentration, samples of **14** appear as a cream-colored crystalline solid, but degradation during handling of the material (as observed by TLC and NMR spectroscopy) is apparent. Solutions of **14** at ambient temperature darken over time and their concentration leads to a viscous, amber-colored and, eventually, molasses-brown-colored substance. The spectral properties and instability of this material precluded our ability to obtain a meaningful carbon NMR spectrum or melting point. Notably, we find no examples in the literature describing NMR characterization of any mono-*N*-arylated 9,10-dihydrophenazines.

^{1}H NMR ($CDCl_3$, 500 MHz): δ 8.75 (br s, 1H, *H4* or *H5*), 8.61 (br s, 1H, *H4* or *H5*), 8.24–8.03 (br m, 1H, *H1* or *H8*), 7.99–7.79 (br m, 1H, *H1* or *H8*), 7.78–7.26 (br m, 6H), 6.99–6.76 (br m, 5H), 6.55 (br s, 1H, N*H*), 2.61–2.26 (br m, 3H, C*H*$_3$), and 2.25–2.09 (br m, 3H, C*H*$_3$′).

^{13}C NMR: precluded by rapid degradation (see comment at end of the above paragraph)

HRMS (ESI-TOF) *m/z*: $[M+H^+]^+$ Calcd for $C_{28}H_{22}N_2$ 387.1856; Found 387.1856.

IR (neat): 3422, 3065, 3026, 2919, 2858, 1605, 1500, 1448, 1421, 1291, 907, 787, and 722 cm^{-1}

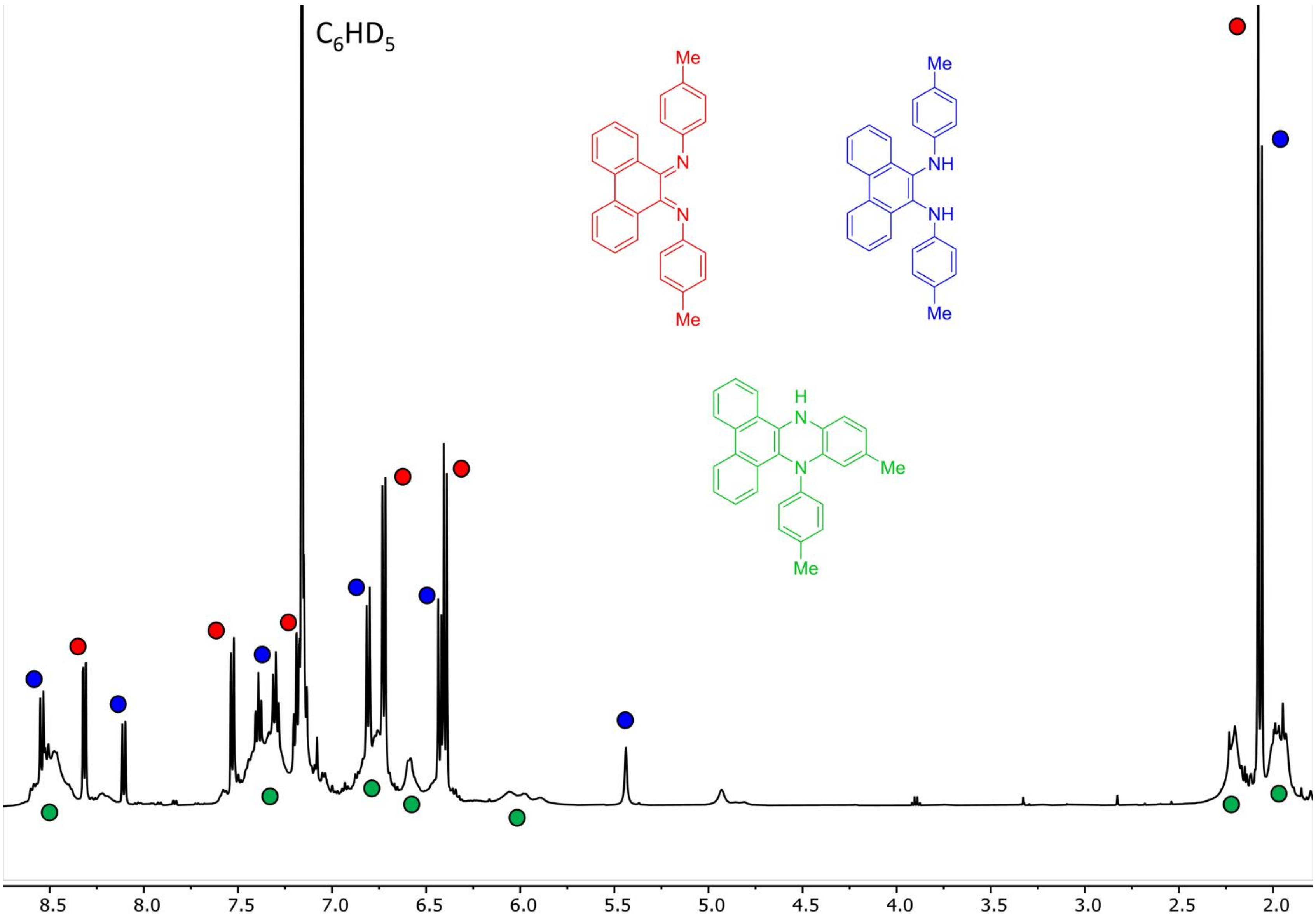

Figure S4. Proton NMR spectrum (in deuterobenzene) of the *in situ* reaction mixture described on the previous page.

i) APCI MS evidence for the generation of phenazinium ion 15 during analysis of 12 and ii) LC-MS (ESI) evidence for the presence of 15 in the product mixture arising from 12:

11-Methyl-9-(*p*-tolyl)dibenzo[*a*,*c*]phenazin-9-ium (15)

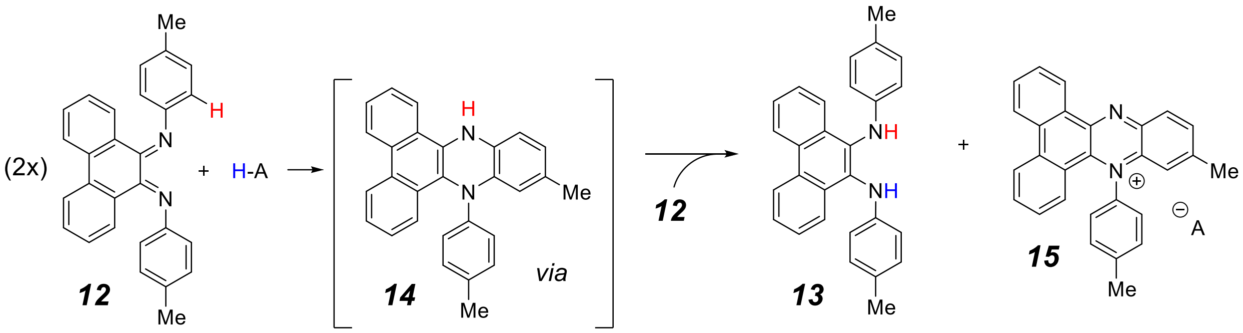

i) The proton NMR spectra of diamine **13**, diimine **12**, and dihydrophenazine **14** recorded in deuterobenzene are provided in Section VIII of the electronic supporting information (ESI). The MS data of the diimine **12**, shown as Figure 6d in the manuscript, was obtained in the positive APCI mode on an Advion CMS instrument with direct insertion of a solid sample into the ion source by way of the glass ASAP probe.

ii) Additionally, a sample of the crude mixture arising following heating of **12** for one hour, as described above (NMR spectrum shown in Figure S4), was subjected to LCMS analysis on a Waters Acuity UPLC system. An ca. 0.5 mg/mL sample of the crude material in MeCN was analyzed using a 20% to 100% MeCN/H_2O gradient method over 4 minutes with 1 minute final hold time on a UPLC column (2.1 x 50 mm) packed with C8 reverse-phase media. The chromatographic and MS traces (Waters Acuity QDa detector) are provided below.

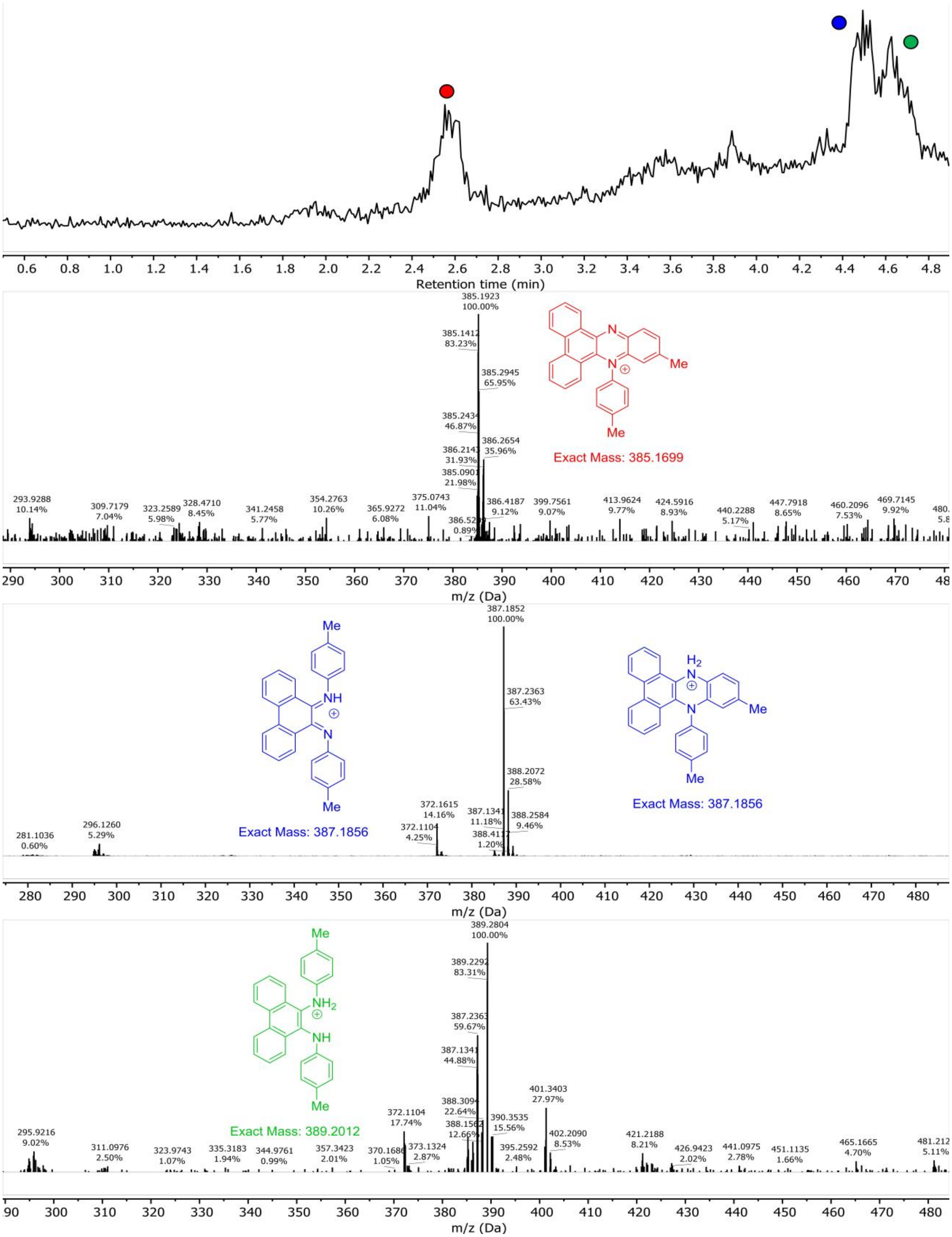

Figure S5. LCMS data corresponding to a sample of the crude mixture formed following heating of **12**. The phenazinium ion **15** is identified at a retention time of 2.6 min.

V. Variable Temperature NMR Analysis of the Carbazole 11d

Variable Temperature Experiment to Study the Line Broadening in the Proton NMR Spectra of the Carbazole Derivative 11d

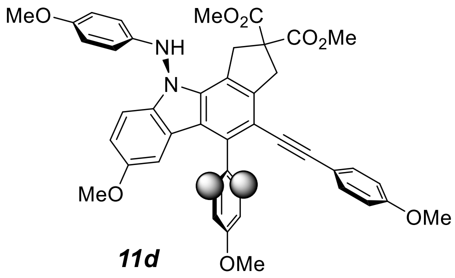

11d

An NMR sample of dimethyl 7-methoxy-5-(4-methoxyphenyl)-10-((4-methoxyphenyl)amino)-4-((4-methoxyphenyl)ethynyl)-3,10-dihydrocyclopenta[a]carbazole-2,2(1H)-dicarboxylate (**11d**) (ca. 5 mg in DMSO-d_6) was prepared. The proton NMR spectrum was collected sequentially at 298, 328, 358, and 378 K, and the aromatic proton resonances for each are shown in Figure S6.

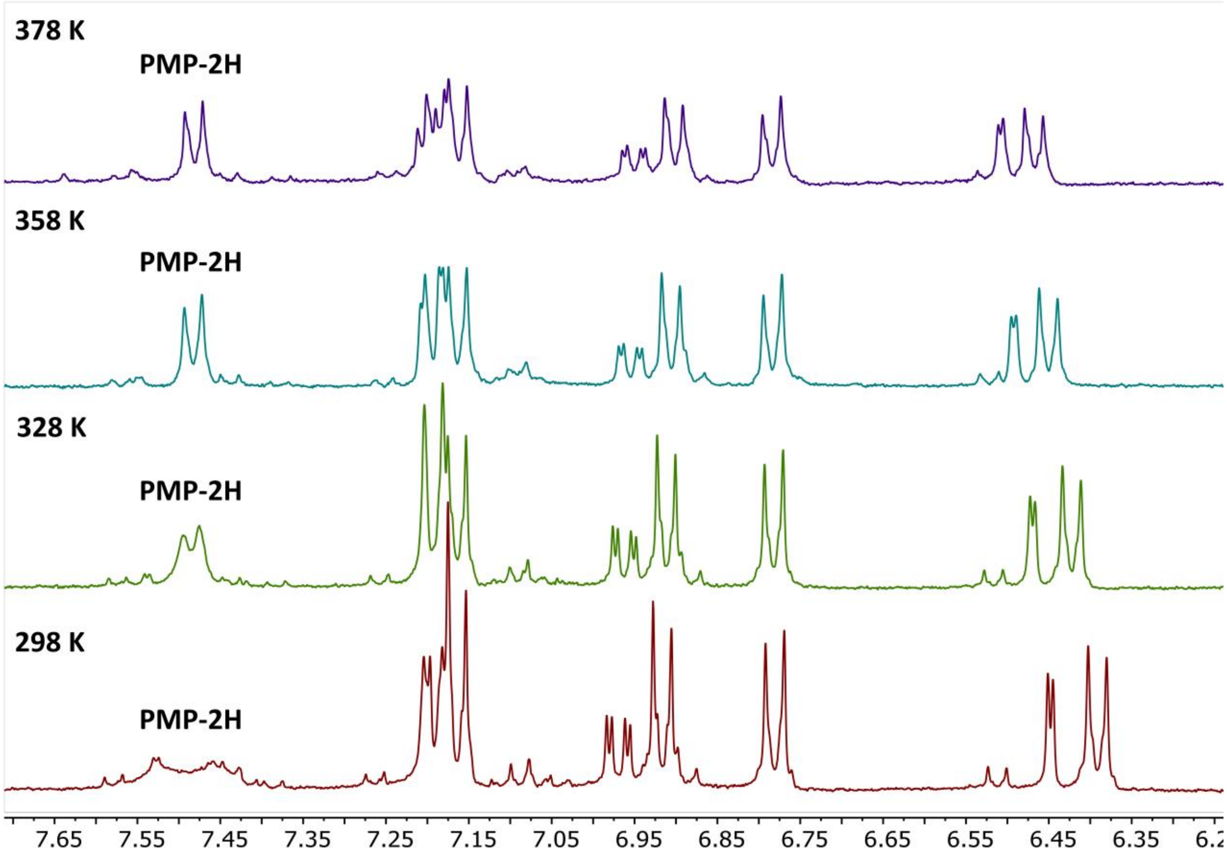

Figure S6. Variable temperature proton NMR spectra in DMSO-d_6 of the carbazole **11d**, most notably affecting the resonances for the PMP protons highlighted in the structure above (7.4–7.6 ppm).

VI. Discussion of Computational Results

All DFT calculations were performed in Gaussian 16.[14] All structures were optimized in and characterized by a frequency calculation (performed at 298 K) at the MN15/6-311++G(d,p) level of theory with solvation treatment (SMD) using benzene.

Each of the species in Figure 3 in the manuscript was subjected to an initial Monte Carlo conformational search in MacroModel (using Maestro) with the OPLS_2005 molecular mechanics (MM) force field within the Schrodinger software suite (Release 2020-4).[15] Each of these MM conformers was then optimized in Gaussian using DFT. The resulting ensemble of conformers for each intermediate on the reaction coordinate was checked for convergence and the presence of imaginary frequencies. The energies of unique conformers were then Boltzmann averaged to arrive at the reported Gibbs energy values. (A protocol outlining this process and the Python scripts used to streamline the dataflow are available in the literature.[16])

Given on the following pages are the Gibbs energy values (in Hartree atomic units) and the Cartesian coordinates for each of the computed structures. 3D representations (generated in CYLview20) for each of the structures are also shown.

Examination of the Possibility for Geometric Isomerization of *cis*-9b to *trans*-9b.

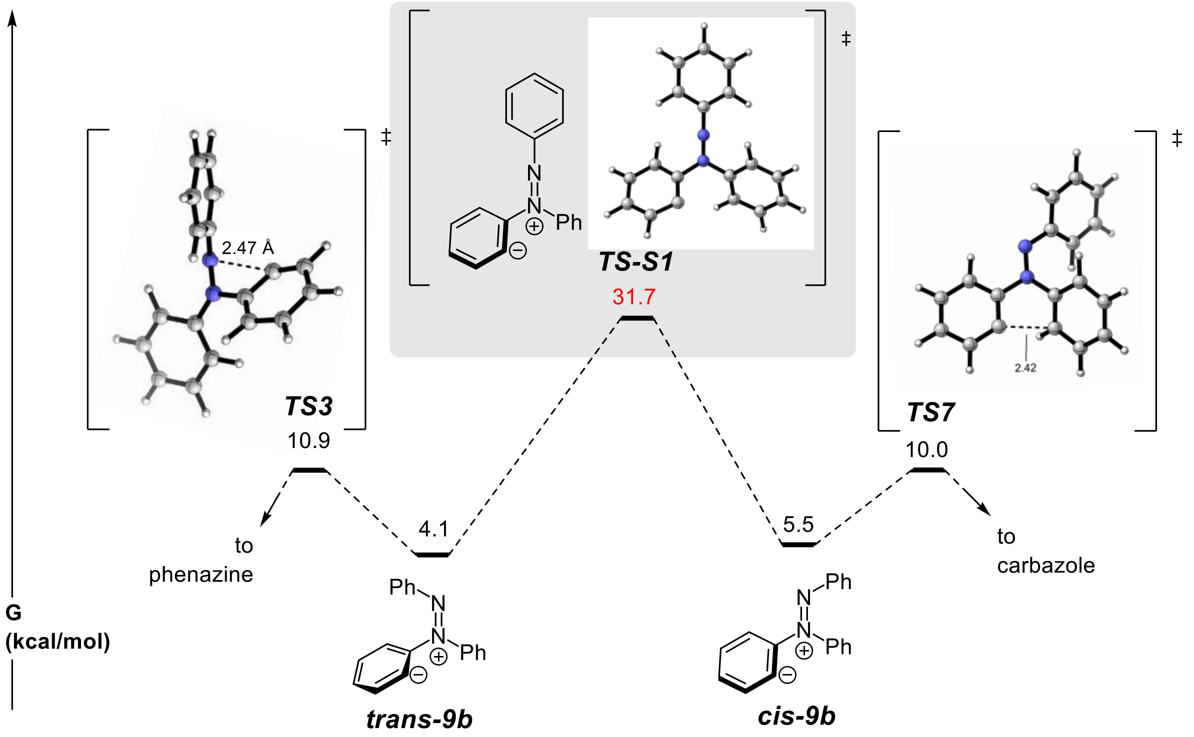

Figure S7. To check the possibility of geometric isomerization of the *cis*- and *trans*-zwitterions **9b**, we searched for a TS for this interconversion. The linearization of the azo moiety (which is the same as *N*-inversion) shows a very high barrier for the isomerization of ***cis*-9b** to ***trans*-9b** (26.2 kcal mol^{-1}) via **TS-S1**, and a TS for out-of-plane rotation about the N=N bond could not be located. It is much more likely that each of ***trans*-9b** or ***cis*-9b**, once born, proceeds on to phenazine or carbazole, respectively, without geometric interconversion. SMD(Benzene)/MN15/6-311++G(d,p)

Free Energy and Geometry for Transition State TS-S1

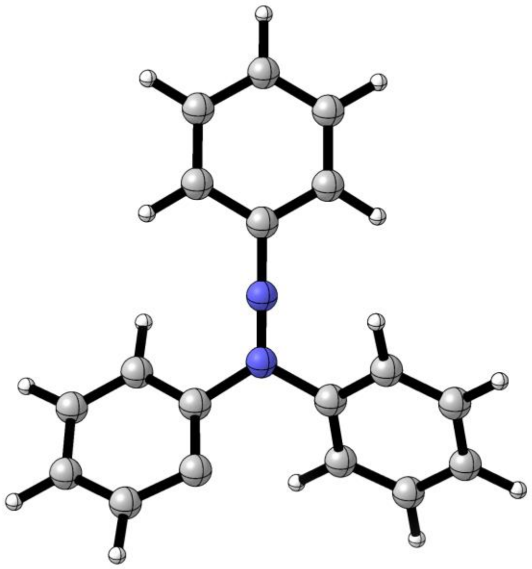

Sum of electronic and thermal free energies: -802.630711 a.u.

Number of imaginary frequencies: 1

Center Number	Atomic Number	Atomic Type	Coordinates (Ångstroms) X	Y	Z
1	6	0	-0.58971	-0.49146	-0.09032
2	6	0	1.44384	-1.49650	0.00922
3	6	0	2.19406	-2.28051	0.88739
4	6	0	1.67766	-2.84102	1.65818
5	6	0	3.57851	-2.30332	0.77255
6	6	0	4.20294	-1.58551	-0.24579
7	7	0	2.06308	-0.78909	-1.02547
8	7	0	3.44421	-0.84191	-1.15062
9	6	0	3.93259	-0.30411	-1.95477
10	6	0	1.45861	-0.22754	-1.72778
11	6	0	4.16906	-2.88866	1.46735
12	6	0	0.23966	1.42339	0.10552
13	6	0	0.85622	1.65246	1.20643
14	6	0	-0.05265	2.13700	-1.02962
15	6	0	1.36553	2.96595	1.29970
16	6	0	0.46363	3.43495	-0.92682
17	6	0	-0.60509	1.77442	-1.88849
18	6	0	1.16144	3.83672	0.22381
19	6	0	1.91051	3.31679	2.17230
20	6	0	0.31991	4.13220	-1.74526
21	1	0	1.55113	4.84918	0.27268
22	1	0	5.28196	-1.61621	-0.34505
23	1	0	-2.01109	-0.60853	-0.02496

24	1	0	-2.63130	-1.83002	-0.30480
25	1	0	-2.77167	0.52071	0.28202
26	1	0	-4.01600	-1.91890	-0.26309
27	1	0	-2.02044	-2.68677	-0.56192
28	1	0	-4.15782	0.41579	0.33407
29	1	0	-2.28054	1.45909	0.50519
30	1	0	-4.78188	-0.79856	0.05974
31	1	0	-4.50048	-2.86206	-0.48776
32	1	0	-4.74990	1.28676	0.58926
33	1	0	-5.86285	-0.87205	0.09131
34	1	0	0.04309	-1.52638	0.19018

Free Energy and Geometry for (*E*)-1,2-diphenyldiazene (*trans*-1a)

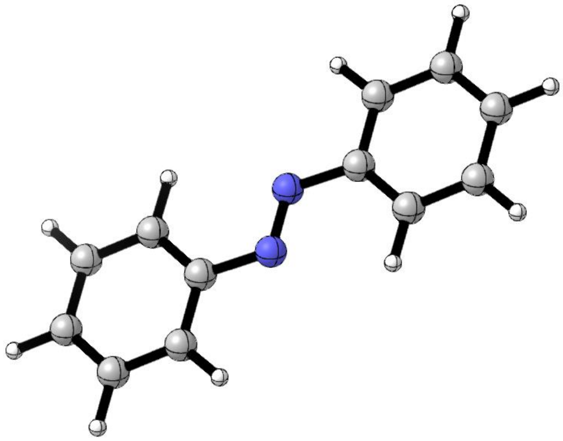

Sum of electronic and thermal free energies: -572.046643 a.u.

Number of imaginary frequencies: 0

Center Number	Atomic Number	Atomic Type	Coordinates (Ångstroms) X	Y	Z
1	6	0	-3.66266	1.30048	0.00001
2	6	0	-2.28768	1.11669	0.00001
3	6	0	1.77005	-0.18384	0.00000
4	6	0	2.62810	-1.28367	-0.00001
5	6	0	4.00643	-1.09202	-0.00001
6	6	0	-4.52430	0.20006	-0.00001
7	1	0	-4.07066	2.30498	0.00002
8	1	0	-1.60696	1.95891	0.00003
9	1	0	-2.19337	-2.27713	-0.00002
10	1	0	-4.67270	-1.94688	-0.00003
11	1	0	-5.59735	0.35480	-0.00001
12	7	0	-0.38322	-0.49140	0.00001
13	7	0	0.38322	0.49140	0.00000
14	6	0	1.77005	0.18384	0.00000
15	6	0	2.62809	1.28367	-0.00001
16	6	0	2.28768	-1.11669	0.00001
17	6	0	4.00643	1.09202	-0.00001
18	1	0	2.19337	2.27713	-0.00002
19	6	0	3.66267	-1.30048	0.00001
20	1	0	1.60697	-1.95891	0.00002
21	6	0	4.52431	-0.20006	-0.00000
22	1	0	4.67269	1.94688	-0.00002
23	1	0	4.07066	-2.30498	0.00002
24	1	0	5.59735	-0.35480	-0.00001

Free Energy and Geometry for (*Z*)-1,2-diphenyldiazene (*cis*-1a, conformer 1 of 2)

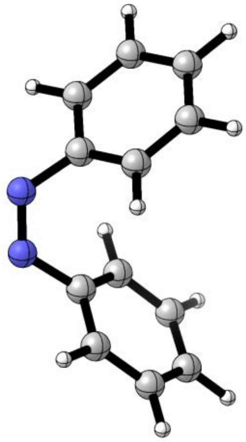

Sum of electronic and thermal free energies: -572.024902 a.u.

Number of imaginary frequencies: 0

Center Number	Atomic Number	Atomic Type	Coordinates (Ångstroms) X	Y	Z
1	7	0	-0.62115	1.99435	-0.00759
2	7	0	0.62121	1.99435	0.00765
3	6	0	-1.39797	0.79246	-0.10003
4	6	0	1.39800	0.79244	0.10004
5	6	0	1.18513	-0.15706	1.10142
6	6	0	2.04264	-1.24669	1.19924
7	6	0	3.09662	-1.39762	0.29879
8	6	0	3.31369	-0.43513	-0.68521
9	6	0	2.48178	0.67644	-0.76852
10	6	0	-1.18522	-0.15696	-1.10152
11	6	0	-2.04277	-1.24655	-1.19937
12	6	0	-3.09667	-1.39754	-0.29881
13	6	0	-3.31361	-0.43515	0.68530
14	6	0	-2.48166	0.67640	0.76862
15	1	0	0.36242	-0.03564	1.79704
16	1	0	1.88634	-1.98204	1.98020
17	1	0	3.75626	-2.25426	0.37534
18	1	0	4.14319	-0.53960	-1.37513
19	1	0	2.65222	1.45581	-1.50307
20	1	0	-0.36260	-0.03548	-1.79724
21	1	0	-1.88659	-1.98182	-1.98042
22	1	0	-3.75634	-2.25416	-0.37539
23	1	0	-4.14304	-0.53965	1.37529
24	1	0	-2.65201	1.45571	1.50327

Free Energy and Geometry for (*Z*)-1,2-diphenyldiazene (*cis*-1a, conformer 2 of 2)

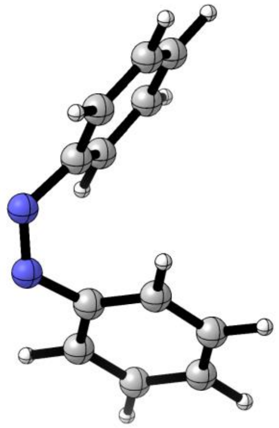

Sum of electronic and thermal free energies: -572.024894 a.u.

Number of imaginary frequencies: 0

Center Number	Atomic Number	Atomic Type	Coordinates (Ångstroms) X	Y	Z
1	7	0	-0.62119	1.99429	0.00759
2	7	0	0.62118	1.99429	-0.00758
3	6	0	-1.39793	0.79238	0.09990
4	6	0	1.39793	0.79239	-0.09989
5	6	0	1.18518	-0.15710	-1.10139
6	6	0	2.04284	-1.24664	-1.19935
7	6	0	3.09686	-1.39754	-0.29883
8	6	0	3.31388	-0.43503	0.68520
9	6	0	2.48180	0.67649	0.76861
10	6	0	-1.18515	-0.15714	1.10136
11	6	0	-2.04281	-1.24669	1.19931
12	6	0	-3.09685	-1.39756	0.29882
13	6	0	-3.31391	-0.43502	-0.68518
14	6	0	-2.48183	0.67651	-0.76858
15	1	0	0.36246	-0.03565	-1.79699
16	1	0	1.88671	-1.98190	-1.98043
17	1	0	3.75659	-2.25410	-0.37544
18	1	0	4.14342	-0.53941	1.37508
19	1	0	2.65228	1.45586	1.50315
20	1	0	-0.36241	-0.03570	1.79695
21	1	0	-1.88665	-1.98196	1.98036
22	1	0	-3.75658	-2.25412	0.37542
23	1	0	-4.14346	-0.53939	-1.37504
24	1	0	-2.65233	1.45590	-1.50309

Free Energy and Geometry for *o*-Benzyne

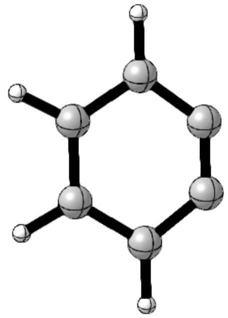

Sum of electronic and thermal free energies: -230.634555 a.u.

Number of imaginary frequencies: 0

Center Number	Atomic Number	Atomic Type	Coordinates (Ångstroms)		
			X	Y	Z
1	6	0	1.46393	-0.13290	-0.00002
2	6	0	0.62162	-1.22928	0.00002
3	6	0	-0.62171	-1.22929	0.00002
4	6	0	-1.46392	-0.13282	-0.00002
5	6	0	-0.70288	1.05062	0.00000
6	6	0	0.70295	1.05058	0.00000
7	1	0	2.54587	-0.13325	-0.00001
8	1	0	-2.54587	-0.13308	-0.00001
9	1	0	-1.22419	2.00252	0.00002
10	1	0	1.22432	2.00245	0.00002

Free Energy and Geometry for Transition State TS1

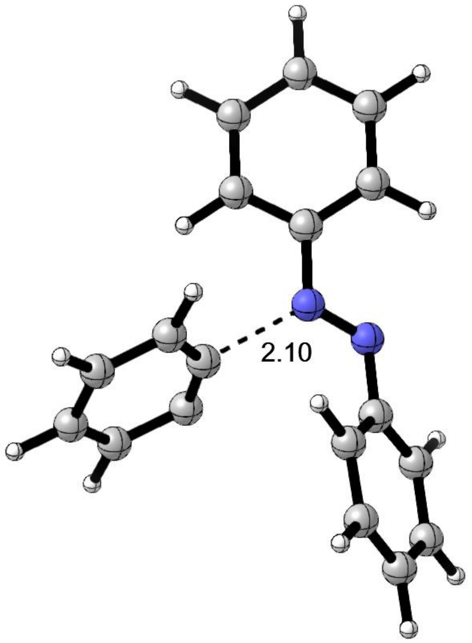

Sum of electronic and thermal free energies: -802.656165 a.u.

Number of imaginary frequencies: 1

Center Number	Atomic Number	Atomic Type	Coordinates (Ångstroms) X	Y	Z
1	7	0	-0.58971	-0.49146	-0.09032
2	6	0	1.44384	-1.49650	0.00922
3	6	0	2.19406	-2.28051	0.88739
4	1	0	1.67766	-2.84102	1.65818
5	6	0	3.57851	-2.30332	0.77255
6	6	0	4.20294	-1.58551	-0.24579
7	6	0	2.06308	-0.78909	-1.02547
8	6	0	3.44421	-0.84191	-1.15062
9	1	0	3.93259	-0.30411	-1.95477
10	1	0	1.45861	-0.22754	-1.72778
11	1	0	4.16906	-2.88866	1.46735
12	6	0	0.23966	1.42339	0.10552
13	6	0	0.85622	1.65246	1.20643
14	6	0	-0.05265	2.13700	-1.02962
15	6	0	1.36553	2.96595	1.29970
16	6	0	0.46363	3.43495	-0.92682
17	1	0	-0.60509	1.77442	-1.88849
18	6	0	1.16144	3.83672	0.22381
19	1	0	1.91051	3.31679	2.17230
20	1	0	0.31991	4.13220	-1.74526
21	1	0	1.55113	4.84918	0.27268

22	1	0	5.28196	-1.61621	-0.34505
23	6	0	-2.01109	-0.60853	-0.02496
24	6	0	-2.63130	-1.83002	-0.30480
25	6	0	-2.77167	0.52071	0.28202
26	6	0	-4.01600	-1.91890	-0.26309
27	1	0	-2.02044	-2.68677	-0.56192
28	6	0	-4.15782	0.41579	0.33407
29	1	0	-2.28054	1.45909	0.50519
30	6	0	-4.78188	-0.79856	0.05974
31	1	0	-4.50048	-2.86206	-0.48776
32	1	0	-4.74990	1.28676	0.58926
33	1	0	-5.86285	-0.87205	0.09131
34	7	0	0.04309	-1.52638	0.19018

Free Energy and Geometry for Transition State (*cis*-TS1)

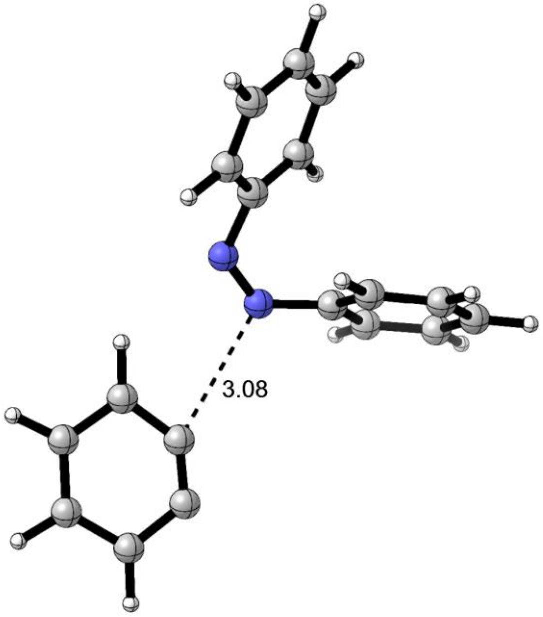

Sum of electronic and thermal free energies: -802.647161 a.u.

Number of imaginary frequencies: 1

Center Number	Atomic Number	Atomic Type	Coordinates (Ångstroms) X	Y	Z
1	6	0	3.16003	-1.19419	-0.36631
2	6	0	3.23495	0.13259	-0.01364
3	6	0	4.19414	0.84028	0.34581
4	6	0	5.49336	0.35896	0.46857
5	6	0	5.57020	-1.00454	0.13576
6	6	0	4.44872	-1.75296	-0.26599
7	1	0	2.28028	-1.74711	-0.66837
8	1	0	6.36326	0.92610	0.77613
9	1	0	6.53334	-1.50252	0.18986
10	1	0	4.58036	-2.80281	-0.50843
11	7	0	0.18812	0.07032	-0.46597
12	7	0	-0.26404	-1.08591	-0.42891
13	6	0	-1.65480	-1.37343	-0.22941
14	6	0	-1.96811	-2.37494	0.68758
15	6	0	-2.65117	-0.79016	-1.01443
16	6	0	-3.29643	-2.74295	0.87514
17	1	0	-1.16744	-2.84408	1.24879
18	6	0	-3.97163	-1.18927	-0.84442
19	1	0	-2.38929	-0.03883	-1.75089
20	6	0	-4.29965	-2.15245	0.10927
21	1	0	-3.54598	-3.50427	1.60521
22	1	0	-4.74794	-0.74490	-1.45667

23	1	0	-5.33254	-2.45235	0.24326
24	6	0	-0.59053	1.22907	-0.14533
25	6	0	-1.33089	1.32814	1.03467
26	6	0	-0.45896	2.32522	-0.99598
27	6	0	-1.97680	2.52192	1.33304
28	1	0	-1.38865	0.48304	1.71129
29	6	0	-1.13920	3.50361	-0.70731
30	1	0	0.17016	2.23556	-1.87471
31	6	0	-1.89801	3.60418	0.45701
32	1	0	-2.54496	2.60750	2.25218
33	1	0	-1.05846	4.35011	-1.37942
34	1	0	-2.41206	4.52915	0.69169

Free Energy and Geometry for Intermediate 9a (conformer 1 of 2)

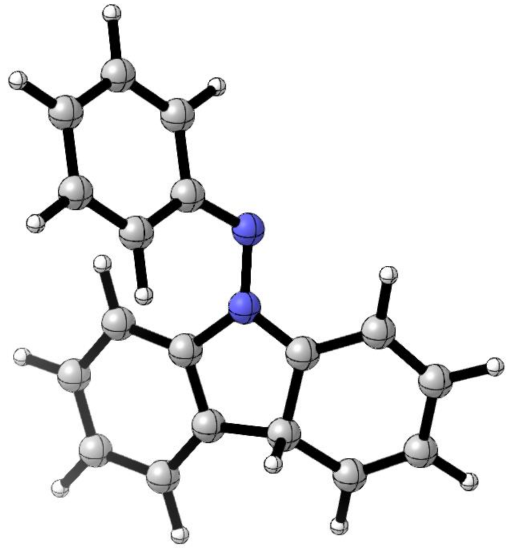

Sum of electronic and thermal free energies: -802.741372 a.u.

Number of imaginary frequencies: 0

Center Number	Atomic Number	Atomic Type	Coordinates (Ångstroms) X	Y	Z
1	6	0	2.15121	2.53616	-0.25301
2	6	0	1.70110	1.22539	-0.21646
3	6	0	0.40292	0.94587	0.20927
4	6	0	-0.47357	1.93010	0.63893
5	6	0	-0.00752	3.24333	0.60684
6	6	0	1.28276	3.54529	0.16501
7	7	0	0.20903	-0.48175	0.22736
8	6	0	1.33431	-1.07101	-0.13971
9	6	0	2.35396	-0.07624	-0.58536
10	6	0	3.74329	-0.46381	-0.17572
11	6	0	4.01008	-1.76146	0.04863
12	6	0	2.96387	-2.77947	0.02104
13	6	0	1.64592	-2.45771	-0.04155
14	1	0	2.35692	-0.12397	-1.69352
15	7	0	-0.82872	-1.13289	0.79402
16	6	0	-2.06510	-0.80105	0.27697
17	6	0	-3.20199	-1.16806	1.03009
18	6	0	-4.48150	-0.90413	0.56895
19	6	0	-4.68582	-0.26851	-0.65979
20	6	0	-3.57408	0.08075	-1.42243
21	6	0	-2.28357	-0.18525	-0.97447
22	1	0	3.15740	2.77206	-0.58169
23	1	0	-1.46911	1.68722	0.98664
24	1	0	-0.66011	4.04350	0.93558

25	1	0	1.61629	4.57639	0.15723
26	1	0	4.51920	0.29363	-0.15618
27	1	0	5.02266	-2.07780	0.27181
28	1	0	3.25809	-3.81761	0.12580
29	1	0	0.84955	-3.18281	0.07098
30	1	0	-3.04245	-1.65816	1.98486
31	1	0	-5.33389	-1.19423	1.17491
32	1	0	-5.68824	-0.06258	-1.01632
33	1	0	-3.70943	0.55690	-2.38848
34	1	0	-1.43667	0.06882	-1.60475

Free Energy and Geometry for Intermediate 9a (conformer 2 of 2)

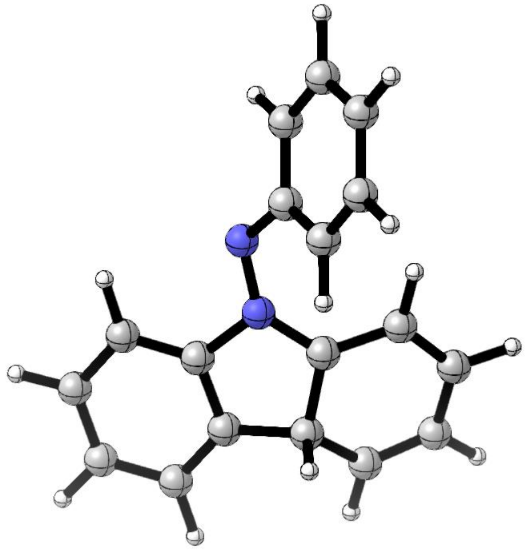

Sum of electronic and thermal free energies: -802.741633 a.u.

Number of imaginary frequencies: 0

Center Number	Atomic Number	Atomic Type	Coordinates (Ångstroms) X	Y	Z
1	6	0	3.74207	-0.55697	0.45813
2	6	0	2.43339	-0.12988	0.29610
3	6	0	1.45094	-1.04456	-0.05533
4	6	0	1.69646	-2.39107	-0.25534
5	6	0	3.01893	-2.80897	-0.11093
6	6	0	4.02744	-1.90628	0.23649
7	7	0	0.18341	-0.38031	-0.17610
8	6	0	0.36382	0.92952	0.03166
9	6	0	1.76341	1.20029	0.50487
10	6	0	2.32858	2.46442	-0.07208
11	6	0	1.49684	3.42365	-0.50746
12	6	0	0.05427	3.22360	-0.53914
13	6	0	-0.50842	2.00945	-0.30554
14	1	0	1.73222	1.35655	1.60196
15	7	0	-0.84564	-1.11880	-0.57158
16	6	0	-2.12643	-0.74251	-0.19581
17	6	0	-3.18155	-1.22771	-0.99288
18	6	0	-4.50619	-1.00671	-0.64333
19	6	0	-4.82841	-0.30468	0.51876
20	6	0	-3.79350	0.16702	1.32533
21	6	0	-2.46335	-0.03769	0.97814
22	1	0	4.52710	0.13531	0.74249
23	1	0	0.88972	-3.06438	-0.51465
24	1	0	3.26801	-3.85179	-0.26904

25	1	0	5.04674	-2.26072	0.33797
26	1	0	3.40348	2.60910	-0.04685
27	1	0	1.89264	4.36866	-0.86222
28	1	0	-0.57719	4.05506	-0.82993
29	1	0	-1.56900	1.85001	-0.45052
30	1	0	-2.92573	-1.78583	-1.88716
31	1	0	-5.29537	-1.38797	-1.28285
32	1	0	-5.86276	-0.13223	0.79220
33	1	0	-4.02303	0.70271	2.24078
34	1	0	-1.68087	0.33377	1.63170

Free Energy and Geometry for Intermediate *trans*-9b (Conformer 1 of 2)

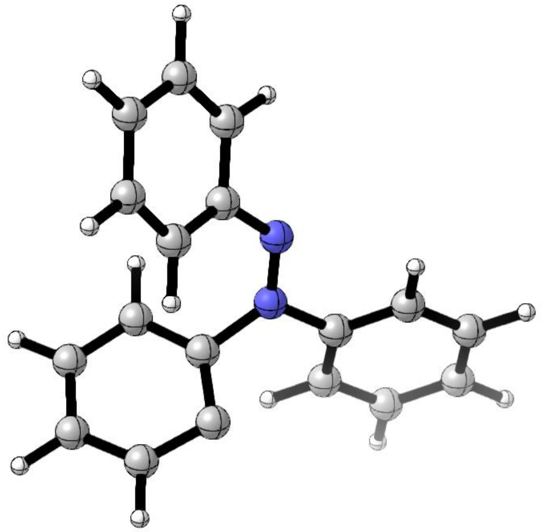

Sum of electronic and thermal free energies: -802.674898 a.u.

Number of imaginary frequencies: 0

Center Number	Atomic Number	Atomic Type	Coordinates (Ångstroms) X	Y	Z
1	6	0	0.14226	1.16892	0.05051
2	6	0	-0.63928	1.57137	1.14076
3	6	0	-1.12995	2.86484	1.11282
4	6	0	-0.83940	3.67056	0.00039
5	6	0	-0.04801	3.19404	-1.04288
6	6	0	0.52692	1.89564	-1.07083
7	7	0	0.55846	-0.24026	0.06661
8	7	0	-0.20886	-1.23072	0.15055
9	6	0	1.96627	-0.53753	0.01046
10	6	0	-1.60559	-1.15935	0.02732
11	6	0	2.38378	-1.74191	-0.55079
12	6	0	3.73937	-2.04099	-0.56608
13	6	0	4.65983	-1.14524	-0.02357
14	6	0	4.22576	0.05759	0.52992
15	6	0	2.87335	0.37514	0.54376
16	6	0	-2.25706	-2.25351	0.61537
17	6	0	-3.63656	-2.37557	0.53042
18	6	0	-4.36486	-1.43862	-0.19850
19	6	0	-3.71154	-0.37966	-0.83276
20	6	0	-2.34025	-0.21387	-0.71248
21	1	0	-0.85632	0.89953	1.96708
22	1	0	-1.72414	3.24752	1.93479
23	1	0	-1.23004	4.68433	-0.03334

24	1	0	0.14141	3.87575	-1.87152
25	1	0	1.65315	-2.41331	-0.98367
26	1	0	4.07901	-2.96738	-1.01350
27	1	0	5.71771	-1.38044	-0.04227
28	1	0	4.94178	0.75580	0.94610
29	1	0	2.52173	1.31048	0.95889
30	1	0	-1.65487	-2.98997	1.13478
31	1	0	-4.13742	-3.20875	1.00834
32	1	0	-5.44083	-1.53698	-0.28551
33	1	0	-4.27921	0.33324	-1.41860
34	1	0	-1.84950	0.61582	-1.20490

Free Energy and Geometry for Intermediate *trans*-9b (Conformer 2 of 2)

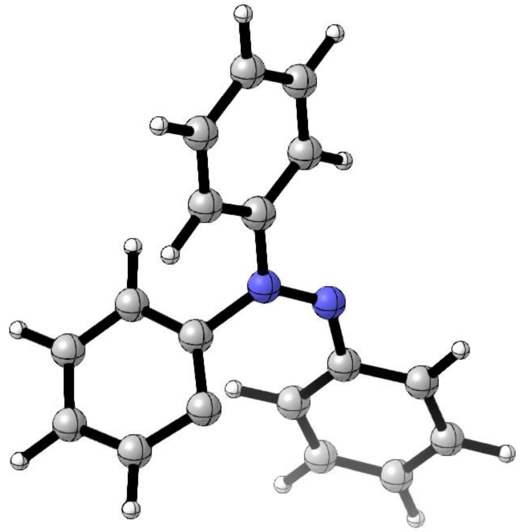

Sum of electronic and thermal free energies: -802.674165 a.u.

Number of imaginary frequencies: 0

Center Number	Atomic Number	Atomic Type	Coordinates (Ångstroms) X	Y	Z
1	6	0	-0.15034	1.15715	-0.10059
2	6	0	-0.59164	1.89811	1.00222
3	6	0	-0.24125	3.23952	1.03747
4	6	0	0.47766	3.77112	-0.04109
5	6	0	0.82639	2.97332	-1.13237
6	6	0	0.55346	1.58550	-1.22122
7	7	0	-0.48862	-0.27781	-0.06719
8	7	0	0.32408	-1.22851	-0.14491
9	6	0	-1.88219	-0.64376	-0.03667
10	6	0	1.71293	-1.10164	-0.03309
11	6	0	-2.25722	-1.84701	0.55873
12	6	0	-3.60066	-2.19705	0.57752
13	6	0	-4.55218	-1.35429	0.00370
14	6	0	-4.16101	-0.15519	-0.58755
15	6	0	-2.82076	0.21409	-0.60668
16	6	0	2.43575	-0.08352	0.61751
17	6	0	3.81147	-0.20060	0.72019
18	6	0	4.48295	-1.28839	0.15457
19	6	0	3.77000	-2.29765	-0.48563
20	6	0	2.38629	-2.21838	-0.55074
21	1	0	-1.16982	1.44207	1.80176
22	1	0	-0.53011	3.86324	1.87578
23	1	0	0.75216	4.82280	-0.02730
24	1	0	1.35267	3.46276	-1.95175

25	1	0	-1.50246	-2.48174	1.00593
26	1	0	-3.90644	-3.12424	1.04724
27	1	0	-5.59972	-1.63172	0.02108
28	1	0	-4.89910	0.49557	-1.04054
29	1	0	-2.49727	1.13449	-1.07635
30	1	0	1.93767	0.77582	1.04396
31	1	0	4.37182	0.57421	1.22937
32	1	0	5.56317	-1.34778	0.22334
33	1	0	4.28595	-3.14803	-0.91438
34	1	0	1.79530	-3.00134	-1.01118

Free Energy and Geometry for Intermediate diMe-*trans*-9b

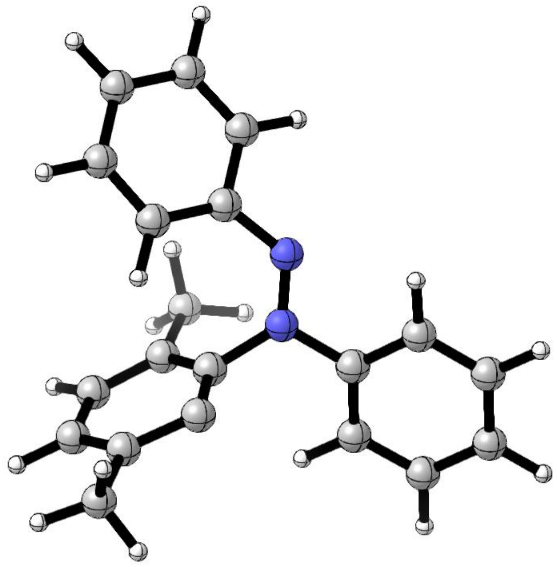

Sum of electronic and thermal free energies: -881.173558 a.u.

Number of imaginary frequencies: 0

Center Number	Atomic Number	Atomic Type	Coordinates (Ångstroms) X	Y	Z
1	6	0	0.17097	0.89955	0.19950
2	6	0	-0.53277	1.15657	1.38970
3	6	0	-1.03578	2.44680	1.48130
4	6	0	-0.83947	3.35942	0.43352
5	6	0	-0.10694	3.02681	-0.70683
6	6	0	0.47405	1.73789	-0.85765
7	7	0	0.59902	-0.49314	-0.02107
8	7	0	-0.16535	-1.48036	-0.15336
9	6	0	2.00974	-0.77226	-0.08447
10	6	0	-1.55991	-1.39538	-0.25414
11	6	0	2.46132	-1.86748	-0.81696
12	6	0	3.81996	-2.15213	-0.82913
13	6	0	4.71060	-1.35228	-0.11365
14	6	0	4.24288	-0.25843	0.61124
15	6	0	2.88686	0.04517	0.62425
16	6	0	-2.18251	-2.62125	0.03696
17	6	0	-3.56094	-2.74682	-0.03827
18	6	0	-4.32545	-1.66152	-0.46199
19	6	0	-3.70689	-0.45779	-0.80315
20	6	0	-2.33308	-0.29986	-0.68760
21	1	0	-1.57405	2.74979	2.37475
22	1	0	-1.25310	4.36125	0.53351
23	1	0	1.75424	-2.46455	-1.37850

24	1	0	4.18621	-2.99201	-1.40735
25	1	0	5.77094	-1.57625	-0.13015
26	1	0	4.93491	0.36646	1.16270
27	1	0	2.50884	0.90066	1.16952
28	1	0	-1.55326	-3.45530	0.32546
29	1	0	-4.03474	-3.68867	0.21051
30	1	0	-5.40275	-1.75423	-0.53876
31	1	0	-4.30331	0.37579	-1.15418
32	1	0	-1.87644	0.64346	-0.95501
33	6	0	-0.73536	0.14417	2.48503
34	1	0	-0.89490	0.64686	3.44134
35	1	0	-1.61156	-0.48746	2.29769
36	1	0	0.12878	-0.51900	2.59574
37	6	0	0.09474	4.06027	-1.78924
38	1	0	-0.42490	4.99793	-1.57015
39	1	0	1.15952	4.27798	-1.91496
40	1	0	-0.26223	3.68287	-2.75173

Free Energy and Geometry for Intermediate diMe-*trans*-9b′

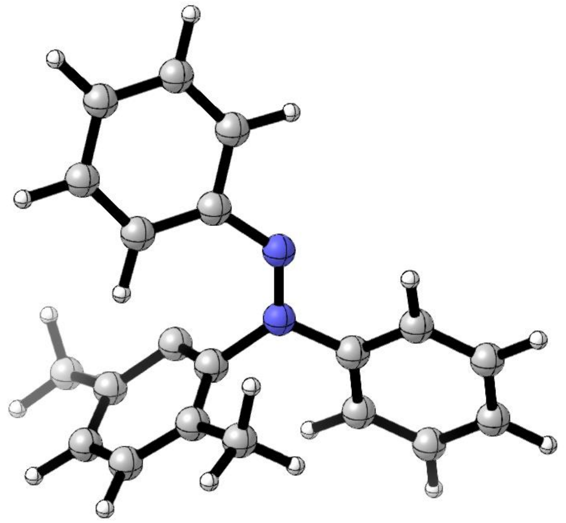

Sum of electronic and thermal free energies: -881.173558 a.u.

Number of imaginary frequencies: 0

Center Number	Atomic Number	Atomic Type	Coordinates (Ångstroms) X	Y	Z
1	6	0	-0.09592	0.93499	0.05269
2	6	0	-0.25373	1.45353	1.34887
3	6	0	0.27216	2.72743	1.52185
4	6	0	0.86232	3.39496	0.43920
5	6	0	0.93767	2.81672	-0.82979
6	6	0	0.46616	1.49796	-1.07451
7	7	0	-0.56973	-0.46042	-0.13655
8	7	0	0.15928	-1.46941	-0.27076
9	6	0	-1.98898	-0.68397	-0.24342
10	6	0	1.54619	-1.49765	-0.11525
11	6	0	-2.51318	-1.93249	0.09083
12	6	0	-3.88492	-2.12938	0.01092
13	6	0	-4.72059	-1.09014	-0.39803
14	6	0	-4.18205	0.15188	-0.72463
15	6	0	-2.81034	0.36748	-0.64433
16	6	0	2.41019	-0.52956	0.44045
17	6	0	3.76264	-0.81862	0.53807
18	6	0	4.27717	-2.03068	0.07357
19	6	0	3.42944	-2.98988	-0.47545
20	6	0	2.07111	-2.72872	-0.55149
21	1	0	0.22307	3.20110	2.49805
22	1	0	1.26873	4.39166	0.60112
23	1	0	-1.84980	-2.72173	0.42110
24	1	0	-4.30402	-3.09233	0.27761

25	1	0	-5.79111	-1.24889	-0.45703
26	1	0	-4.82890	0.95842	-1.04825
27	1	0	-2.37314	1.32166	-0.90960
28	1	0	2.04721	0.42282	0.79683
29	1	0	4.42809	-0.08054	0.96930
30	1	0	5.34129	-2.22540	0.14490
31	1	0	3.82332	-3.93386	-0.83144
32	1	0	1.37713	-3.45636	-0.95544
33	6	0	-0.88656	0.68048	2.47522
34	1	0	-1.96310	0.54746	2.32306
35	1	0	-0.44895	-0.31953	2.57825
36	1	0	-0.74653	1.20289	3.42374
37	6	0	1.55632	3.59980	-1.96352
38	1	0	1.91353	4.58338	-1.64324
39	1	0	2.39597	3.04881	-2.39668
40	1	0	0.82968	3.74446	-2.76869

Free Energy and Geometry for Intermediate *cis*-9b

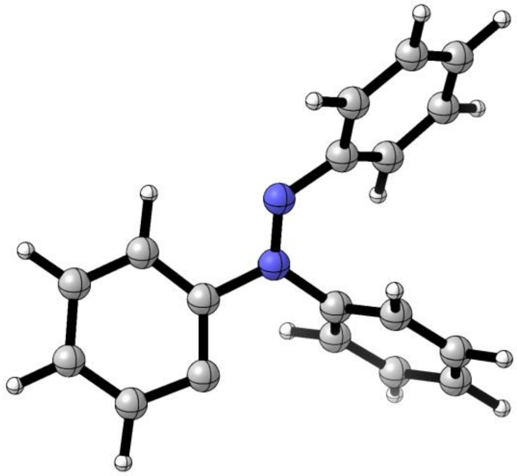

Sum of electronic and thermal free energies: -802.672580 a.u.

Number of imaginary frequencies: 0

Center Number	Atomic Number	Atomic Type	Coordinates (Ångstroms) X	Y	Z
1	6	0	4.21104	0.16090	0.45685
2	6	0	2.83890	0.52423	0.48725
3	6	0	2.02379	-0.51906	0.02681
4	6	0	2.43378	-1.78409	-0.44999
5	6	0	3.78399	-2.05270	-0.45419
6	6	0	4.67776	-1.06956	0.01479
7	7	0	0.60115	-0.29205	0.07197
8	7	0	-0.17217	-1.28529	0.12806
9	6	0	0.13285	1.08649	0.10624
10	6	0	-1.56941	-1.20426	-0.01456
11	6	0	-2.25494	-0.35375	-0.89552
12	6	0	-3.63368	-0.46163	-1.00499
13	6	0	-4.33945	-1.38624	-0.23328
14	6	0	-3.65570	-2.23999	0.62866
15	6	0	-2.27133	-2.17112	0.71530
16	6	0	-0.68172	1.47879	1.15806
17	6	0	-1.18589	2.77445	1.16136
18	6	0	-0.88202	3.64395	0.11600
19	6	0	-0.05288	3.23038	-0.92631
20	6	0	0.47460	1.94504	-0.93032
21	1	0	4.95411	0.88099	0.79762
22	1	0	1.71498	-2.50726	-0.81901
23	1	0	4.15451	-3.00030	-0.82821
24	1	0	5.74393	-1.28129	0.01248
25	1	0	-1.71845	0.36493	-1.50186
26	1	0	-4.16282	0.18283	-1.69701
27	1	0	-5.41793	-1.44995	-0.31870

28	1	0	-4.19615	-2.97334	1.21516
29	1	0	-1.70958	-2.84784	1.34870
30	1	0	-0.90455	0.78556	1.96099
31	1	0	-1.81169	3.10469	1.98174
32	1	0	-1.28100	4.65171	0.11989
33	1	0	0.19688	3.91468	-1.72818
34	1	0	1.13664	1.60505	-1.71680

Free Energy and Geometry for Intermediate *cis*-9b′

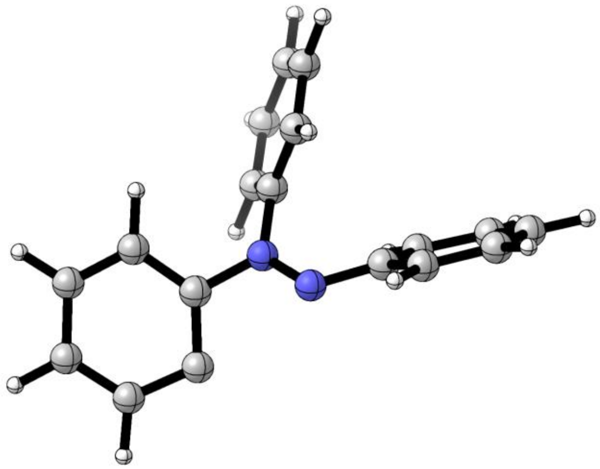

Sum of electronic and thermal free energies: -802.669508 a.u.

Number of imaginary frequencies: 0

Center Number	Atomic Number	Atomic Type	Coordinates (Ångstroms) X	Y	Z
1	6	0	3.69147	-2.06635	0.61503
2	6	0	2.29998	-1.82844	0.64278
3	6	0	1.94589	-0.65438	-0.05081
4	6	0	2.83589	0.26444	-0.64406
5	6	0	4.18841	-0.00396	-0.56798
6	6	0	4.61583	-1.17662	0.07394
7	7	0	0.55056	-0.35979	-0.13159
8	7	0	-0.22556	-1.35528	-0.26363
9	6	0	0.11885	1.02299	-0.09561
10	6	0	-1.60909	-1.22638	-0.04498
11	6	0	-2.42067	-1.99486	-0.88581
12	6	0	-3.80054	-1.96788	-0.72724
13	6	0	-4.36708	-1.22350	0.30518
14	6	0	-3.55034	-0.50745	1.18219
15	6	0	-2.17385	-0.49694	1.01082
16	6	0	-0.70487	1.49952	-1.11020
17	6	0	-1.10594	2.82965	-1.07541
18	6	0	-0.68603	3.65906	-0.03630
19	6	0	0.15067	3.16705	0.96398
20	6	0	0.57007	1.84129	0.93391
21	1	0	4.08161	-2.96586	1.08954
22	1	0	2.47600	1.13373	-1.18672
23	1	0	4.90629	0.67285	-1.01684
24	1	0	5.68030	-1.37977	0.15479
25	1	0	-1.94868	-2.59497	-1.65509
26	1	0	-4.43094	-2.54546	-1.39285
27	1	0	-5.44203	-1.21831	0.44325

28	1	0	-3.99020	0.03981	2.00763
29	1	0	-1.53811	0.03941	1.70501
30	1	0	-1.02028	0.83665	-1.90803
31	1	0	-1.74239	3.21908	-1.86087
32	1	0	-1.00511	4.69449	-0.01149
33	1	0	0.48111	3.81593	1.76615
34	1	0	1.22553	1.43448	1.69546

Free Energy and Geometry for Transition State TS2

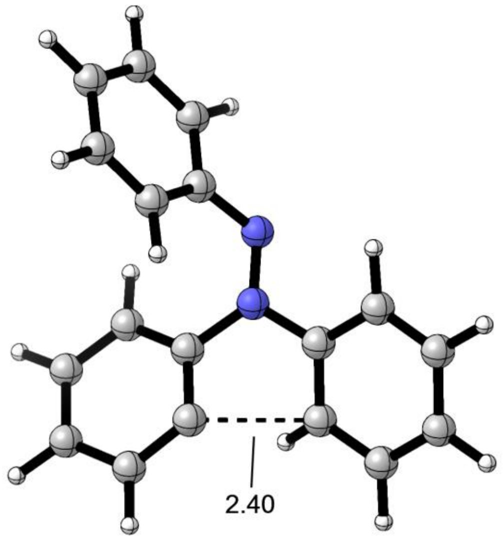

Sum of electronic and thermal free energies: -802.663547 a.u.

Number of imaginary frequencies: 1

Center Number	Atomic Number	Atomic Type	Coordinates (Ångstroms) X	Y	Z
1	6	0	-0.28776	1.07649	-0.20383
2	6	0	0.77476	1.77713	-0.79936
3	6	0	0.85443	3.14020	-0.59565
4	6	0	-0.14742	3.80408	0.13257
5	6	0	-1.25556	3.10267	0.59792
6	6	0	-1.29163	1.70549	0.52675
7	7	0	-0.40532	-0.33762	-0.42898
8	7	0	0.53479	-1.16557	-0.78229
9	6	0	-1.70367	-0.82069	-0.31869
10	6	0	1.82019	-0.99064	-0.26081
11	6	0	-1.90998	-2.14256	0.12140
12	6	0	-3.17842	-2.54668	0.46760
13	6	0	-4.25892	-1.64142	0.38371
14	6	0	-4.06682	-0.35374	-0.06584
15	6	0	-2.77937	0.08674	-0.43817
16	6	0	2.87108	-1.54944	-1.00299
17	6	0	4.17498	-1.48940	-0.52940
18	6	0	4.44773	-0.89839	0.70369
19	6	0	3.39965	-0.37598	1.46318
20	6	0	2.09475	-0.41523	0.99240
21	1	0	1.47510	1.26571	-1.45202
22	1	0	1.66597	3.70511	-1.03958
23	1	0	-0.06131	4.87163	0.31139
24	1	0	-2.05800	3.64886	1.08756

25	1	0	-1.05160	-2.79374	0.22910
26	1	0	-3.34306	-3.55203	0.83600
27	1	0	-5.25396	-1.97290	0.65918
28	1	0	-4.90713	0.32099	-0.17394
29	1	0	-2.68828	0.96414	-1.06276
30	1	0	2.63696	-2.01935	-1.95180
31	1	0	4.98005	-1.91301	-1.11921
32	1	0	5.46420	-0.85743	1.07744
33	1	0	3.60089	0.06203	2.43444
34	1	0	1.28571	-0.01940	1.59609

Free Energy and Geometry for Intermediate diMe-TS2

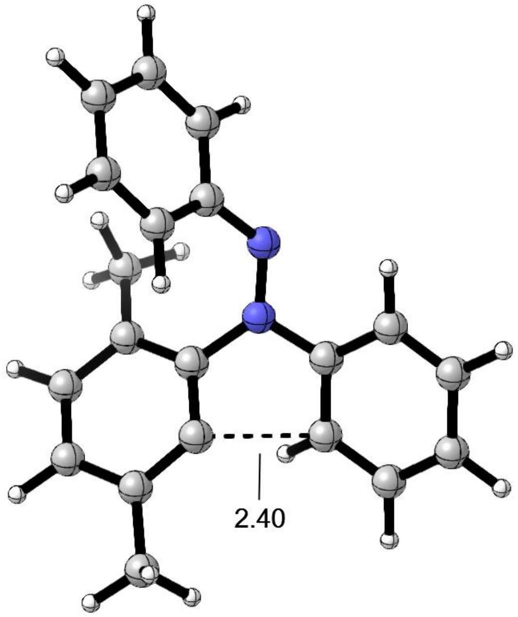

Sum of electronic and thermal free energies: -881.159035 a.u.

Number of imaginary frequencies: 1

Center Number	Atomic Number	Atomic Type	Coordinates (Ångstroms) X	Y	Z
1	6	0	0.33586	0.75293	0.29032
2	6	0	-0.56985	1.62528	0.94730
3	6	0	-0.37049	2.98015	0.71669
4	6	0	0.71569	3.45809	-0.02552
5	6	0	1.69354	2.58686	-0.50574
6	6	0	1.43569	1.21659	-0.43689
7	7	0	0.21754	-0.67084	0.42524
8	7	0	-0.86368	-1.37566	0.61845
9	6	0	1.43073	-1.34618	0.35401
10	6	0	-2.05268	-0.97325	0.01513
11	6	0	1.46379	-2.66295	-0.14401
12	6	0	2.67592	-3.24128	-0.44366
13	6	0	3.87345	-2.51656	-0.26122
14	6	0	3.84883	-1.23263	0.23692
15	6	0	2.62192	-0.61743	0.56245
16	6	0	-3.22462	-1.50729	0.57735
17	6	0	-4.46732	-1.20815	0.03773
18	6	0	-4.56351	-0.38959	-1.08768
19	6	0	-3.40041	0.11056	-1.67491
20	6	0	-2.15181	-0.17214	-1.13712
21	1	0	-1.04567	3.68916	1.18640
22	1	0	0.80794	4.52698	-0.20389

23	1	0	0.52660	-3.17217	-0.32926
24	1	0	2.70861	-4.24429	-0.85186
25	1	0	4.82171	-2.98419	-0.50192
26	1	0	4.77243	-0.69586	0.41676
27	1	0	2.62932	0.24959	1.20795
28	1	0	-3.12658	-2.14536	1.44892
29	1	0	-5.36299	-1.61684	0.49188
30	1	0	-5.53252	-0.15506	-1.51273
31	1	0	-3.46595	0.72407	-2.56656
32	1	0	-1.25820	0.21049	-1.61587
33	6	0	-1.60117	1.19774	1.95751
34	1	0	-2.59058	1.08569	1.50147
35	1	0	-1.35378	0.24269	2.42723
36	1	0	-1.67597	1.95381	2.74202
37	6	0	2.94629	3.10216	-1.16706
38	1	0	3.02211	4.18972	-1.09125
39	1	0	3.83411	2.66476	-0.70058
40	1	0	2.97326	2.82483	-2.22410

Free Energy and Geometry for Transition State TS3

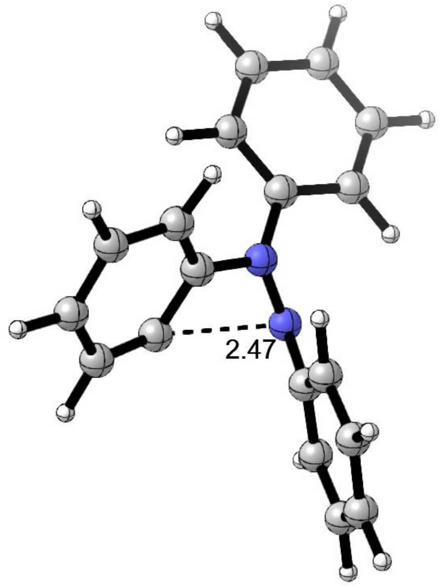

Sum of electronic and thermal free energies: -802.663833 a.u.

Number of imaginary frequencies: 1

Center Number	Atomic Number	Atomic Type	Coordinates (Ångstroms)		
			X	Y	Z
1	7	0	0.60762	-0.09507	-0.12820
2	6	0	-1.51194	-1.09818	-0.25320
3	6	0	-2.40031	-1.78898	-1.08754
4	1	0	-2.06586	-2.06421	-2.08118
5	6	0	-3.68313	-2.08139	-0.64981
6	6	0	-4.09024	-1.72082	0.63556
7	6	0	-1.91026	-0.75422	1.04777
8	6	0	-3.19184	-1.07081	1.48035
9	1	0	-3.48945	-0.80879	2.48967
10	1	0	-1.21679	-0.25642	1.71600
11	1	0	-4.36813	-2.60060	-1.31065
12	6	0	0.09582	1.19923	-0.08722
13	6	0	-0.78670	1.42687	-1.16956
14	6	0	0.34205	2.15077	0.90681
15	6	0	-1.55486	2.58246	-1.13705
16	6	0	-0.32637	3.36353	0.81892
17	1	0	0.98887	1.91898	1.74768
18	6	0	-1.26637	3.57828	-0.19651
19	1	0	-2.30706	2.77332	-1.89777
20	1	0	-0.14799	4.13390	1.56005
21	1	0	-1.76027	4.54252	-0.26829
22	1	0	-5.09084	-1.95543	0.97924

23	6	0	1.98157	-0.43156	-0.04214
24	6	0	2.32384	-1.77204	0.14677
25	6	0	2.96734	0.55137	-0.15616
26	6	0	3.66412	-2.13111	0.20388
27	1	0	1.53609	-2.50869	0.24914
28	6	0	4.30422	0.17899	-0.08020
29	1	0	2.69014	1.58397	-0.32875
30	6	0	4.65656	-1.15789	0.09645
31	1	0	3.93437	-3.17075	0.34676
32	1	0	5.07410	0.93566	-0.17574
33	1	0	5.70148	-1.44015	0.15131
34	7	0	-0.20185	-0.93784	-0.73468

Free Energy and Geometry for Intermediate diMe-TS3

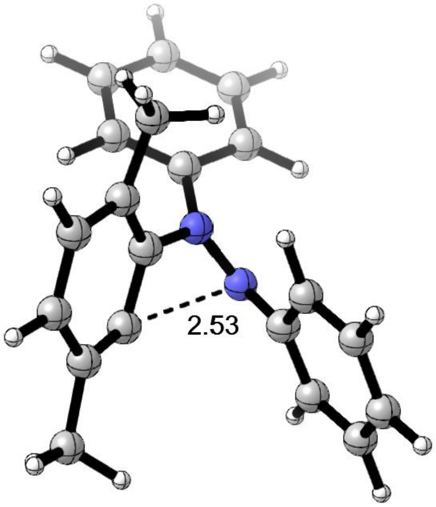

Sum of electronic and thermal free energies: -881.162848 a.u.

Number of imaginary frequencies: 1

Center Number	Atomic Number	Atomic Type	Coordinates (Ångstroms) X	Y	Z
1	6	0	0.72063	-0.29865	-0.15340
2	6	0	-1.30662	-1.47224	-0.17403
3	6	0	-2.18740	-2.19504	-0.98823
4	6	0	-1.88263	-2.42214	-2.00327
5	6	0	-3.43129	-2.57256	-0.50479
6	6	0	-3.80173	-2.26855	0.80595
7	7	0	-1.66512	-1.18726	1.15268
8	7	0	-2.90567	-1.58950	1.63049
9	6	0	-3.17301	-1.37194	2.65862
10	6	0	-0.97138	-0.66717	1.80463
11	6	0	-4.11388	-3.11371	-1.15037
12	6	0	0.08053	0.93737	-0.02316
13	6	0	-0.85782	1.13639	-1.06576
14	6	0	0.26197	1.82603	1.05240
15	6	0	-1.74945	2.20145	-0.95480
16	6	0	-0.53830	2.96206	1.03198
17	6	0	-1.51197	3.15260	0.04439
18	6	0	-0.43470	3.69304	1.82809
19	6	0	-2.09610	4.07020	0.05103
20	6	0	-4.77142	-2.56942	1.18484
21	1	0	2.12862	-0.46950	-0.22181
22	1	0	2.65373	-1.75108	-0.05438
23	1	0	2.95891	0.62297	-0.47571

24	1	0	4.02629	-1.94034	-0.15395
25	1	0	1.98153	-2.57501	0.15355
26	1	0	4.33122	0.42135	-0.56028
27	1	0	2.52714	1.60573	-0.62615
28	1	0	4.86681	-0.85617	-0.40207
29	1	0	4.44148	-2.93326	-0.02756
30	1	0	4.98315	1.26226	-0.76562
31	1	0	5.93787	-1.00639	-0.47206
32	1	0	-0.02670	-1.22259	-0.69927
33	6	0	1.17899	1.52414	2.20652
34	1	0	2.22965	1.69431	1.95165
35	1	0	0.93630	2.15625	3.06282
36	1	0	1.09469	0.47786	2.51832
37	6	0	-2.87391	2.37243	-1.94426
38	1	0	-3.52615	1.49476	-1.93522
39	1	0	-3.48004	3.25108	-1.71059
40	1	0	-2.48667	2.47493	-2.96113

Free Energy and Geometry for Intermediate *trans*-9c

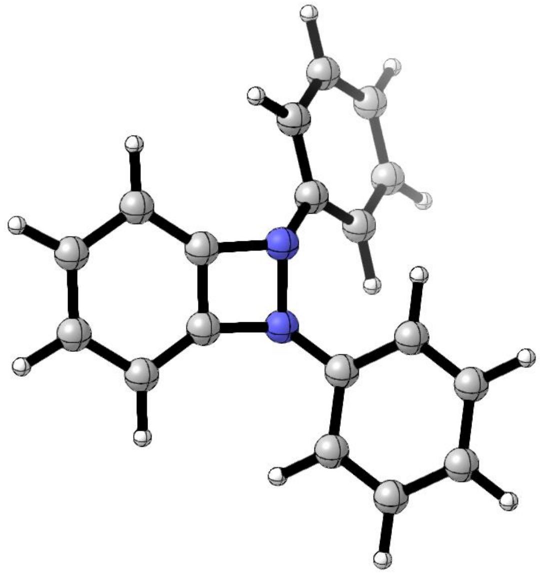

Sum of electronic and thermal free energies: -802.762762 a.u.

Number of imaginary frequencies: 0

Center Number	Atomic Number	Atomic Type	Coordinates (Ångstroms) X	Y	Z
1	6	0	0.60191	3.96277	0.35074
2	6	0	-0.60438	3.96240	-0.35048
3	6	0	-1.25458	2.77538	-0.74216
4	6	0	-0.58762	1.63255	-0.37030
5	6	0	0.58693	1.63289	0.37004
6	6	0	1.25298	2.77616	0.74220
7	7	0	0.58261	0.20614	0.43713
8	7	0	-0.58240	0.20579	-0.43756
9	6	0	1.68865	-0.60391	0.08906
10	6	0	-1.68812	-0.60463	-0.08923
11	6	0	1.54990	-1.67408	-0.79492
12	6	0	2.65188	-2.47859	-1.07484
13	6	0	3.88731	-2.22360	-0.48690
14	6	0	4.01593	-1.15151	0.39505
15	6	0	2.92351	-0.34529	0.69067
16	6	0	-1.54938	-1.67369	0.79607
17	6	0	-2.65115	-2.47842	1.07623
18	6	0	-3.88633	-2.22471	0.48722
19	6	0	-4.01492	-1.15373	-0.39611
20	6	0	-2.92270	-0.34736	-0.69200
21	1	0	1.05178	4.91385	0.61146
22	1	0	-1.05495	4.91319	-0.61101
23	1	0	-2.18212	2.79023	-1.29975

24	1	0	2.18048	2.79160	1.29985
25	1	0	0.59226	-1.86485	-1.26462
26	1	0	2.53954	-3.30759	-1.76457
27	1	0	4.74093	-2.85206	-0.71170
28	1	0	4.97211	-0.94301	0.86176
29	1	0	3.01945	0.47600	1.39235
30	1	0	-0.59192	-1.86342	1.26657
31	1	0	-2.53885	-3.30657	1.76698
32	1	0	-4.73978	-2.85331	0.71222
33	1	0	-4.97090	-0.94630	-0.86371
34	1	0	0.60191	3.96277	0.35074

Free Energy and Geometry for Transition State TS4

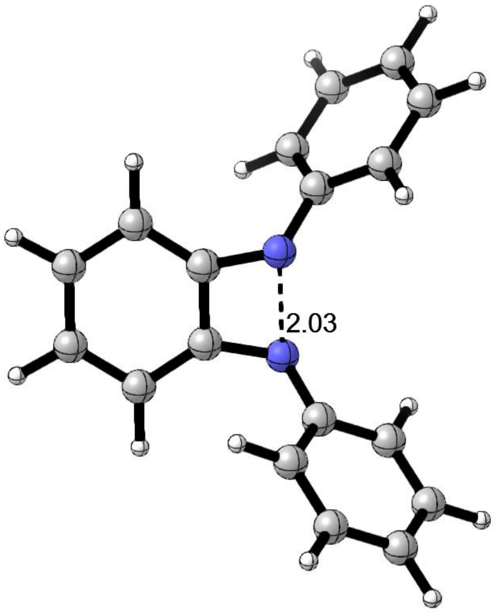

Sum of electronic and thermal free energies: -802.733023 a.u.

Number of imaginary frequencies: 1

Center Number	Atomic Number	Atomic Type	Coordinates (Ångstroms) X	Y	Z
1	6	0	1.35400	2.58452	0.49626
2	6	0	0.63325	1.40955	0.29516
3	6	0	-0.63325	1.40955	-0.29515
4	6	0	-1.35399	2.58453	-0.49626
5	6	0	-0.66194	3.76795	-0.23567
6	6	0	0.66195	3.76795	0.23566
7	7	0	-0.80685	0.06789	-0.61777
8	7	0	0.80685	0.06789	0.61777
9	6	0	-1.99494	-0.58100	-0.31401
10	6	0	1.99493	-0.58100	0.31401
11	6	0	-2.26897	-1.77968	-0.99679
12	6	0	-3.43965	-2.47722	-0.74162
13	6	0	-4.34618	-1.99523	0.20499
14	6	0	-4.07287	-0.81829	0.90407
15	6	0	-2.90097	-0.11723	0.65747
16	6	0	2.90097	-0.11724	-0.65747
17	6	0	4.07287	-0.81829	-0.90407
18	6	0	4.34617	-1.99523	-0.20499
19	6	0	3.43964	-2.47723	0.74162
20	6	0	2.26897	-1.77969	0.99678
21	1	0	2.36262	2.58894	0.89307
22	1	0	-2.36262	2.58895	-0.89307

23	1	0	-1.15137	4.71803	-0.41903
24	1	0	1.15138	4.71802	0.41902
25	1	0	-1.54662	-2.12660	-1.72710
26	1	0	-3.65180	-3.39570	-1.27672
27	1	0	-5.26056	-2.54214	0.40557
28	1	0	-4.77088	-0.45831	1.65134
29	1	0	-2.66311	0.78051	1.21811
30	1	0	2.66311	0.78051	-1.21810
31	1	0	4.77088	-0.45831	-1.65134
32	1	0	5.26056	-2.54215	-0.40557
33	1	0	3.65179	-3.39571	1.27672
34	1	0	1.54661	-2.12660	1.72710

Free Energy and Geometry for Intermediate *E*,*E*-9d

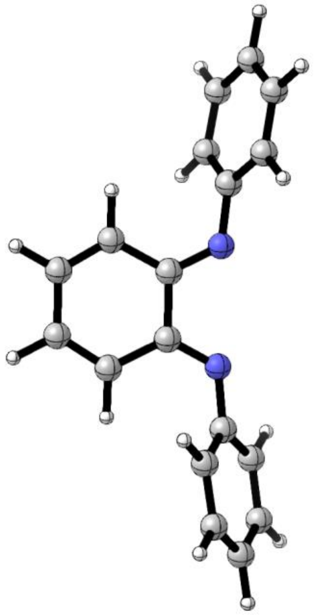

Sum of electronic and thermal free energies: -802.779909 a.u.

Number of imaginary frequencies: 0

Center Number	Atomic Number	Atomic Type	Coordinates (Ångstroms) X	Y	Z
1	6	0	1.43035	1.93177	0.02833
2	6	0	0.75483	0.63199	0.02552
3	6	0	-0.75483	0.63198	-0.02527
4	6	0	-1.43037	1.93174	-0.02840
5	6	0	-0.72626	3.07889	-0.02860
6	6	0	0.72623	3.07891	0.02831
7	7	0	-1.34452	-0.50375	-0.13915
8	7	0	1.34454	-0.50371	0.13949
9	6	0	-2.73572	-0.64501	-0.07762
10	6	0	2.73574	-0.64497	0.07776
11	6	0	-3.38450	-1.34948	-1.09949
12	6	0	-4.75878	-1.54751	-1.04688
13	6	0	-5.49776	-1.08767	0.04292
14	6	0	-4.84920	-0.42171	1.08062
15	6	0	-3.47826	-0.19386	1.02255
16	6	0	3.47810	-0.19408	-1.02263
17	6	0	4.84903	-0.42194	-1.08087
18	6	0	5.49776	-1.08765	-0.04311
19	6	0	4.75896	-1.54724	1.04692
20	6	0	3.38468	-1.34921	1.09969
21	1	0	2.51108	1.94537	0.09504
22	1	0	-2.51110	1.94531	-0.09517

23	1	0	-1.24216	4.03136	-0.07842
24	1	0	1.24211	4.03139	0.07791
25	1	0	-2.79396	-1.72711	-1.92651
26	1	0	-5.25372	-2.07835	-1.85249
27	1	0	-6.56671	-1.26060	0.08934
28	1	0	-5.41181	-0.07907	1.94204
29	1	0	-2.96498	0.30960	1.83537
30	1	0	2.96468	0.30917	-1.83549
31	1	0	5.41149	-0.07951	-1.94247
32	1	0	6.56670	-1.26059	-0.08967
33	1	0	5.25403	-2.07788	1.85257
34	1	0	2.79427	-1.72664	1.92690

Free Energy and Geometry for Transition State TS5

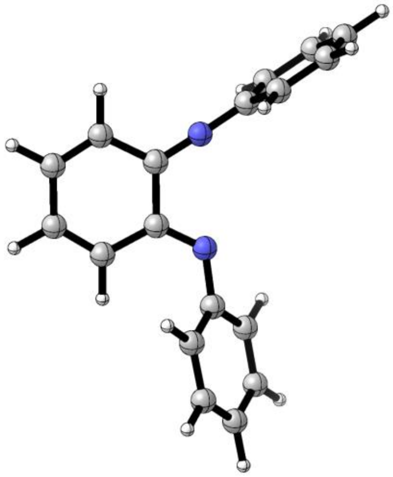

Sum of electronic and thermal free energies: -802.755487 a.u.

Number of imaginary frequencies: 1

Center Number	Atomic Number	Atomic Type	Coordinates (Ångstroms) X	Y	Z
1	6	0	0.91743	2.96717	0.00854
2	6	0	0.69392	1.51198	-0.04149
3	6	0	-0.75303	1.03824	-0.11574
4	6	0	-1.79284	2.05898	-0.25752
5	6	0	-1.47986	3.36783	-0.24782
6	6	0	-0.10776	3.83094	-0.08260
7	7	0	-0.93949	-0.23006	-0.08114
8	7	0	1.63728	0.69009	-0.00953
9	6	0	-2.21315	-0.81184	-0.02347
10	6	0	2.67839	-0.15531	0.03764
11	6	0	3.16014	-0.63604	1.27356
12	6	0	4.23991	-1.50763	1.30782
13	6	0	4.87155	-1.92611	0.13758
14	6	0	4.39379	-1.45218	-1.08329
15	6	0	3.31485	-0.58112	-1.14729
16	6	0	-3.14467	-0.46737	0.96544
17	6	0	-4.36888	-1.12468	1.02951
18	6	0	-4.68396	-2.11916	0.10635
19	6	0	-3.75303	-2.47140	-0.87059
20	6	0	-2.51605	-1.84087	-0.92342
21	1	0	1.94520	3.29790	0.10406
22	1	0	-2.81471	1.73300	-0.41034
23	1	0	-2.25869	4.11031	-0.38238
24	1	0	0.07527	4.89953	-0.05697
25	1	0	2.67097	-0.31941	2.18714
26	1	0	4.59106	-1.86492	2.27031

27	1	0	5.71339	-2.60670	0.17596
28	1	0	4.86686	-1.76510	-2.00832
29	1	0	2.94628	-0.22038	-2.10033
30	1	0	-2.88729	0.29479	1.69356
31	1	0	-5.07742	-0.85998	1.80648
32	1	0	-5.64009	-2.62699	0.15689
33	1	0	-3.98488	-3.25478	-1.58340
34	1	0	-1.77088	-2.12594	-1.65732

Free Energy and Geometry for Intermediate *Z,E*-9d

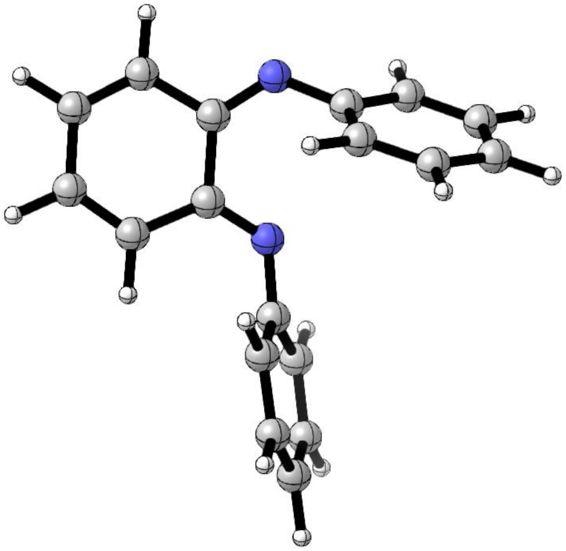

Sum of electronic and thermal free energies: -802.778645 a.u.

Number of imaginary frequencies: 0

Center Number	Atomic Number	Atomic Type	Coordinates (Ångstroms) X	Y	Z
1	6	0	2.16931	2.83951	-0.30865
2	6	0	1.64039	1.47629	-0.25500
3	6	0	0.14787	1.32661	-0.10695
4	6	0	-0.58979	2.49790	0.37118
5	6	0	0.00709	3.70504	0.41255
6	6	0	1.40200	3.88827	0.03721
7	7	0	-0.37361	0.22416	-0.50851
8	7	0	2.48627	0.51289	-0.30770
9	6	0	-1.71974	-0.10325	-0.31055
10	6	0	2.18556	-0.83642	-0.05872
11	6	0	2.48382	-1.79363	-1.03054
12	6	0	2.24631	-3.14057	-0.77523
13	6	0	1.75222	-3.55066	0.46044
14	6	0	1.49928	-2.59712	1.44685
15	6	0	1.71396	-1.24893	1.19447
16	6	0	-2.28134	-0.13005	0.97330
17	6	0	-3.59865	-0.54024	1.14939
18	6	0	-4.37218	-0.91438	0.05283
19	6	0	-3.81007	-0.89877	-1.22348
20	6	0	-2.48628	-0.51793	-1.40589
21	1	0	3.21787	2.94091	-0.56335
22	1	0	-1.64541	2.39142	0.58716
23	1	0	-0.56800	4.57941	0.69718
24	1	0	1.81471	4.89048	0.04942

25	1	0	2.88053	-1.46685	-1.98508
26	1	0	2.45966	-3.87441	-1.54475
27	1	0	1.57861	-4.60184	0.65929
28	1	0	1.13363	-2.90667	2.41995
29	1	0	1.52018	-0.50255	1.95867
30	1	0	-1.66734	0.14819	1.82431
31	1	0	-4.02067	-0.56913	2.14787
32	1	0	-5.39982	-1.22917	0.19245
33	1	0	-4.40206	-1.20078	-2.08023
34	1	0	-2.02921	-0.52841	-2.38879

Free Energy and Geometry for Transition State TS6

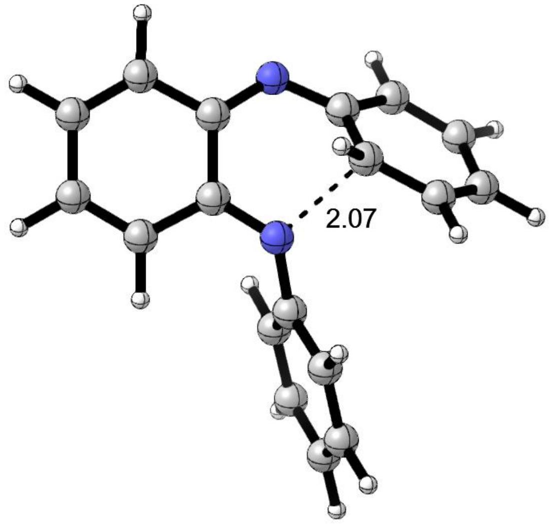

Sum of electronic and thermal free energies: -802.753093 a.u.

Number of imaginary frequencies: 1

Center Number	Atomic Number	Atomic Type	Coordinates (Ångstroms) X	Y	Z
1	6	0	3.63135	-0.29498	-0.01440
2	6	0	2.30163	0.22042	-0.16566
3	6	0	1.20629	-0.73557	-0.34677
4	6	0	1.49715	-2.10604	-0.02420
5	6	0	2.78358	-2.54300	0.11038
6	6	0	3.87748	-1.63034	0.08422
7	7	0	2.08031	1.47615	0.24803
8	6	0	0.87890	2.04176	0.05674
9	6	0	0.38744	2.96911	1.02249
10	7	0	-0.00217	-0.36757	-0.76543
11	6	0	0.05823	1.67682	-1.05570
12	6	0	-1.26292	2.20296	-1.13107
13	6	0	-1.73409	3.01671	-0.13571
14	6	0	-0.90105	3.41991	0.94817
15	1	0	4.42787	0.43670	0.06148
16	1	0	0.67520	-2.81250	-0.04316
17	1	0	2.97489	-3.60444	0.22368
18	1	0	4.89074	-1.99936	0.18983
19	1	0	1.05036	3.24825	1.83394
20	1	0	0.57524	1.38733	-1.96204
21	1	0	-1.88776	1.95149	-1.98065
22	1	0	-2.74738	3.39988	-0.19154
23	1	0	-1.29520	4.08681	1.70576

24	6	0	-1.16744	-0.98047	-0.28475
25	6	0	-2.21472	-1.21831	-1.18399
26	6	0	-1.34905	-1.26353	1.07657
27	6	0	-3.40573	-1.77669	-0.73579
28	1	0	-2.06606	-0.97271	-2.23008
29	6	0	-2.54625	-1.81252	1.52039
30	1	0	-0.54814	-1.03534	1.77308
31	6	0	-3.57410	-2.07673	0.61592
32	1	0	-4.20719	-1.97314	-1.43896
33	1	0	-2.68226	-2.02659	2.57451
34	1	0	-4.50742	-2.50358	0.96491

Free Energy and Geometry for Intermediate 9e

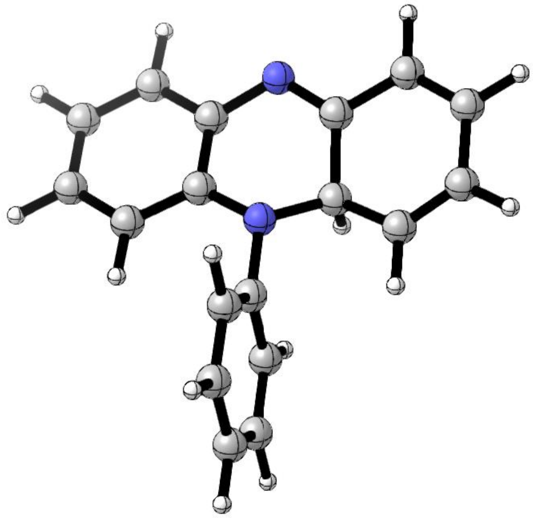

Sum of electronic and thermal free energies: -802.798510 a.u.

Number of imaginary frequencies: 0

Center Number	Atomic Number	Atomic Type	Coordinates (Ångstroms) X	Y	Z
1	6	0	2.57972	2.47714	-0.06359
2	6	0	1.91727	1.24922	0.01182
3	6	0	0.50561	1.21508	0.07383
4	6	0	-0.20053	2.42415	0.08569
5	6	0	0.47994	3.63469	0.00119
6	6	0	1.87198	3.67071	-0.08453
7	7	0	2.67419	0.08289	0.13034
8	6	0	2.07681	-1.04596	-0.02975
9	6	0	0.62750	-1.12454	-0.48362
10	7	0	-0.11165	-0.03704	0.16650
11	6	0	2.81805	-2.28060	0.15574
12	6	0	2.18738	-3.47002	0.16903
13	6	0	0.73742	-3.55265	0.02725
14	6	0	-0.00162	-2.46664	-0.22699
15	1	0	-1.54273	-0.07583	0.09265
16	1	0	-2.28602	-0.05438	1.26886
17	1	0	-3.67788	-0.07635	1.21002
18	1	0	-4.32431	-0.12831	-0.02274
19	1	0	-3.57785	-0.15088	-1.19996
20	1	0	-2.18747	-0.11700	-1.14383
21	1	0	3.66384	2.45897	-0.09196
22	1	0	-1.28200	2.41608	0.15126
23	1	0	-0.08814	4.55834	-0.00265
24	6	0	2.39463	4.61738	-0.15093

25	6	0	0.64182	-0.96758	-1.58553
26	6	0	3.88374	-2.18481	0.33013
27	6	0	2.74703	-4.38363	0.33550
28	1	0	0.25925	-4.51791	0.15055
29	6	0	-1.07918	-2.53949	-0.32171
30	1	0	-1.76034	-0.02217	2.21680
31	6	0	-4.25622	-0.06031	2.12680
32	1	0	-5.40723	-0.15194	-0.06728
33	1	0	-4.07861	-0.18887	-2.16076
34	1	0	-1.59604	-0.12200	-2.05462

Free Energy and Geometry for Intermediate 9a′

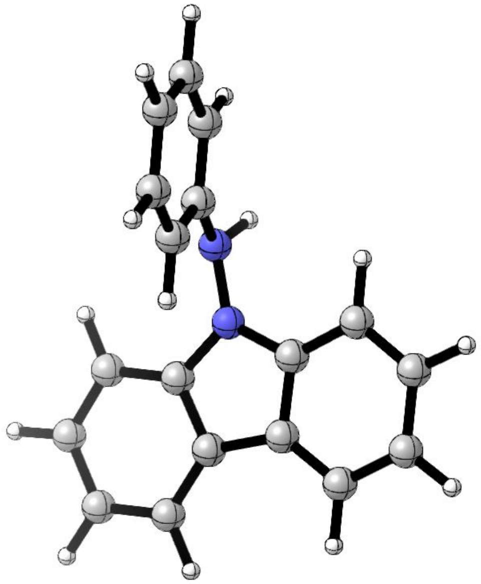

Sum of electronic and thermal free energies: -802.833856 a.u.

Number of imaginary frequencies: 0

Center Number	Atomic Number	Atomic Type	Coordinates (Ångstroms) X	Y	Z
1	6	0	-2.54585	-2.23288	0.47955
2	6	0	-1.91031	-1.04737	0.10225
3	6	0	-0.61298	-1.10821	-0.44895
4	6	0	0.07189	-2.30849	-0.62088
5	6	0	-0.58259	-3.47272	-0.23563
6	6	0	-1.87705	-3.43831	0.30601
7	7	0	-0.20249	0.18569	-0.74955
8	6	0	-1.19764	1.08323	-0.38528
9	6	0	-2.28334	0.35556	0.14539
10	6	0	-3.41631	1.04531	0.58526
11	6	0	-3.43698	2.43095	0.49094
12	6	0	-2.34068	3.13328	-0.03524
13	6	0	-1.20289	2.47204	-0.48118
14	7	0	1.01638	0.55111	-1.26475
15	6	0	2.15287	0.44723	-0.43828
16	6	0	2.03776	0.36907	0.95089
17	6	0	3.18761	0.30928	1.73386
18	6	0	4.45184	0.32506	1.15131
19	6	0	4.55844	0.40225	-0.23669
20	6	0	3.41959	0.46231	-1.03012
21	1	0	-3.54460	-2.21176	0.90267
22	1	0	1.07668	-2.32808	-1.02889
23	1	0	-0.08071	-4.42661	-0.35246

24	1	0	-2.35692	-4.36616	0.59505
25	1	0	-4.26394	0.50711	0.99621
26	1	0	-4.30797	2.98044	0.82889
27	1	0	-2.38224	4.21522	-0.09333
28	1	0	-0.34866	3.00216	-0.88602
29	1	0	1.14548	0.28332	-2.23597
30	1	0	1.05699	0.35644	1.41332
31	1	0	3.08810	0.24802	2.81187
32	1	0	5.34146	0.27674	1.76786
33	1	0	5.53528	0.41402	-0.70736
34	1	0	3.50357	0.52601	-2.11095

Free Energy and Geometry for Intermediate 9e′

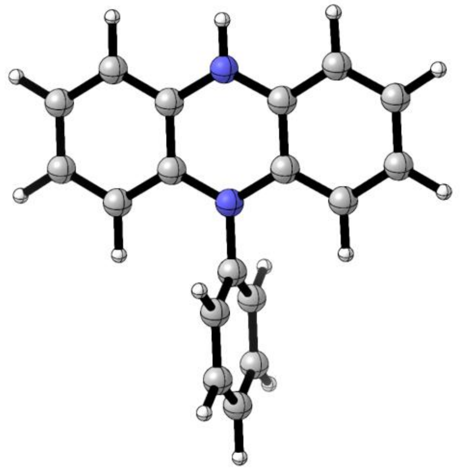

Sum of electronic and thermal free energies: -802.846584 a.u.

Number of imaginary frequencies: 0

Center Number	Atomic Number	Atomic Type	Coordinates (Ångstroms) X	Y	Z
1	6	0	0.10903	2.45254	0.00042
2	6	0	-0.55580	1.23033	0.00018
3	6	0	-1.96569	1.22326	0.00017
4	6	0	-2.66221	2.42460	0.00030
5	6	0	-1.98530	3.64741	0.00049
6	6	0	-0.60063	3.65915	0.00057
7	7	0	0.13133	-0.00000	0.00003
8	6	0	-0.55580	-1.23032	-0.00017
9	6	0	-1.96569	-1.22326	-0.00013
10	7	0	-2.63024	0.00000	0.00006
11	6	0	0.10903	-2.45254	-0.00047
12	6	0	-0.60063	-3.65915	-0.00064
13	6	0	-1.98531	-3.64740	-0.00054
14	6	0	-2.66222	-2.42460	-0.00030
15	1	0	1.56087	-0.00000	0.00002
16	1	0	2.25298	-0.00099	1.20871
17	1	0	3.64557	-0.00100	1.20732
18	1	0	4.34159	-0.00000	-0.00000
19	1	0	3.64555	0.00100	-1.20731
20	1	0	2.25297	0.00098	-1.20869
21	1	0	1.19243	2.46724	0.00051
22	1	0	-3.74782	2.39666	0.00028
23	1	0	-2.54786	4.57354	0.00059
24	6	0	-0.05586	4.59582	0.00075
25	6	0	-3.64073	0.00000	0.00005
26	6	0	1.19242	-2.46724	-0.00057

27	6	0	-0.05587	-4.59582	-0.00087
28	1	0	-2.54786	-4.57354	-0.00067
29	6	0	-3.74782	-2.39665	-0.00025
30	1	0	1.69177	-0.00174	2.13711
31	6	0	4.18613	-0.00177	2.14698
32	1	0	5.42577	-0.00000	-0.00001
33	1	0	4.18610	0.00177	-2.14698
34	1	0	1.69175	0.00173	-2.13708

Free Energy and Geometry for Transition State TS7

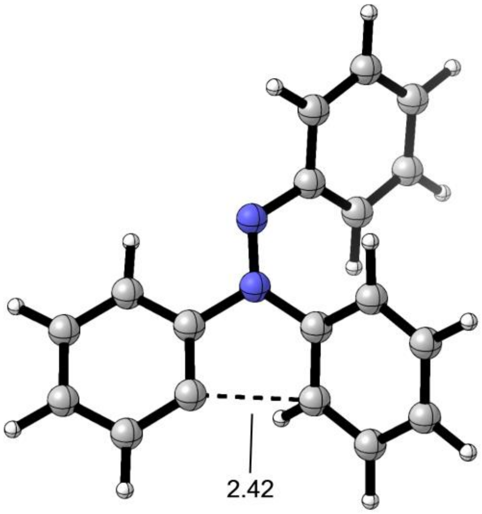

Sum of electronic and thermal free energies: -802.665281 a.u.

Number of imaginary frequencies: 1

Center Number	Atomic Number	Atomic Type	Coordinates (Ångstroms) X	Y	Z
1	6	0	-4.07505	-0.29823	0.06875
2	6	0	-2.76919	0.15576	-0.14246
3	6	0	-1.75798	-0.80122	-0.09641
4	6	0	-2.00172	-2.17796	0.04012
5	6	0	-3.31456	-2.60314	0.09911
6	6	0	-4.35732		-1.66235
7	7	0	-0.39517	-0.35932	-0.17674
8	7	0	0.51571	-1.22930	-0.45595
9	6	0	-0.22784	1.03022	-0.06514
10	6	0	1.85147	-1.00001	-0.10656
11	6	0	2.27847	-0.26208	1.01140
12	6	0	3.62857	-0.19923	1.32144
13	6	0	4.57646	-0.84352	0.52294
14	6	0	4.15558	-1.58251	-0.57967
15	6	0	2.80247	-1.67576	-0.88334
16	6	0	0.66244	1.69332	-0.92826
17	6	0	0.62801	3.06699	-1.00385
18	6	0	-0.28826	3.79908	-0.21815
19	6	0	-1.15874	3.15303	0.63006
20	6	0	-1.15609	1.74380	0.71552
21	1	0	-4.90048	0.40776	0.12346
22	1	0	-1.16915	-2.86550	0.14589
23	1	0	-3.53802	-3.65975	0.19236
24	1	0	-5.38748	-2.00554	0.12299
25	1	0	1.55636	0.24231	1.64271

26	1	0	3.94677	0.35839	2.19515
27	1	0	5.63007	-0.77673	0.76760
28	1	0	4.88062	-2.09837	-1.19882
29	1	0	2.45297	-2.26655	-1.72258
30	1	0	1.32678	1.11917	-1.56263
31	1	0	1.28949	3.58439	-1.68810
32	1	0	-0.29692	4.88155	-0.27971
33	1	0	-1.84428	3.71816	1.24958
34	1	0	-1.65076	1.26397	1.54728

Free Energy and Geometry for Transition State TS8

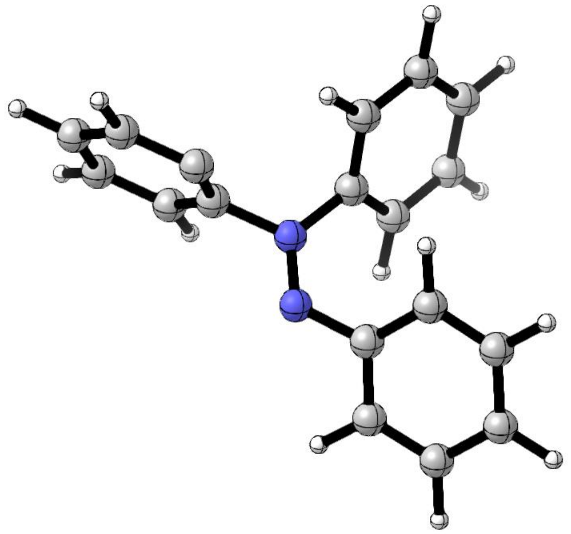

Sum of electronic and thermal free energies: -802.662518 a.u.

Number of imaginary frequencies: 1

Center Number	Atomic Number	Atomic Type	Coordinates (Ångstroms) X	Y	Z
1	6	0	-3.82107	-1.07393	1.30501
2	6	0	-2.43002	-0.80348	1.29212
3	6	0	-1.96708	-0.67475	0.00201
4	6	0	-2.65259	-0.75162	-1.20721
5	6	0	-4.01048	-1.03259	-1.12101
6	6	0	-4.58689	-1.19530	0.14431
7	7	0	-0.50419	-0.36754	-0.07202
8	7	0	0.27579	-1.33483	-0.15008
9	6	0	-0.13075	1.03441	-0.10171
10	6	0	1.66589	-1.20001	0.03587
11	6	0	2.44861	-2.08076	-0.71603
12	6	0	3.83033	-2.05640	-0.57703
13	6	0	4.41676	-1.20746	0.35915
14	6	0	3.62310	-0.38034	1.15660
15	6	0	2.24620	-0.35802	0.99420
16	6	0	-0.64150	1.91179	0.84796
17	6	0	-0.30022	3.25735	0.75394
18	6	0	0.51539	3.70842	-0.28161
19	6	0	0.99573	2.81486	-1.23811
20	6	0	0.67793	1.46570	-1.15162
21	1	0	-4.33582	-1.20061	2.25684
22	1	0	-2.15638	-0.60961	-2.16342
23	1	0	-4.61115	-1.12335	-2.01907
24	1	0	-5.64913	-1.41384	0.21714

25	1	0	1.95672	-2.76185	-1.40067
26	1	0	4.44520	-2.71697	-1.17618
27	1	0	5.49294	-1.20523	0.48692
28	1	0	4.08155	0.24859	1.91022
29	1	0	1.62650	0.26828	1.62418
30	1	0	-1.27428	1.52494	1.63815
31	1	0	-0.67820	3.95440	1.49218
32	1	0	0.77094	4.75947	-0.34956
33	1	0	1.61400	3.16801	-2.05468
34	1	0	1.03987	0.75723	-1.88833

Free Energy and Geometry for Intermediate *cis*-14

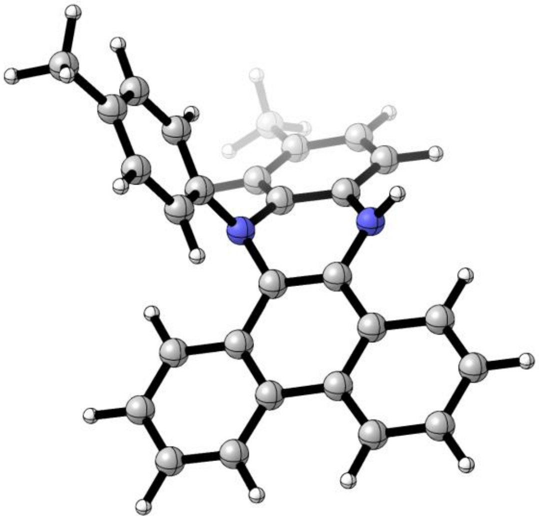

Sum of electronic and thermal free energies: -1188.233083 a.u.

Number of imaginary frequencies: 0

Center Number	Atomic Number	Atomic Type	Coordinates (Ångstroms) X	Y	Z
1	6	0	-2.60962	-1.63203	1.23228
2	6	0	-1.54648	-1.28363	0.40360
3	6	0	-1.03400	-2.20777	-0.50862
4	6	0	-1.65979	-3.44588	-0.65126
5	6	0	-2.76760	-3.75513	0.12898
6	6	0	-3.24891	-2.86444	1.09508
7	7	0	-0.92405	0.00426	0.44488
8	6	0	0.49953	-0.07833	0.31842
9	6	0	1.01277	-0.95779	-0.58762
10	7	0	0.14899	-1.87810	-1.19711
11	6	0	1.35541	0.74194	1.13284
12	6	0	2.75215	0.73888	0.89519
13	6	0	3.29693	-0.13456	-0.13719
14	6	0	2.43336	-1.01062	-0.84147
15	6	0	-1.57829	1.09085	-0.20181
16	6	0	-0.85021	2.18281	-0.69337
17	6	0	-1.49891	3.25637	-1.29709
18	6	0	-2.88451	3.29253	-1.44530
19	6	0	-3.60032	2.19188	-0.97080
20	6	0	-2.97141	1.11323	-0.35983
21	1	0	-2.95218	-0.91370	1.97140
22	1	0	-1.25884	-4.16624	-1.35665
23	1	0	-3.24963	-4.72091	0.01152
24	1	0	0.22961	2.19871	-0.61952

25	1	0	-0.90174	4.08420	-1.66872
26	1	0	-4.68085	2.17173	-1.08237
27	1	0	-3.57303	0.28136	-0.01769
28	6	0	2.96619	-1.91013	-1.78833
29	6	0	4.31487	-1.92295	-2.07225
30	1	0	4.71156	-2.61857	-2.80282
31	6	0	4.67187	-0.16991	-0.45339
32	6	0	5.17267	-1.03624	-1.40334
33	1	0	6.23398	-1.03739	-1.62424
34	6	0	0.81288	1.57692	2.13303
35	6	0	3.56032	1.58042	1.68896
36	6	0	3.01577	2.38789	2.66632
37	1	0	3.66192	3.02261	3.26203
38	6	0	1.62950	2.38579	2.89261
39	1	0	1.20355	3.01884	3.66277
40	1	0	5.35962	0.49385	0.05523
41	1	0	2.30293	-2.60810	-2.28601
42	1	0	4.63287	1.59693	1.54144
43	1	0	-0.25956	1.56327	2.29123
44	1	0	0.17046	-1.97013	-2.20520
45	6	0	-4.43816	-3.21834	1.94808
46	1	0	-5.37633	-3.00987	1.42410
47	1	0	-4.43818	-4.27983	2.20641
48	1	0	-4.44459	-2.64307	2.87618
49	6	0	-3.58106	4.47170	-2.07003
50	1	0	-4.45333	4.15772	-2.64865
51	1	0	-3.93129	5.17640	-1.30894
52	1	0	-2.91133	5.01816	-2.73804

Free Energy and Geometry for Intermediate *trans*-14

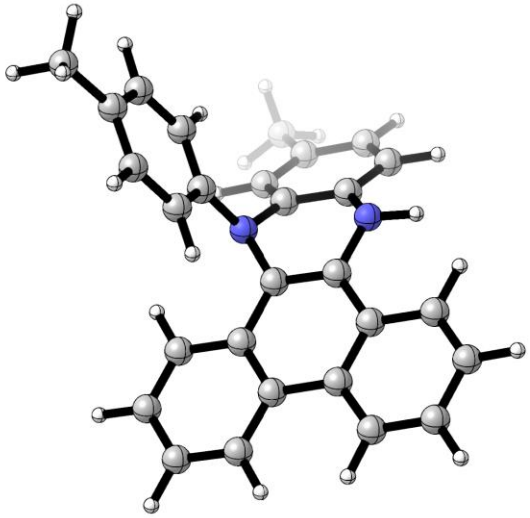

Sum of electronic and thermal free energies: -1188.236162 a.u.

Number of imaginary frequencies: 0

Center Number	Atomic Number	Atomic Type	Coordinates (Ångstroms) X	Y	Z
1	6	0	-2.53247	-1.69694	1.24464
2	6	0	-1.50502	-1.29771	0.39683
3	6	0	-0.99007	-2.18750	-0.54972
4	6	0	-1.56156	-3.45199	-0.68417
5	6	0	-2.62406	-3.81832	0.13417
6	6	0	-3.12067	-2.95634	1.11751
7	7	0	-0.92879	0.00971	0.43209
8	6	0	0.49429	-0.04000	0.29144
9	6	0	1.01332	-0.89491	-0.63919
10	7	0	0.13445	-1.77212	-1.27805
11	6	0	1.34367	0.75629	1.13132
12	6	0	2.74482	0.73731	0.92596
13	6	0	3.29366	-0.09517	-0.13703
14	6	0	2.43270	-0.91704	-0.90961
15	6	0	-1.62245	1.07803	-0.20112
16	6	0	-3.01133	1.03717	-0.38553
17	6	0	-3.67934	2.10200	-0.97931
18	6	0	-3.00745	3.24766	-1.40943
19	6	0	-1.62460	3.27385	-1.23357
20	6	0	-0.93708	2.21634	-0.64527
21	1	0	-2.88675	-0.99681	1.99578
22	1	0	-1.16456	-4.14778	-1.41686
23	1	0	-3.06382	-4.80473	0.02275
24	1	0	0.54499	-2.48018	-1.87250
25	1	0	-3.57660	0.16665	-0.07829

26	1	0	-4.75526	2.03422	-1.11448
27	1	0	-1.06145	4.13950	-1.57056
28	1	0	0.13920	2.27928	-0.54633
29	6	0	2.97495	-1.71341	-1.94139
30	6	0	4.32710	-1.71388	-2.20765
31	1	0	4.72257	-2.32697	-3.00908
32	6	0	4.67324	-0.12131	-0.43349
33	6	0	5.18469	-0.91108	-1.44157
34	1	0	6.24944	-0.90487	-1.64446
35	6	0	0.79268	1.56497	2.14937
36	6	0	3.54944	1.53252	1.76967
37	6	0	2.99721	2.31299	2.76438
38	1	0	3.64041	2.91268	3.39825
39	1	0	1.60575	2.32936	2.95687
40	1	0	1.17270	2.94192	3.73961
41	1	0	5.35621	0.49624	0.13563
42	1	0	2.32882	-2.32374	-2.56265
43	1	0	4.62529	1.53563	1.64789
44	1	0	-0.28308	1.56452	2.28355
45	6	0	-4.27416	-3.36331	1.99576
46	1	0	-5.23379	-3.12369	1.52680
47	1	0	-4.26464	-4.43852	2.18852
48	1	0	-4.24096	-2.84627	2.95720
49	6	0	-3.74718	4.40885	-2.01825
50	1	0	-4.58838	4.06916	-2.62758
51	1	0	-4.15053	5.07330	-1.24727
52	1	0	-3.08984	5.00673	-2.65374

VII. X-ray Data for 3a and 11b

Data for 3a (CCDC Deposition Number 2251646)

Data collection

A crystal (approximate dimensions 0.080 x 0.040 x 0.030 mm^3) was placed onto the tip of a 50μm MiTeGen Dual Thickness Microloop and mounted on a Bruker PHOTON-III CPAD diffractometer for a data collection at 130(2) K.[17] A preliminary set of cell constants was calculated from reflections harvested from three sets of frames. These initial sets of frames were oriented such that orthogonal wedges of reciprocal space were surveyed. This produced an initial orientation matrix determined from 993 reflections. The data collection was carried out using MoKα radiation (parabolic mirrors) with a frame time of 300 seconds and a detector distance of 10.0 cm. A strategy program was used to assure complete coverage of all unique data to a resolution of 0.95 Å. All major sections of frames were collected with 1.20º steps in ω or ϕ at different detector positions in 2θ. The intensity data were corrected for absorption and decay (SADABS).[18] Final cell constants were calculated from 2981 strong reflections from the actual data collection after integration (SAINT).[19] Please refer to Table S1 for additional crystal and refinement information.

Structure solution and refinement

The structure was solved using SHELXT-2018/2 (Sheldrick, 2018)[20] and refined using SHELXL-2018/3 (Sheldrick, 2018).[20] The space group P-1 was determined based on systematic absences and intensity statistics. A direct-methods solution was calculated which provided most non-hydrogen atoms from the E-map. Full-matrix least squares / difference Fourier cycles were performed which located the remaining non-hydrogen atoms. All non-hydrogen atoms were refined with anisotropic displacement parameters. All hydrogen atoms were placed in ideal positions and refined as riding atoms with relative isotropic displacement parameters. The final full matrix least squares refinement converged to $R1$ = 0.0931 and $wR2$ = 0.2098 (F^2, obs. data).

Data collection and structure solution were conducted at the X-Ray Crystallographic Laboratory, 192 Kolthoff Hall, Department of Chemistry, University of Minnesota. All calculations were performed using Pentium computers using the current SHELXTL suite of programs.

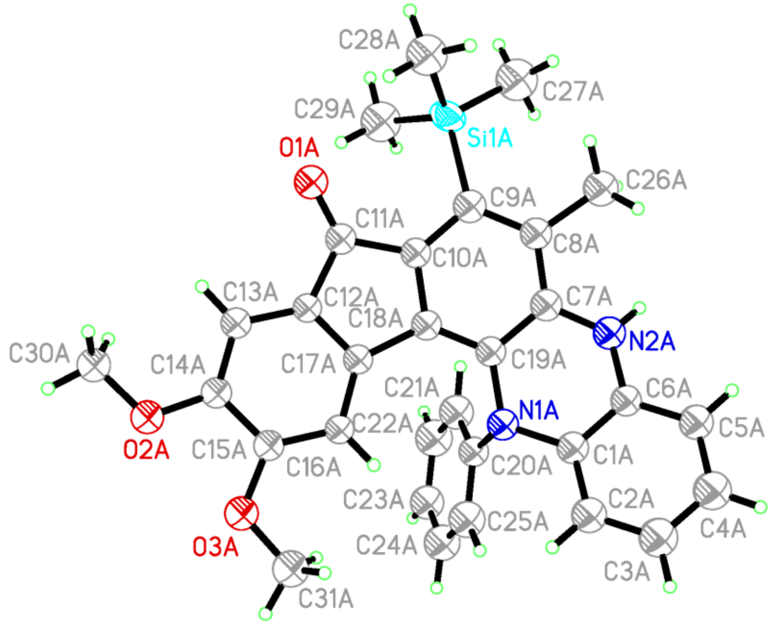

Table S1. Crystal data and structure refinement for 23003z.

Identification code	23003z	
Empirical formula	$C_{126} H_{123} N_9 O_{12} Si_4$	
Formula weight	2067.69	
Temperature	130(2) K	
Wavelength	0.71073 Å	
Crystal system	triclinic	
Space group	P-1	
Unit cell dimensions	*a* = 11.824(4) Å	α = 105.035(7)°
	b = 19.935(6) Å	β = 102.319(7)°
	c = 24.553(8) Å	γ = 97.010(8)°
Volume	5363(3) $Å^3$	
Z	2	
Density (calculated)	1.280 Mg/m^3	
Absorption coefficient	0.124 mm^{-1}	
F(000)	2188	
Crystal color, morphology	Red, Block	
Crystal size	0.080 x 0.040 x 0.030 mm^3	
Theta range for data collection	1.912 to 21.994°	
Index ranges	$-12 \le h \le 12$, $-20 \le k \le 18$, $-25 \le l \le 25$	

Reflections collected	40278
Independent reflections	13075 [*R*(int) = 0.0964]
Observed reflections	7246
Completeness to theta = 21.994°	99.4%
Absorption correction	Multi-scan
Max. and min. transmission	0.7447 and 0.6139
Refinement method	Full-matrix least-squares on F^2
Data / restraints / parameters	13075 / 0 / 650
Goodness-of-fit on F^2	1.023
Final *R* indices [*I*>2sigma(*I*)]	*R*1 = 0.0931, *wR*2 = 0.2098
R indices (all data)	*R*1 = 0.1737, *wR*2 = 0.2564
Largest diff. peak and hole	0.490 and -0.492 e.Å^{-3}

Data for 11b (CCDC Deposition Number 2251645)

Data collection

A crystal (approximate dimensions 0.080 x 0.040 x 0.030 mm^3) was placed onto the tip of a 50μm MiTeGen Dual Thickness Microloop and mounted on a Bruker PHOTON-III CPAD diffractometer for a data collection at 130(2) K.[21] A preliminary set of cell constants was calculated from reflections harvested from three sets of frames. These initial sets of frames were oriented such that orthogonal wedges of reciprocal space were surveyed. This produced an initial orientation matrix determined from 993 reflections. The data collection was carried out using MoKα radiation (parabolic mirrors) with a frame time of 300 seconds and a detector distance of 10.0 cm. A strategy program was used to assure complete coverage of all unique data to a resolution of 0.95 Å. All major sections of frames were collected with 1.20º steps in ω or ϕ at different detector positions in 2θ. The intensity data were corrected for absorption and decay (SADABS).[22] Final cell constants were calculated from 2981 strong reflections from the actual data collection after integration (SAINT).[23] Please refer to Table S2 for additional crystal and refinement information.

Structure solution and refinement

The structure was solved using SHELXS-97 (Sheldrick 2008)[24] and refined using SHELXL-2019/1 (Sheldrick, 2019).[25] The space group P-1 was determined based on systematic absences and intensity statistics. A direct-methods solution was calculated which provided most non-hydrogen atoms from the E-map. Full-matrix least squares / difference Fourier cycles were performed which located the remaining non-hydrogen atoms. All non-hydrogen atoms were refined with anisotropic displacement parameters. All hydrogen atoms bound to carbon were placed in ideal positions and refined as riding atoms with relative isotropic displacement parameters. Hydrogen atom H2 was placed via Fourier peak and refined with isotropic displacement parameters relative to N2. The final full matrix least squares refinement converged to $R1 = 0.0450$ and $wR2 = 0.1234$ (F^2, all data).

Data collection and structure solution were conducted at the X-Ray Crystallographic Laboratory, K193 Kolthoff Hall, Department of Chemistry, University of Minnesota. All calculations were performed using Pentium computers using the current SHELXTL suite of programs.

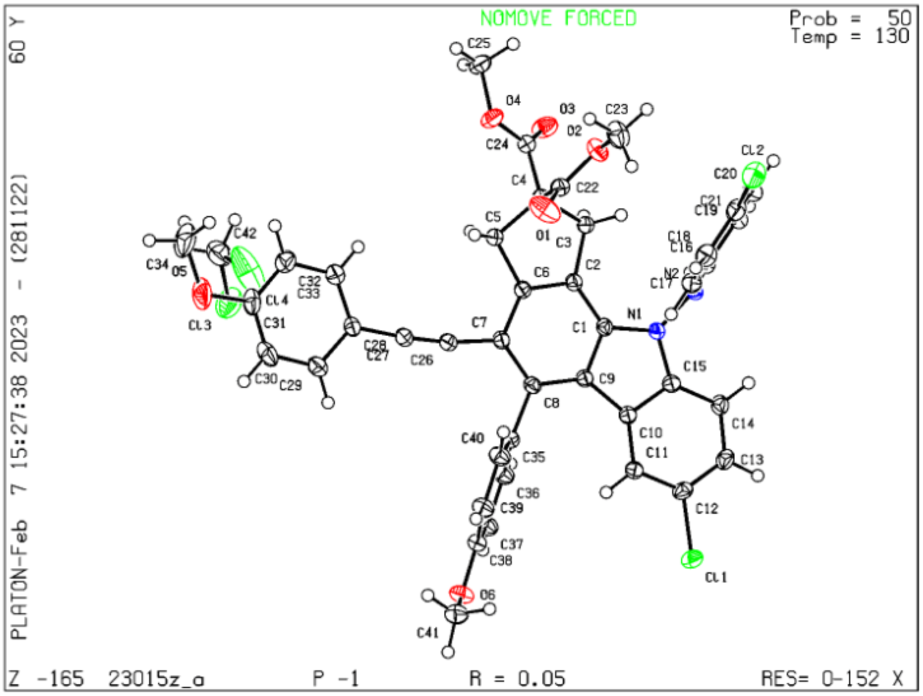

Table 1. Crystal data and structure refinement for 23015.

Identification code	23015z_a	
Empirical formula	$C_{42} H_{34} Cl_4 N_2 O_6$	
Formula weight	804.51	
Temperature	130(2) K	
Wavelength	0.71073 Å	
Crystal system	Triclinic	
Space group	P-1	
Unit cell dimensions	*a* = 11.5264(8) Å	α = 88.001(2)°
	b = 12.3665(9) Å	β = 88.826(2)°
	c = 14.7242(8) Å	γ = 64.702(2)°
Volume	1896.3(2) Å^3	
Z	2	
Density (calculated)	1.409 Mg/m^3	
Absorption coefficient	0.364 mm^{-1}	
F(000)	832	
Crystal color, morphology	colorless, block	
Crystal size	0.300 x 0.160 x 0.160 mm^3	
Theta range for data collection	1.954 to 29.599°	

Index ranges	$-16 \le h \le 16$, $-17 \le k \le 17$, $-14 \le l \le 20$
Reflections collected	44925
Independent reflections	10652 [*R*(int) = 0.0381]
Observed reflections	8797
Completeness to theta = 25.242°	99.9%
Absorption correction	multi-scan
Max. and min. transmission	0.7454 and 0.7011
Refinement method	Full-matrix least-squares on F^2
Data / restraints / parameters	10652 / 0 / 494
Goodness-of-fit on F^2	1.033
Final *R* indices [*I*>2sigma(*I*)]	*R*1 = 0.0450, *wR*2 = 0.1141
R indices (all data)	*R*1 = 0.0570, *wR*2 = 0.1234
Extinction coefficient	n/a
Largest diff. peak and hole	0.755 and -0.711 e.Å^{-3}

VIII. Supporting Information References

[1] T. R. Hoye, P. R. Hanson and J. R. Vyvyan, A Practical Guide to First-Order Multiplet Analysis in ^{1}H NMR Spectroscopy, *J. Org. Chem.* 1994, **59**, 4096–4103.

[2] T. R. Hoye and H. Zhao, A Method for Easily Determining Coupling Constant Values: An Addendum to "A Practical Guide to First-Order Multiplet Analysis in ^{1}H NMR Spectroscopy", *J. Org. Chem.* 2002, **67**, 4014–4016.

[3] D. S. Sneddon and T. R. Hoye, Arylhydrazine Trapping of Benzynes: Mechanistic Insights and a Route to Azoarenes, *Org. Lett.* 2021, **23**, 3432–3436.

[4] S. Arora, J. Zhang, V. Pogula and T. R. Hoye, Reactions of thermally generated benzynes with six-membered N-heteroaromatics: pathway and product diversity, *Chem. Sci.* 2019, **10**, 9069–9076.

[5] F. Xu, X. Xiao and T. R. Hoye, Reactions of HDDA-Derived Benzynes with Perylenes: Rapid Construction of Polycyclic Aromatic Compounds, *Org. Lett.* 2016, **18**, 5636–5639.

[6] F. Xu, X. Xiao, and T. R. Hoye, Photochemical Hexadehydro-Diels–Alder Reaction, *J. Am. Chem. Soc.* 2017, **139**, 8400–8403.

[7] C. Zhang and N. Jiao, Copper-catalyzed aerobic oxidative dehydrogenative coupling of anilines leading to aromatic azo compounds using dioxygen as an oxidant, *Angew. Chem. Int. Ed.* 2010, **49**, 6174–6177.

[8] D. J. Tindall, C. Werlé, R. Goddard, P. Philipps, C. Farès and A. Fürstner, Structure and Reactivity of Half-Sandwich Rh(+3) and Ir(+3) Carbene Complexes. Catalytic Metathesis of Azobenzene Derivatives, *J. Am. Chem. Soc.* 2018, **140**, 1884–1893.

[9] W. Zhang, J. Bu, L. Wang, P. Li and H. Li. Sunlight-mediated [3+2] cycloaddition of azobenzenes with arynes: an approach towards the carbazole skeleton, *Org. Chem. Front.*, 2021, **8**, 5045–5051.

[10] (*a*) A. Dall'Anese, V. Rosar, L. Cusin, T. Montini, G. Balducci, I. D'Auria, C. Pellecchia, P. Fornasiero, F. Felluga and B. Milani, Palladium-Catalyzed Ethylene/Methyl Acrylate Copolymerization: Moving from the Acenaphthene to the Phenanthrene Skeleton of α-Diimine Ligands, *Organometallics* 2019, **38**, 3498–3511. (*b*) B. Gao, D. Zhao, X. Li, Y. Cui, R. Duan and X. Pang, Magnesium complexes bearing *N*,*N*-bidentate phenanthrene derivatives for the stereoselective ring-opening polymerization of *rac*-lactides, *RSC Adv.* 2015, **5**, 440–447.

[11] K. L. Bamford, L. E. Longobardi, L. Liu, S. Grimme and D. W. Stephan, FLP reduction and hydroboration of phenanthrene *o*-iminoquinones and α-diimines, Dalton Trans. 2017, **46**, 5308–5319.

[12] L. Chen, Y. Chen, W. Zhou, J. Li, Y. Zhang and Y. Liu, Mitochondrion-targeting chemiluminescent ternary supramolecular assembly for *in situ* photodynamic therapy, *Chem. Commun.* 2020, **56**, 8857–8860.

[13] R. van Belzen, R. A. Klein, W. J. J. Smeets, A. L. Spek, R. Benedix and C. J. Elsevier, Synthesis and characterization of 9,l0-*bis*(arylimino)-9,l0-dihydrophenanthrenes, the structure of (*Z*,*Z*)-9,10-*bis*(phenylimino)-9,10-dihydrophenanthrene and $PdCl_2$-[(*E*,*E*)-9,10-*bis*(phenylimino)-9,l0-dihydrophenanthrene] in the solid state and in solution, *Recl. Trav. Chim. Pays-Bas.* 1996, **115**, 275–285.

[14] M. J. Frisch, G. W. Trucks, H. B. Schlegel, G. E. Scuseria, M. A. Robb, J. R. Cheeseman, G. Scalmani, V. Barone, G. A. Petersson, H. Nakatsuji, X. Li, M. Caricato, A. V. Marenich, J. Bloino, B. G. Janesko, R. Gomperts, B. Mennucci, H. P. Hratchian, J. V. Ortiz, A. F. Izmaylov, J. L. Sonnenberg, D. Williams-Young, F. Ding, F. Lipparini, F. Egidi, J. Goings, B. Peng, A. Petrone, T. Henderson, D. Ranasinghe, V. G. Zakrzewski, J. Gao, N. Rega, G. Zheng, W. Liang, M. Hada, M. Ehara, K. Toyota, R. Fukuda, J. Hasegawa, M. Ishida, T. Nakajima, Y. Honda, O. Kitao, H. Nakai, T. Vreven, K. Throssell, J. A. Montgomery, Jr., J. E. Peralta, F. Ogliaro, M. J. Bearpark, J. J. Heyd, E. N. Brothers, K. N. Kudin, V. N. Staroverov, T. A. Keith, R. Kobayashi, J. Normand, K. Raghavachari, A. P. Rendell, J. C. Burant, S. S. Iyengar, J. Tomasi, M. Cossi, J. M. Millam, M. Klene, C. Adamo, R. Cammi, J. W. Ochterski, R. L. Martin, K. Morokuma, O. Farkas, J. B. Foresman and D. J. Fox, *Gaussian 16*, Revision C.01, Gaussian, Inc., Wallingford CT, 2016.

[15] *Schrödinger Release 2020-4*, Maestro, Schrödinger, LLC, New York, NY, 2020.

[16] (*a*) P. H. Willoughby, M. J. Jansma and T. R. Hoye, A guide to small-molecule structure assignment through computation of (^{1}H and ^{13}C) NMR chemical shifts, *Nat. Protoc.* 2014, **9**, 643–660. (*b*) See the following Addendum for fully reliable versions of the Python scripts: P. H. Willoughby, M. J. Jansma and T. R. Hoye, Addendum: A guide to small-molecule structure assignment through computation of (^{1}H and ^{13}C) NMR chemical shifts, *Nat. Protoc.* 2020, **15**, 2277–2277.

[17] APEX4, Bruker Analytical X-ray Systems, Madison, WI (2014).

[18] SADABS, Bruker Analytical X-ray Systems, Madison, WI (2014).

[19] SAINT Bruker Analytical X-ray Systems, Madison, WI (2014).

[20] SHELXTL 2018, Bruker Analytical X-Ray Systems, Madison, WI (2013); G. M. Sheldrick, *Acta Cryst.* **A64**, 112-122 (2008).

[21] APEX3, Bruker Analytical X-ray Systems, Madison, WI (2016).

[22] SADABS, Bruker Analytical X-ray Systems, Madison, WI (2016).

[23] SAINT Bruker Analytical X-ray Systems, Madison, WI (2016).

[24] SHELXTL 2018/2, Bruker Analytical X-Ray Systems, Madison, WI (2016); G. M. Sheldrick, *Acta Cryst.* **A71**, 3-8 (2015).

[25] SHELXL 2016/6; G. M. Sheldrick, *Acta Cryst.* **C71**, 3-8 (2015).

IX. Copies of 1D and 2D NMR Spectra

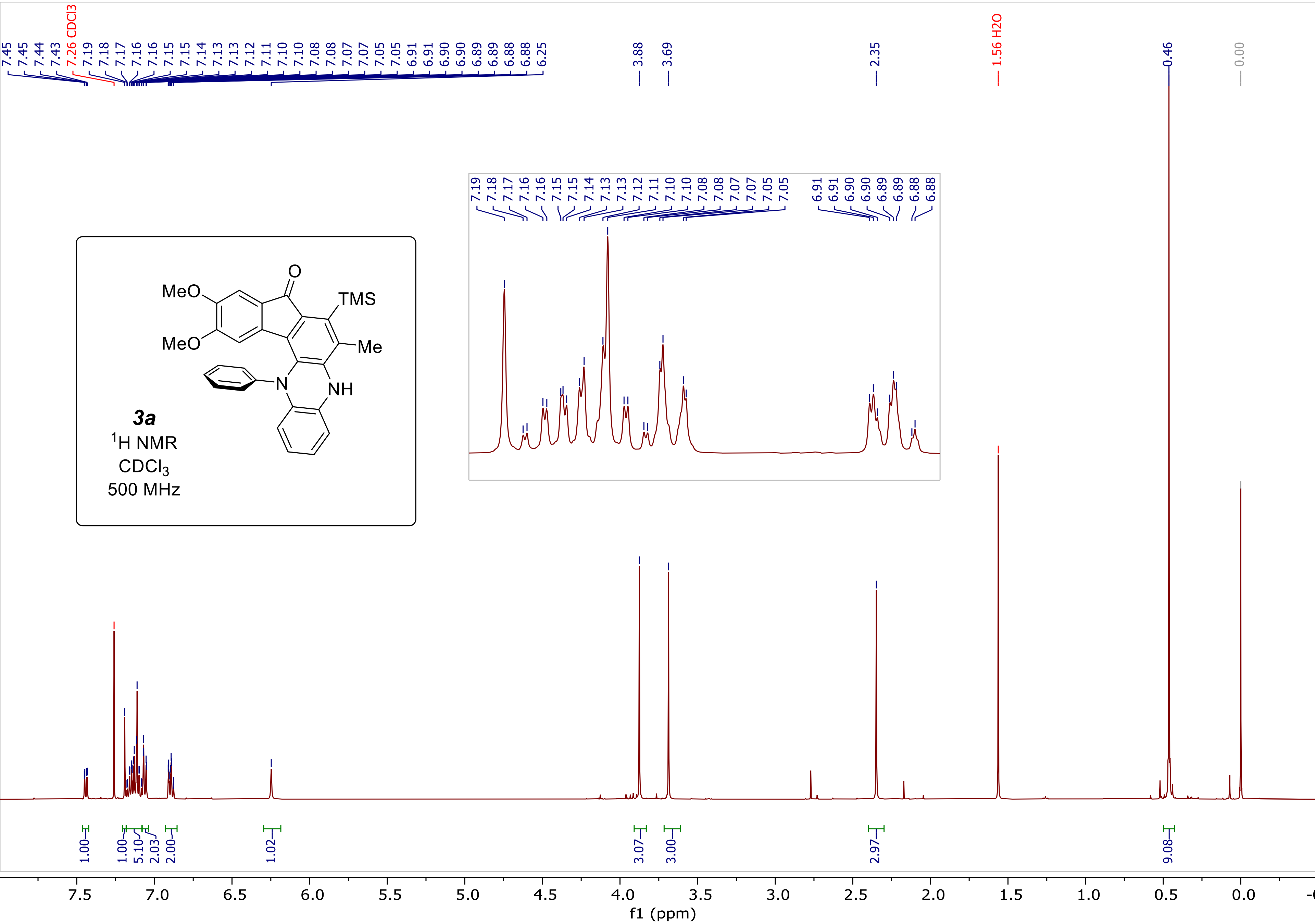
7.45
7.45
7.44
7.43
7.26 CDCl3
7.19
7.18
7.17
7.16
7.16
7.15
7.15
7.14
7.13
7.13
7.12
7.11
7.10
7.10
7.08
7.08
7.07
7.07
7.05
7.05
6.91
6.91
6.90
6.90
6.89
6.89
6.88
6.88
6.25
3.88
3.69
2.35
1.56 H2O
0.46
0.00
MeO
MeO
O
TMS
Me
N
NH
3a
1H NMR
CDCl3
500 MHz
1.00
1.00
5.10
2.03
2.00
1.02
3.07
3.00
2.97
9.08
7.5
7.0
6.5
6.0
5.5
5.0
4.5
4.0
3.5
3.0
2.5
2.0
1.5
1.0
0.5
0.0
f1 (ppm)

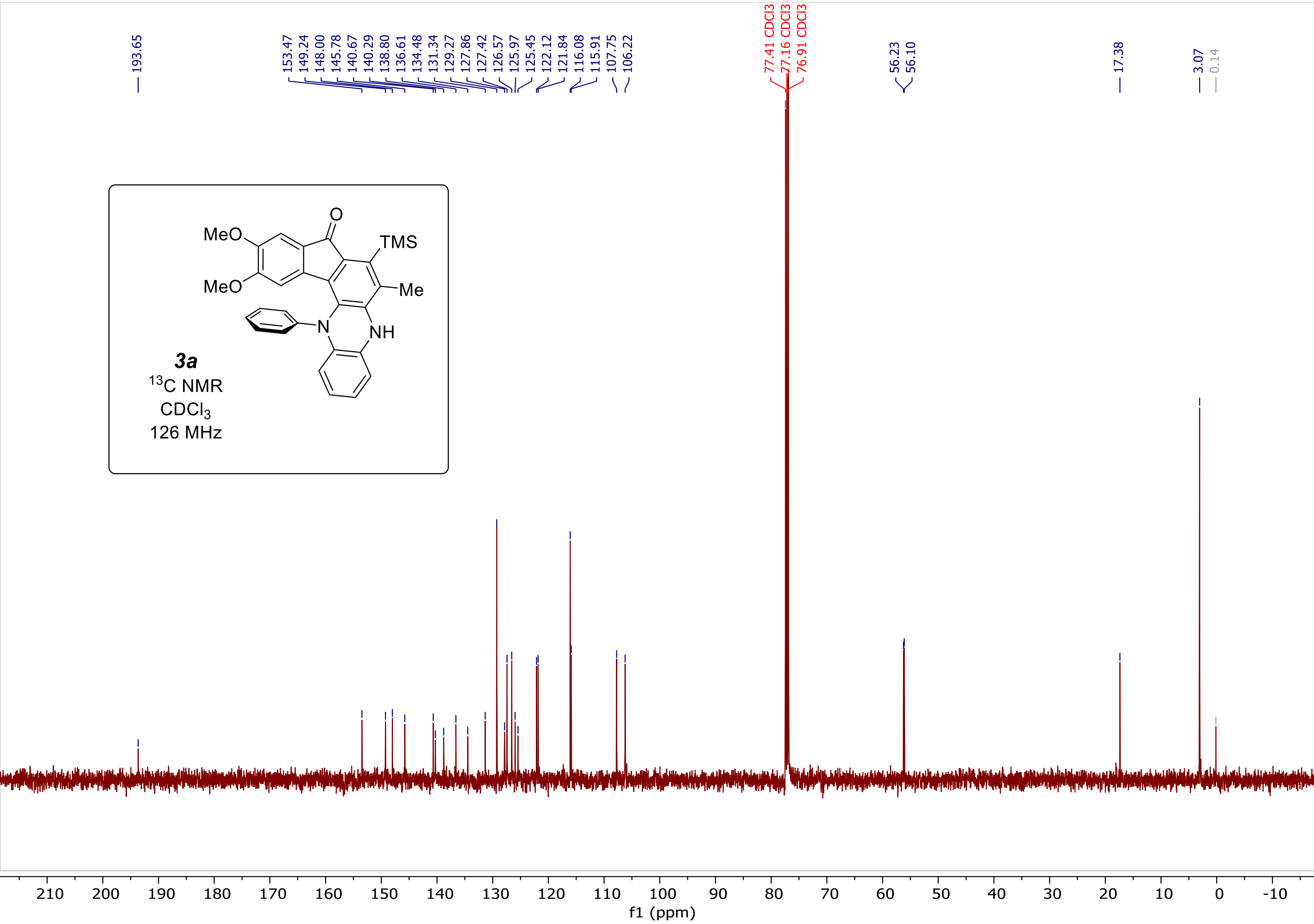
193.65
153.47
149.24
148.00
145.78
140.67
140.29
138.80
136.61
134.48
131.34
129.27
127.86
127.42
126.57
125.97
125.45
122.12
121.84
116.08
115.91
107.75
106.22
77.41 CDCl3
77.16 CDCl3
76.91 CDCl3
56.23
56.10
17.38
3.07
0.14
MeO
MeO
O
TMS
Me
N
NH
3a
13C NMR
CDCl3
126 MHz
f1 (ppm)

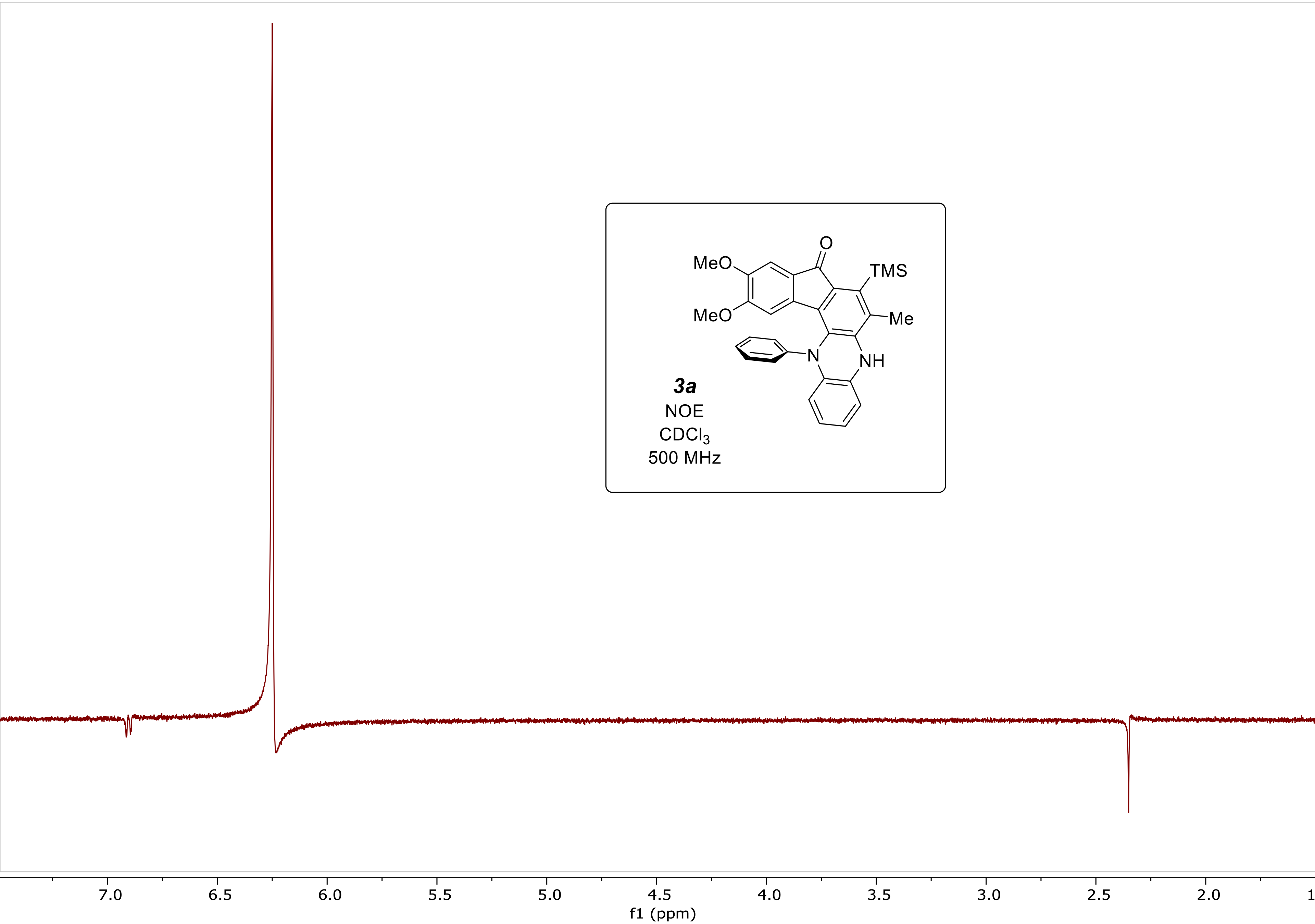
MeO
MeO
O
TMS
Me
N
NH
3a
NOE
$CDCl_3$
500 MHz
7.0
6.5
6.0
5.5
5.0
4.5
4.0
3.5
3.0
2.5
2.0
f1 (ppm)

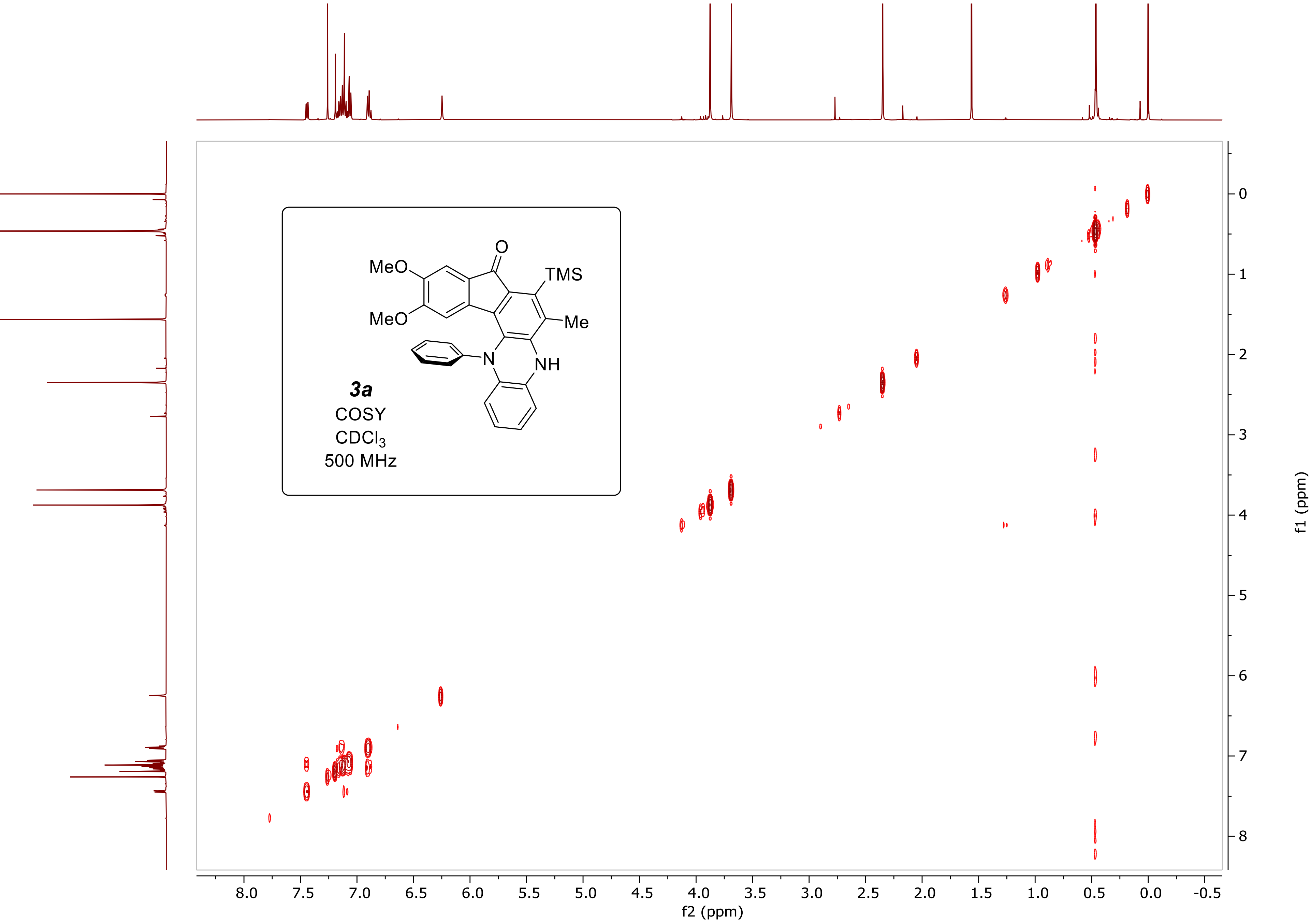
MeO
MeO
O
TMS
Me
N
NH
3a
COSY
CDCl3
500 MHz
f2 (ppm)
f1 (ppm)

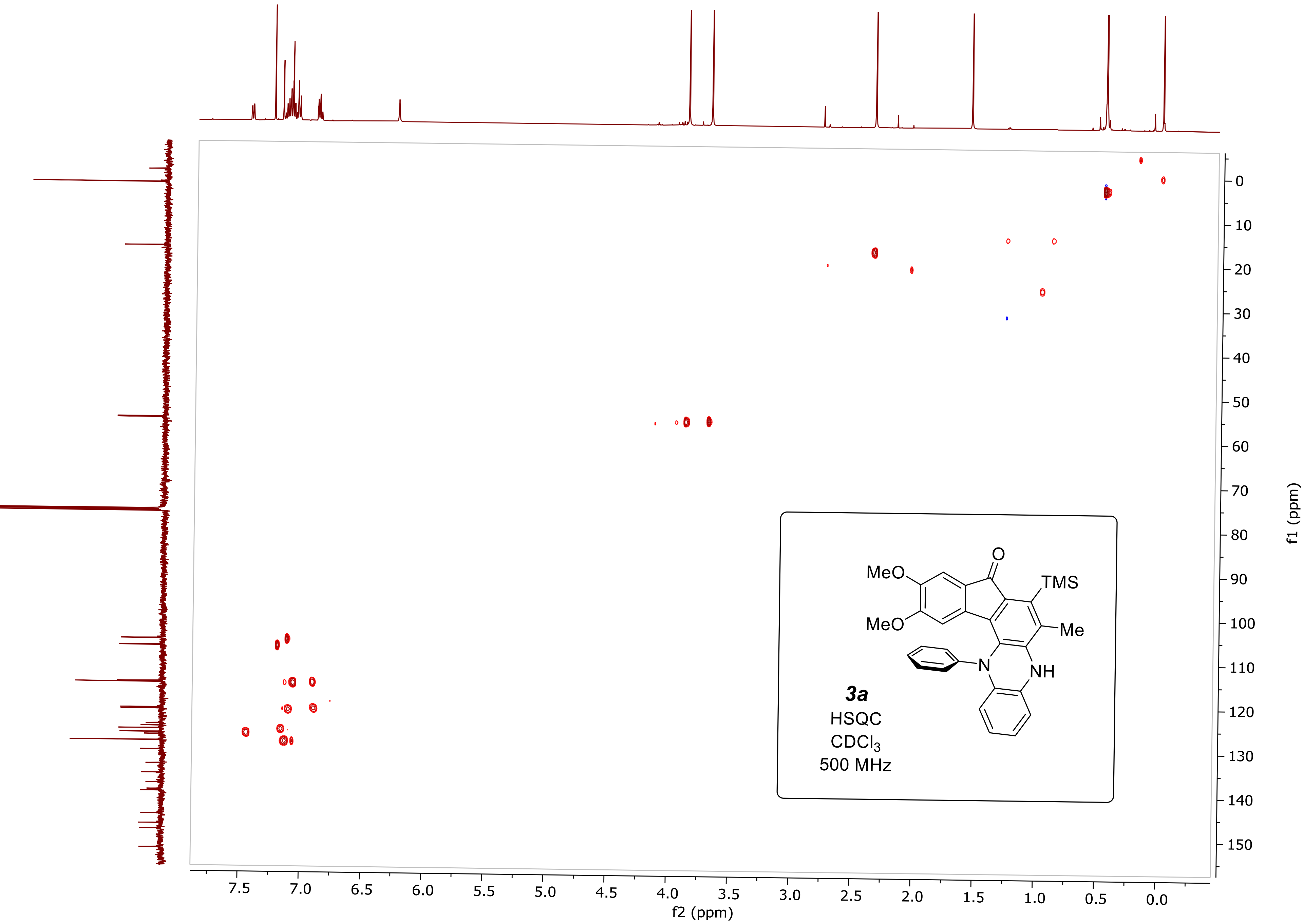
MeO
MeO
O
TMS
Me
N
NH
3a
HSQC
CDCl3
500 MHz
f2 (ppm)
f1 (ppm)
7.5 7.0 6.5 6.0 5.5 5.0 4.5 4.0 3.5 3.0 2.5 2.0 1.5 1.0 0.5 0.0
0 10 20 30 40 50 60 70 80 90 100 110 120 130 140 150

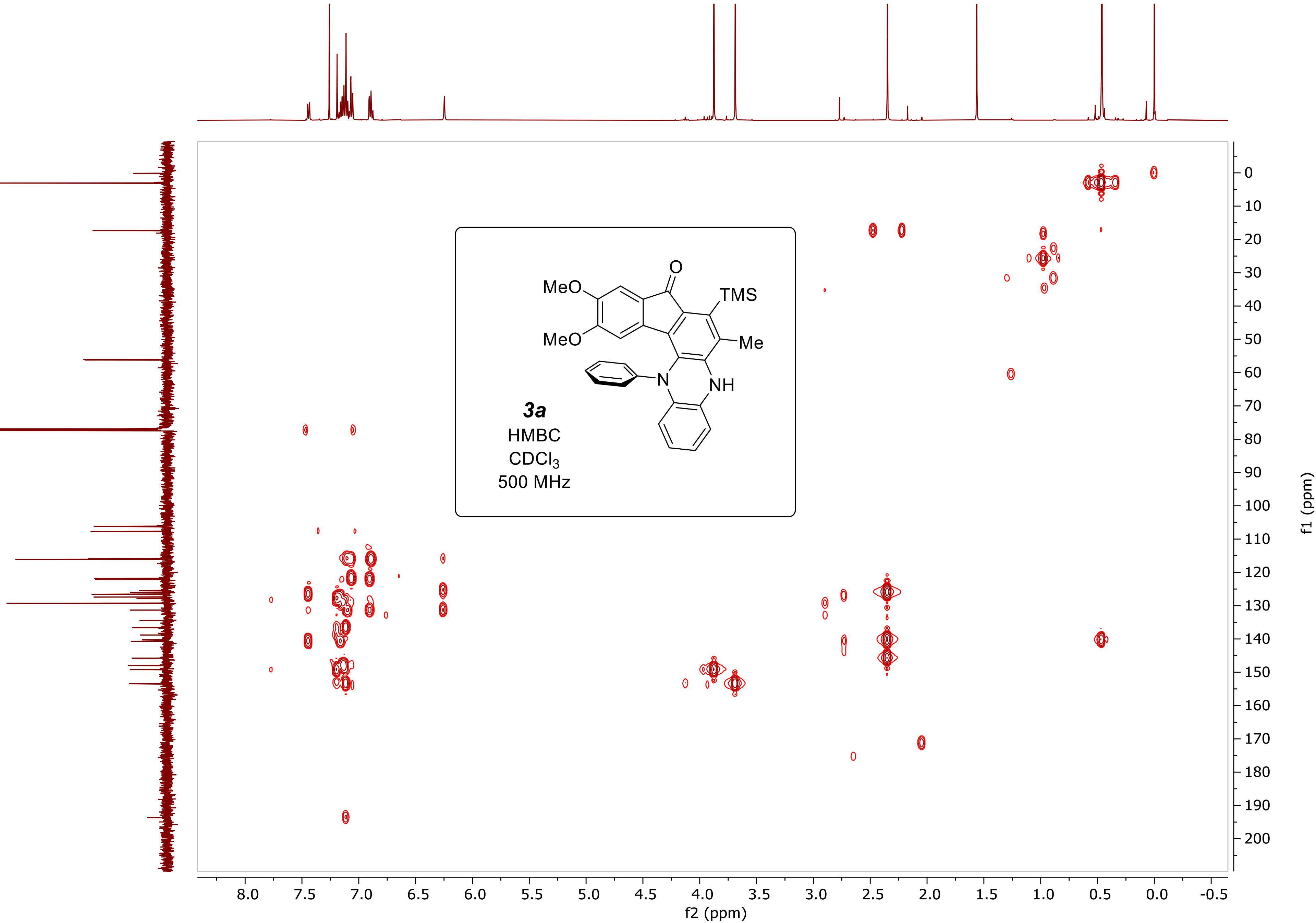

MeO
MeO
O
TMS
Me
N
NH
3a
HMBC
CDCl3
500 MHz
f2 (ppm)
f1 (ppm)

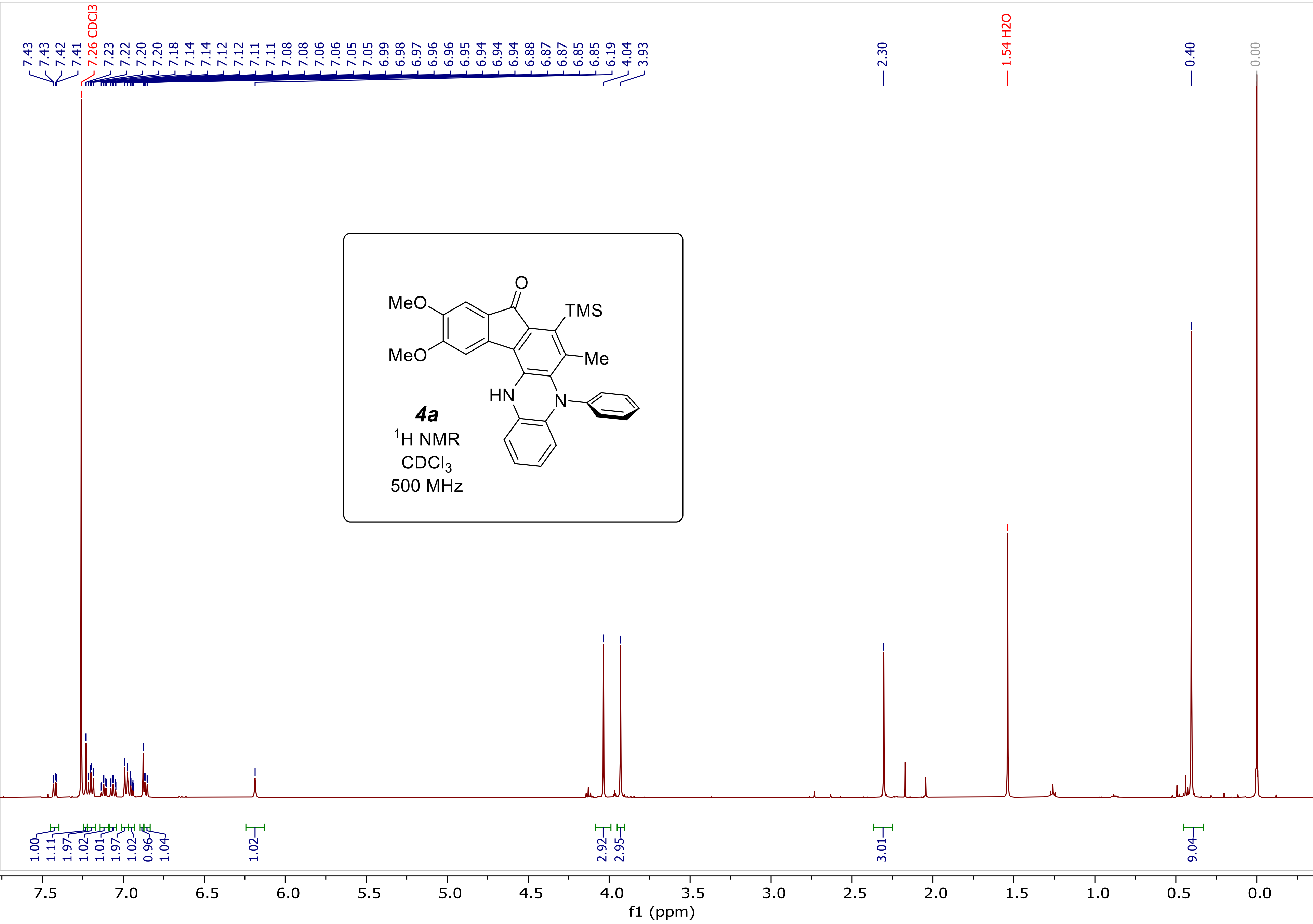
7.43
7.43
7.42
7.41
7.26 CDCl3
7.23
7.22
7.20
7.20
7.18
7.14
7.14
7.12
7.12
7.11
7.11
7.08
7.08
7.06
7.06
7.05
7.05
6.99
6.98
6.97
6.96
6.96
6.95
6.94
6.94
6.94
6.88
6.87
6.87
6.85
6.85
6.19
4.04
3.93
2.30
1.54 H2O
0.40
0.00
MeO
MeO
O
TMS
Me
HN
N
4a
1H NMR
CDCl3
500 MHz
1.00
1.11
1.97
1.02
1.01
1.97
1.02
0.96
1.04
1.02
2.92
2.95
3.01
9.04
7.5
7.0
6.5
6.0
5.5
5.0
4.5
4.0
3.5
3.0
2.5
2.0
1.5
1.0
0.5
0.0
f1 (ppm)

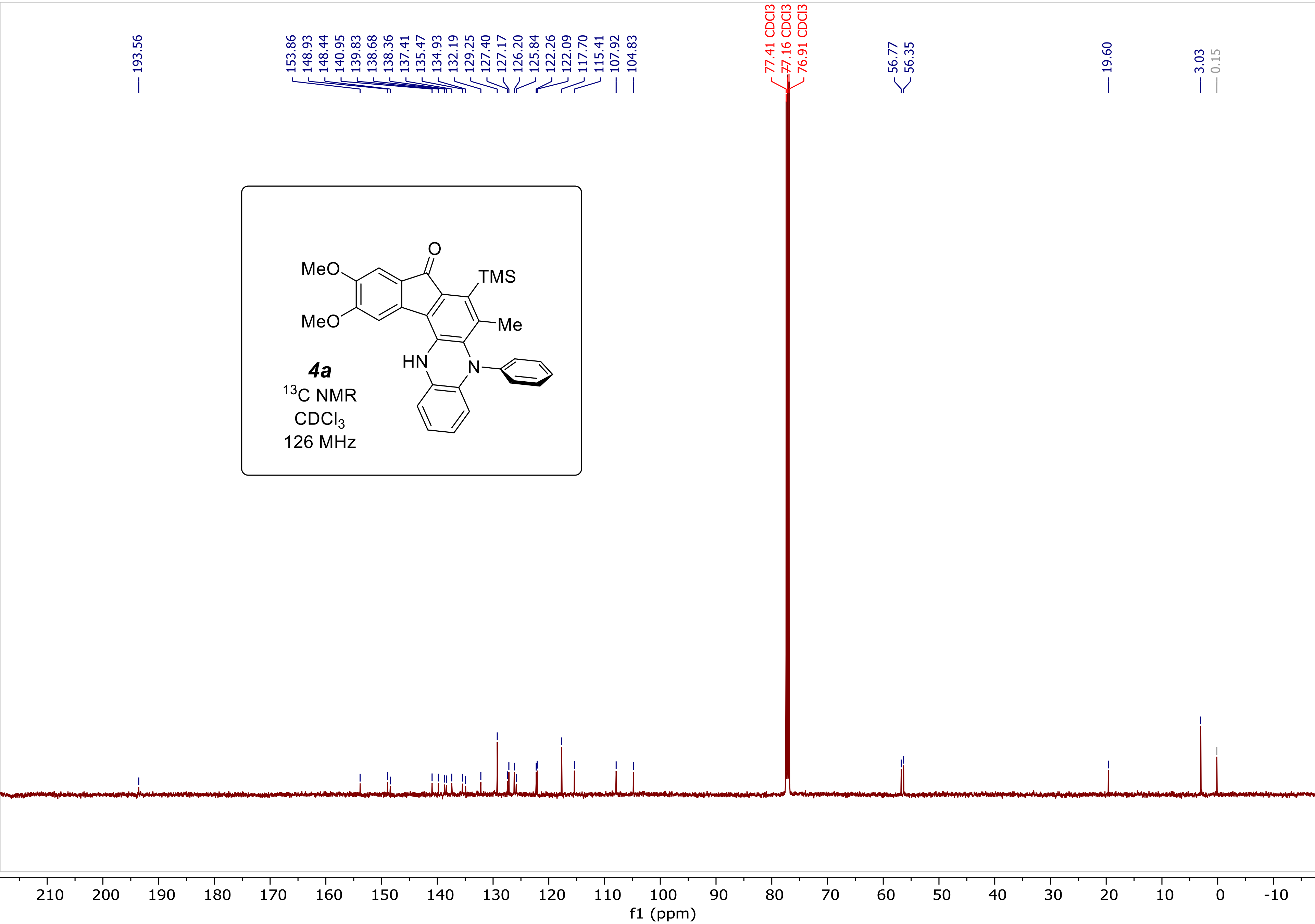
193.56
153.86
148.93
148.44
140.95
139.83
138.68
138.36
137.41
135.47
134.93
132.19
129.25
127.40
127.17
126.20
125.84
122.26
122.09
117.70
115.41
107.92
104.83
77.41 CDCl3
77.16 CDCl3
76.91 CDCl3
56.77
56.35
19.60
3.03
0.15
MeO
MeO
O
TMS
Me
HN
N
4a
13C NMR
CDCl3
126 MHz
210 200 190 180 170 160 150 140 130 120 110 100 90 80 70 60 50 40 30 20 10 0 -10
f1 (ppm)

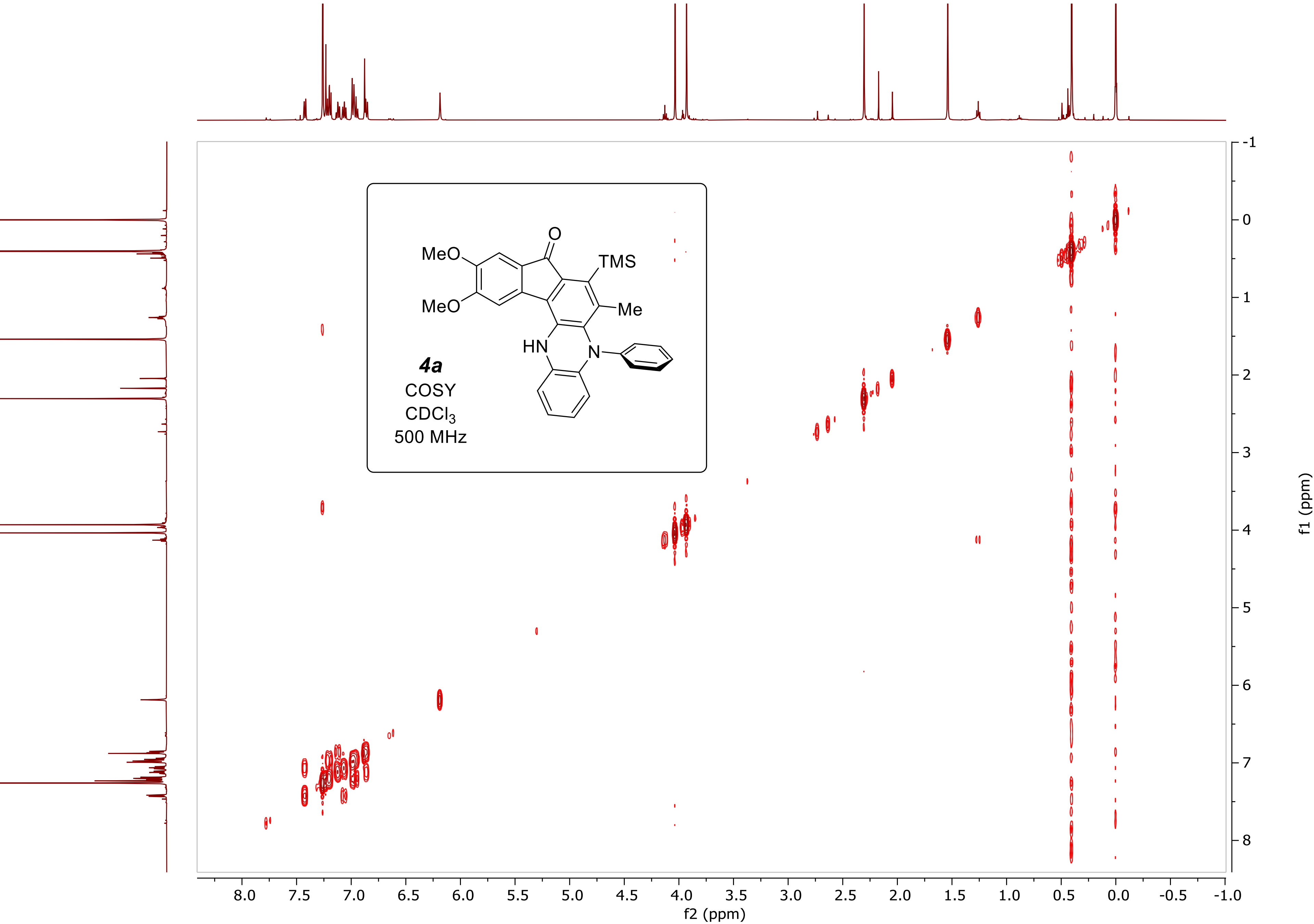
MeO
MeO
O
TMS
Me
HN
N
4a
COSY
CDCl3
500 MHz
f2 (ppm)
f1 (ppm)

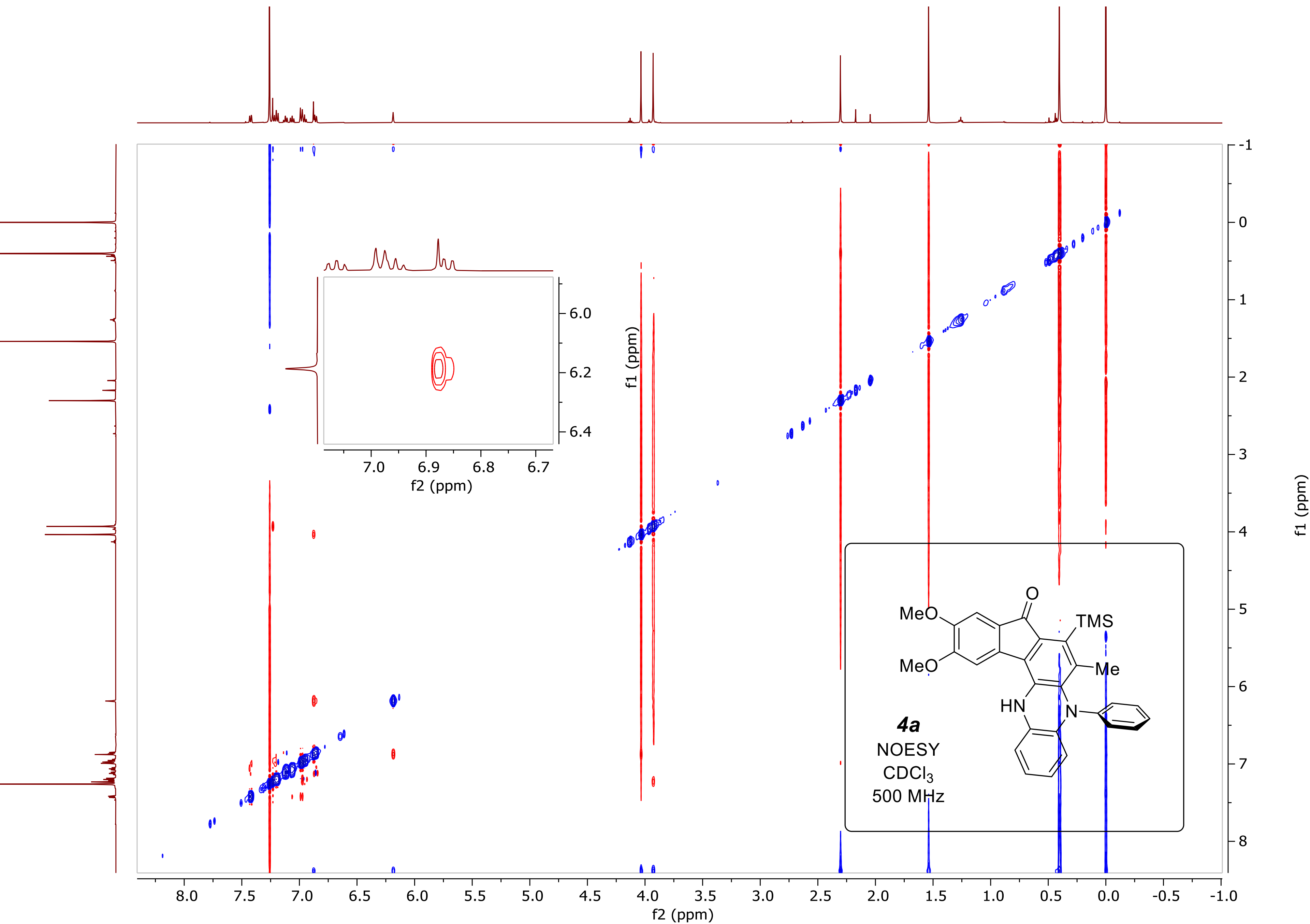

MeO
MeO
O
TMS
Me
HN
N
4a
NOESY
$CDCl_3$
500 MHz
f1 (ppm)
f2 (ppm)

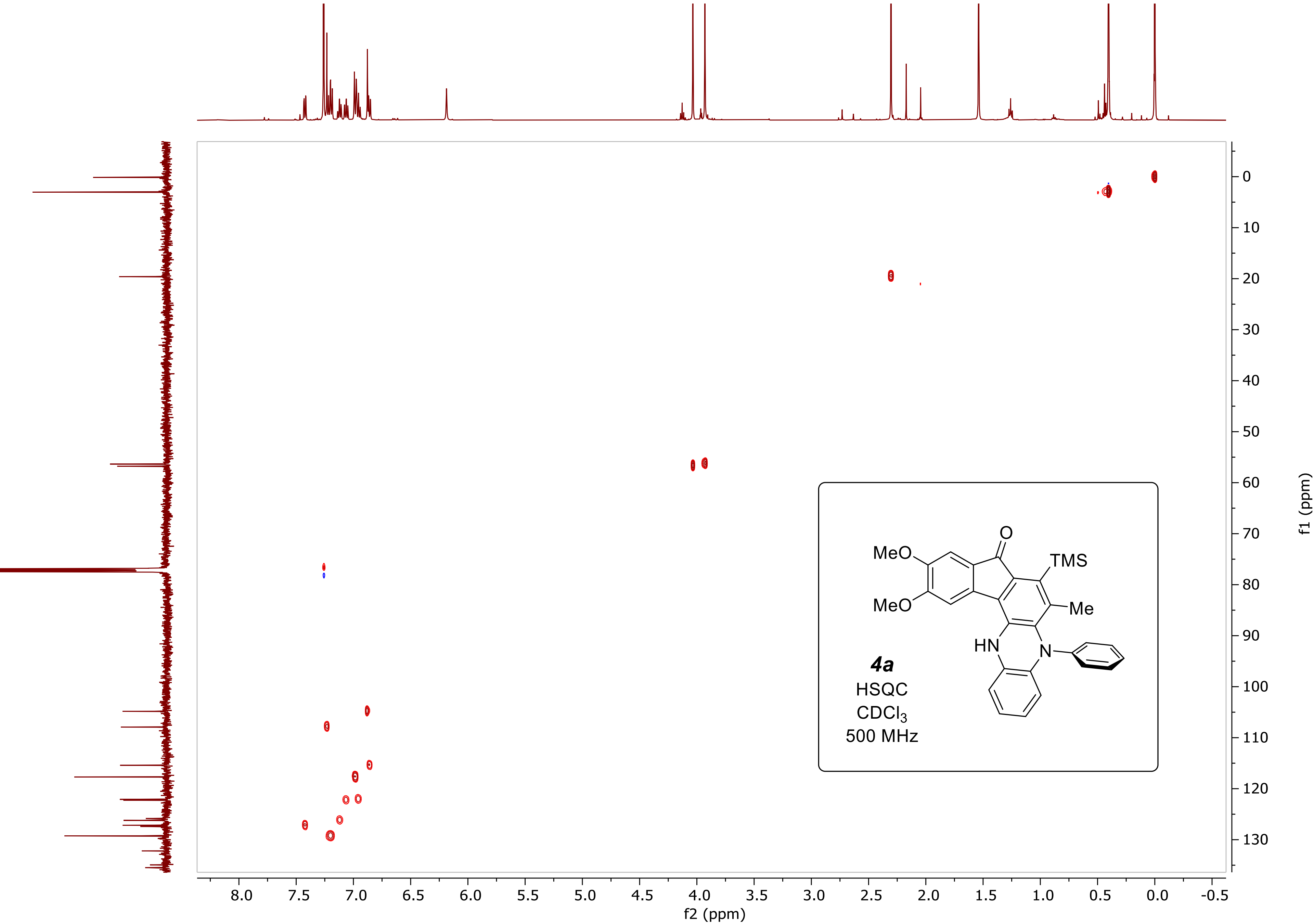
MeO
MeO
O
TMS
Me
HN
N
4a
HSQC
CDCl3
500 MHz
f2 (ppm)
f1 (ppm)
8.0
7.5
7.0
6.5
6.0
5.5
5.0
4.5
4.0
3.5
3.0
2.5
2.0
1.5
1.0
0.5
0.0
-0.5
0
10
20
30
40
50
60
70
80
90
100
110
120
130

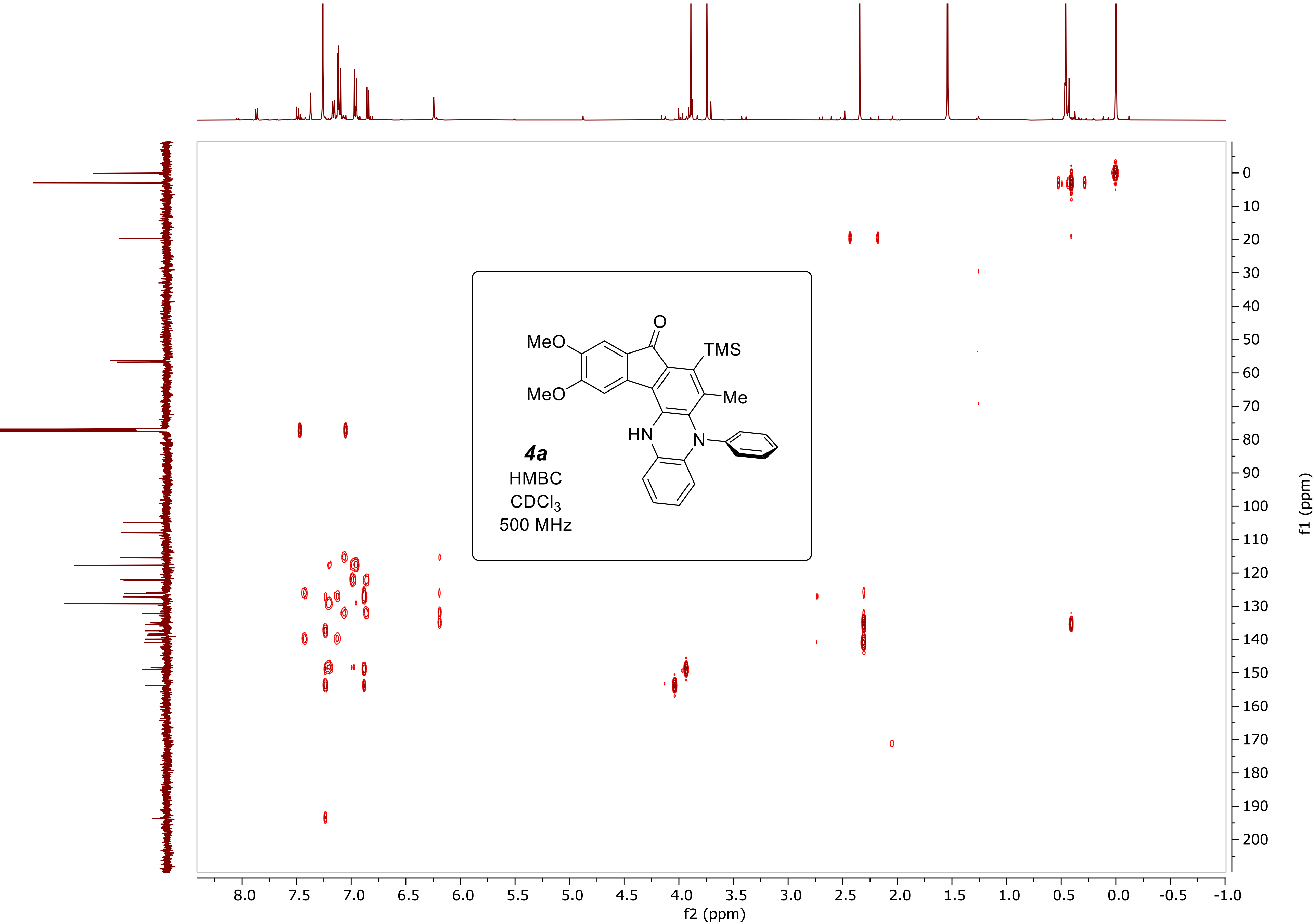
O
MeO
TMS
MeO
Me
HN
N
4a
HMBC
CDCl3
500 MHz
f2 (ppm)
f1 (ppm)
8.0 7.5 7.0 6.5 6.0 5.5 5.0 4.5 4.0 3.5 3.0 2.5 2.0 1.5 1.0 0.5 0.0 -0.5 -1.0
0 10 20 30 40 50 60 70 80 90 100 110 120 130 140 150 160 170 180 190 200

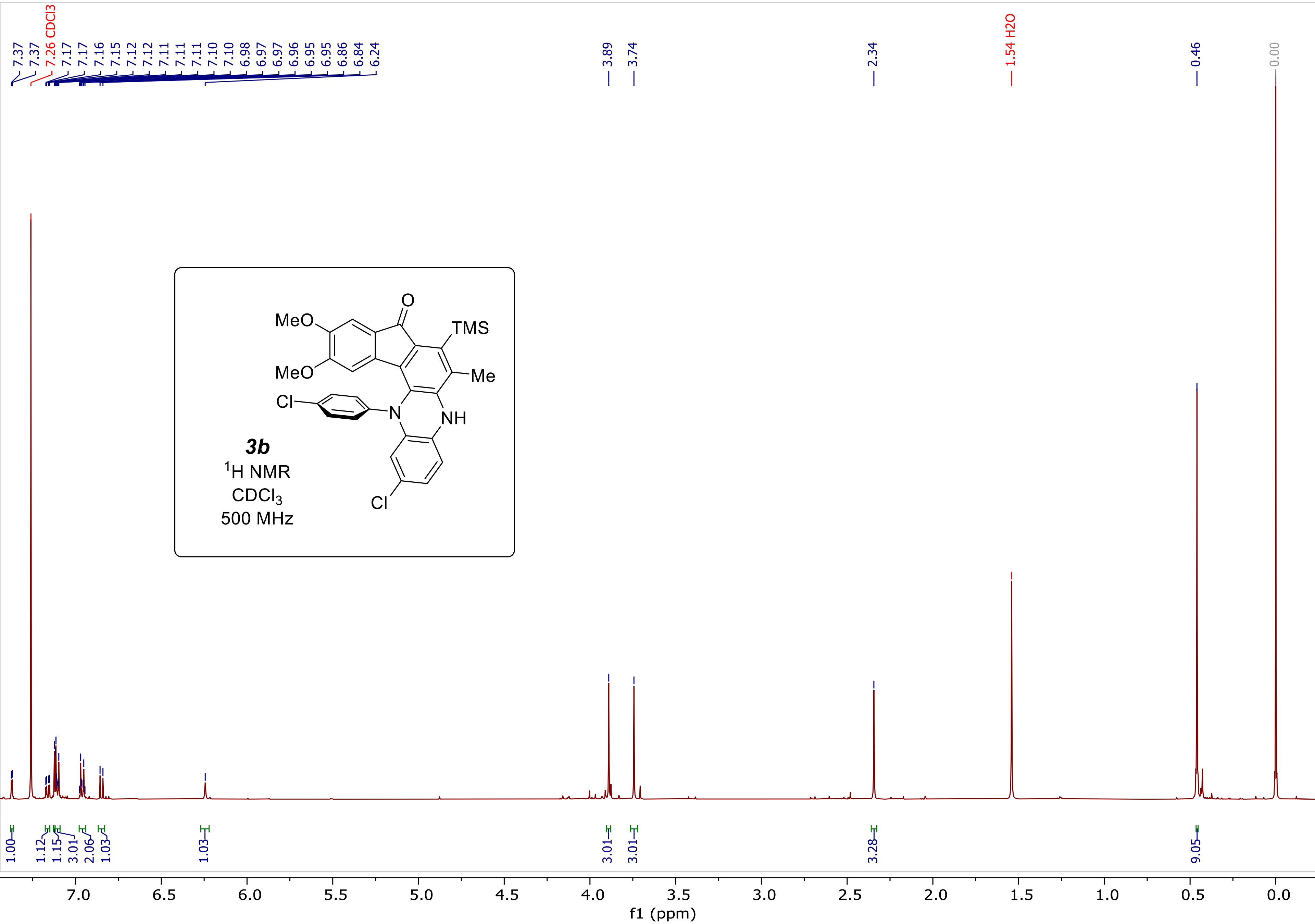
7.37
7.37
7.26 CDCl3
7.17
7.17
7.16
7.15
7.12
7.12
7.11
7.11
7.11
7.10
7.10
6.98
6.97
6.97
6.96
6.95
6.95
6.86
6.84
6.24
3.89
3.74
2.34
1.54 H2O
0.46
0.00
MeO
MeO
O
TMS
Me
Cl
N
NH
Cl
3b
1H NMR
CDCl3
500 MHz
1.00
1.12
1.15
3.01
2.06
1.03
1.03
3.01
3.01
3.28
9.05
7.0
6.5
6.0
5.5
5.0
4.5
4.0
3.5
3.0
2.5
2.0
1.5
1.0
0.5
0.0
f1 (ppm)

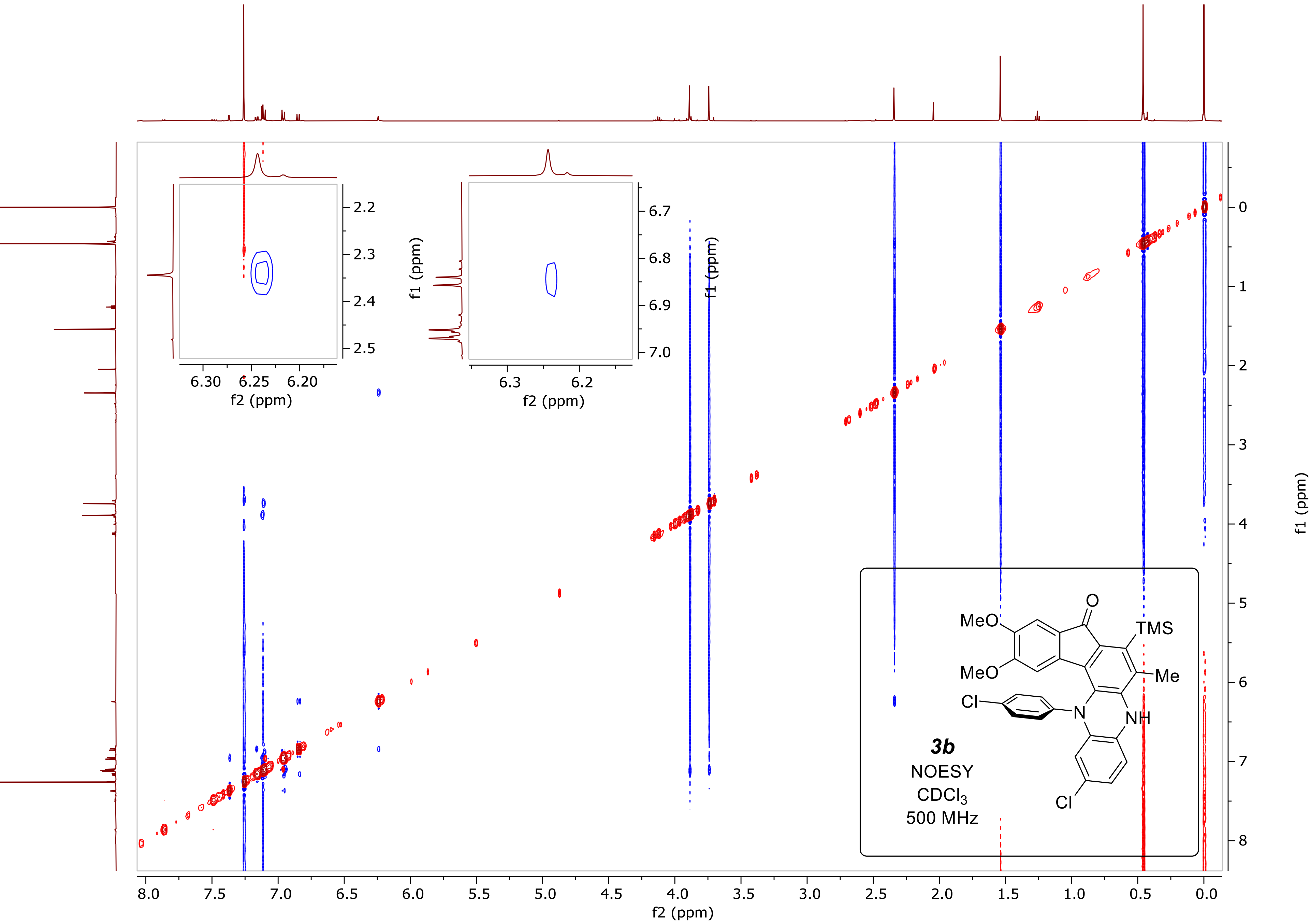
6.30
6.25
6.20
f2 (ppm)
2.2
2.3
2.4
2.5
f1 (ppm)
6.3
6.2
6.7
6.8
6.9
7.0
MeO
MeO
O
TMS
Me
Cl
N
NH
Cl
3b
NOESY
CDCl3
500 MHz
f2 (ppm)
f1 (ppm)

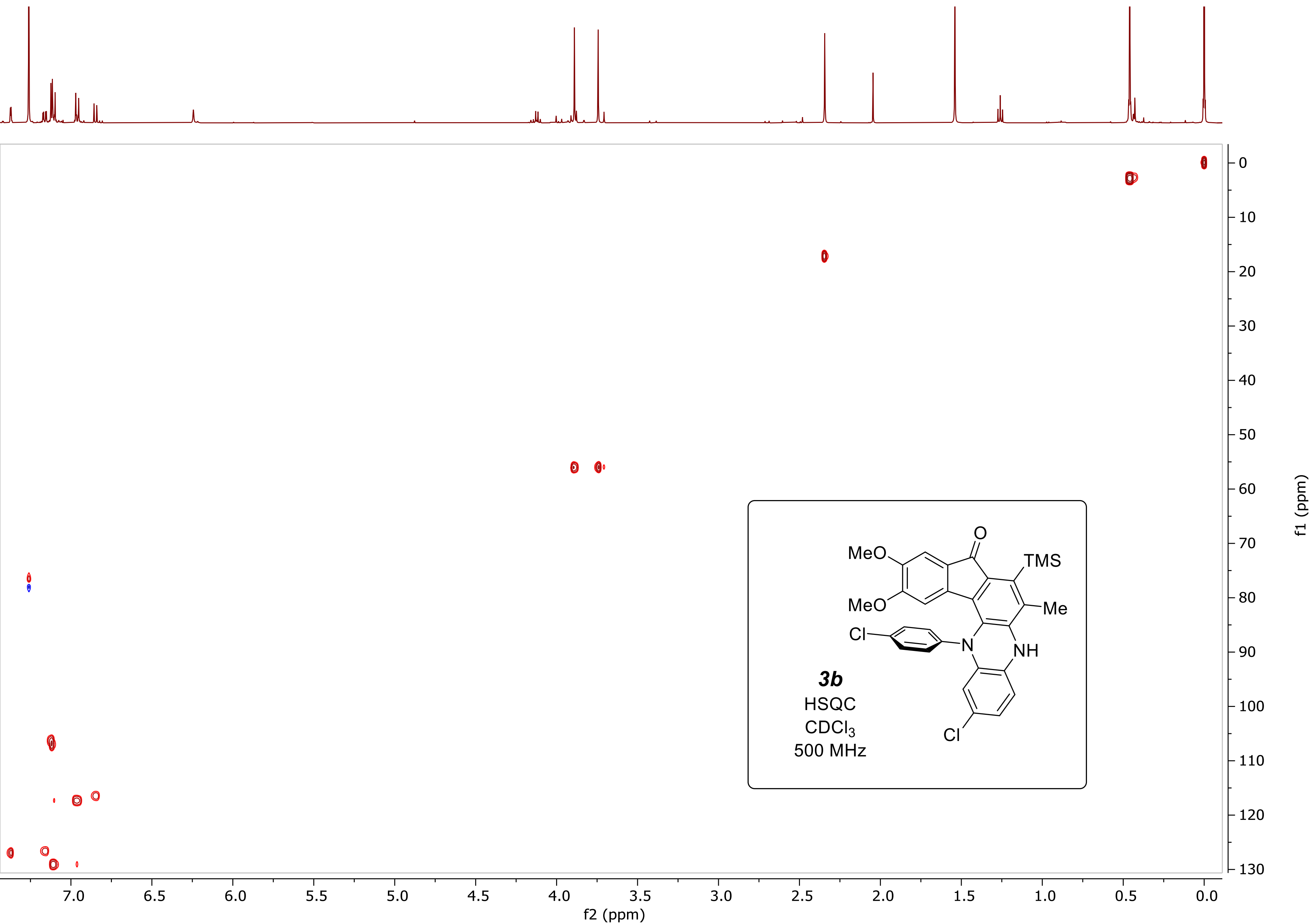
MeO
MeO
O
TMS
Me
Cl
N
NH
Cl
3b
HSQC
CDCl3
500 MHz
f2 (ppm)
f1 (ppm)
7.0 6.5 6.0 5.5 5.0 4.5 4.0 3.5 3.0 2.5 2.0 1.5 1.0 0.5 0.0
0 10 20 30 40 50 60 70 80 90 100 110 120 130

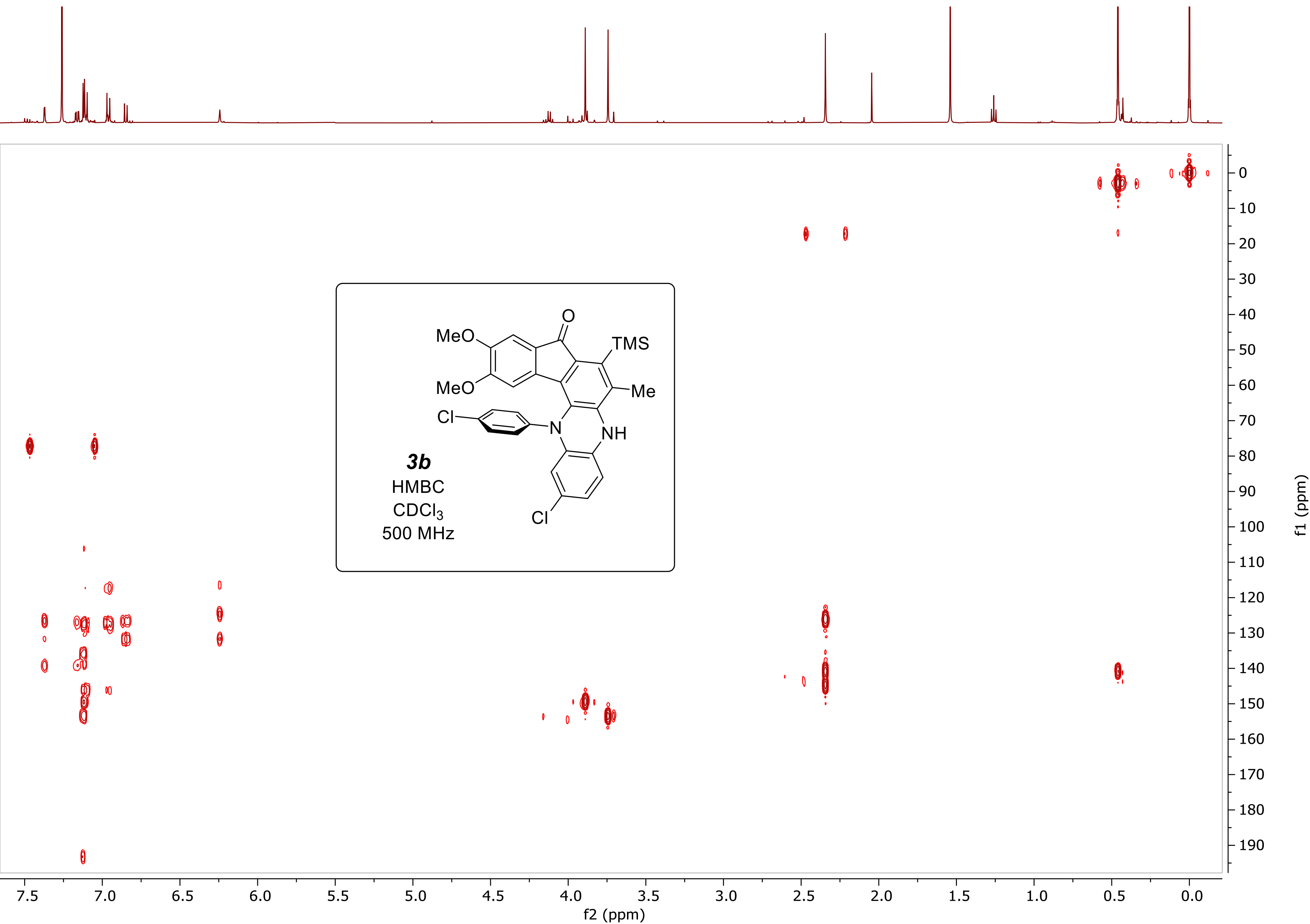
MeO
MeO
O
TMS
Me
Cl
N
NH
Cl
3b
HMBC
CDCl3
500 MHz
f2 (ppm)
f1 (ppm)

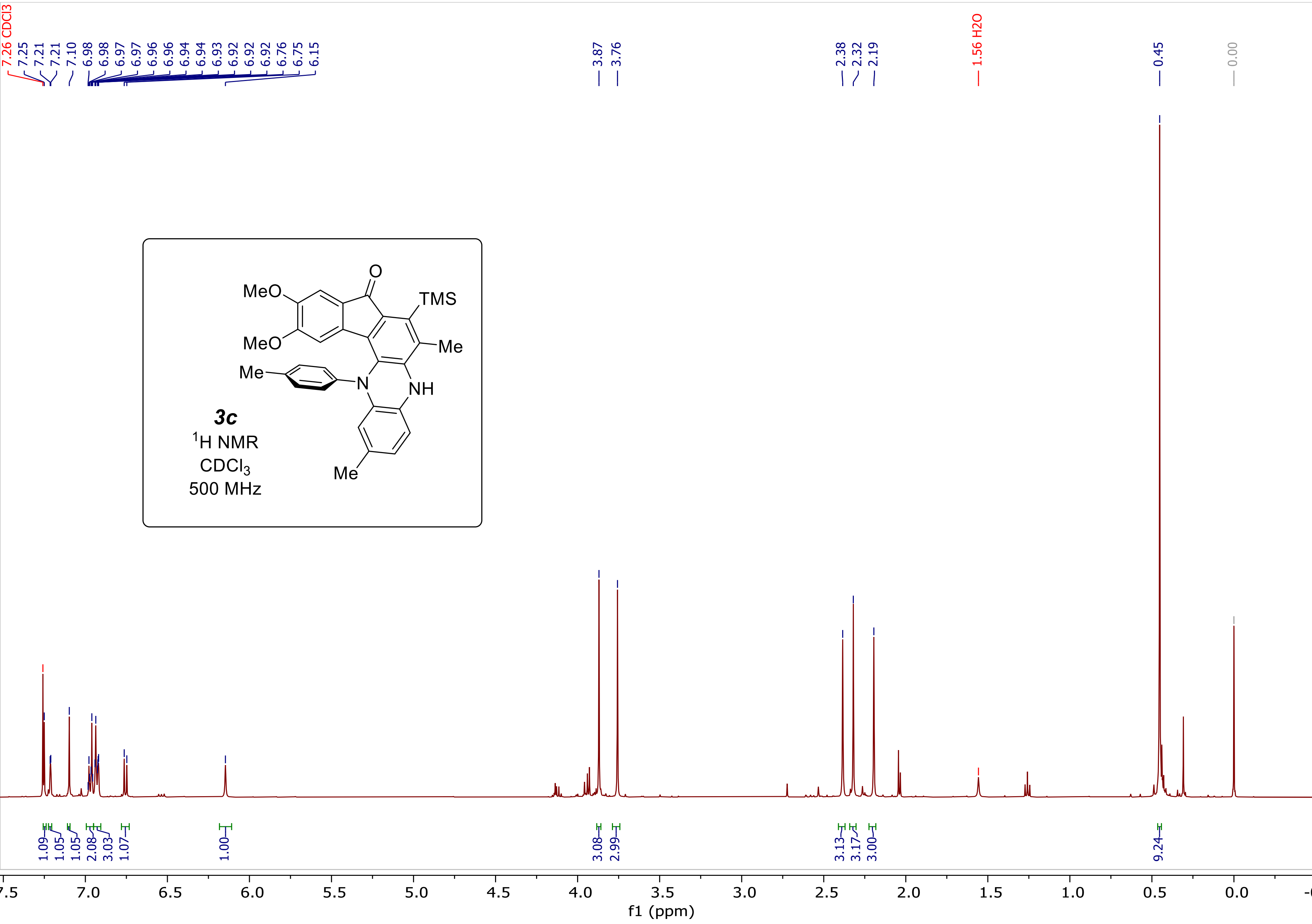
7.26 CDCl3
7.25
7.21
7.21
7.10
6.98
6.98
6.97
6.97
6.96
6.96
6.94
6.94
6.93
6.92
6.92
6.92
6.76
6.75
6.15
3.87
3.76
2.38
2.32
2.19
1.56 H2O
0.45
0.00
MeO
MeO
O
TMS
Me
Me
N
NH
Me
3c
1H NMR
CDCl3
500 MHz
1.09
1.05
1.05
2.08
3.03
1.07
1.00
3.08
2.99
3.13
3.17
3.00
9.24
7.5
7.0
6.5
6.0
5.5
5.0
4.5
4.0
3.5
3.0
2.5
2.0
1.5
1.0
0.5
0.0
f1 (ppm)

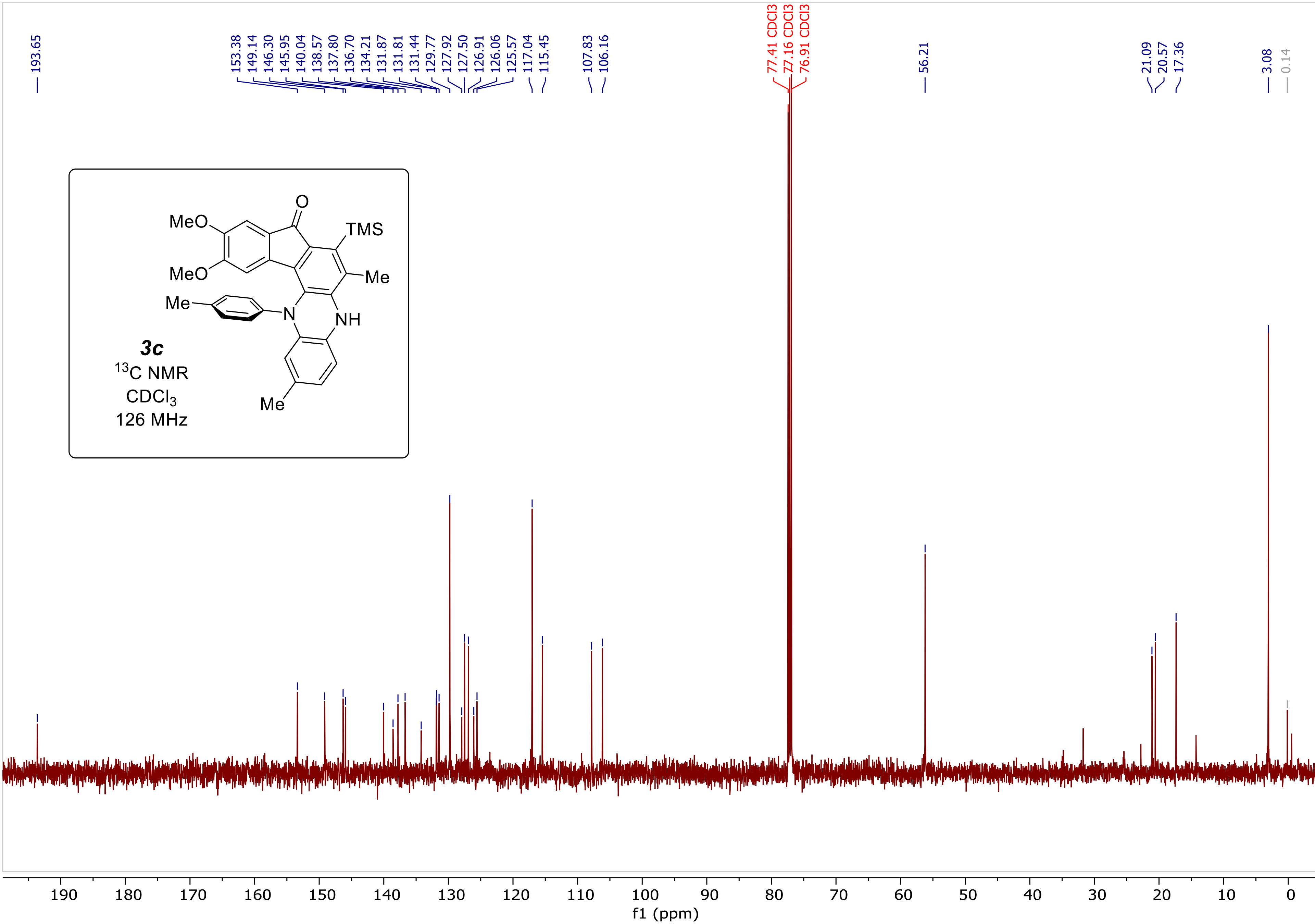
193.65
153.38
149.14
146.30
145.95
140.04
138.57
137.80
136.70
134.21
131.87
131.81
131.44
129.77
127.92
127.50
126.91
126.06
125.57
117.04
115.45
107.83
106.16
77.41 CDCl3
77.16 CDCl3
76.91 CDCl3
56.21
21.09
20.57
17.36
3.08
0.14
MeO
MeO
O
TMS
Me
Me
N
NH
Me
3c
13C NMR
CDCl3
126 MHz
190 180 170 160 150 140 130 120 110 100 90 80 70 60 50 40 30 20 10 0
f1 (ppm)

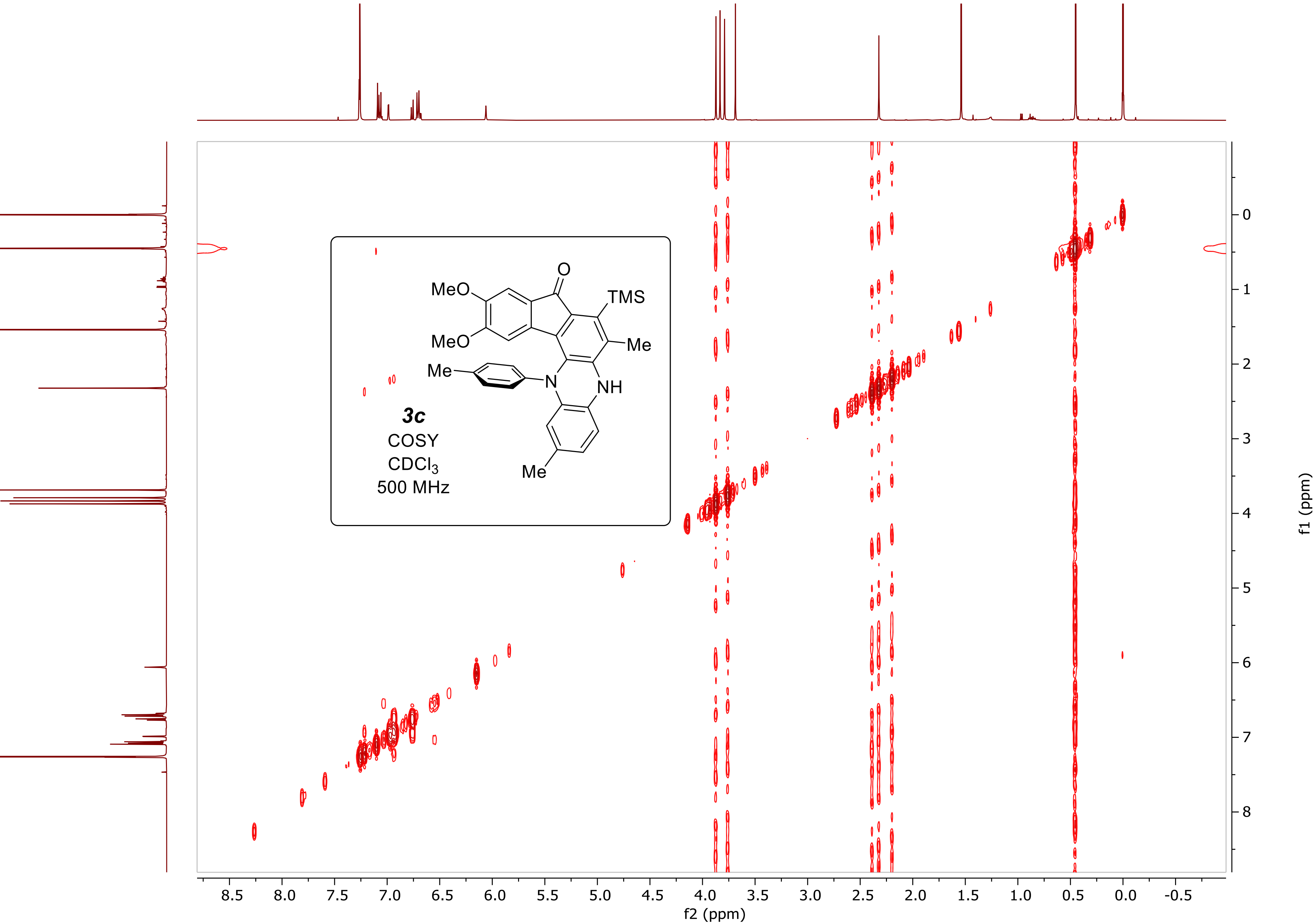
MeO
MeO
O
TMS
Me
Me
N
NH
Me
3c
COSY
CDCl3
500 MHz
8.5
8.0
7.5
7.0
6.5
6.0
5.5
5.0
4.5
4.0
3.5
3.0
2.5
2.0
1.5
1.0
0.5
0.0
-0.5
f2 (ppm)
0
1
2
3
4
5
6
7
8
f1 (ppm)

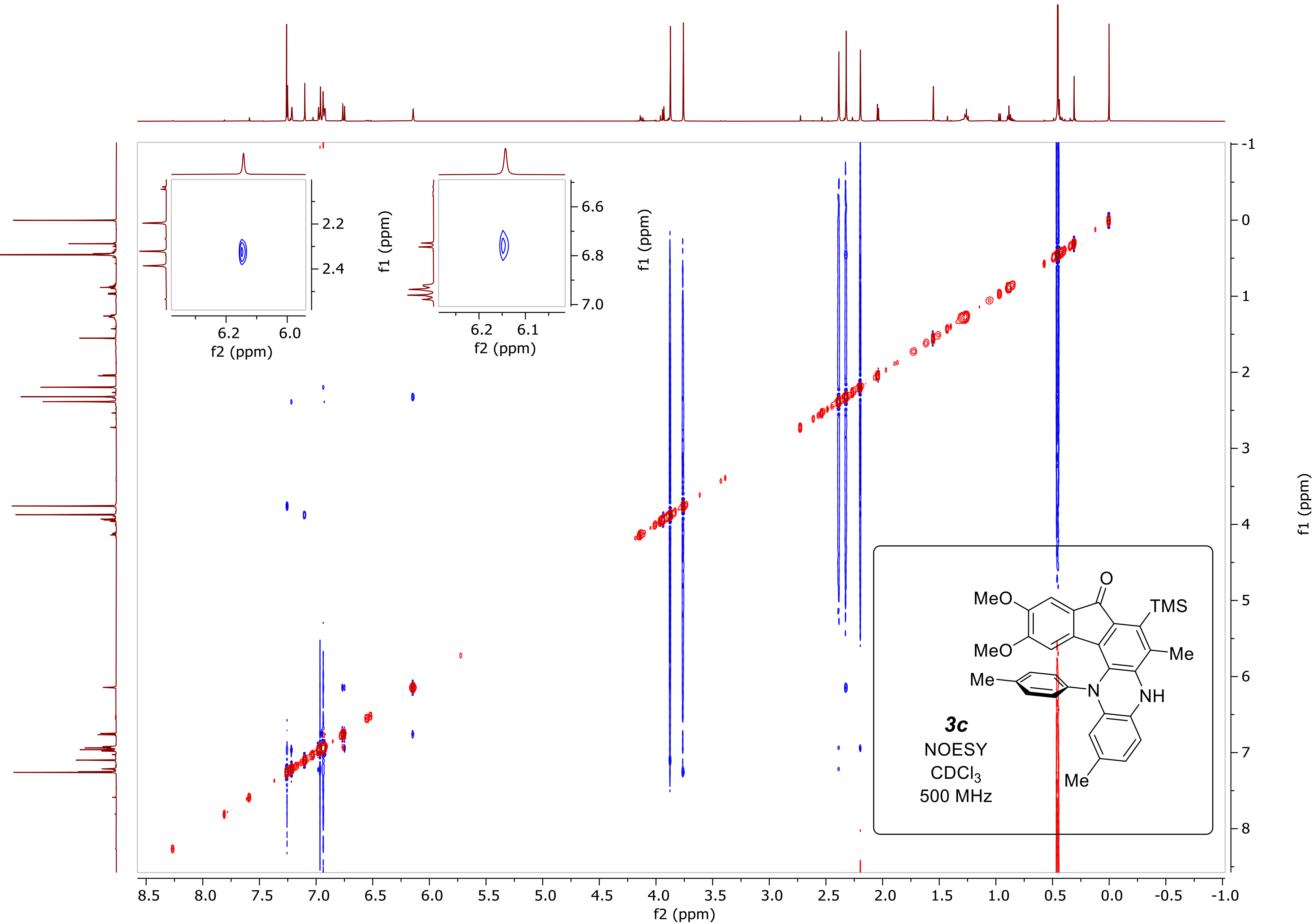
f2 (ppm)
f1 (ppm)
MeO
MeO
O
TMS
Me
Me
N
NH
Me
3c
NOESY
CDCl3
500 MHz

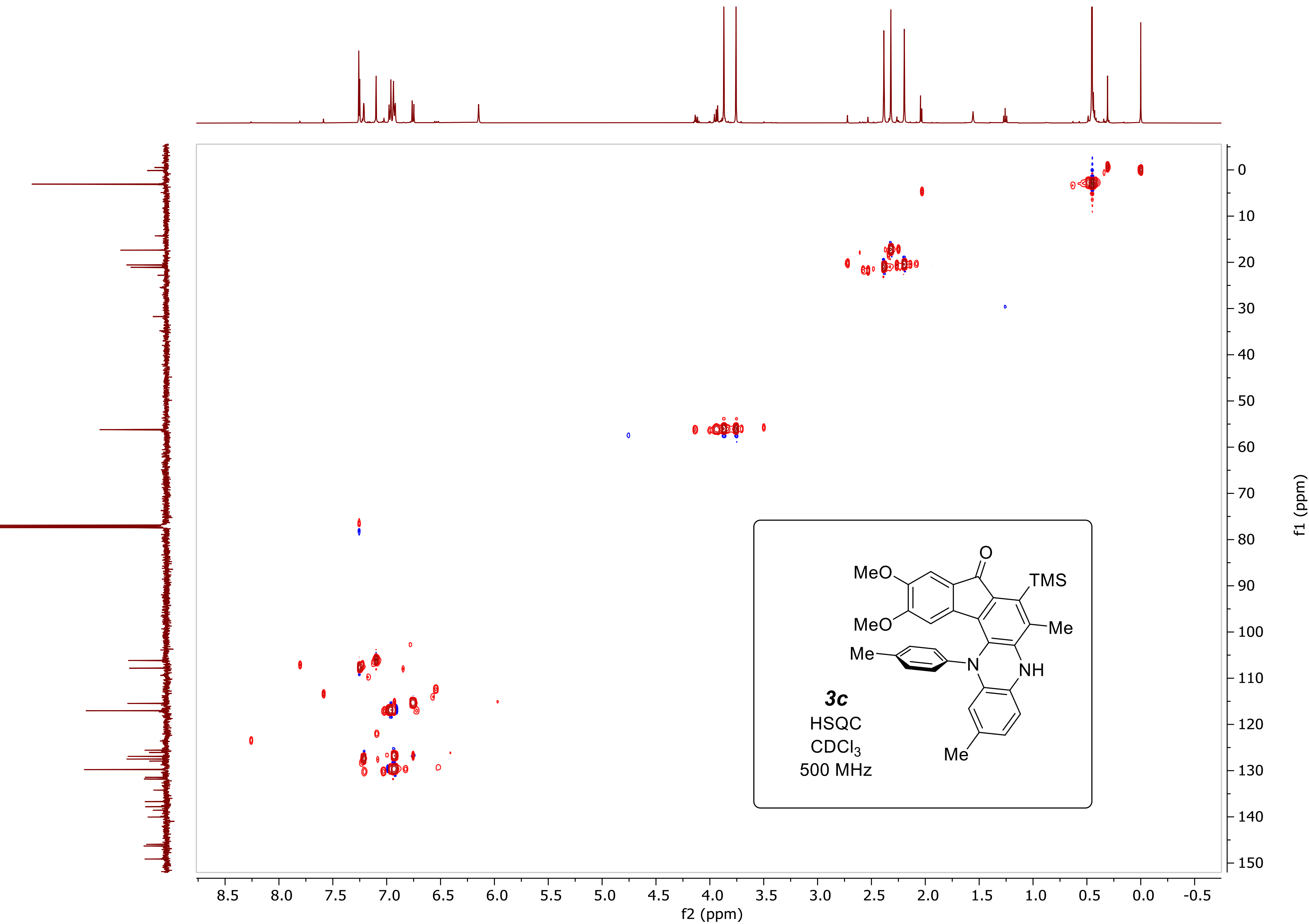
f2 (ppm)
f1 (ppm)
MeO
MeO
O
TMS
Me
Me
N
NH
Me
3c
HSQC
$CDCl_3$
500 MHz

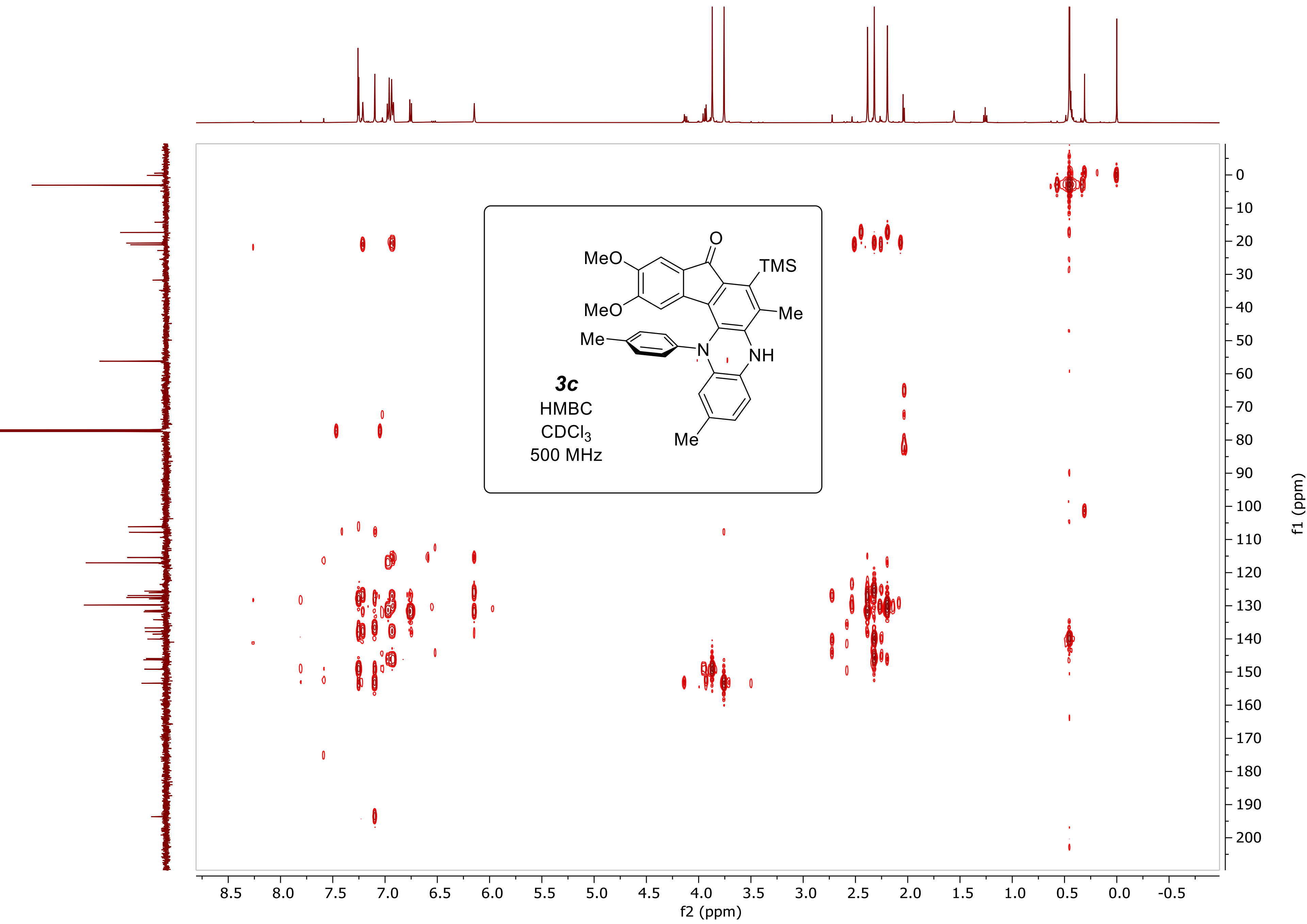
MeO
MeO
O
TMS
Me
Me
N
NH
Me
3c
HMBC
CDCl3
500 MHz
f2 (ppm)
f1 (ppm)

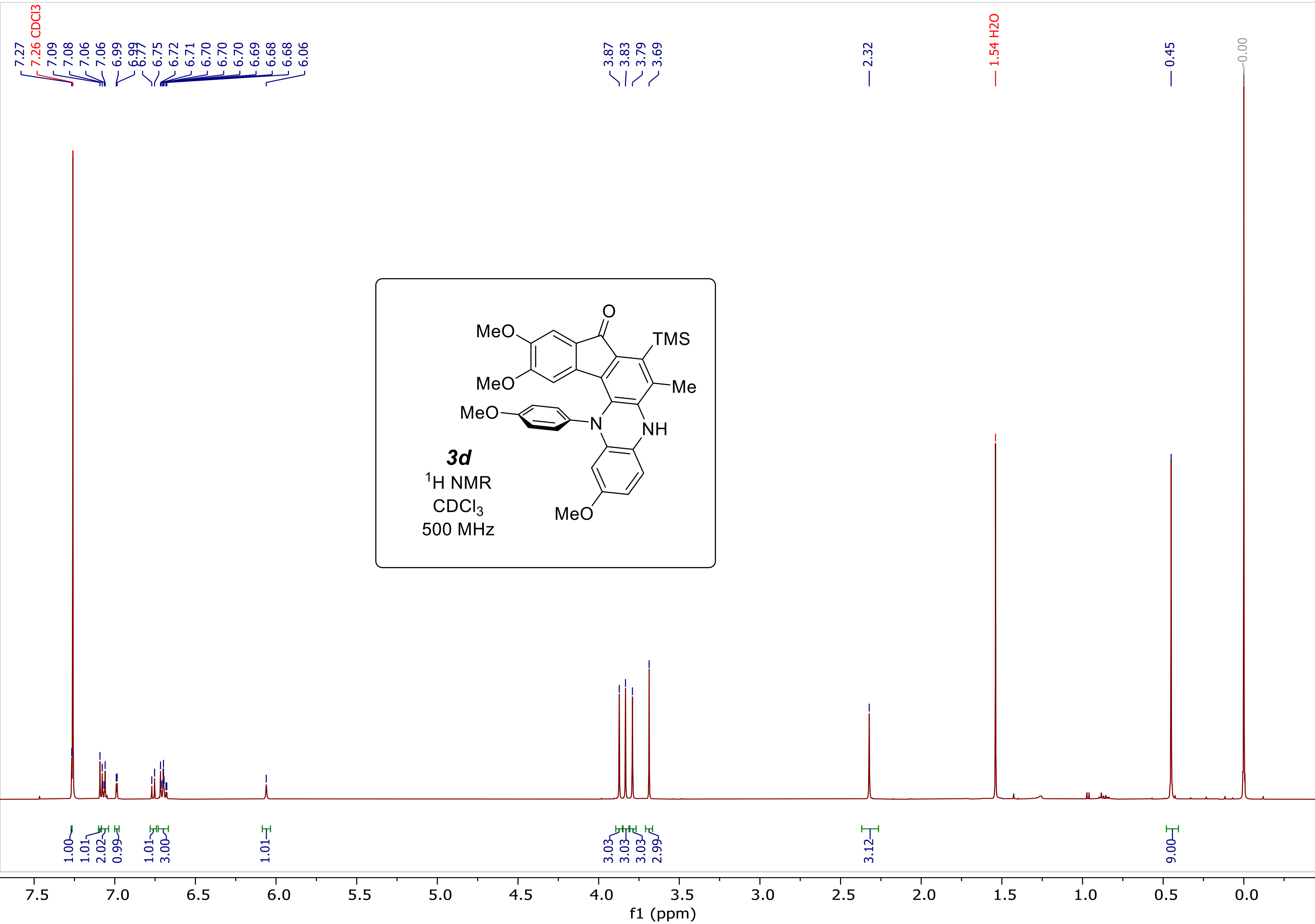
7.27
7.26 CDCl3
7.09
7.08
7.06
7.06
6.99
6.99
6.77
6.75
6.72
6.71
6.70
6.70
6.70
6.69
6.68
6.68
6.06
3.87
3.83
3.79
3.69
2.32
1.54 H2O
0.45
0.00
MeO
MeO
MeO
MeO
O
TMS
Me
N
NH
3d
1H NMR
CDCl3
500 MHz
1.00
1.01
2.02
0.99
1.01
3.00
1.01
3.03
3.03
3.03
2.99
3.12
9.00
7.5
7.0
6.5
6.0
5.5
5.0
4.5
4.0
3.5
3.0
2.5
2.0
1.5
1.0
0.5
0.0
f1 (ppm)

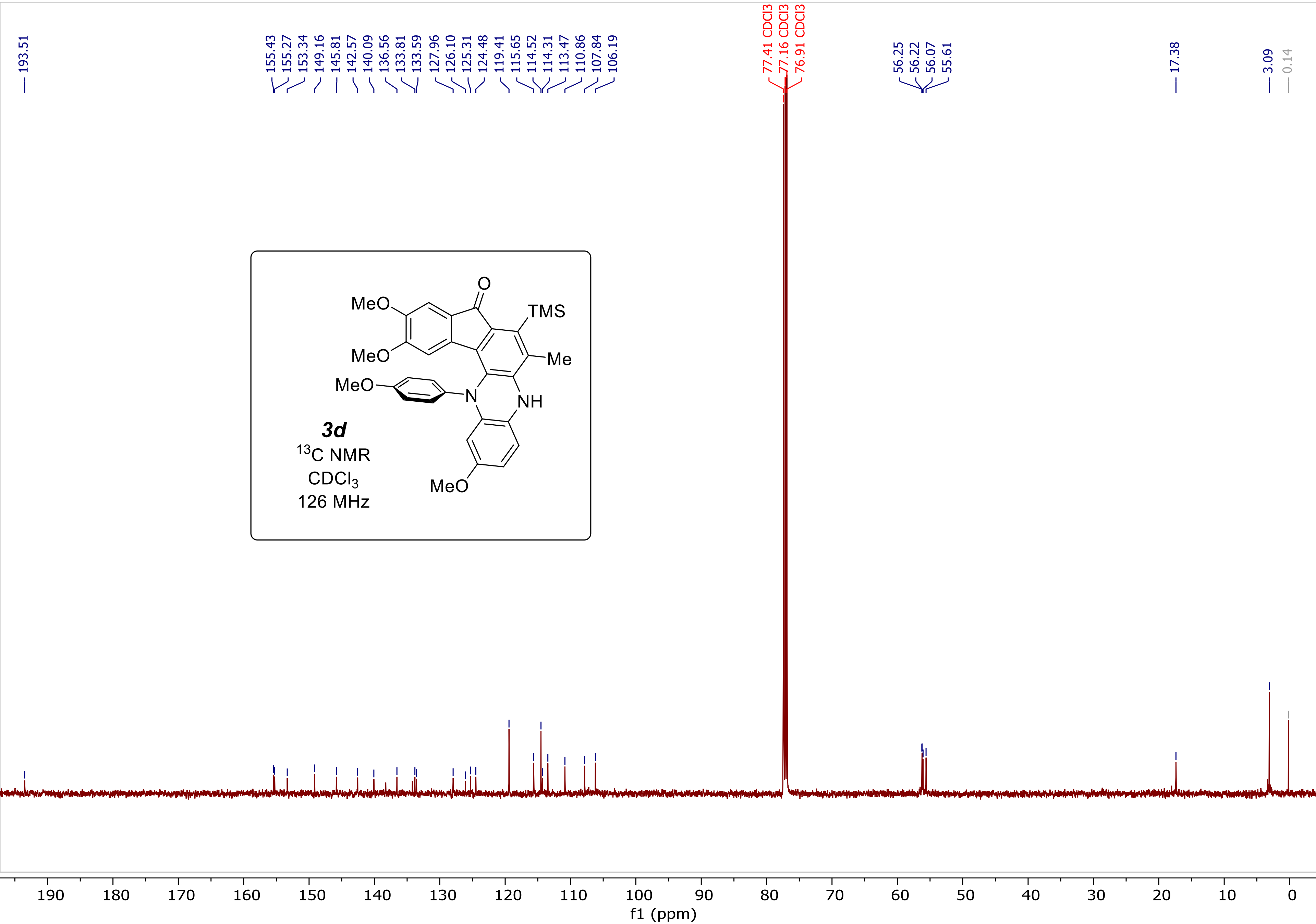
193.51
155.43
155.27
153.34
149.16
145.81
142.57
140.09
136.56
133.81
133.59
127.96
126.10
125.31
124.48
119.41
115.65
114.52
114.31
113.47
110.86
107.84
106.19
77.41 CDCl3
77.16 CDCl3
76.91 CDCl3
56.25
56.22
56.07
55.61
17.38
3.09
0.14
MeO
MeO
MeO
MeO
O
TMS
Me
N
NH
3d
13C NMR
CDCl3
126 MHz
190 180 170 160 150 140 130 120 110 100 90 80 70 60 50 40 30 20 10 0
f1 (ppm)

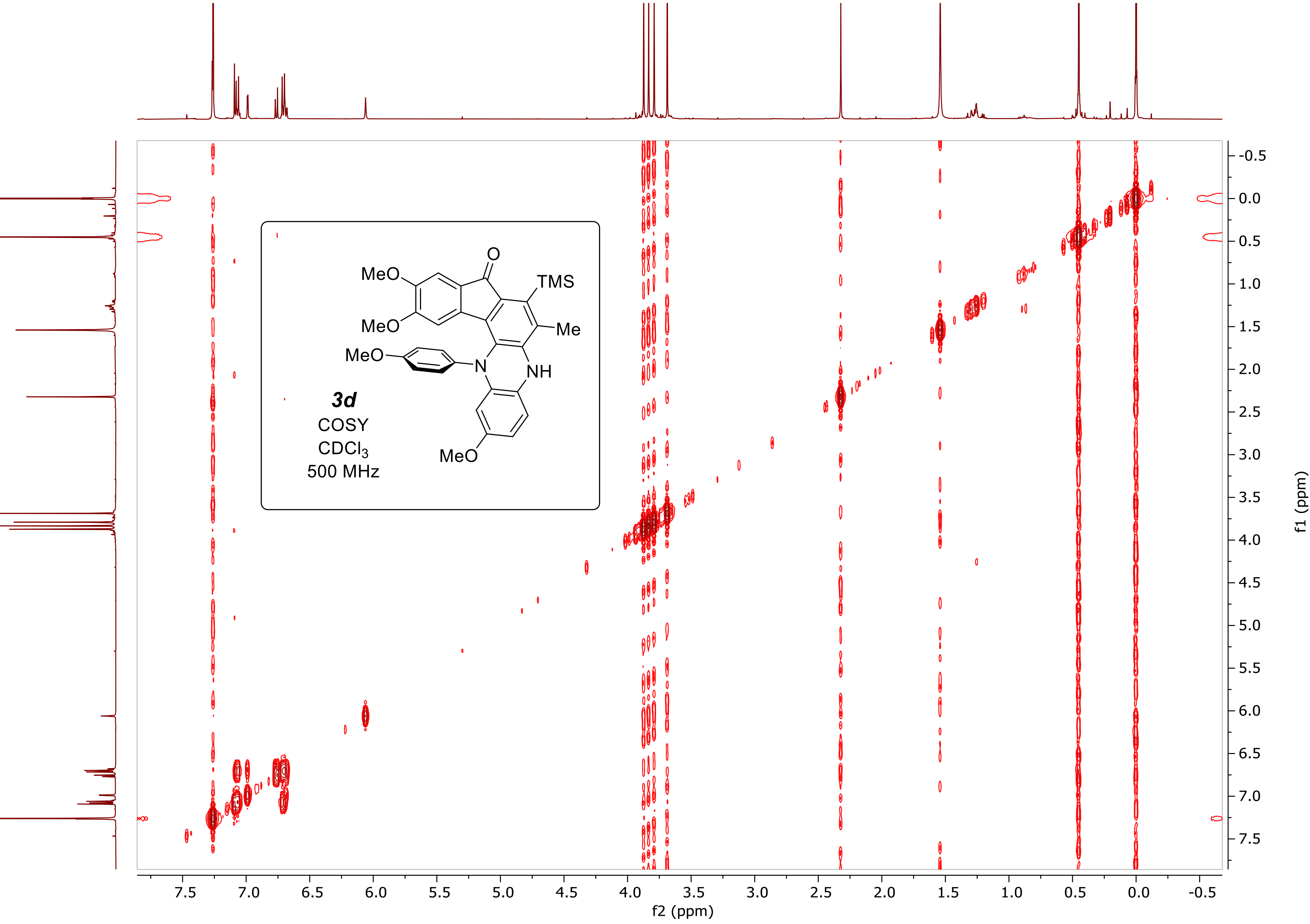

O
MeO
MeO
TMS
Me
MeO
N
NH
MeO
3d
COSY
CDCl3
500 MHz
f2 (ppm)
f1 (ppm)

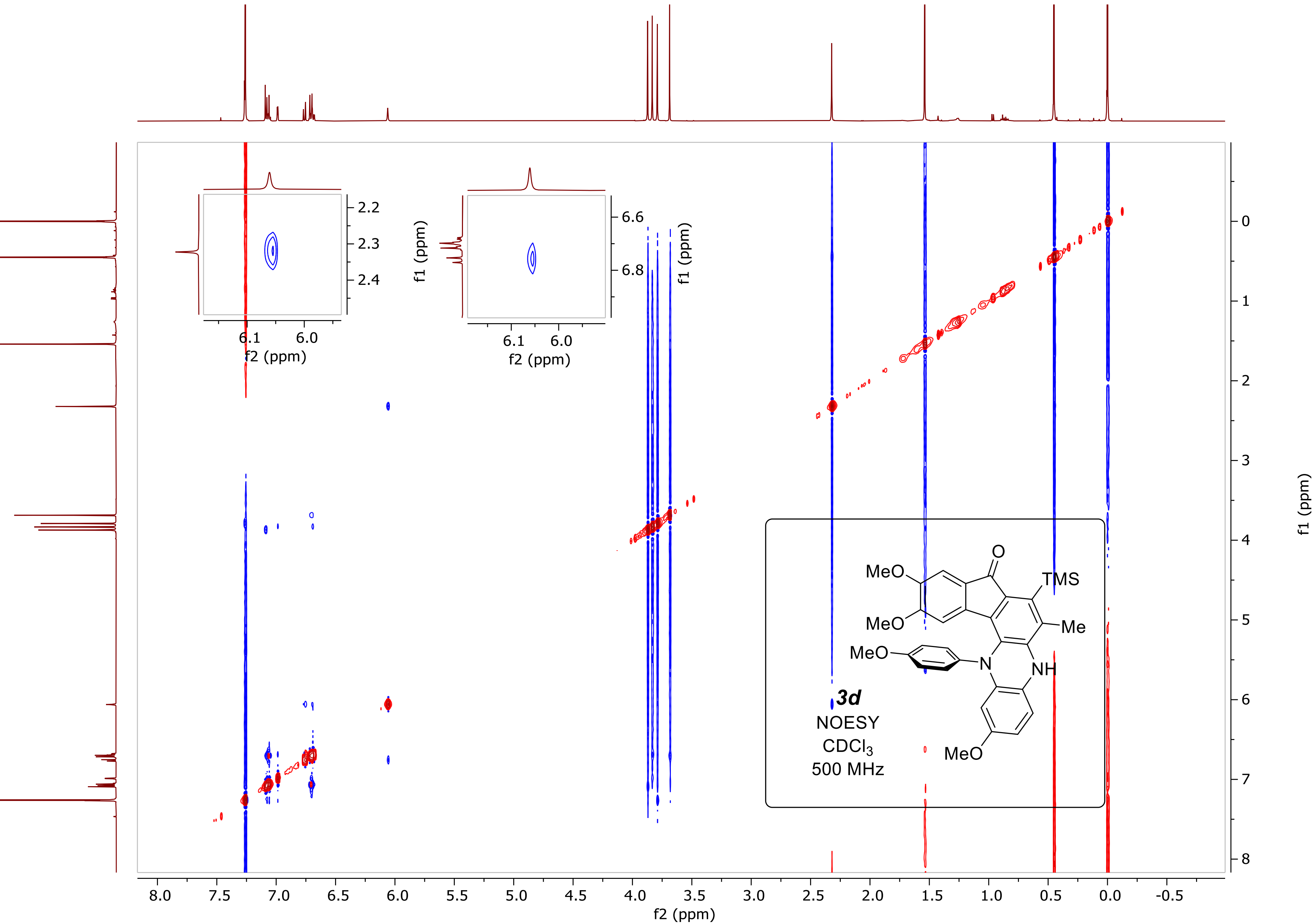
3d
NOESY
CDCl3
500 MHz
MeO
MeO
MeO
MeO
O
TMS
Me
N
NH
f2 (ppm)
f1 (ppm)

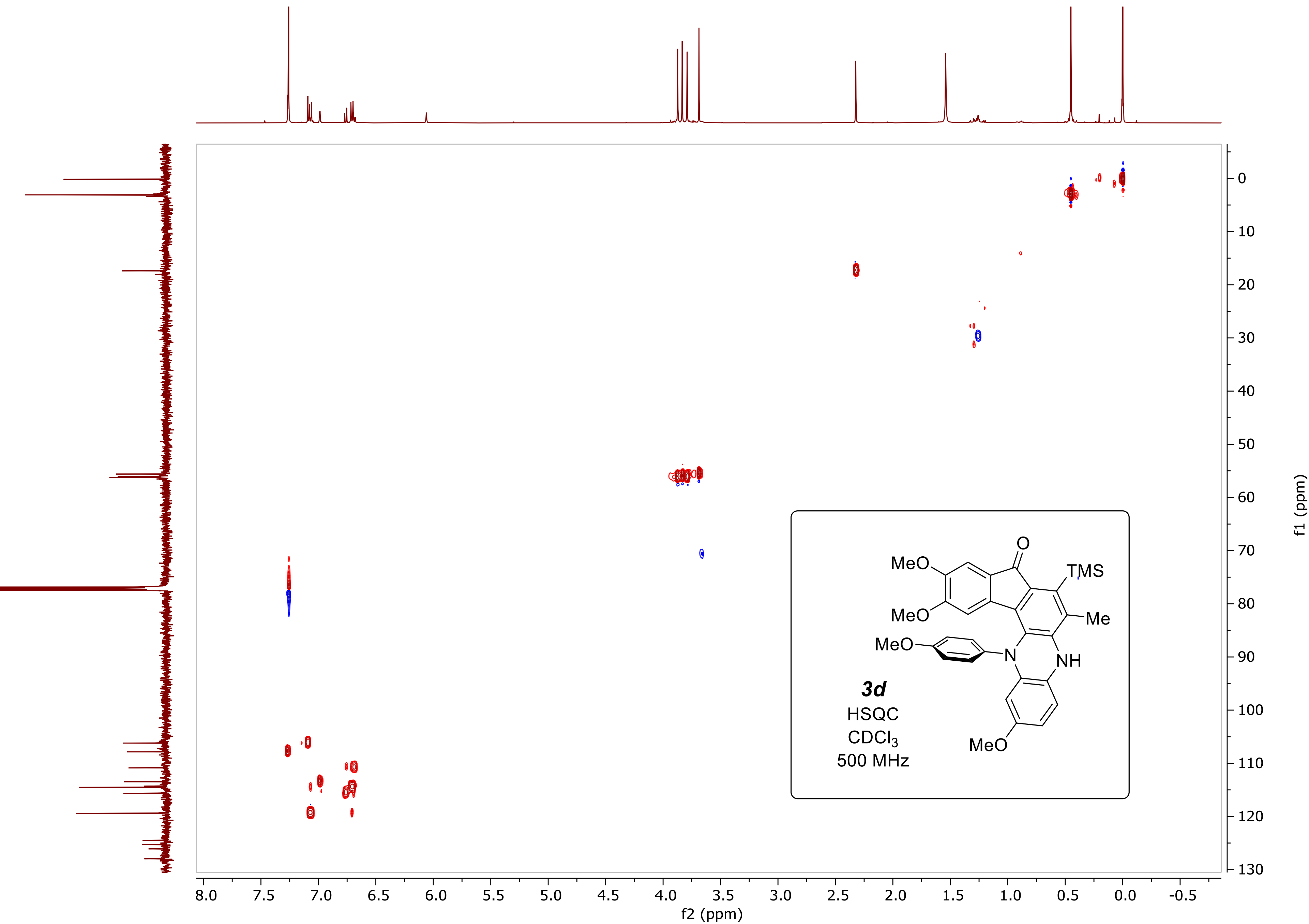

MeO
MeO
O
TMS
Me
MeO
N
NH
MeO
3d
HSQC
$CDCl_3$
500 MHz
f2 (ppm)
f1 (ppm)

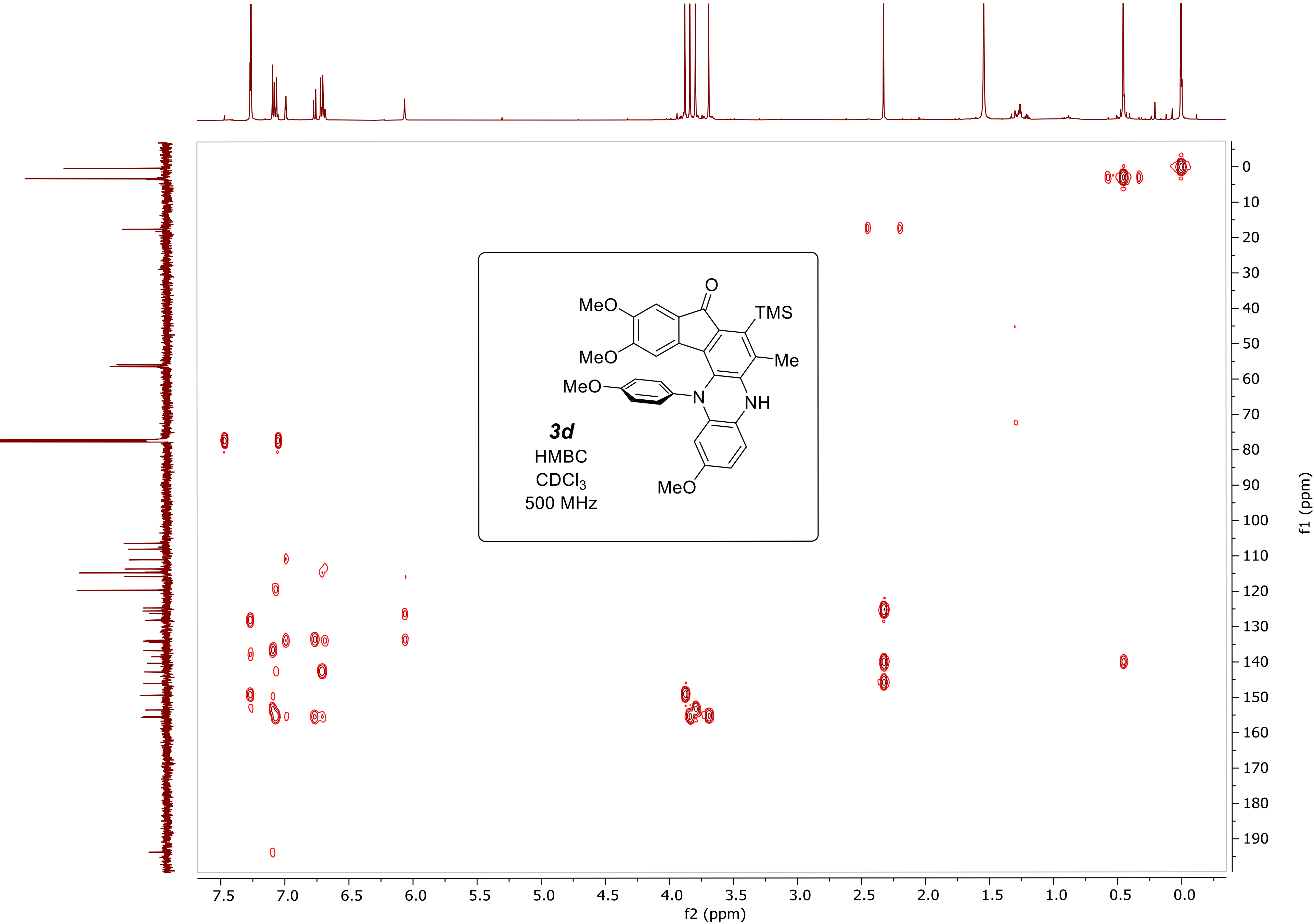
MeO
MeO
MeO
O
TMS
Me
N
NH
MeO
3d
HMBC
CDCl3
500 MHz
f2 (ppm)
f1 (ppm)

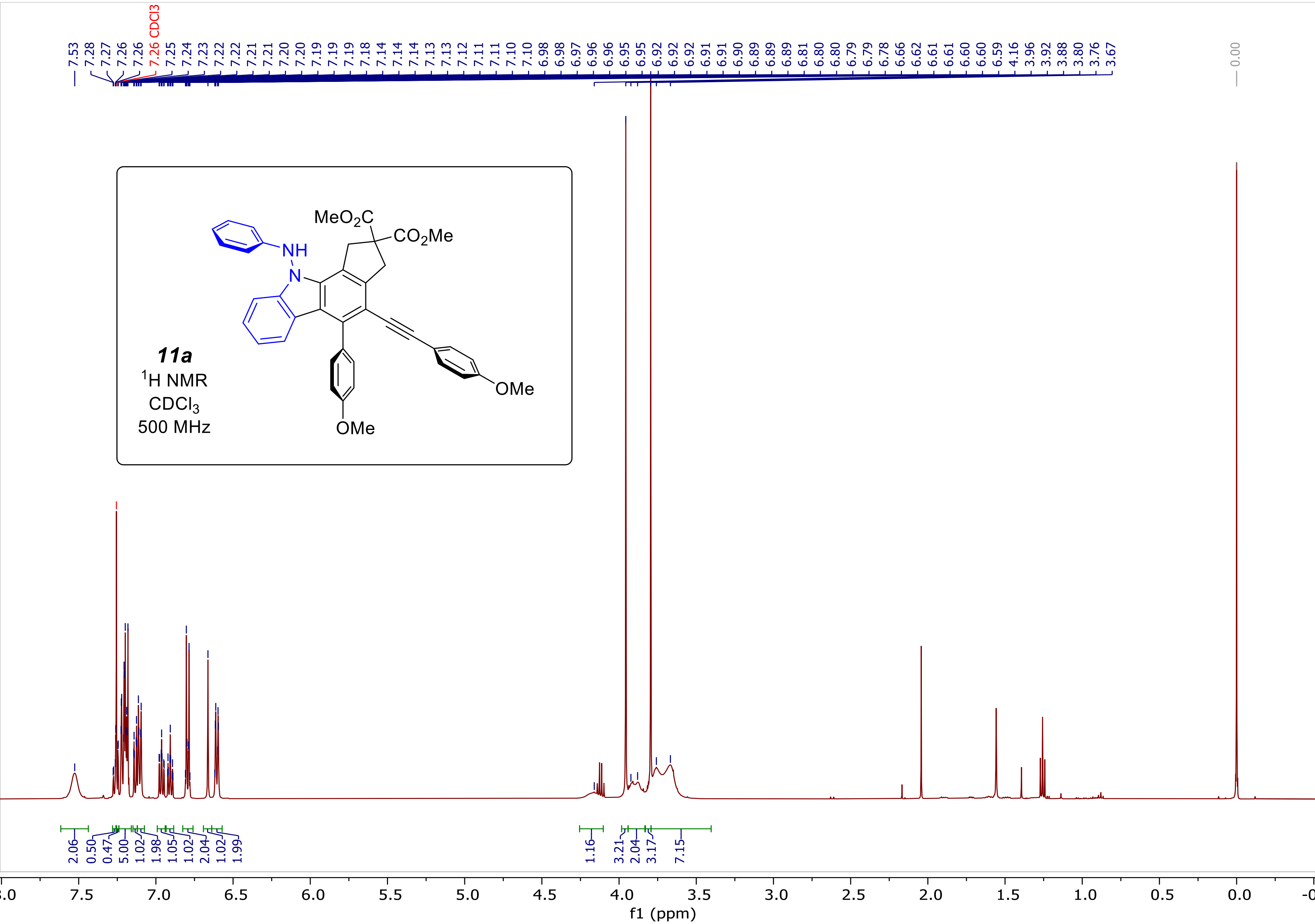
11a
^1H NMR
$CDCl_3$
500 MHz
f1 (ppm)

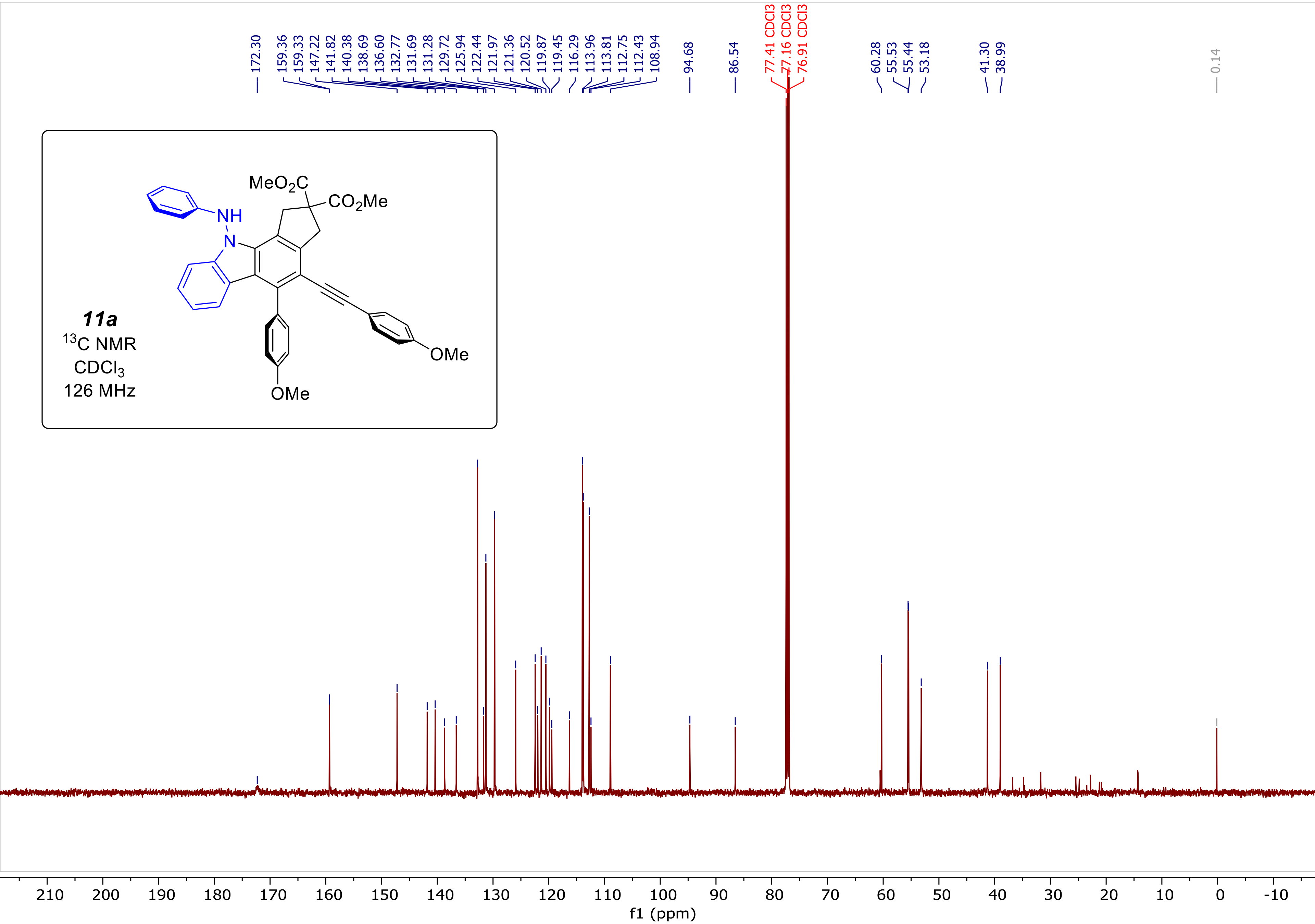
172.30
159.36
159.33
147.22
141.82
140.38
138.69
136.60
132.77
131.69
131.28
129.72
125.94
122.44
121.97
121.36
120.52
119.87
119.45
116.29
113.96
113.81
112.75
112.43
108.94
94.68
86.54
77.41 CDCl3
77.16 CDCl3
76.91 CDCl3
60.28
55.53
55.44
53.18
41.30
38.99
0.14
MeO2C
CO2Me
NH
N
OMe
OMe
11a
13C NMR
CDCl3
126 MHz
f1 (ppm)

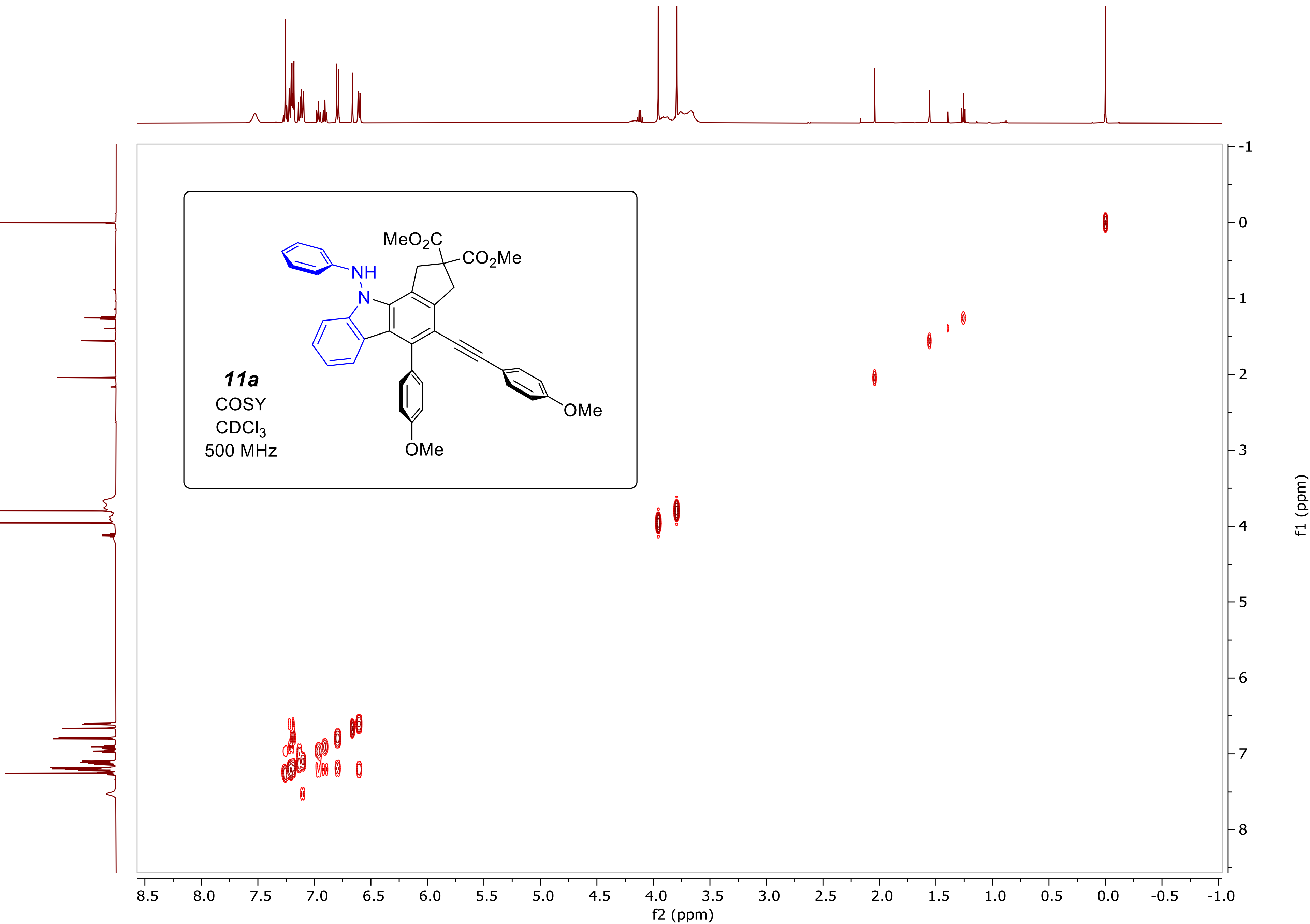
MeO2C
CO2Me
NH
N
OMe
OMe
11a
COSY
CDCl3
500 MHz
f2 (ppm)
f1 (ppm)

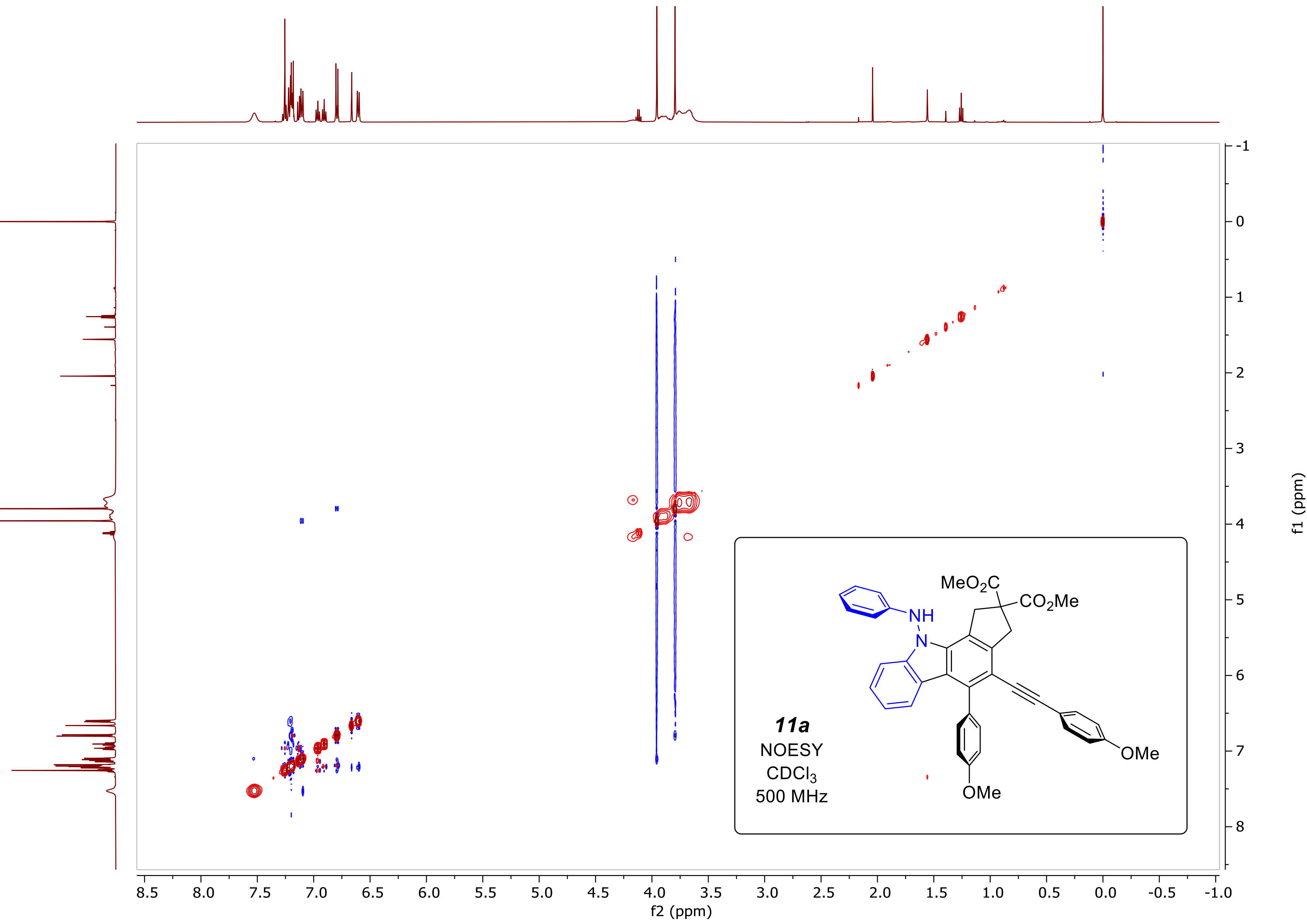

MeO2C
CO2Me
NH
N
OMe
OMe
11a
NOESY
CDCl3
500 MHz
f2 (ppm)
f1 (ppm)

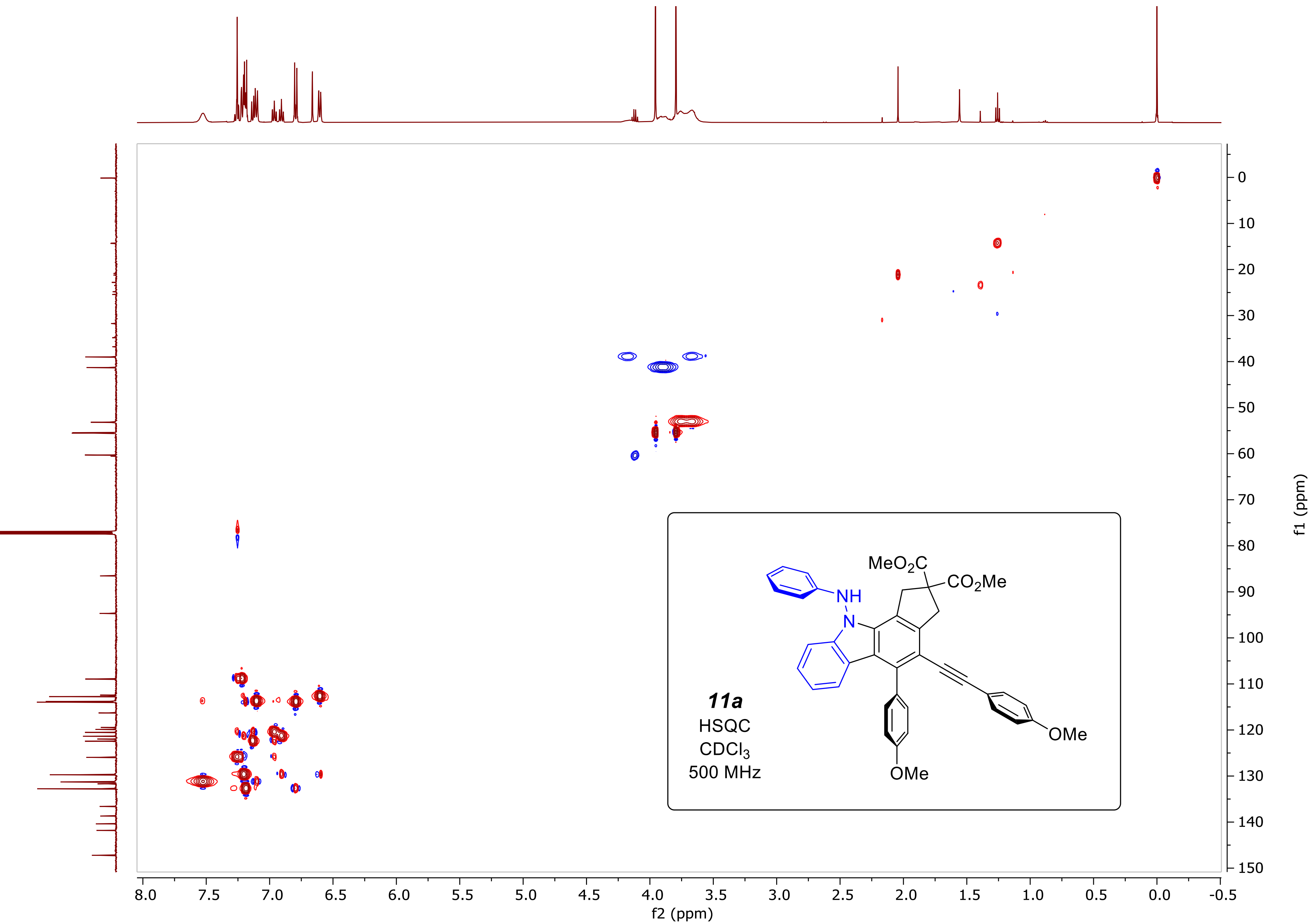
11a
HSQC
CDCl3
500 MHz
MeO2C
CO2Me
NH
N
OMe
OMe
f2 (ppm)
f1 (ppm)

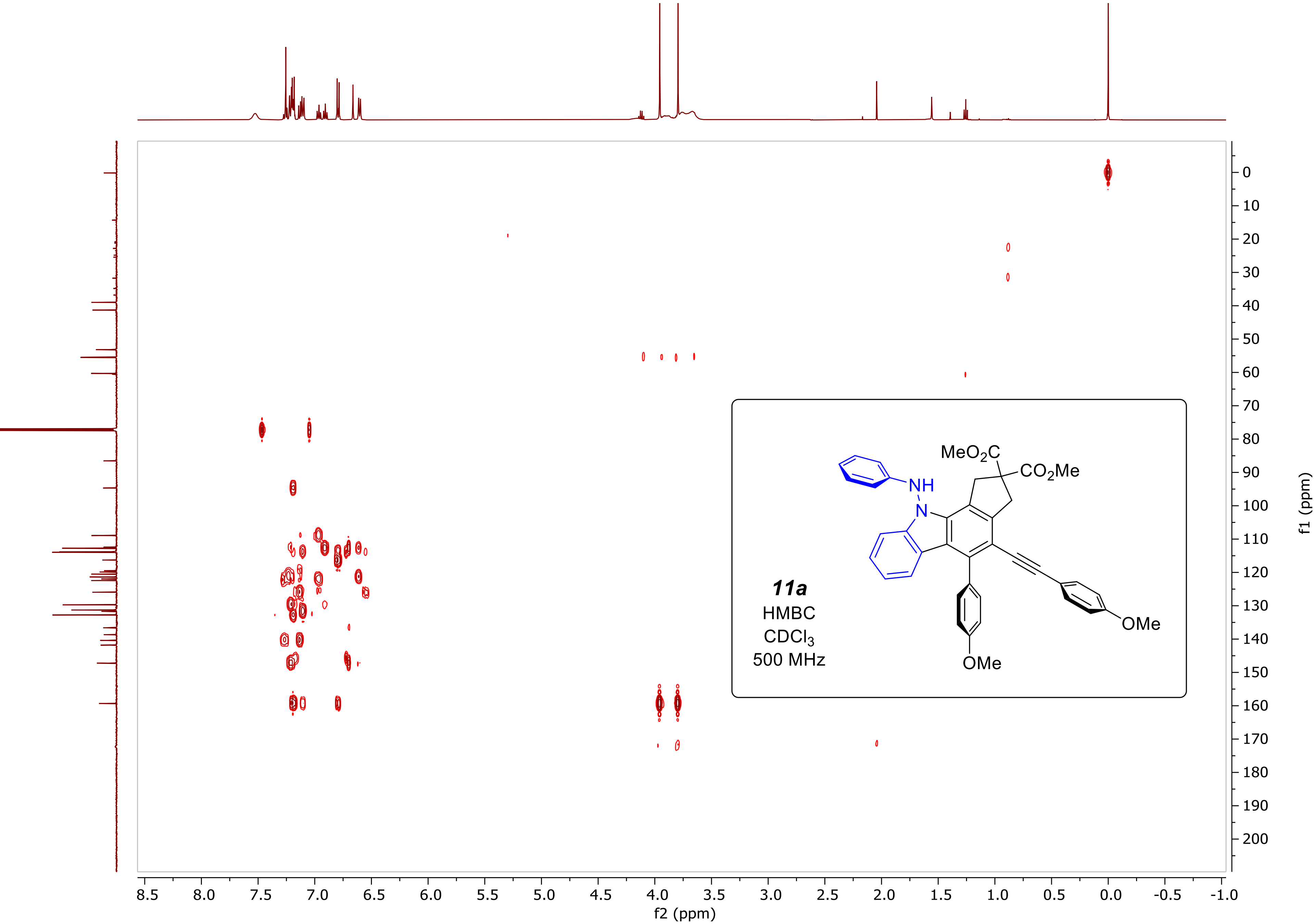
MeO2C
CO2Me
NH
N
OMe
OMe
11a
HMBC
CDCl3
500 MHz
f2 (ppm)
f1 (ppm)

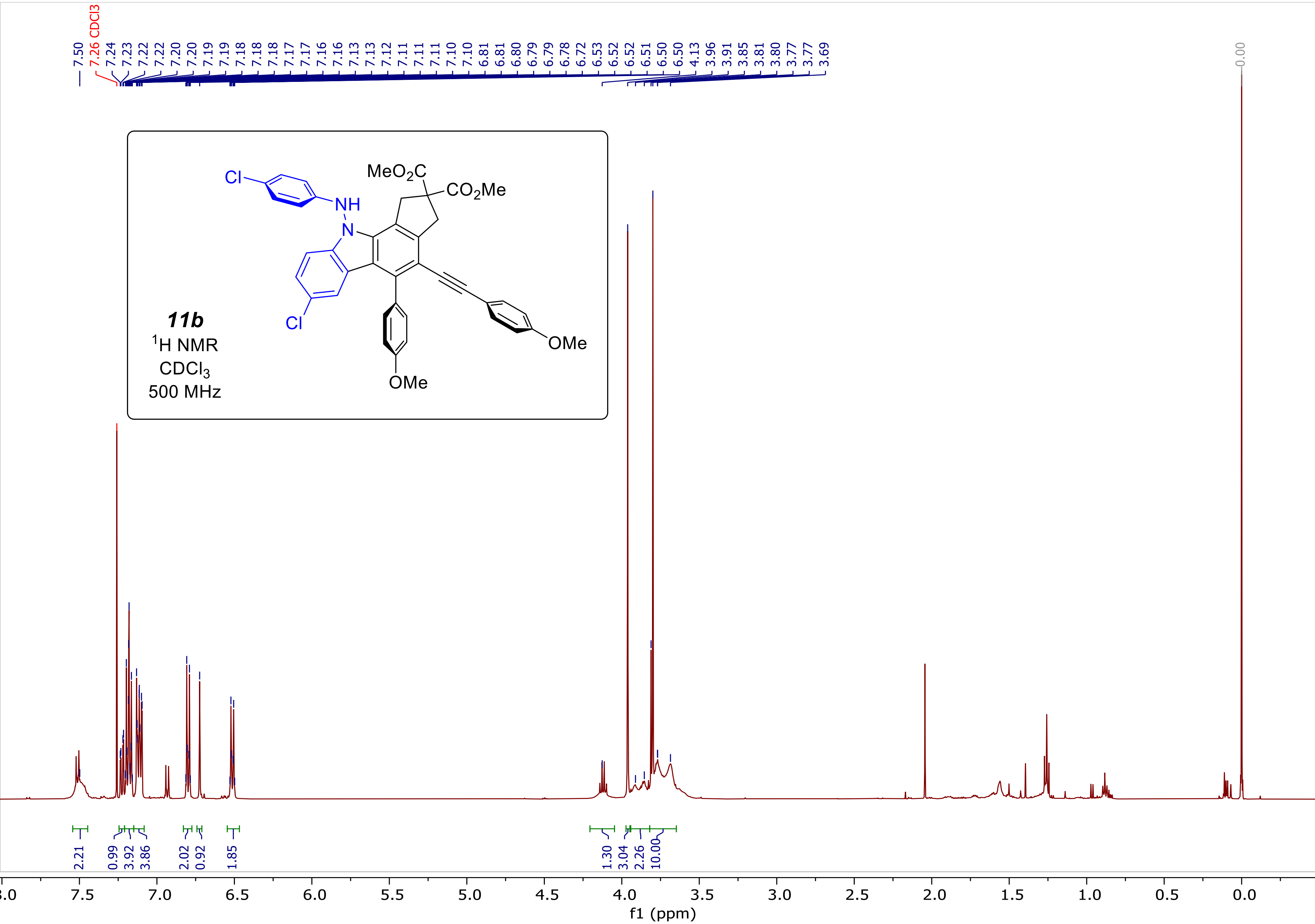

11b
1H NMR
CDCl3
500 MHz
f1 (ppm)

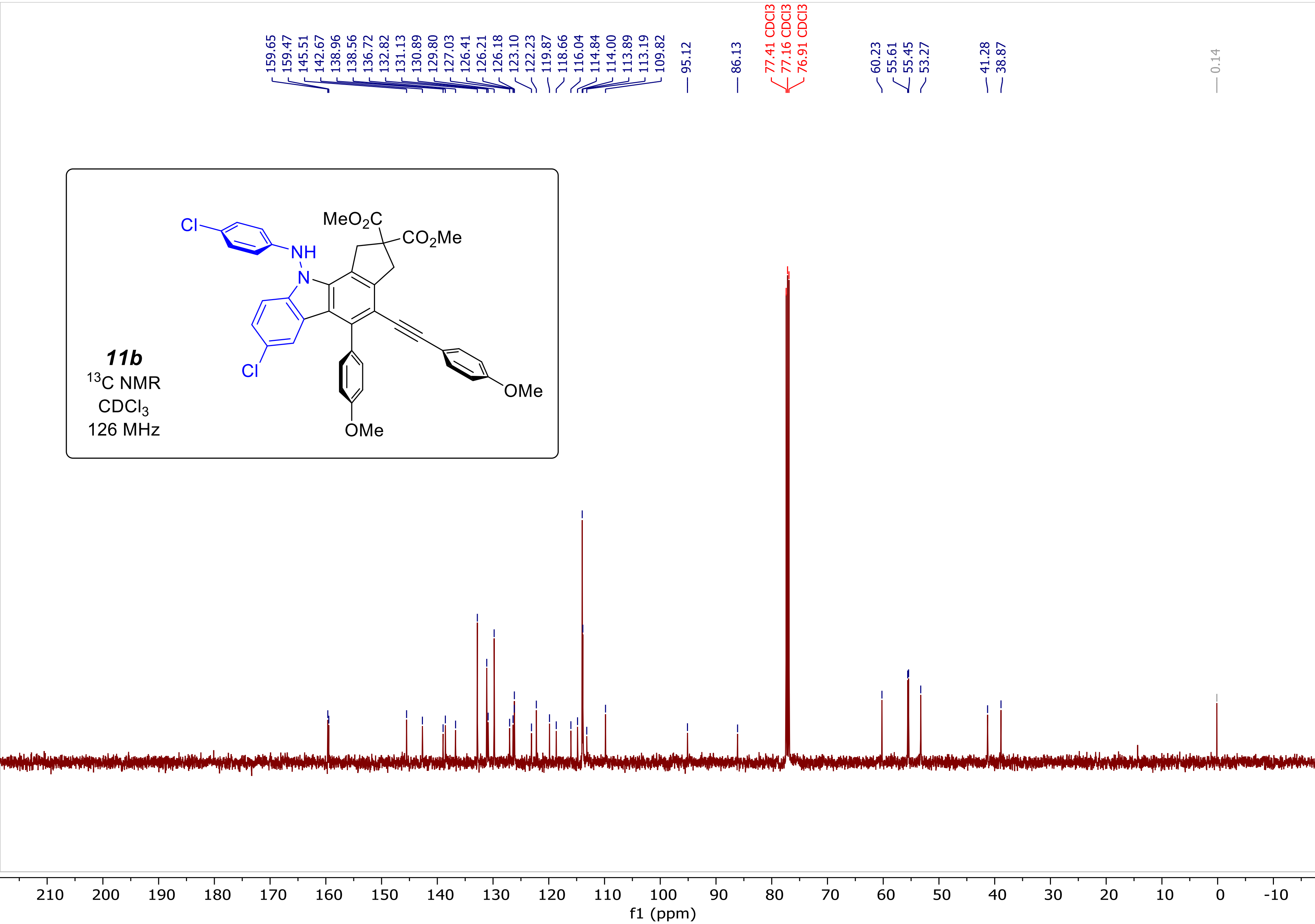
159.65
159.47
145.51
142.67
138.96
138.56
136.72
132.82
131.13
130.89
129.80
127.03
126.41
126.21
126.18
123.10
122.23
119.87
118.66
116.04
114.84
114.00
113.89
113.19
109.82
95.12
86.13
77.41 CDCl3
77.16 CDCl3
76.91 CDCl3
60.23
55.61
55.45
53.27
41.28
38.87
0.14
Cl
NH
N
Cl
MeO2C
CO2Me
OMe
OMe
11b
13C NMR
CDCl3
126 MHz
f1 (ppm)

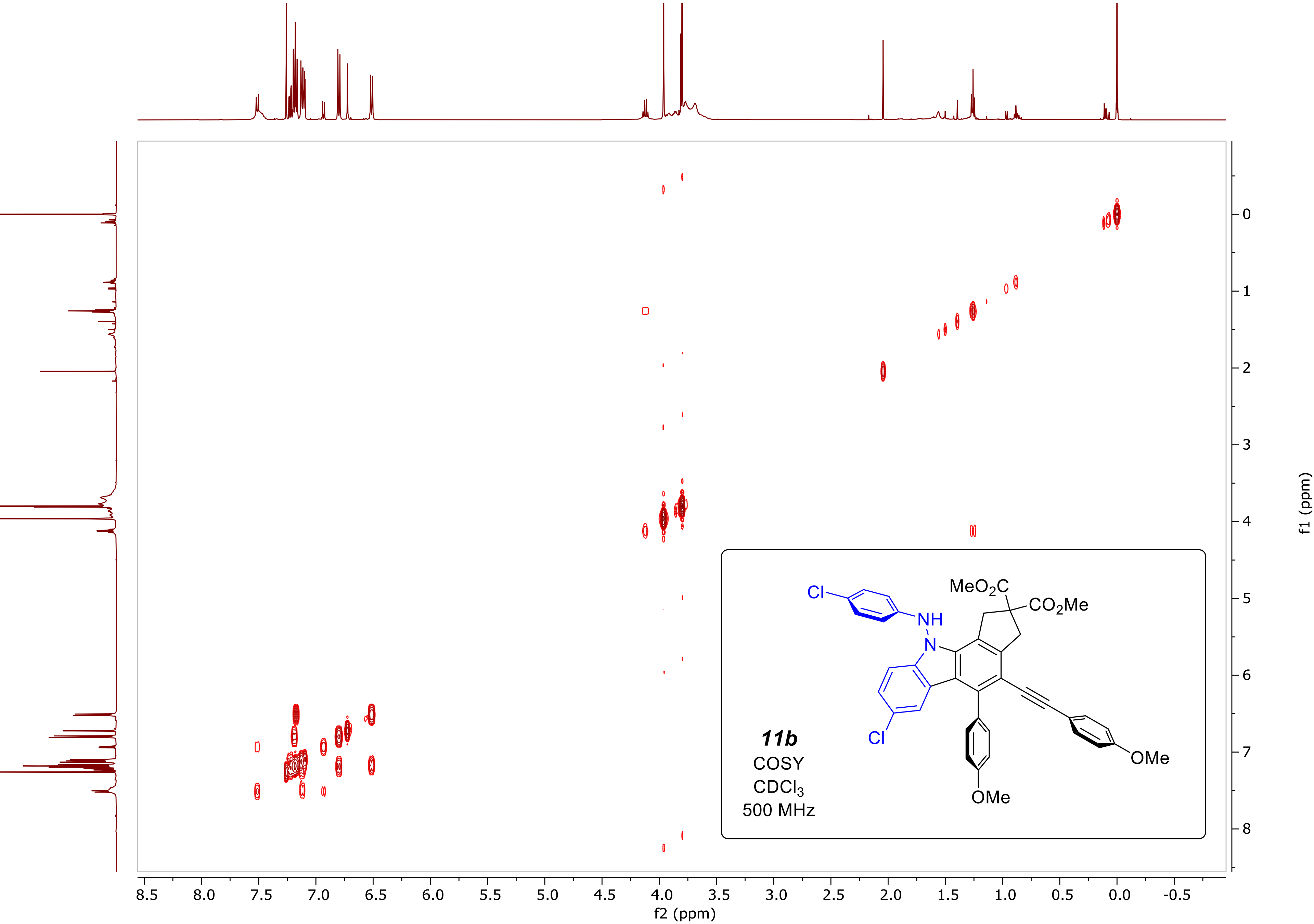

Cl
NH
N
Cl
MeO2C
CO2Me
OMe
OMe
11b
COSY
CDCl3
500 MHz
f2 (ppm)
f1 (ppm)

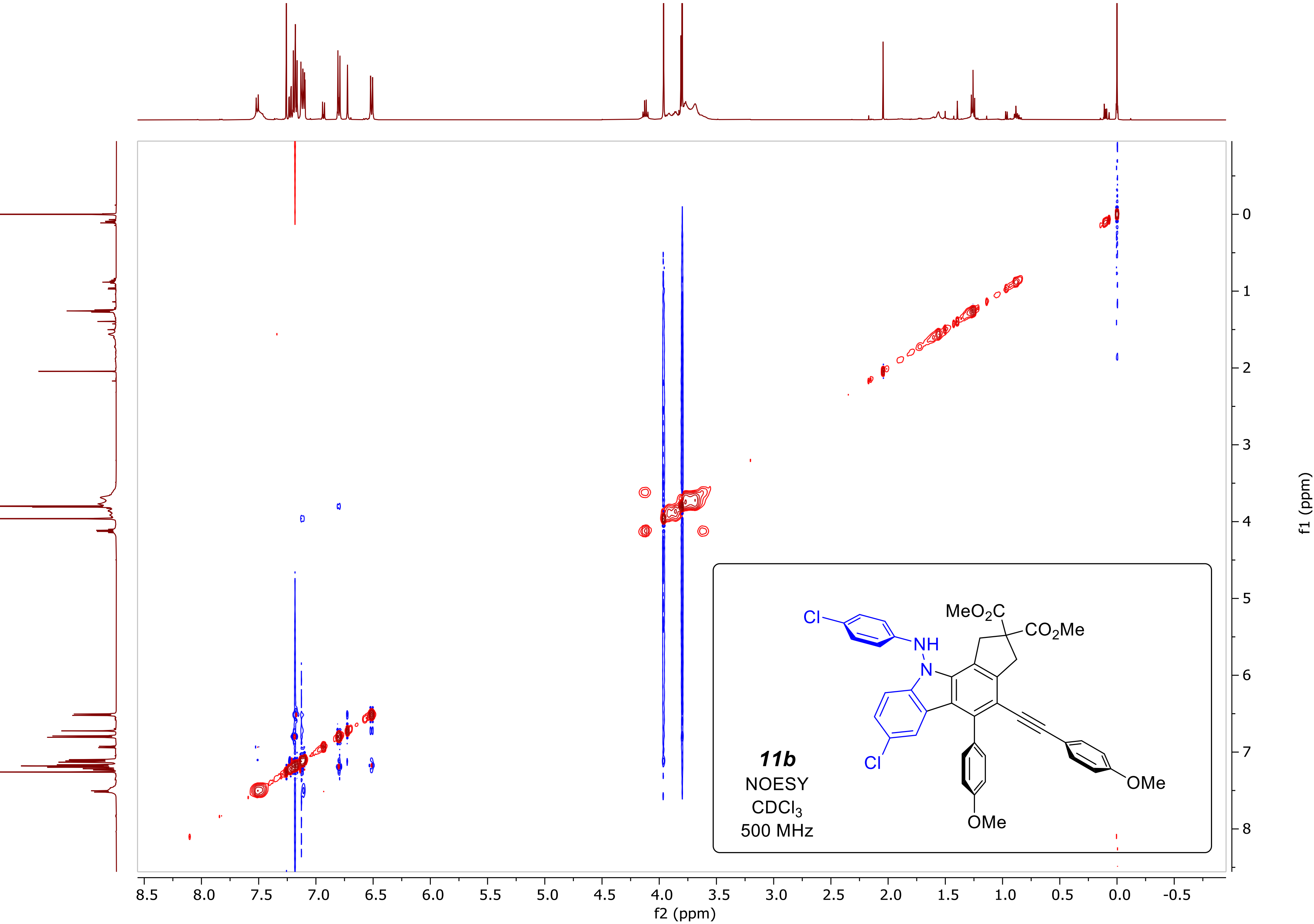

Cl
NH
N
MeO2C
CO2Me
Cl
OMe
OMe
11b
NOESY
CDCl3
500 MHz
f2 (ppm)
f1 (ppm)

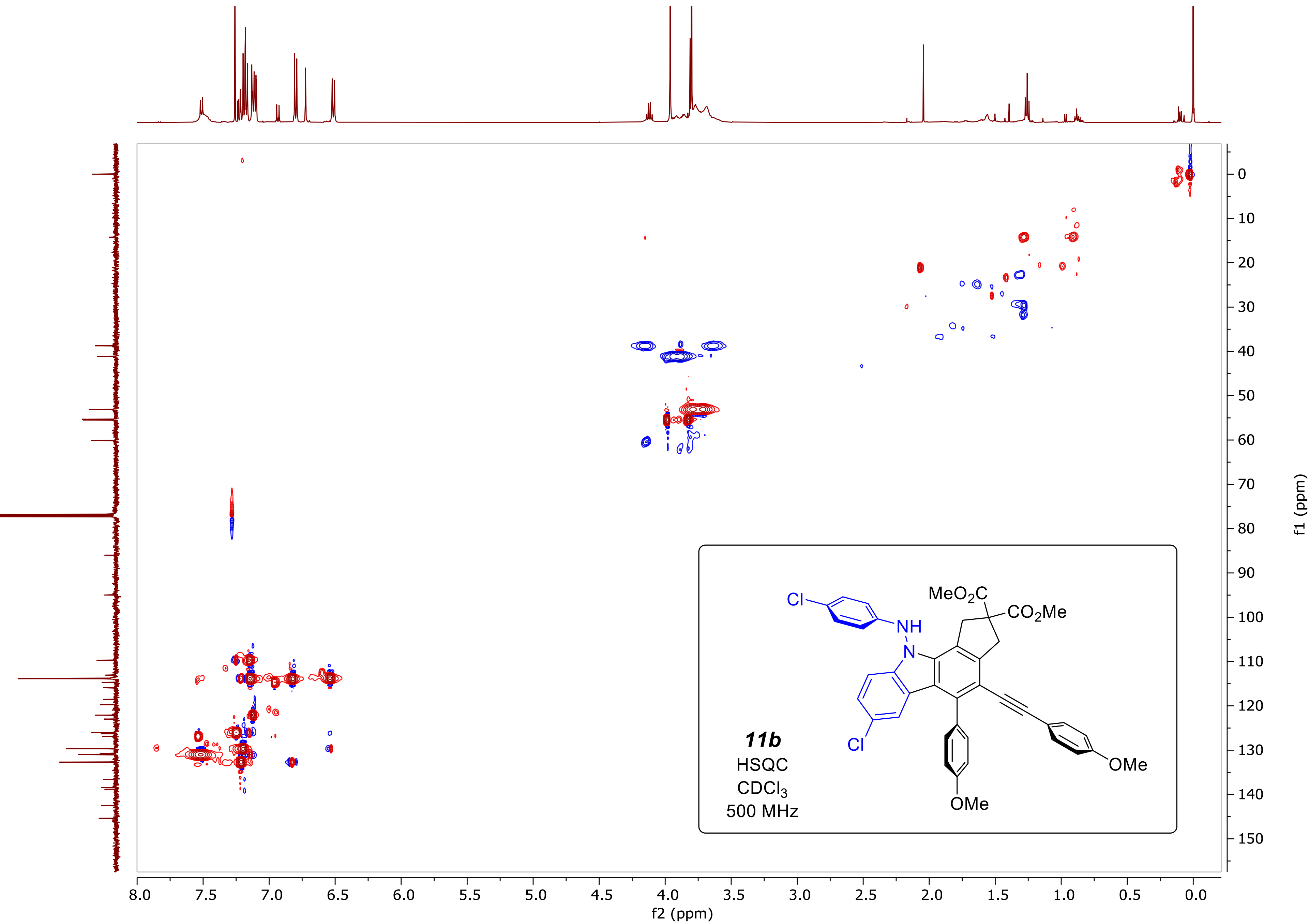

f2 (ppm)
f1 (ppm)
8.0
7.5
7.0
6.5
6.0
5.5
5.0
4.5
4.0
3.5
3.0
2.5
2.0
1.5
1.0
0.5
0.0
0
10
20
30
40
50
60
70
80
90
100
110
120
130
140
150
MeO2C
CO2Me
Cl
NH
N
Cl
OMe
OMe
11b
HSQC
CDCl3
500 MHz

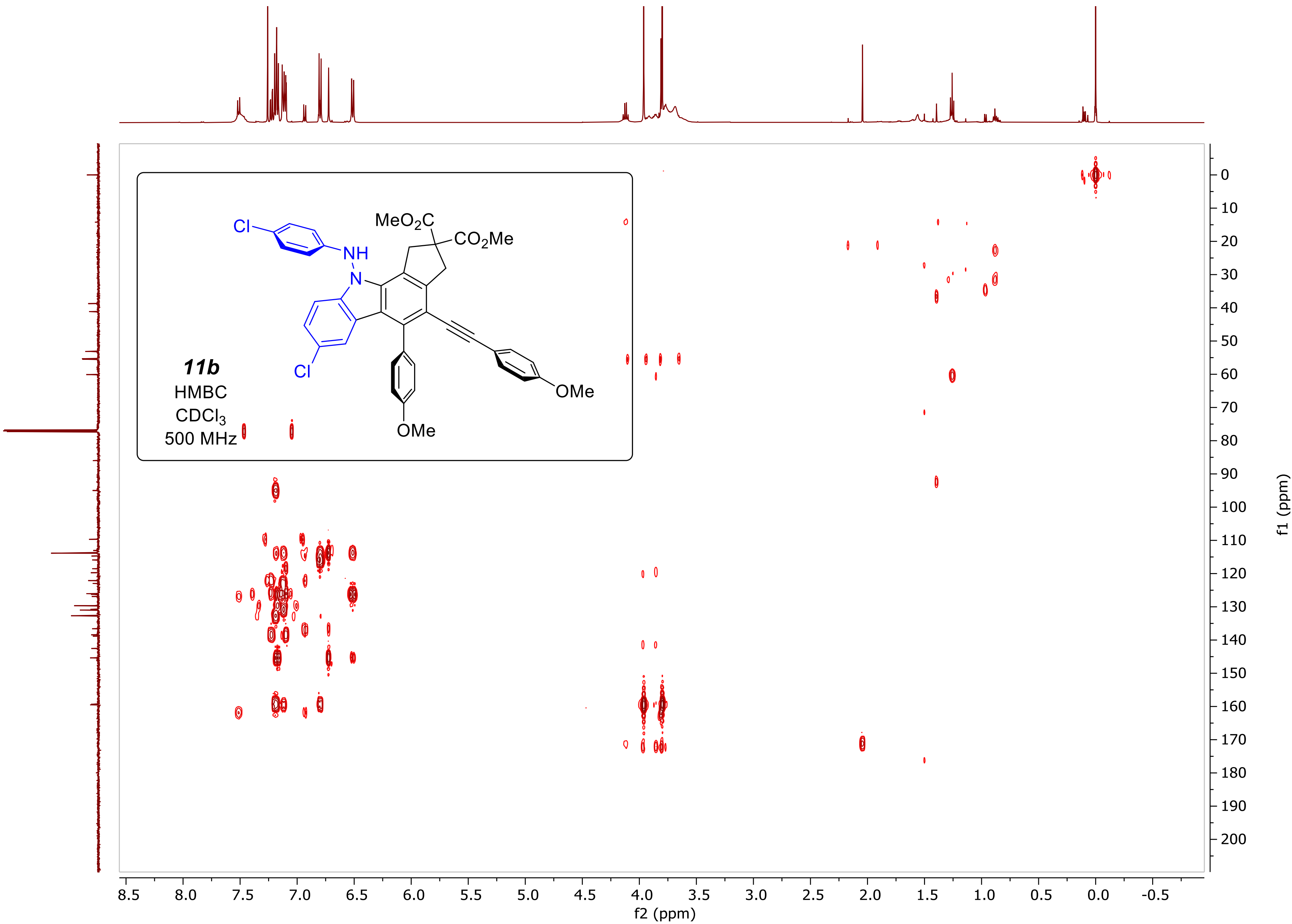
11b
HMBC
CDCl3
500 MHz
MeO2C
CO2Me
NH
N
Cl
Cl
OMe
OMe
f2 (ppm)
f1 (ppm)

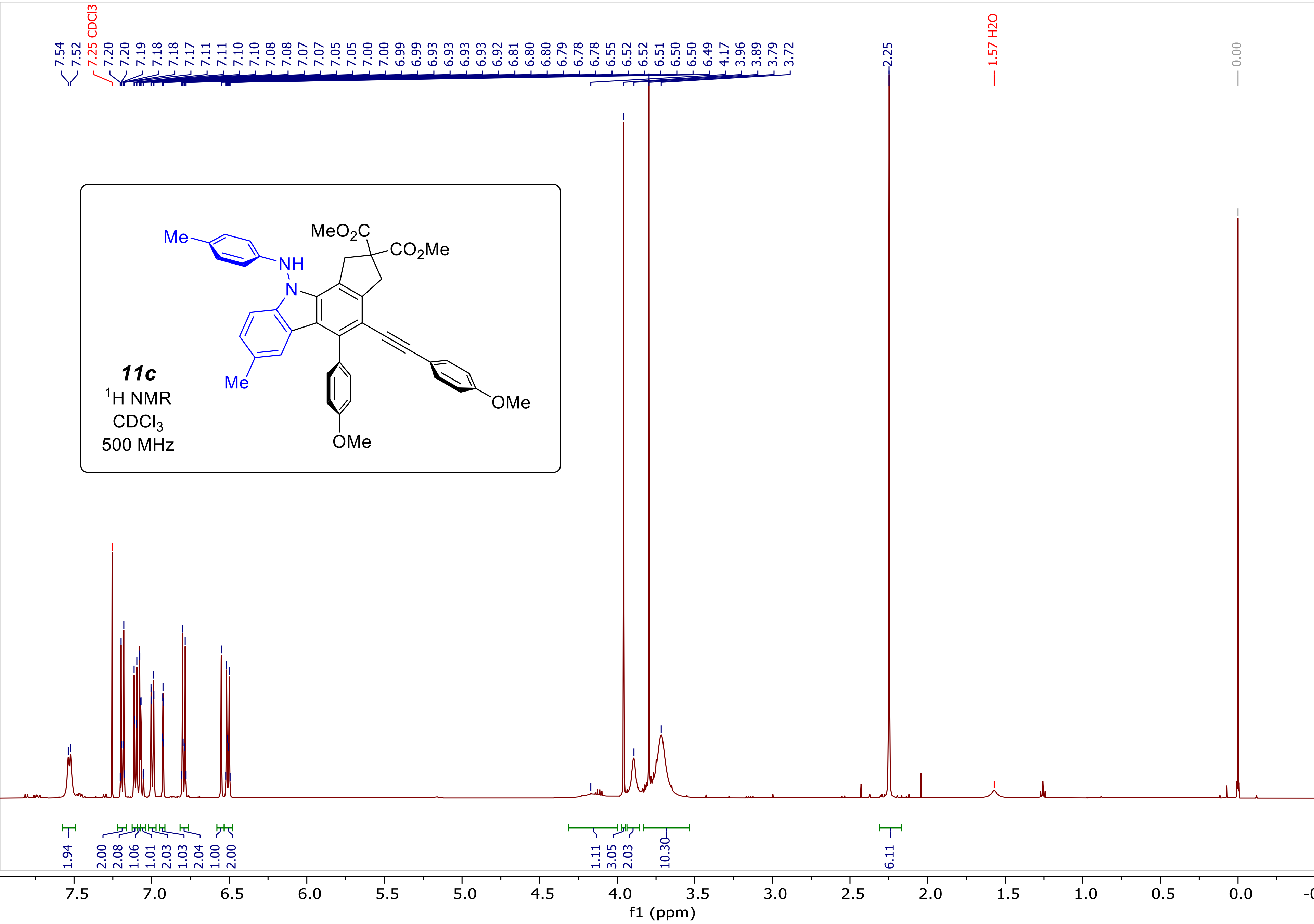
7.54
7.52
7.25 CDCl3
7.20
7.20
7.19
7.18
7.18
7.17
7.11
7.11
7.10
7.10
7.08
7.08
7.07
7.07
7.05
7.05
7.00
7.00
6.99
6.99
6.93
6.93
6.93
6.93
6.92
6.81
6.80
6.80
6.79
6.78
6.78
6.55
6.52
6.52
6.51
6.50
6.50
6.49
4.17
3.96
3.89
3.79
3.72
2.25
1.57 H2O
0.00
Me
NH
N
Me
MeO2C
CO2Me
OMe
OMe
11c
1H NMR
CDCl3
500 MHz
1.94
2.00
2.08
1.06
1.01
2.03
1.03
2.04
1.00
2.00
1.11
3.05
2.03
10.30
6.11
7.5
7.0
6.5
6.0
5.5
5.0
4.5
4.0
3.5
3.0
2.5
2.0
1.5
1.0
0.5
0.0
f1 (ppm)

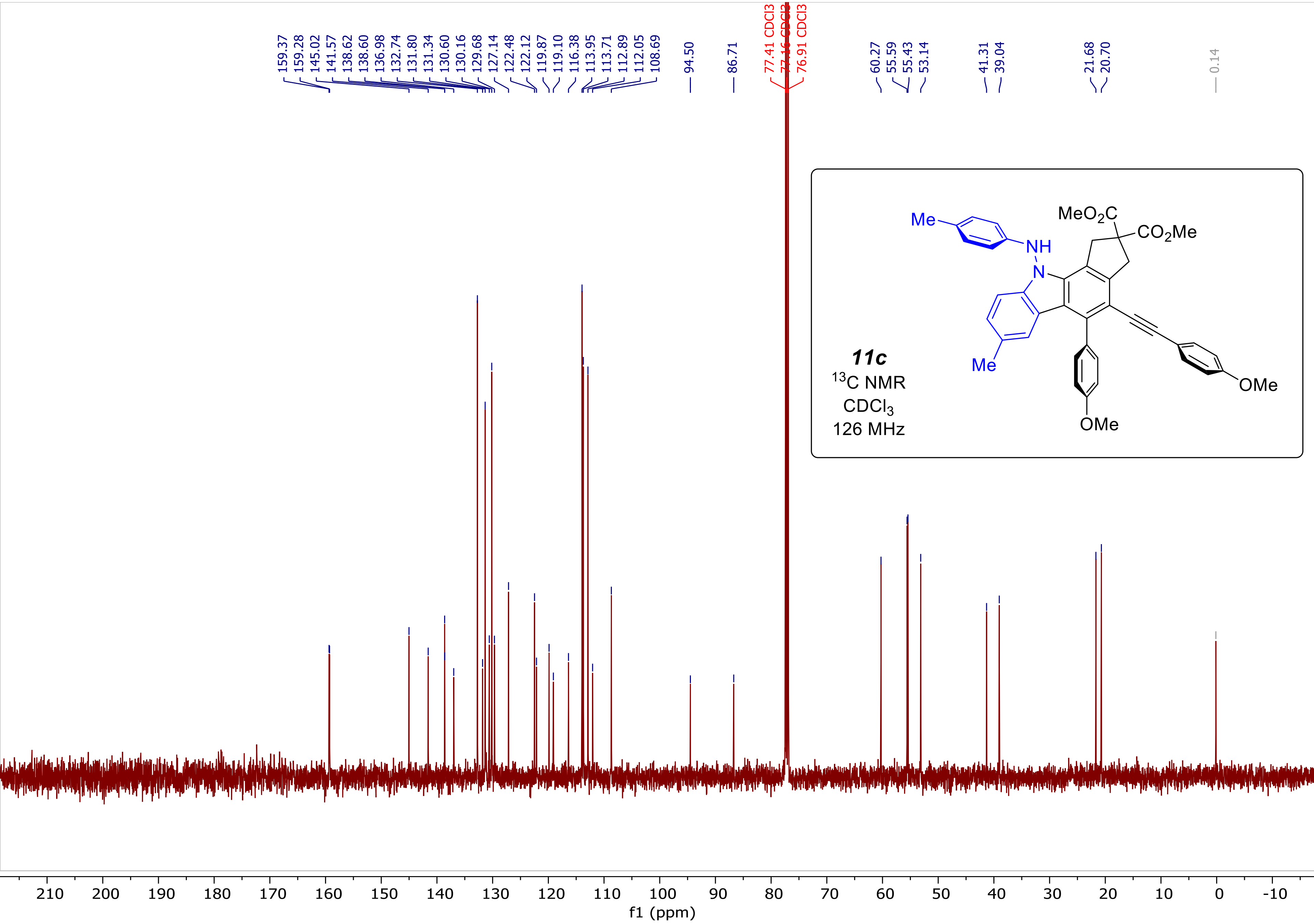
159.37
159.28
145.02
141.57
138.62
138.60
136.98
132.74
131.80
131.34
130.60
130.16
129.68
127.14
122.48
122.12
119.87
119.10
116.38
113.95
113.71
112.89
112.05
108.69
94.50
86.71
77.41 CDCl3
77.16 CDCl3
76.91 CDCl3
60.27
55.59
55.43
53.14
41.31
39.04
21.68
20.70
0.14
Me
NH
N
Me
MeO_2C
CO_2Me
OMe
OMe
11c
^{13}C NMR
$CDCl_3$
126 MHz
210 200 190 180 170 160 150 140 130 120 110 100 90 80 70 60 50 40 30 20 10 0 -10
f1 (ppm)

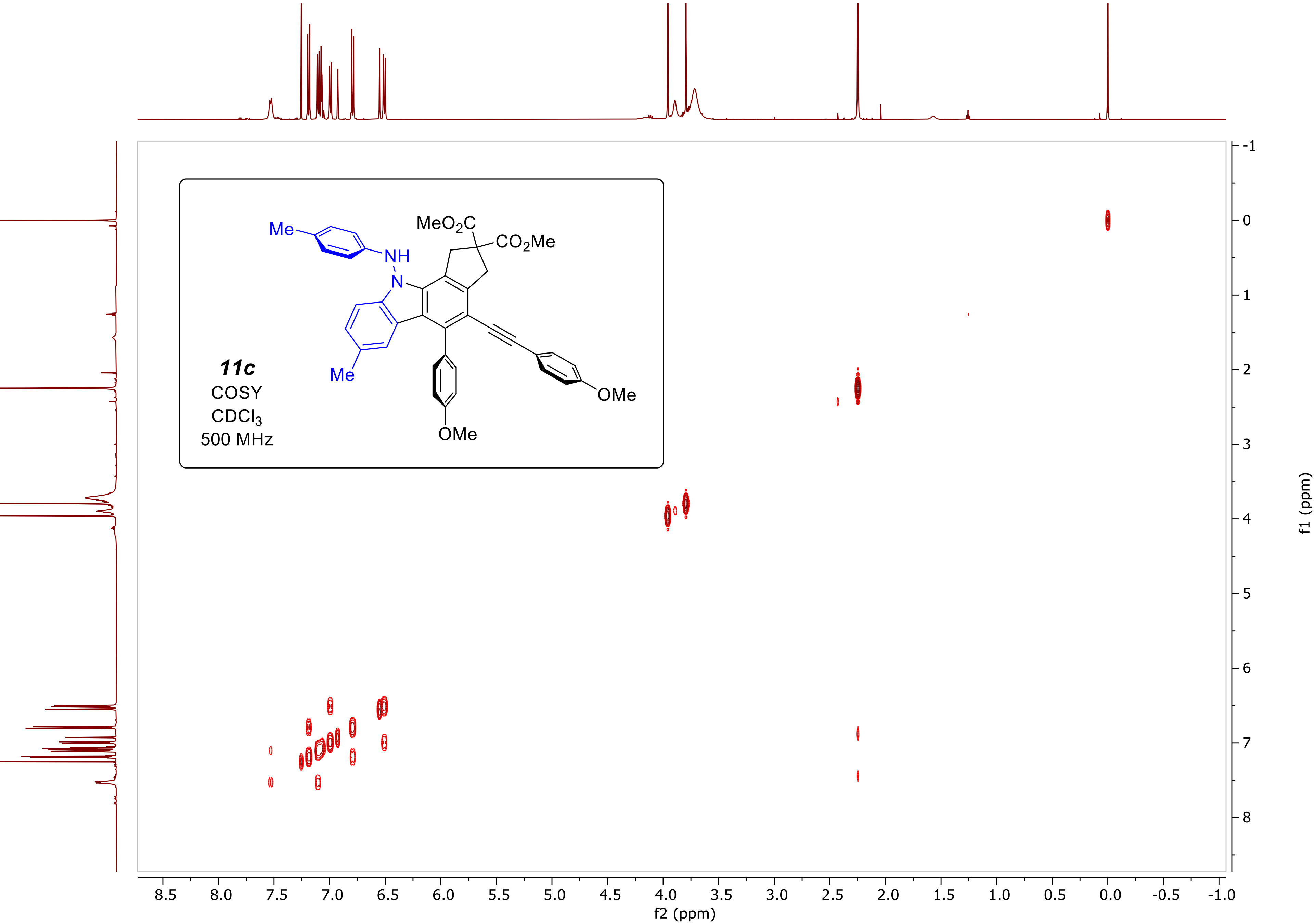

Me
NH
N
MeO2C
CO2Me
Me
OMe
OMe
11c
COSY
CDCl3
500 MHz
f2 (ppm)
f1 (ppm)

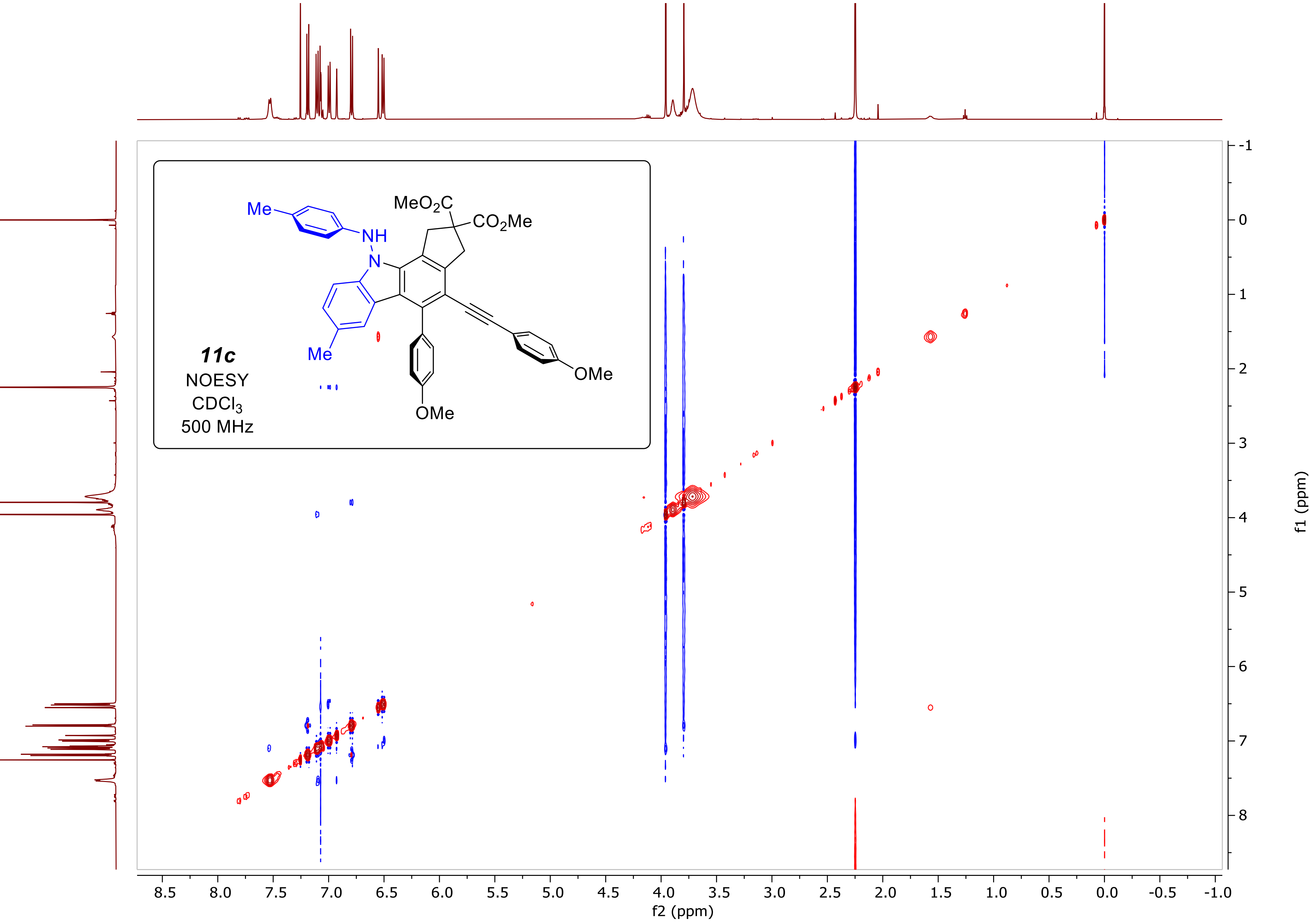

MeO2C
CO2Me
Me
NH
N
Me
OMe
OMe
11c
NOESY
CDCl3
500 MHz
f2 (ppm)
f1 (ppm)

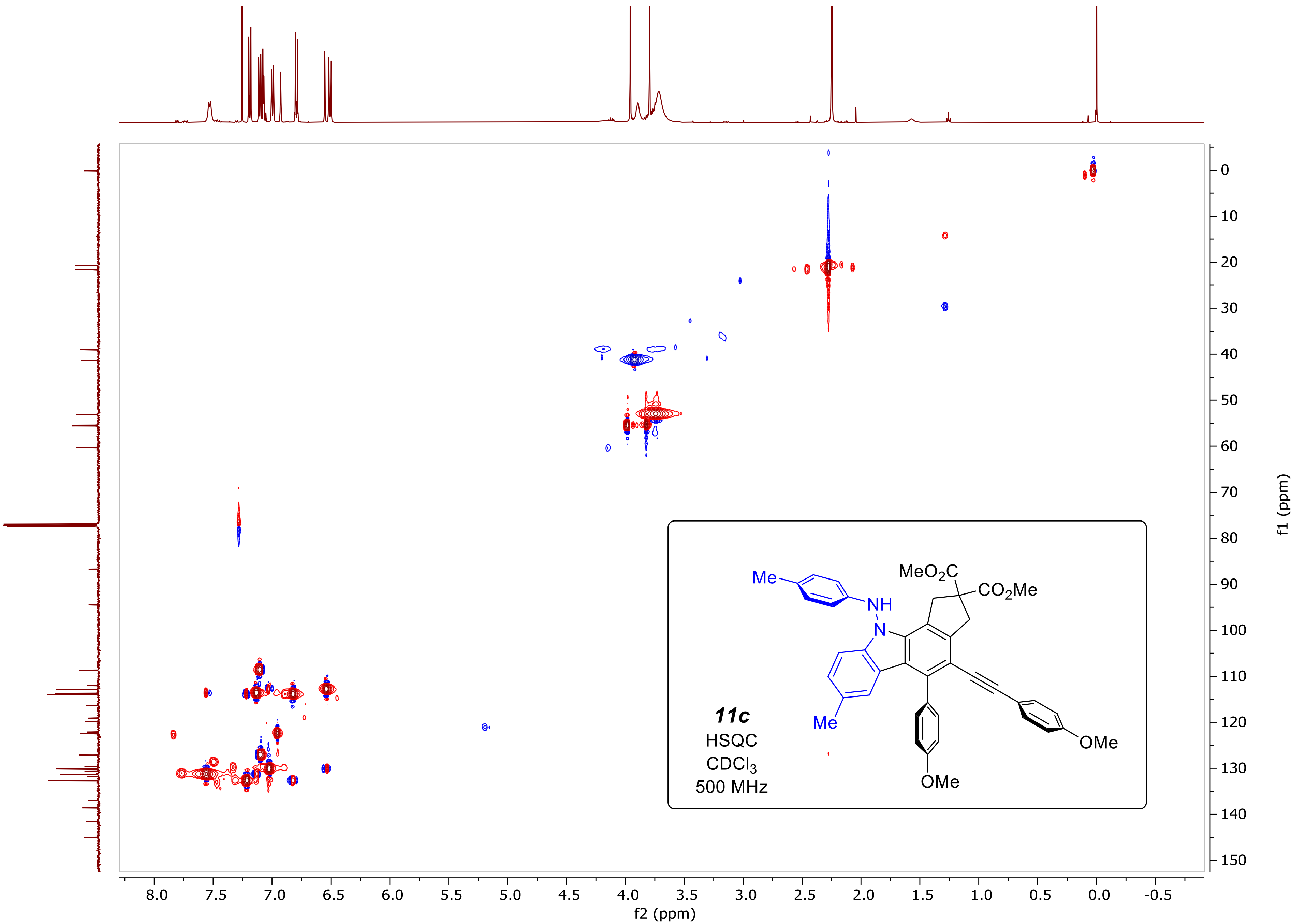
MeO2C
CO2Me
Me
NH
N
Me
OMe
OMe
11c
HSQC
CDCl3
500 MHz
f2 (ppm)
f1 (ppm)
8.0
7.5
7.0
6.5
6.0
5.5
5.0
4.5
4.0
3.5
3.0
2.5
2.0
1.5
1.0
0.5
0.0
-0.5
0
10
20
30
40
50
60
70
80
90
100
110
120
130
140
150

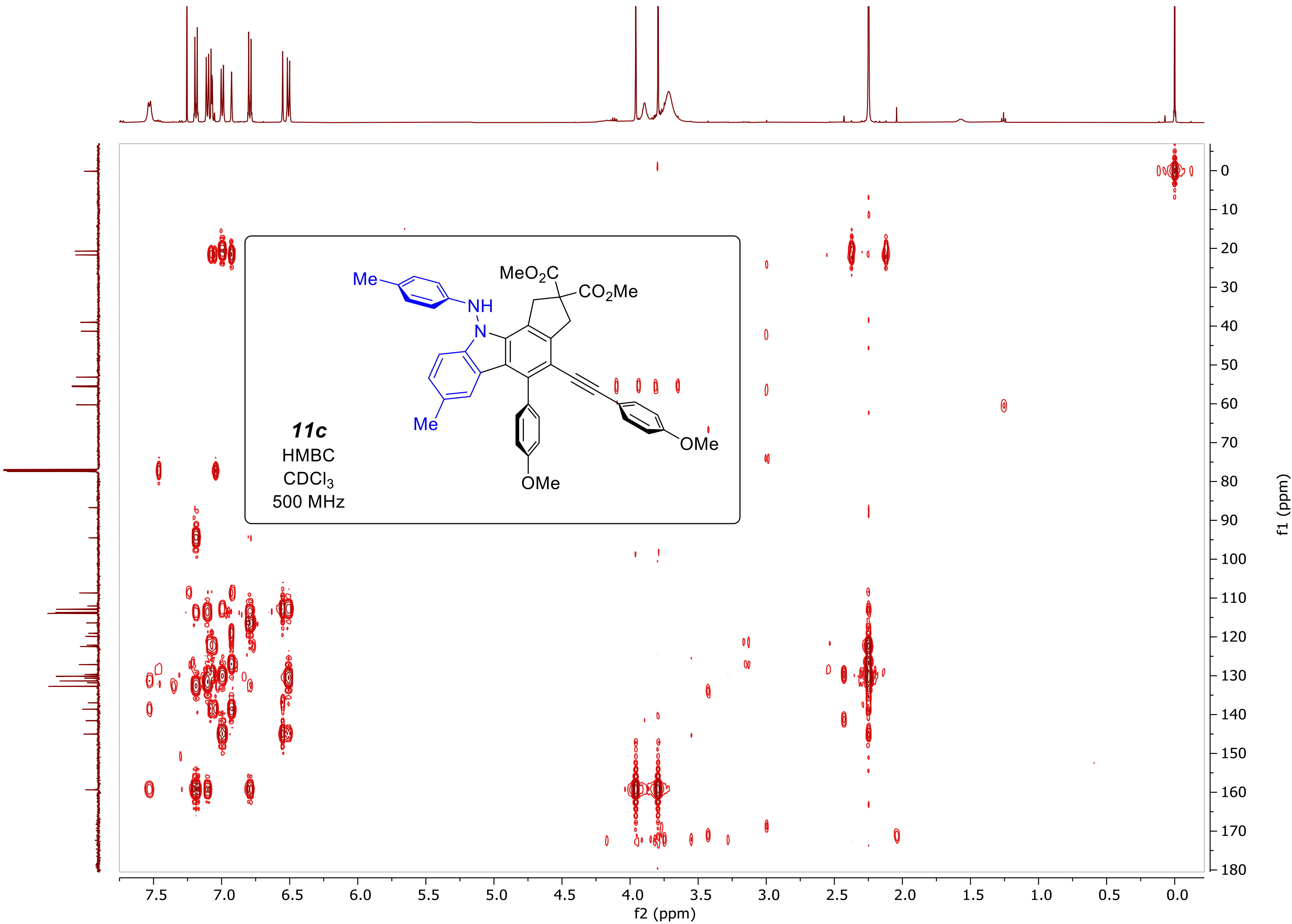
MeO2C
CO2Me
Me
NH
N
Me
OMe
OMe
11c
HMBC
CDCl3
500 MHz
f2 (ppm)
f1 (ppm)

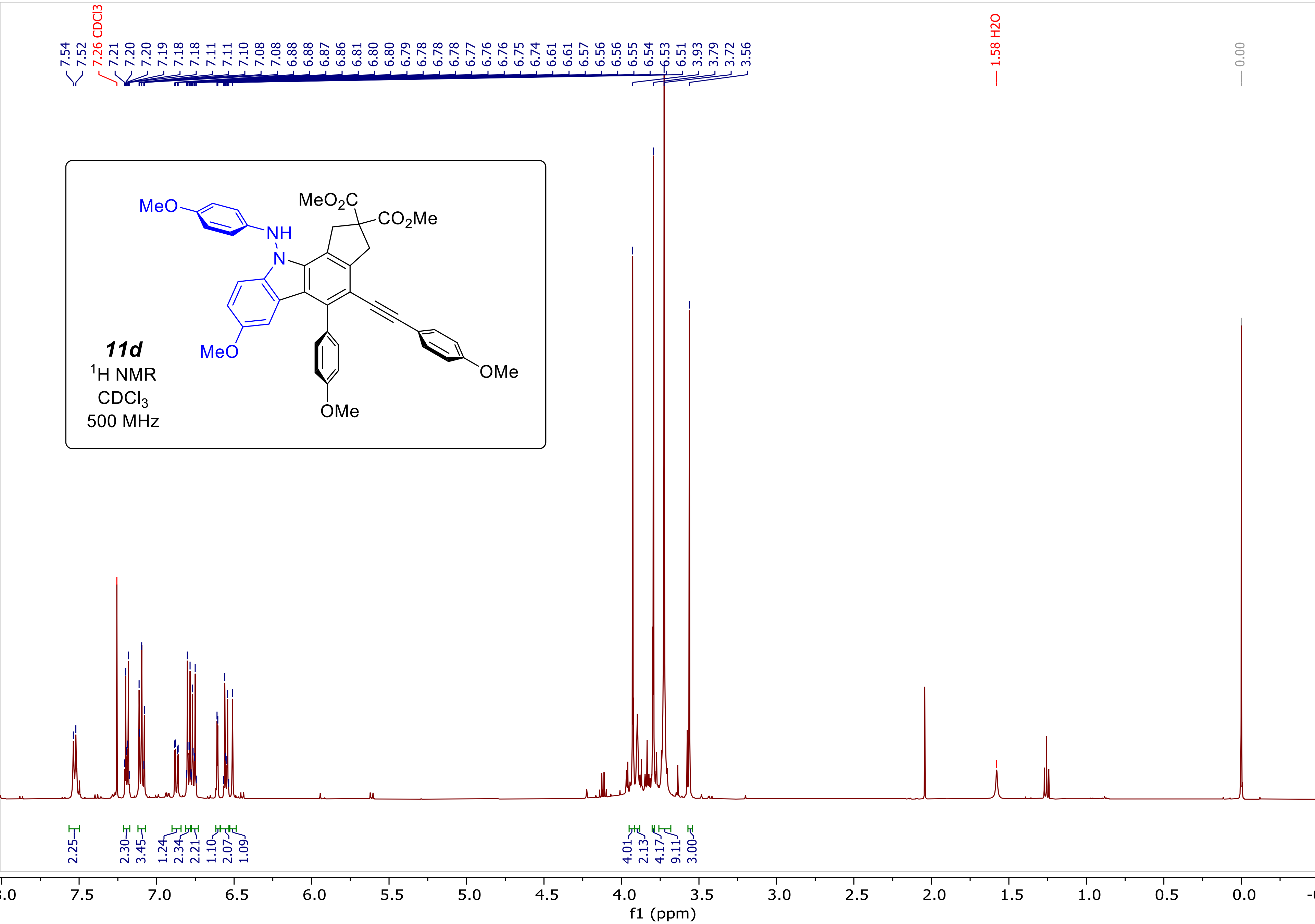
7.54
7.52
7.26 CDCl3
7.21
7.20
7.19
7.18
7.18
7.11
7.11
7.10
7.08
7.08
6.88
6.88
6.87
6.86
6.81
6.80
6.80
6.79
6.78
6.78
6.78
6.77
6.76
6.76
6.75
6.74
6.61
6.61
6.57
6.56
6.56
6.55
6.54
6.53
6.51
3.93
3.79
3.72
3.56
1.58 H2O
0.00
MeO
NH
N
MeO
MeO2C
CO2Me
OMe
OMe
11d
1H NMR
CDCl3
500 MHz
2.25
2.30
3.45
1.24
2.34
2.21
1.10
2.07
1.09
4.01
2.13
4.17
9.11
3.00
f1 (ppm)

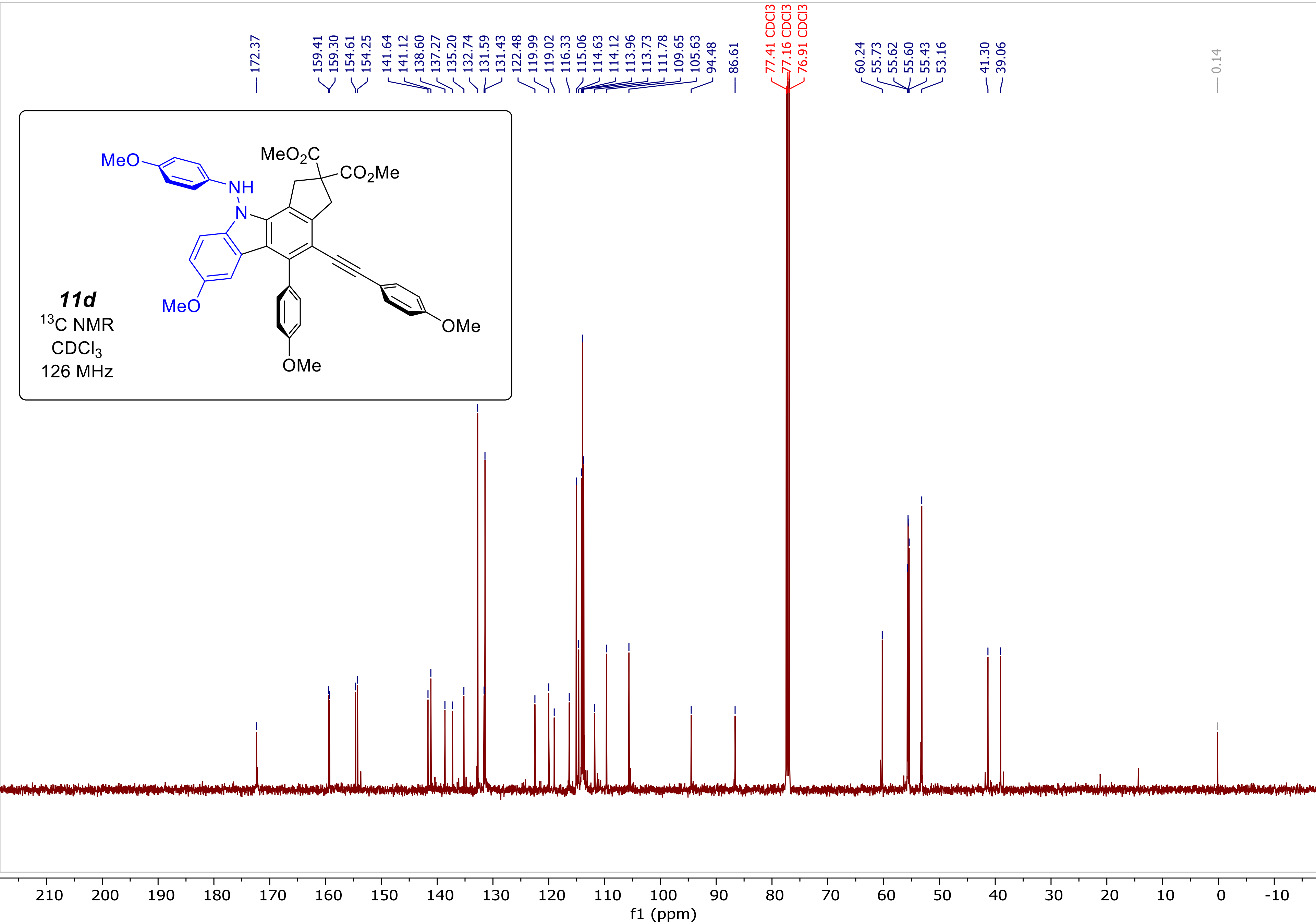
11d
13C NMR
CDCl3
126 MHz
MeO
NH
N
MeO
MeO2C
CO2Me
OMe
OMe
172.37
159.41
159.30
154.61
154.25
141.64
141.12
138.60
137.27
135.20
132.74
131.59
131.43
122.48
119.99
119.02
116.33
115.06
114.63
114.12
113.96
113.73
111.78
109.65
105.63
94.48
86.61
77.41 CDCl3
77.16 CDCl3
76.91 CDCl3
60.24
55.73
55.62
55.60
55.43
53.16
41.30
39.06
0.14
210
200
190
180
170
160
150
140
130
120
110
100
90
80
70
60
50
40
30
20
10
0
-10
f1 (ppm)

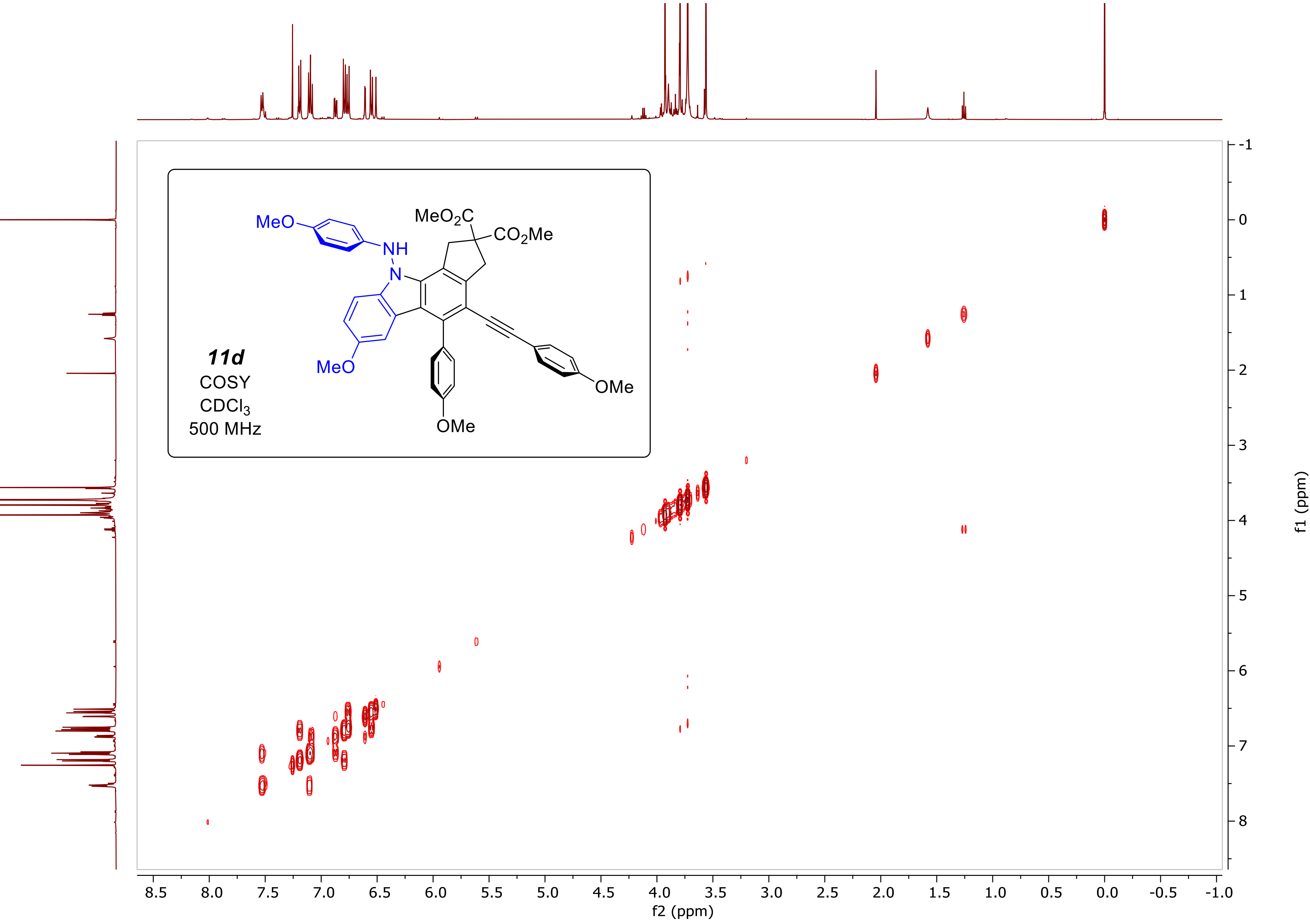
MeO
NH
N
MeO$_2$C
CO$_2$Me
MeO
OMe
OMe
11d
COSY
CDCl$_3$
500 MHz
f2 (ppm)
f1 (ppm)

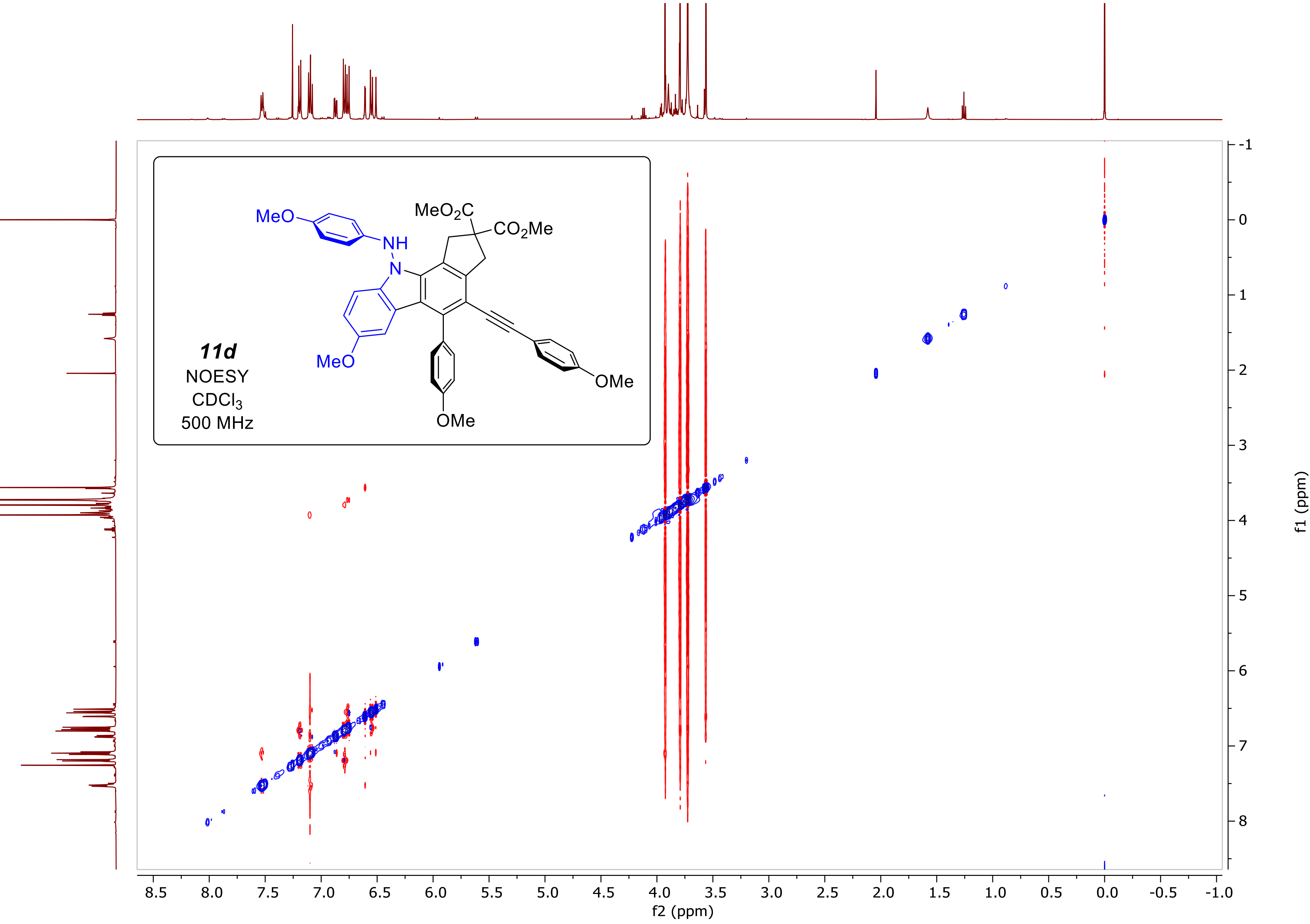
MeO
NH
N
MeO₂C
CO₂Me
MeO
OMe
OMe
11d
NOESY
CDCl₃
500 MHz
f2 (ppm)
f1 (ppm)

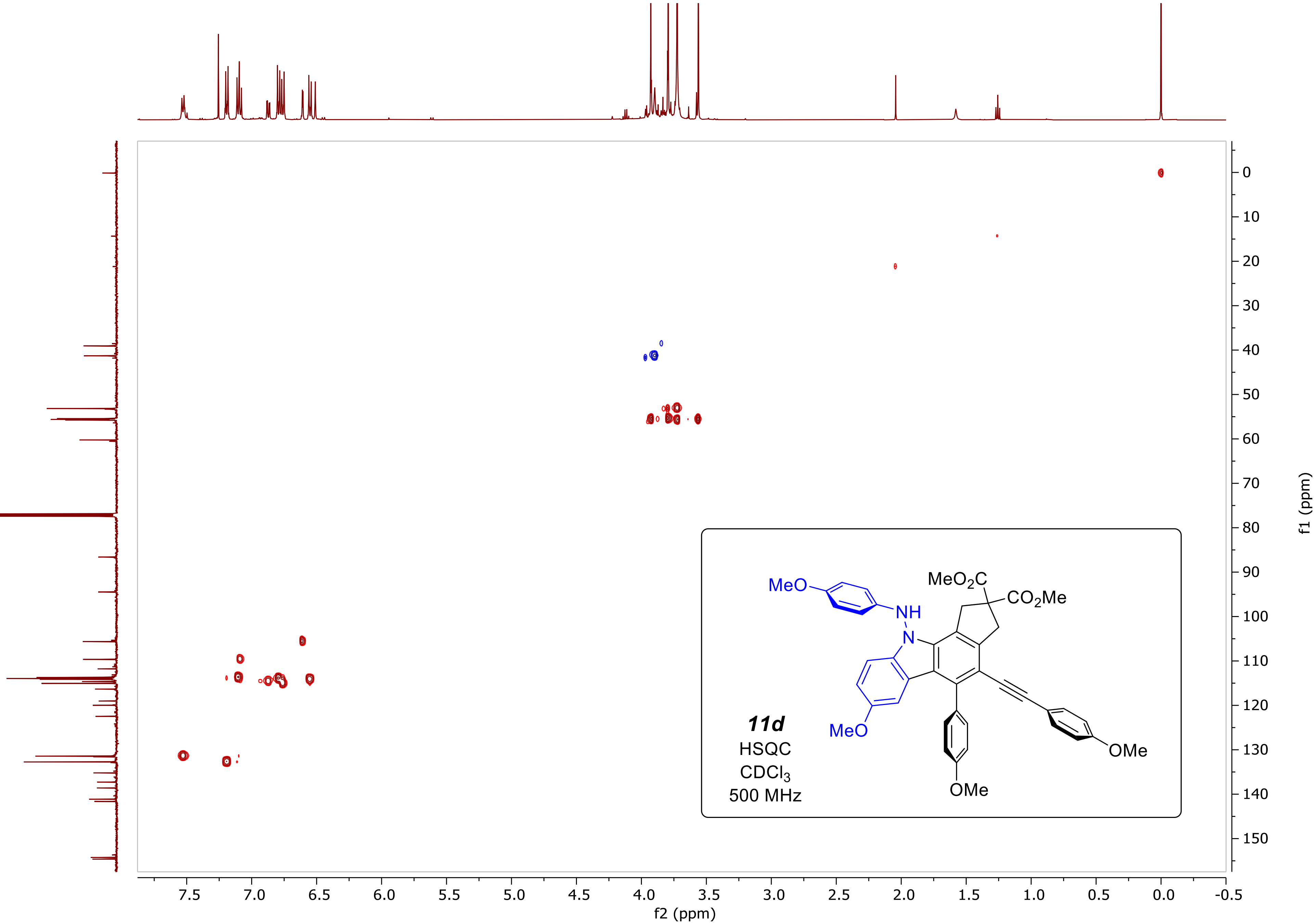
MeO
NH
N
MeO
MeO2C
CO2Me
OMe
OMe
11d
HSQC
CDCl3
500 MHz
f2 (ppm)
f1 (ppm)

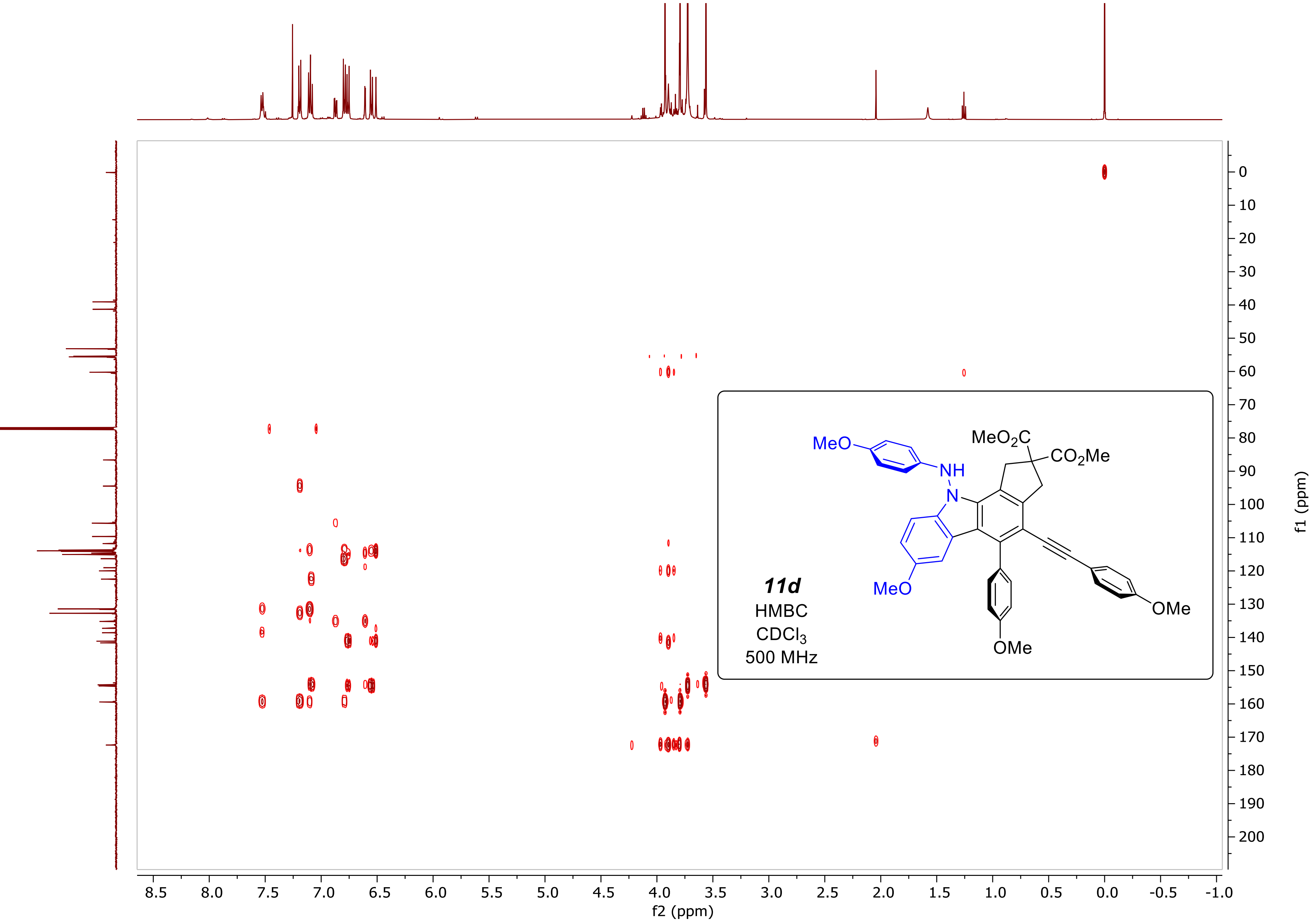
MeO
MeO₂C
CO₂Me
NH
N
MeO
OMe
OMe
11d
HMBC
CDCl₃
500 MHz
f2 (ppm)
f1 (ppm)

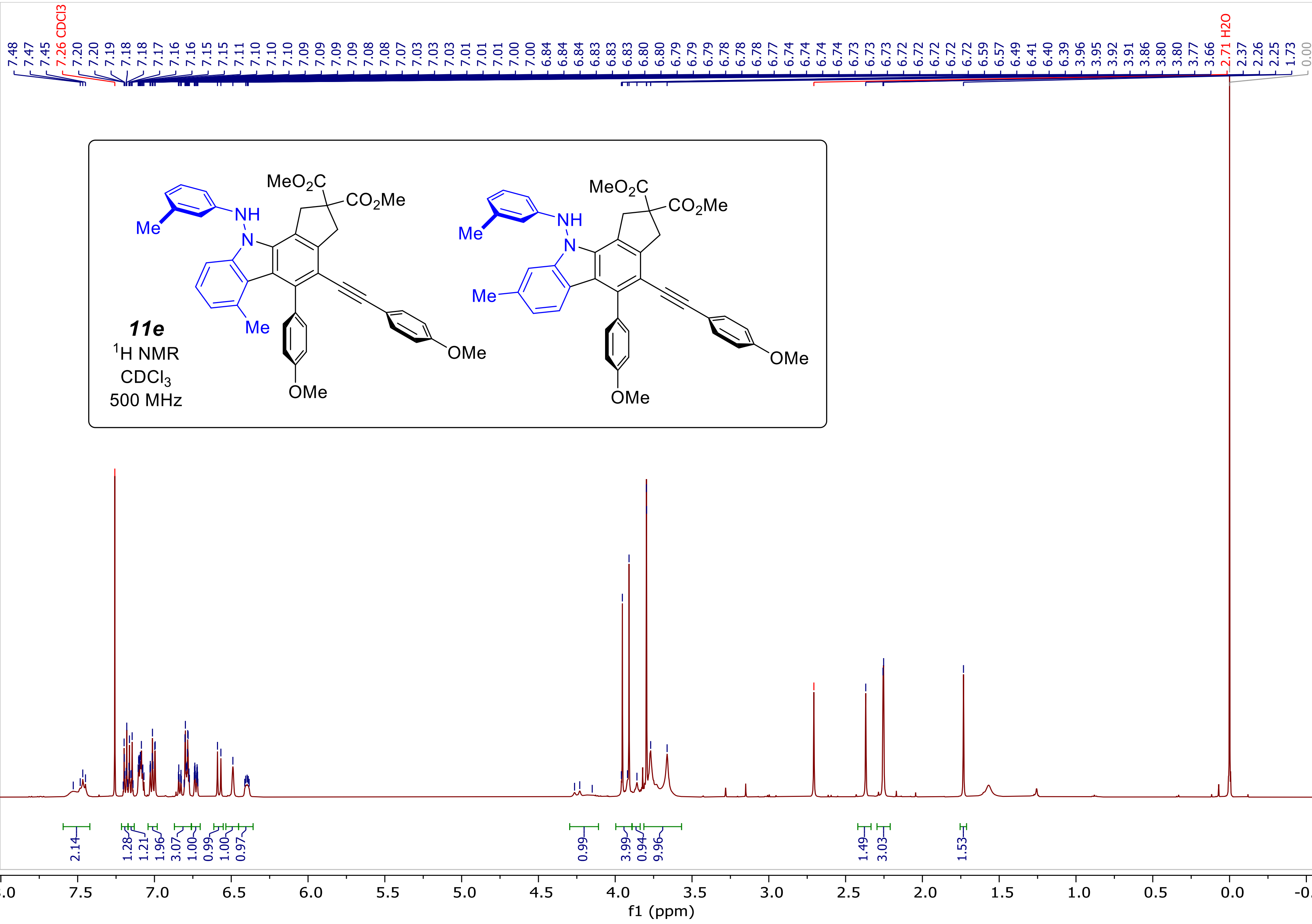
7.48 7.47 7.45 7.26 CDCl3 7.20 7.20 7.19 7.18 7.18 7.17 7.16 7.16 7.15 7.15 7.11 7.10 7.10 7.10 7.09 7.09 7.09 7.09 7.08 7.08 7.07 7.03 7.03 7.03 7.01 7.01 7.01 7.00 7.00 6.84 6.84 6.83 6.83 6.83 6.80 6.80 6.79 6.79 6.79 6.78 6.78 6.78 6.77 6.74 6.74 6.74 6.74 6.73 6.73 6.73 6.72 6.72 6.72 6.72 6.72 6.59 6.57 6.49 6.41 6.40 6.39 3.96 3.95 3.92 3.91 3.86 3.80 3.80 3.77 3.66 2.71 H2O 2.37 2.26 2.25 1.73 0.00
MeO2C
CO2Me
NH
N
Me
Me
OMe
OMe
11e
1H NMR
CDCl3
500 MHz
2.14 1.28 1.21 1.96 3.07 1.00 0.99 1.00 0.97 0.99 3.99 0.94 9.96 1.49 3.03 1.53
7.5 7.0 6.5 6.0 5.5 5.0 4.5 4.0 3.5 3.0 2.5 2.0 1.5 1.0 0.5 0.0
f1 (ppm)

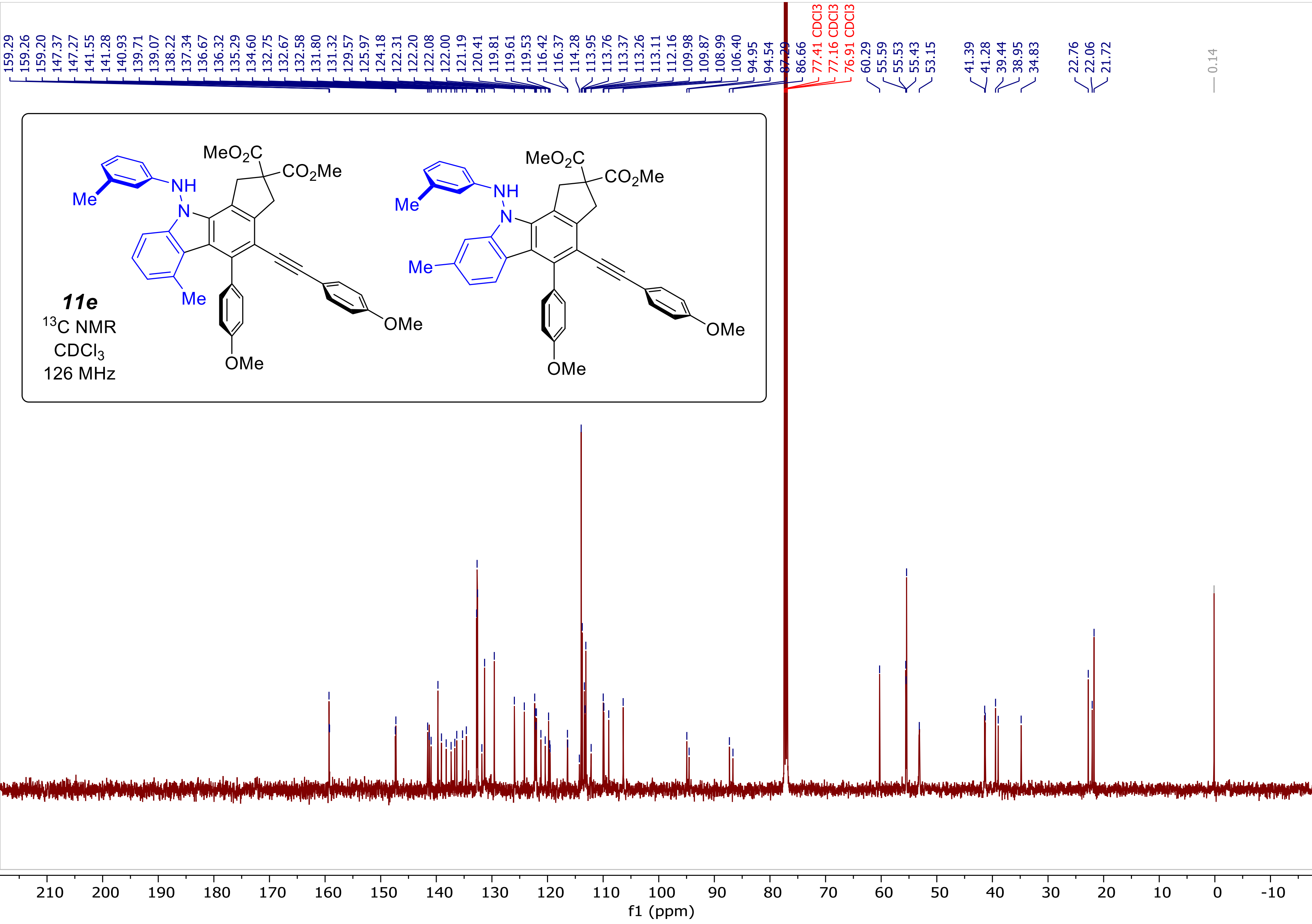
159.29
159.26
159.20
147.37
147.27
141.55
141.28
140.93
139.71
139.07
138.22
137.34
136.67
136.32
135.29
134.60
132.75
132.67
132.58
131.80
131.32
129.57
125.97
124.18
122.31
122.20
122.08
122.00
121.19
120.41
119.81
119.61
119.53
116.42
116.37
114.28
113.95
113.76
113.37
113.26
113.11
112.16
109.98
109.87
108.99
106.40
94.95
94.54
87.29
86.66
77.41 CDCl3
77.16 CDCl3
76.91 CDCl3
60.29
55.59
55.53
55.43
53.15
41.39
41.28
39.44
38.95
34.83
22.76
22.06
21.72
0.14
MeO2C
CO2Me
NH
N
Me
Me
OMe
OMe
11e
13C NMR
CDCl3
126 MHz
f1 (ppm)

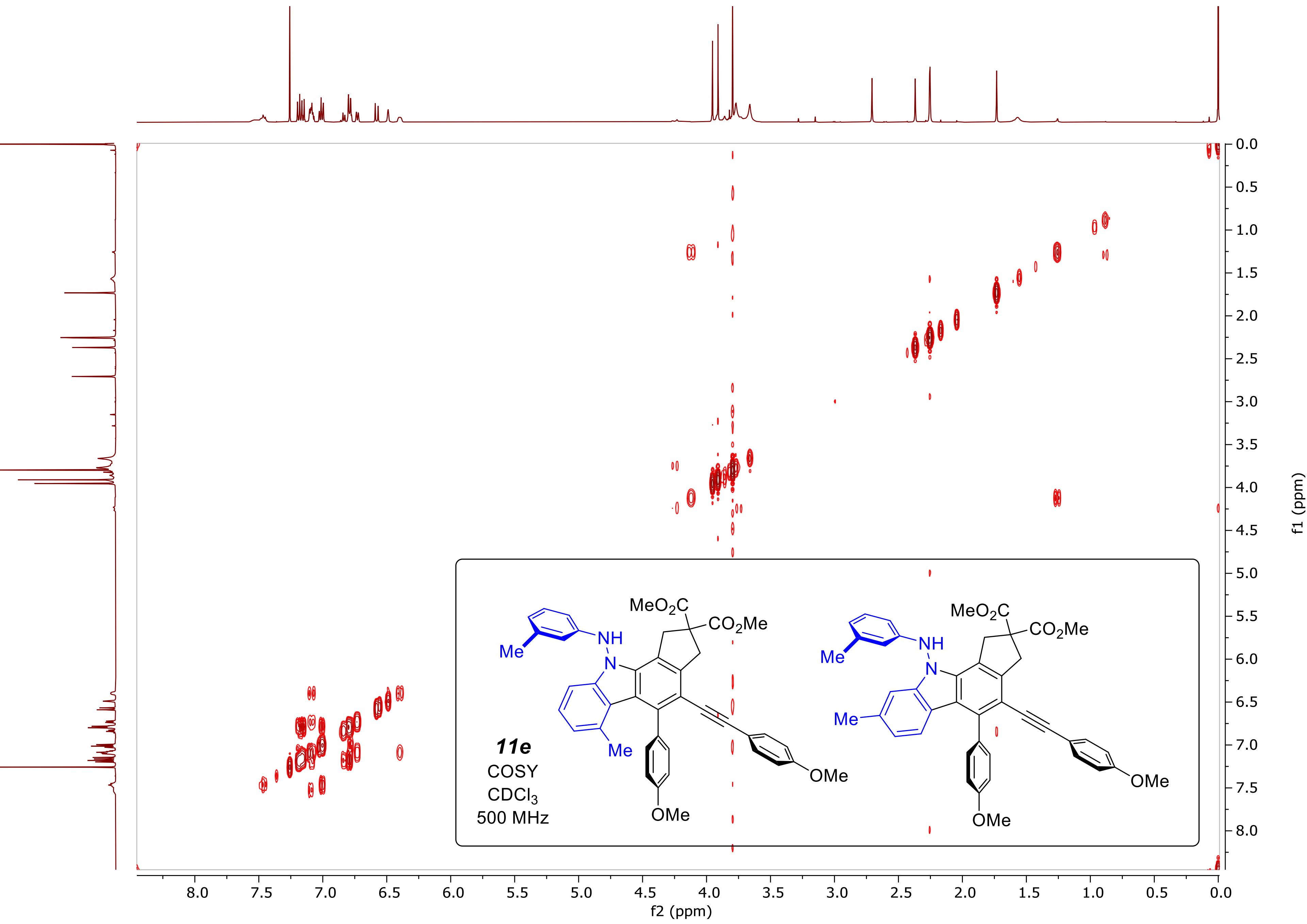

11e
COSY
CDCl3
500 MHz
MeO2C
CO2Me
NH
N
Me
OMe
f1 (ppm)
f2 (ppm)

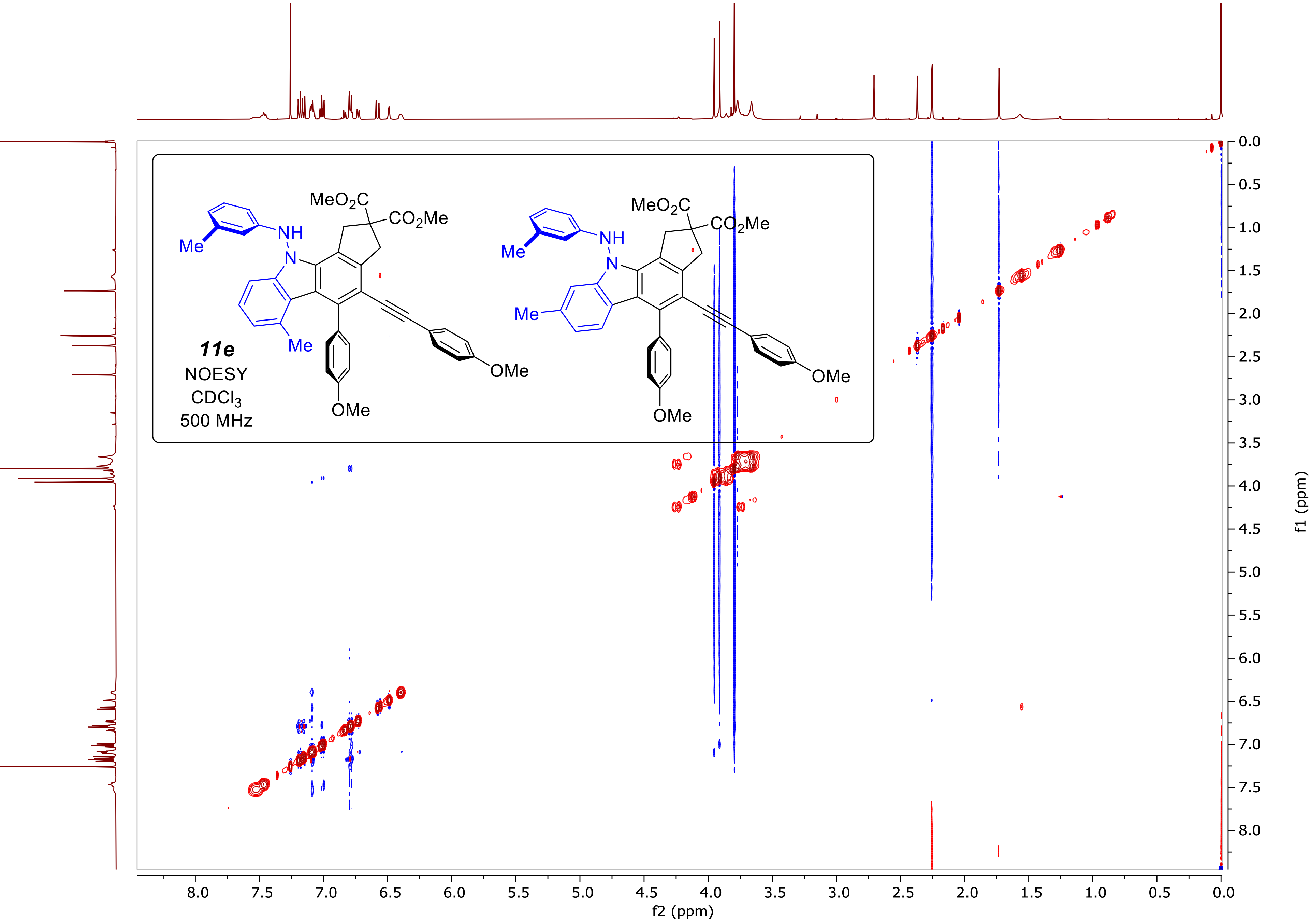

MeO2C
CO2Me
Me
NH
N
Me
OMe
OMe
11e
NOESY
CDCl3
500 MHz
MeO2C
CO2Me
Me
NH
N
Me
OMe
OMe
f2 (ppm)
f1 (ppm)

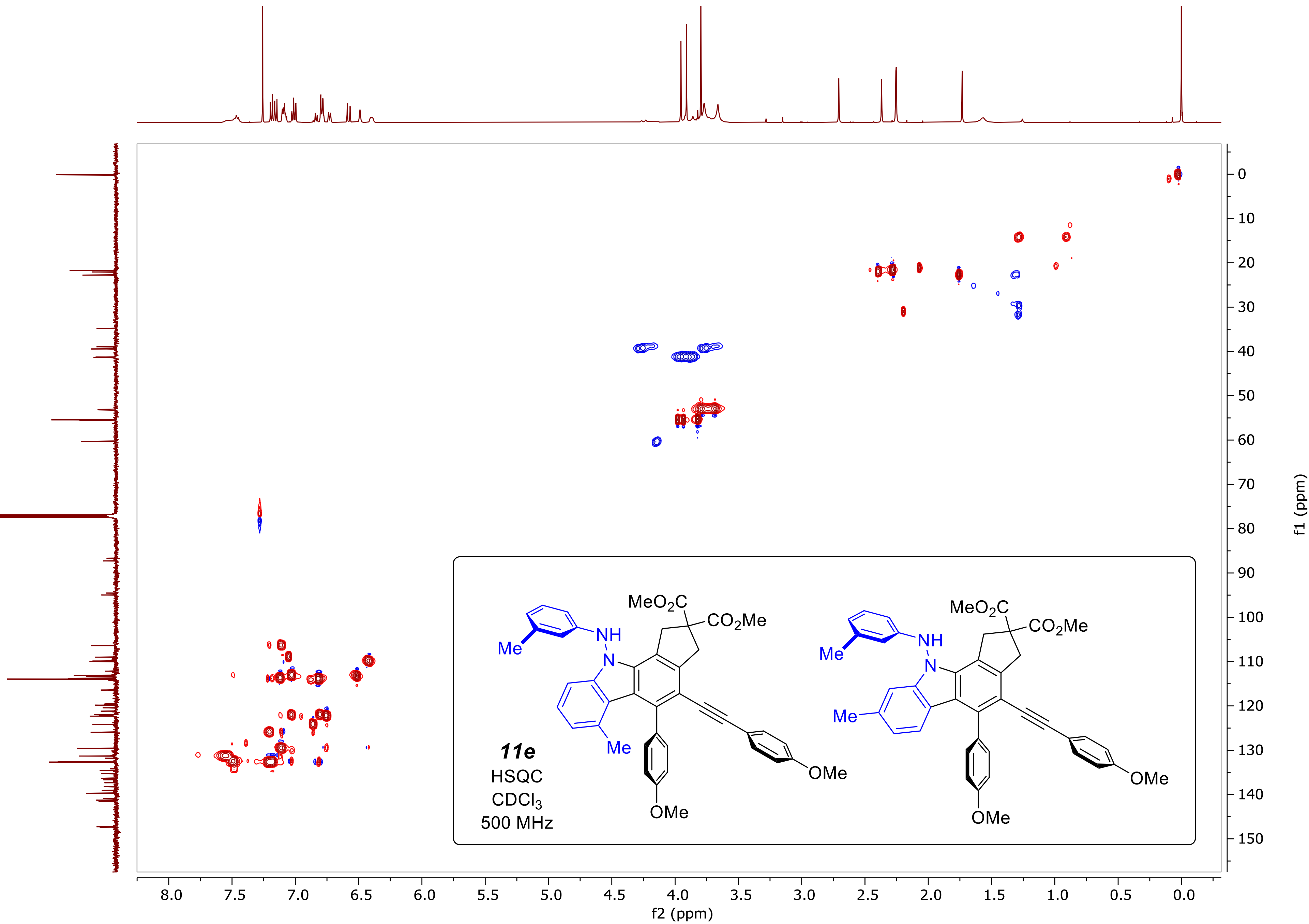
11e
HSQC
CDCl3
500 MHz
MeO2C
CO2Me
NH
N
Me
OMe
f2 (ppm)
f1 (ppm)

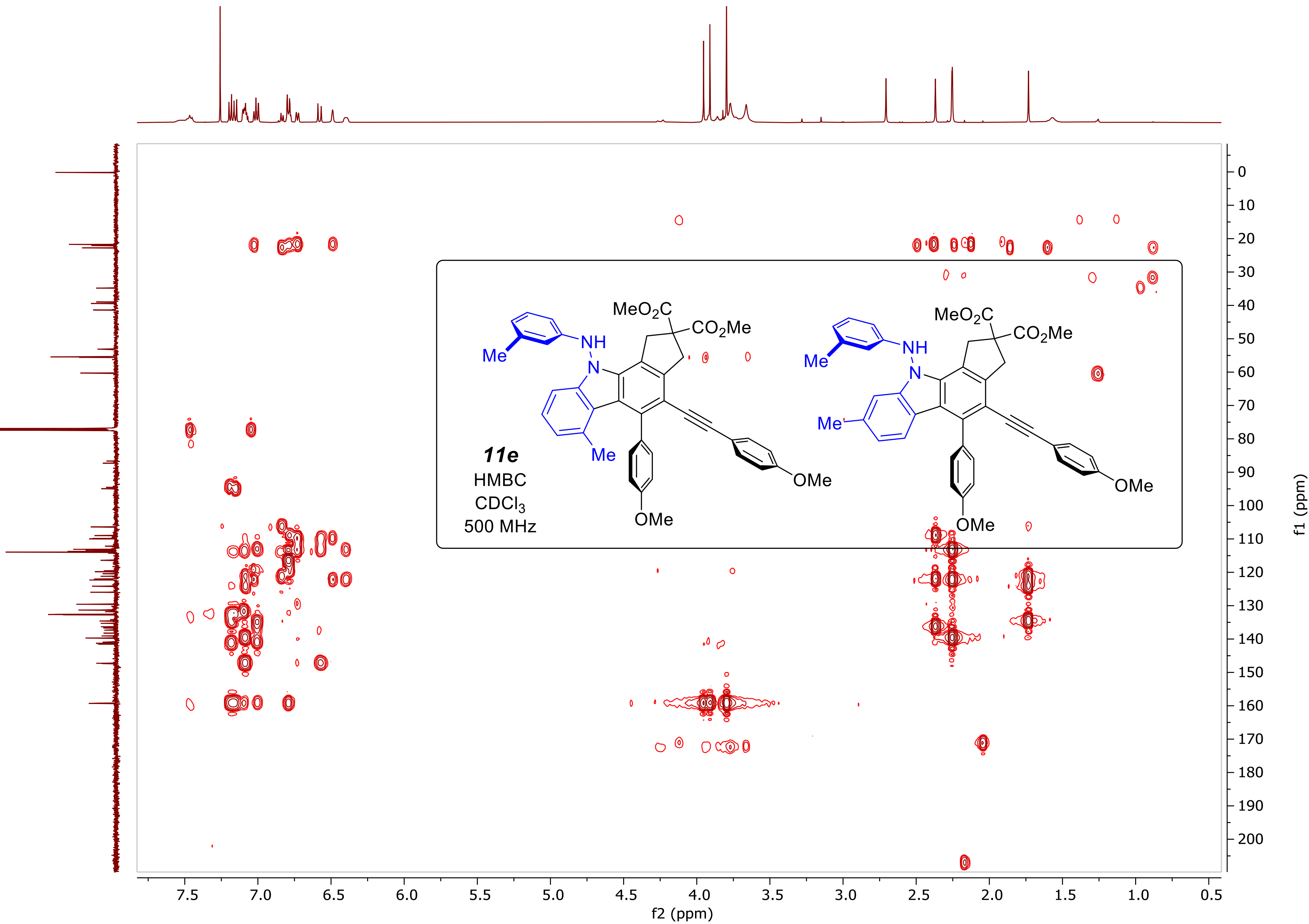
11e
HMBC
CDCl3
500 MHz
MeO2C
CO2Me
NH
N
Me
OMe
f1 (ppm)
f2 (ppm)

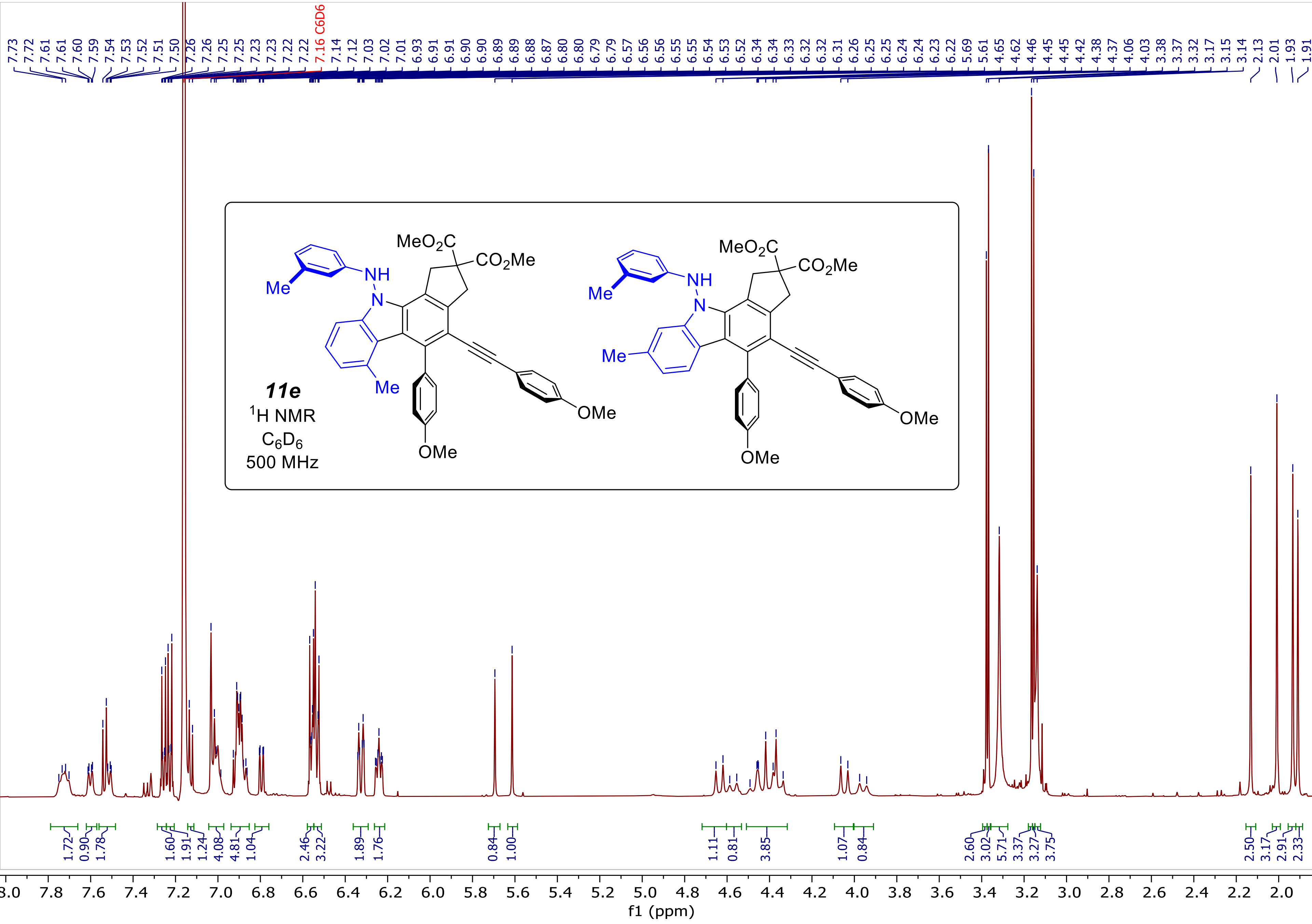
11e
1H NMR
C6D6
500 MHz
f1 (ppm)

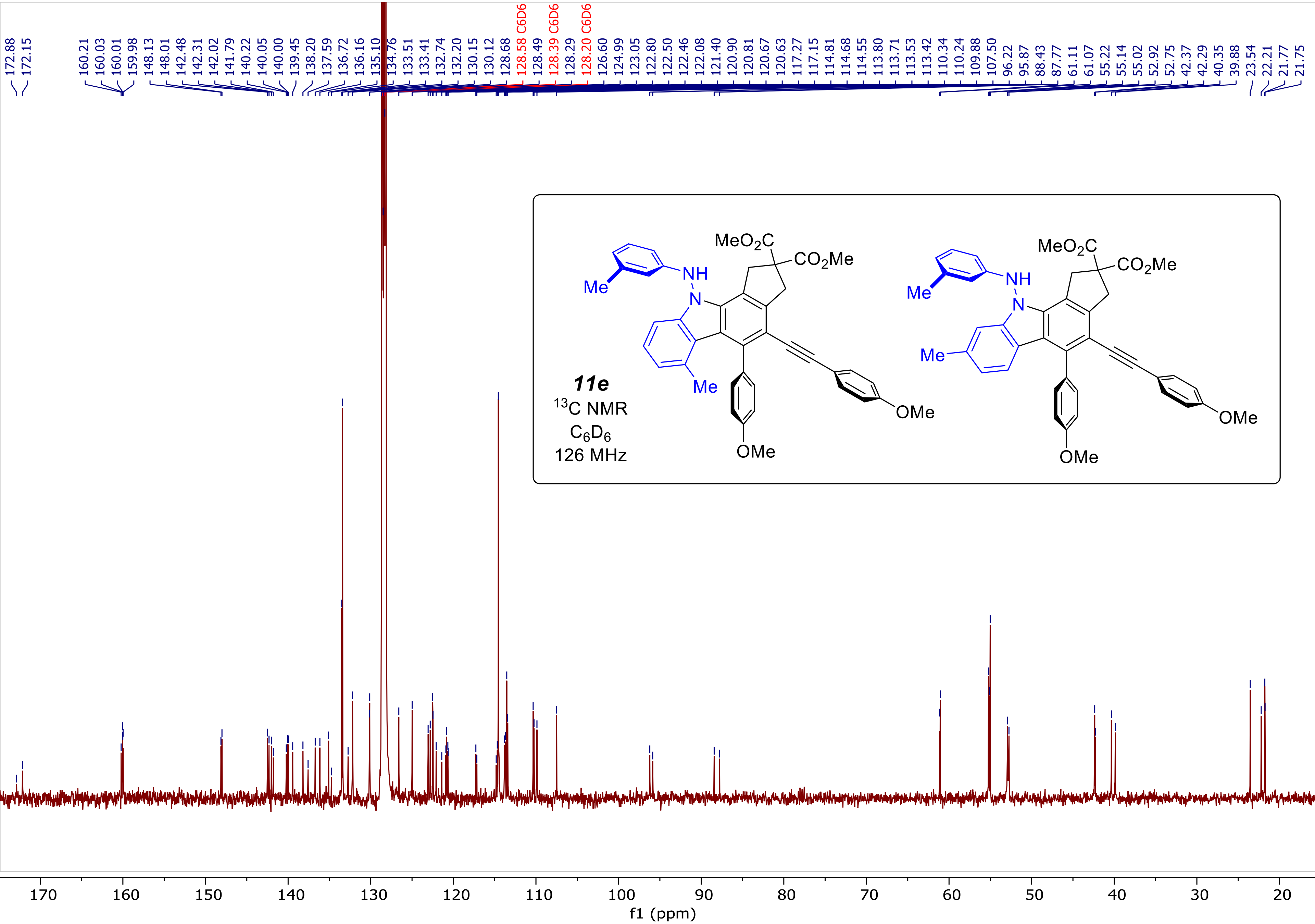
172.88
172.15
160.21
160.03
160.01
159.98
148.13
148.01
142.48
142.31
142.02
141.79
140.22
140.05
140.00
139.45
138.20
137.59
136.72
136.16
135.10
134.76
133.51
133.41
132.74
132.20
130.15
130.12
128.68
128.58 C6D6
128.49
128.39 C6D6
128.29
128.20 C6D6
126.60
124.99
123.05
122.80
122.50
122.46
122.08
121.40
120.90
120.81
120.67
120.63
117.27
117.15
114.81
114.68
114.55
113.80
113.71
113.53
113.42
110.34
110.24
109.88
107.50
96.22
95.87
88.43
87.77
61.11
61.07
55.22
55.14
55.02
52.92
52.75
42.37
42.29
40.35
39.88
23.54
22.21
21.77
21.75
MeO2C
CO2Me
NH
N
Me
Me
OMe
OMe
11e
13C NMR
C6D6
126 MHz
MeO2C
CO2Me
NH
N
Me
Me
OMe
OMe
170 160 150 140 130 120 110 100 90 80 70 60 50 40 30 20
f1 (ppm)

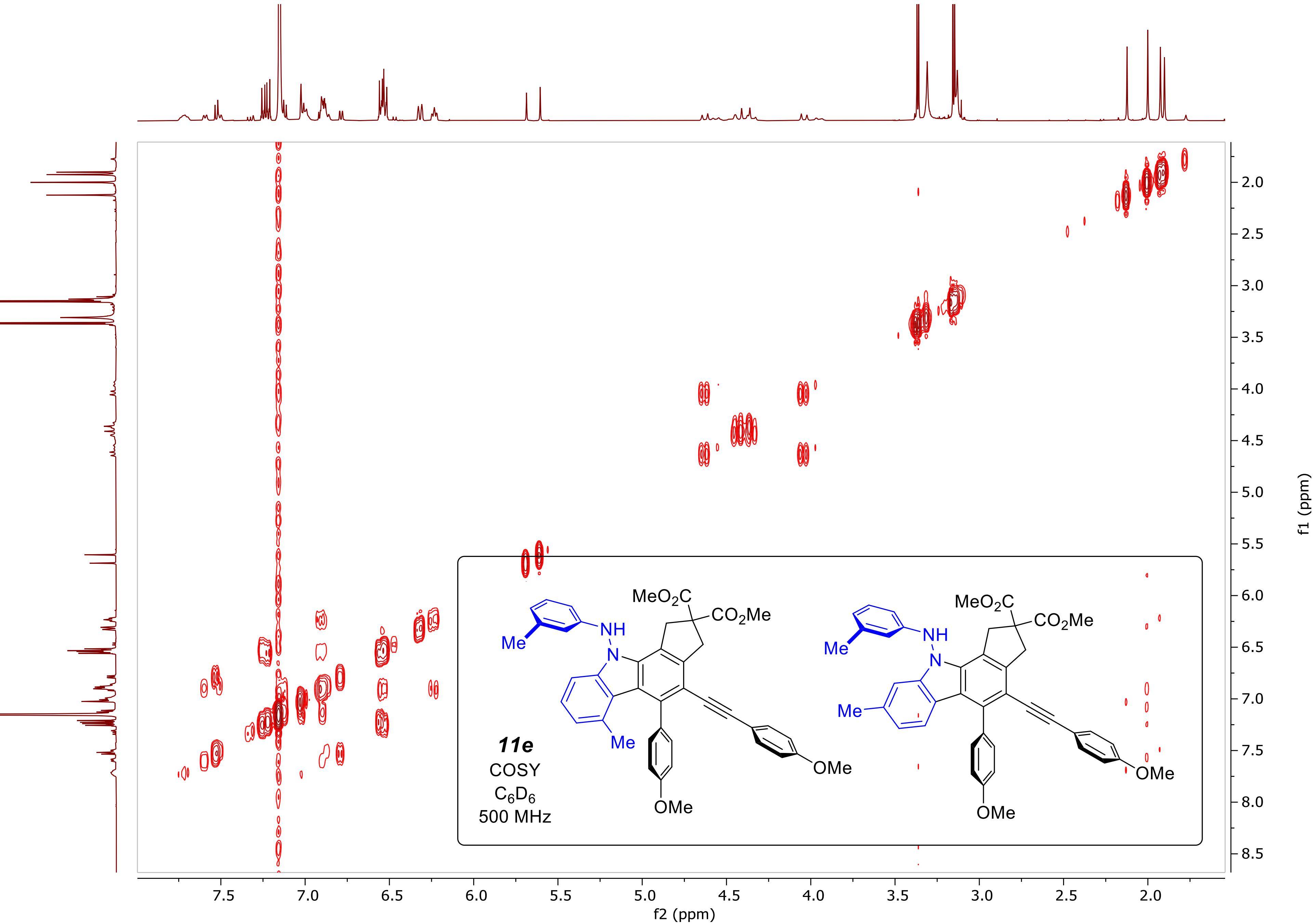
11e
COSY
C6D6
500 MHz
MeO2C
CO2Me
NH
N
Me
OMe
f1 (ppm)
f2 (ppm)

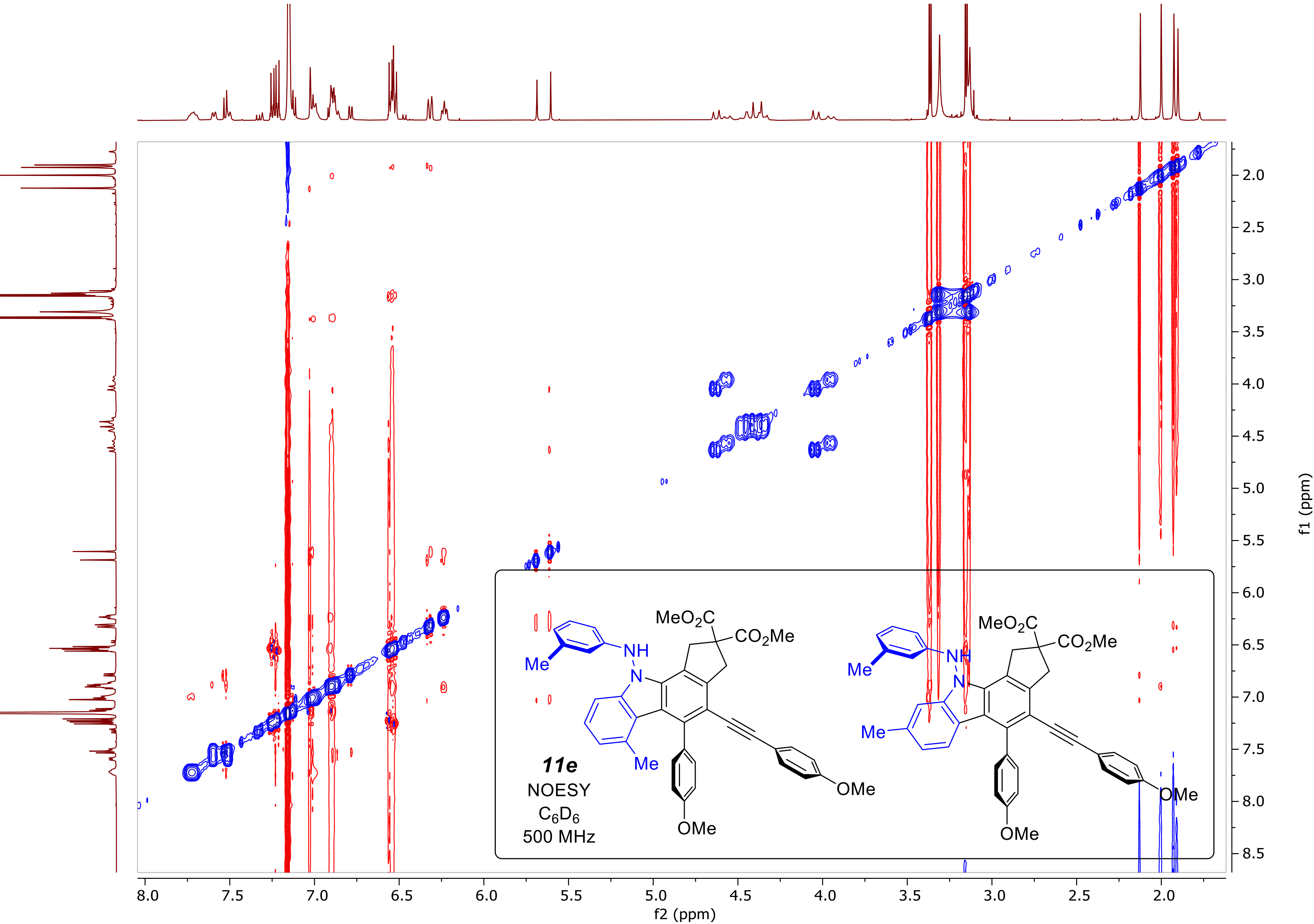
11e
NOESY
C_6D_6
500 MHz
f2 (ppm)
f1 (ppm)

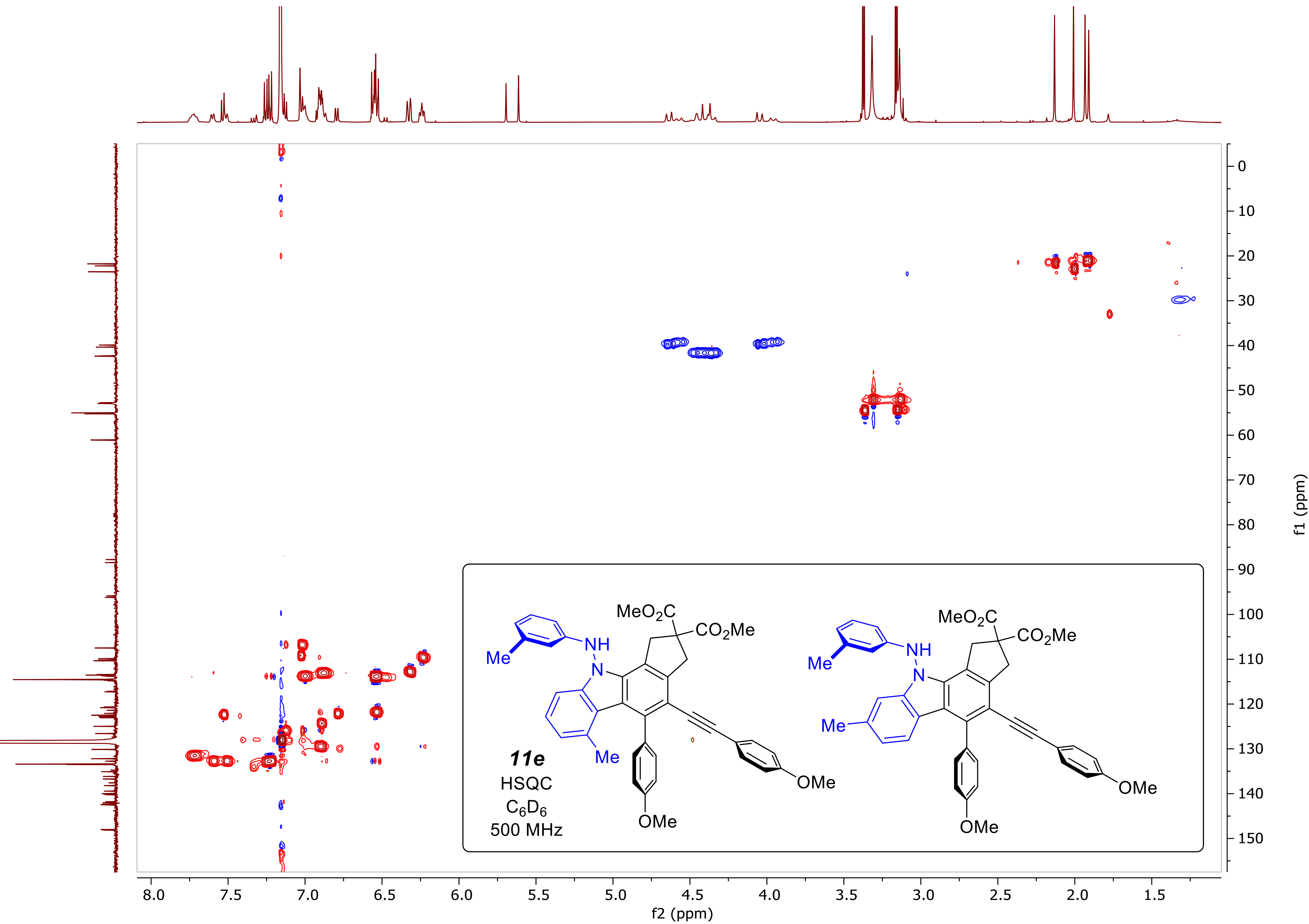

11e
HSQC
C_6D_6
500 MHz
MeO_2C
CO_2Me
NH
N
Me
Me
OMe
OMe
f2 (ppm)
f1 (ppm)

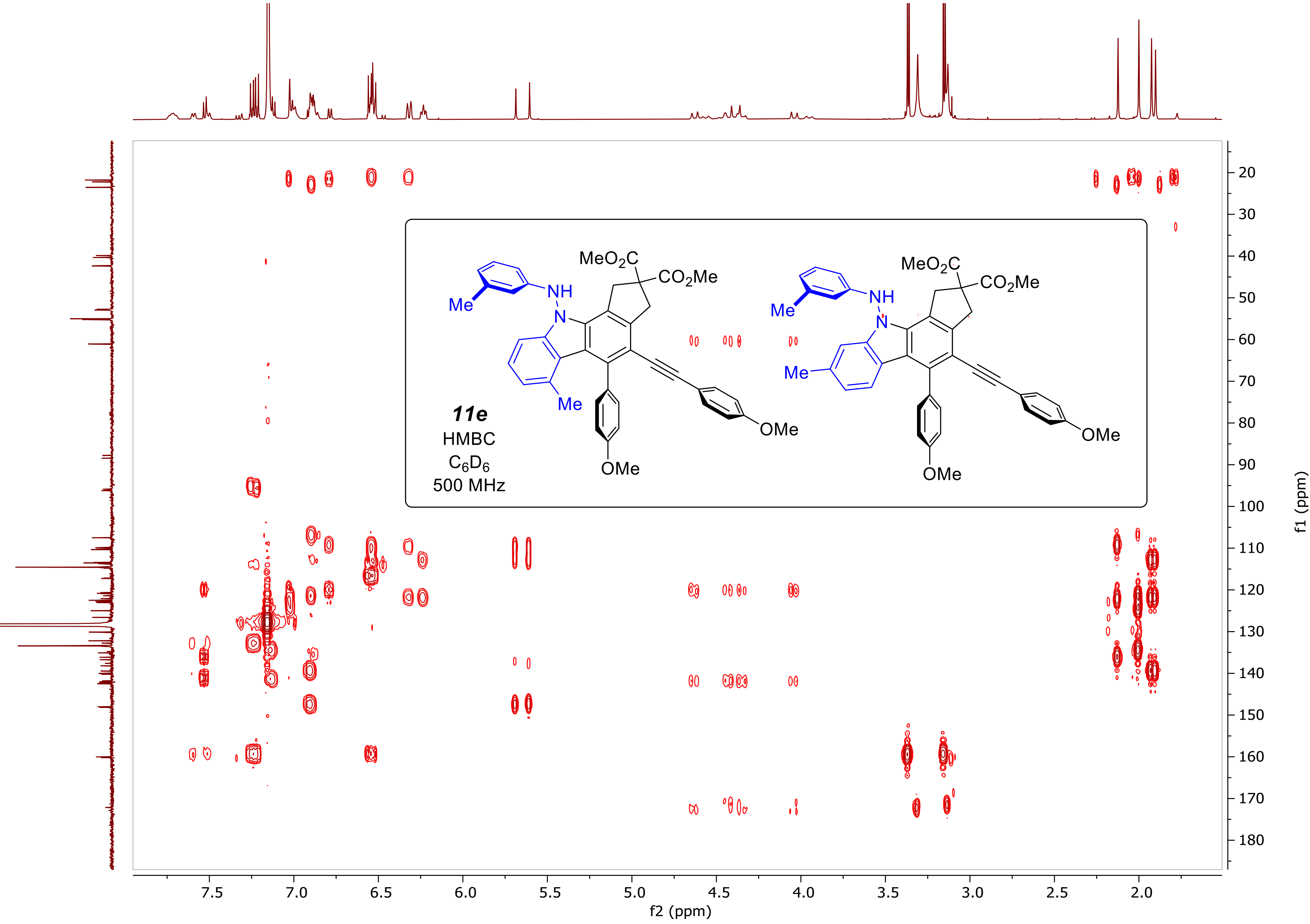
11e
HMBC
C_6D_6
500 MHz
MeO_2C
CO_2Me
NH
N
Me
Me
OMe
OMe
f2 (ppm)
f1 (ppm)

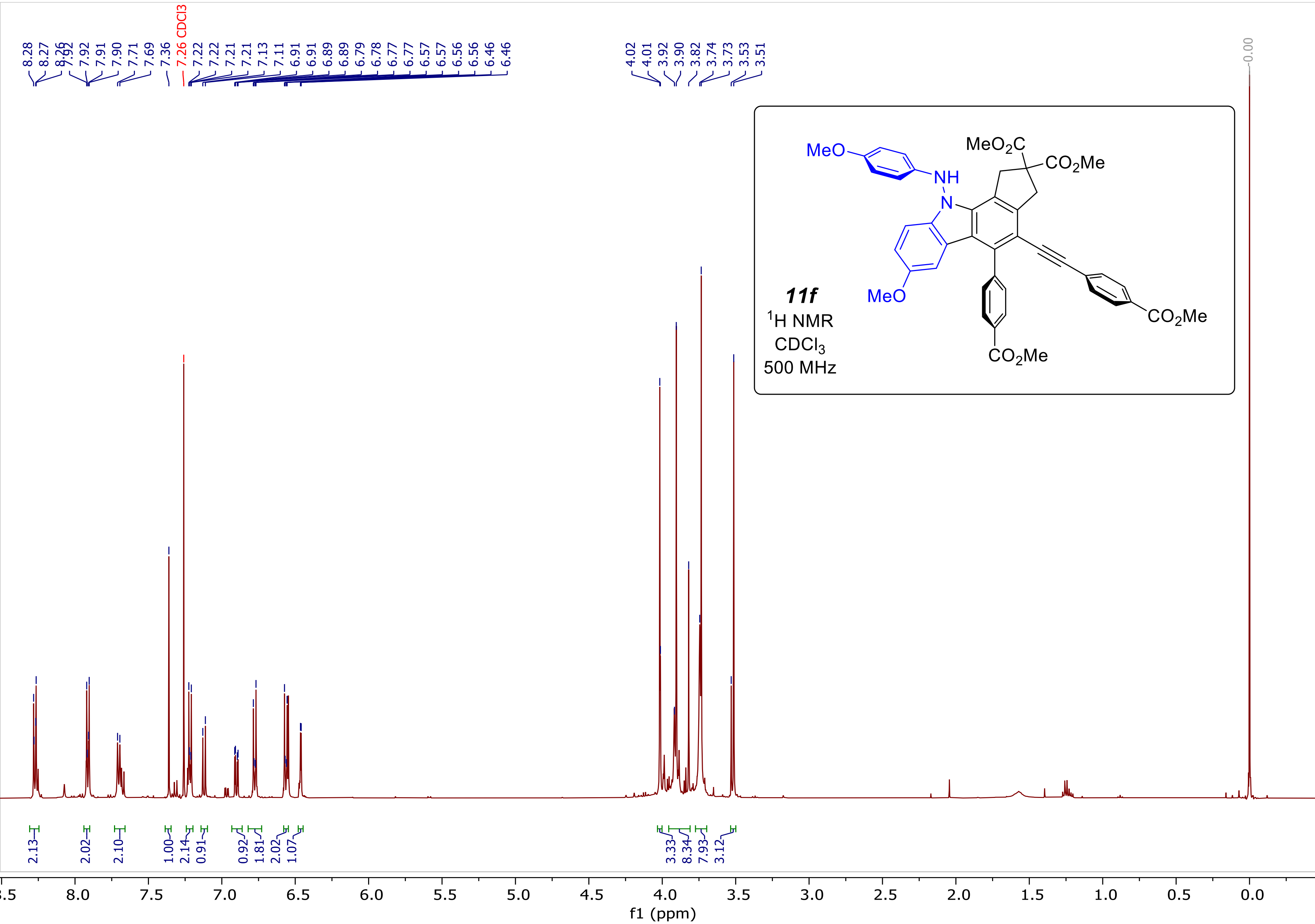

8.28 8.27 8.26 7.92 7.92 7.91 7.90 7.71 7.69 7.36 7.26 CDCl3 7.22 7.22 7.21 7.21 7.13 7.11 6.91 6.91 6.89 6.89 6.79 6.78 6.77 6.77 6.57 6.57 6.56 6.56 6.46 6.46
4.02 4.01 3.92 3.90 3.82 3.74 3.73 3.53 3.51
-0.00
MeO
NH
N
MeO
MeO2C
CO2Me
CO2Me
CO2Me
11f
1H NMR
CDCl3
500 MHz
2.13 2.02 2.10 1.00 2.14 0.91 0.92 1.81 2.02 1.07
3.33 8.34 7.93 3.12
8.5 8.0 7.5 7.0 6.5 6.0 5.5 5.0 4.5 4.0 3.5 3.0 2.5 2.0 1.5 1.0 0.5 0.0
f1 (ppm)

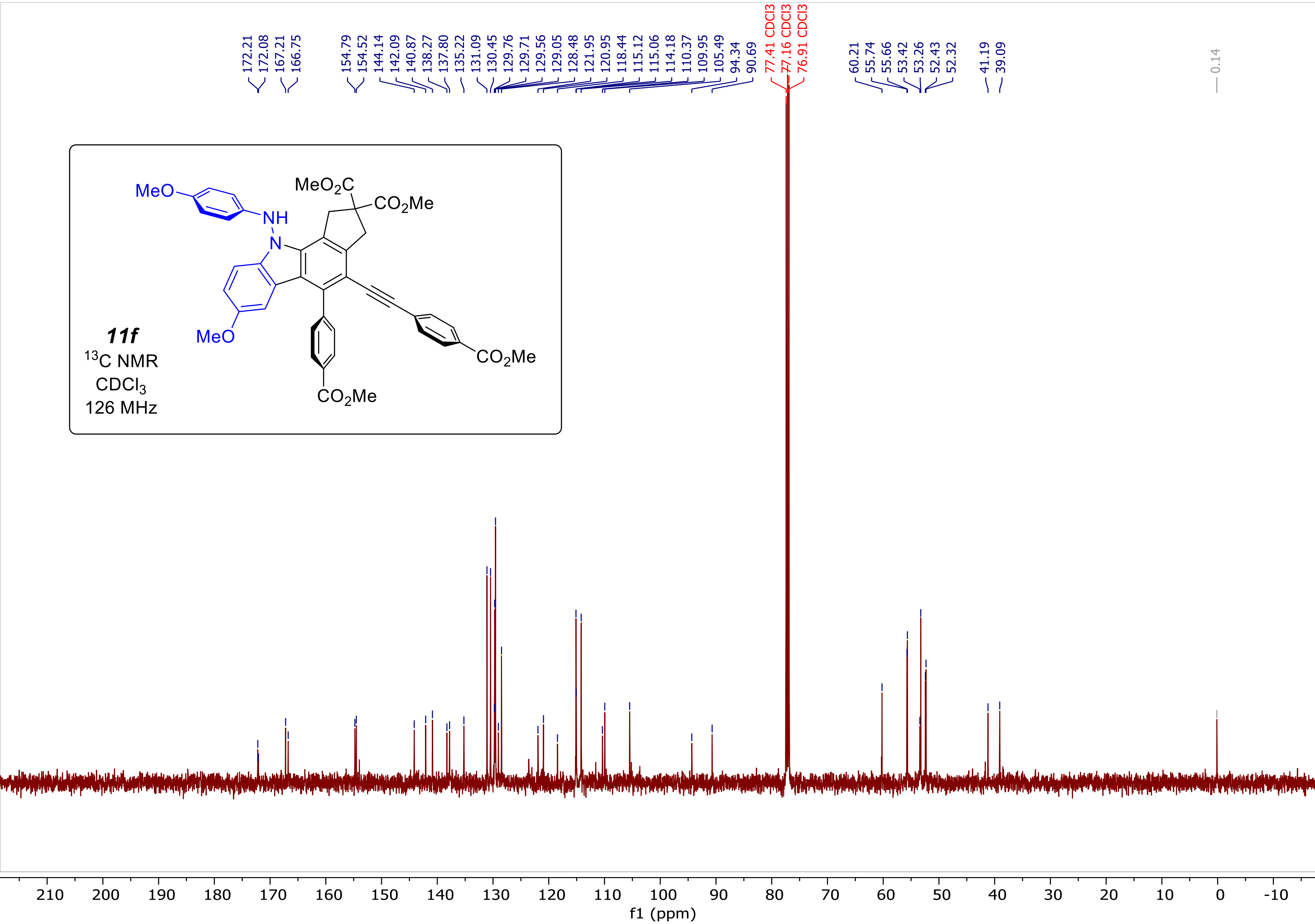
172.21
172.08
167.21
166.75
154.79
154.52
144.14
142.09
140.87
138.27
137.80
135.22
131.09
130.45
129.76
129.71
129.56
129.05
128.48
121.95
120.95
118.44
115.12
115.06
114.18
110.37
109.95
105.49
94.34
90.69
77.41 CDCl3
77.16 CDCl3
76.91 CDCl3
60.21
55.74
55.66
53.42
53.26
52.43
52.32
41.19
39.09
0.14
MeO
NH
N
MeO
MeO2C
CO2Me
CO2Me
CO2Me
11f
13C NMR
CDCl3
126 MHz
f1 (ppm)

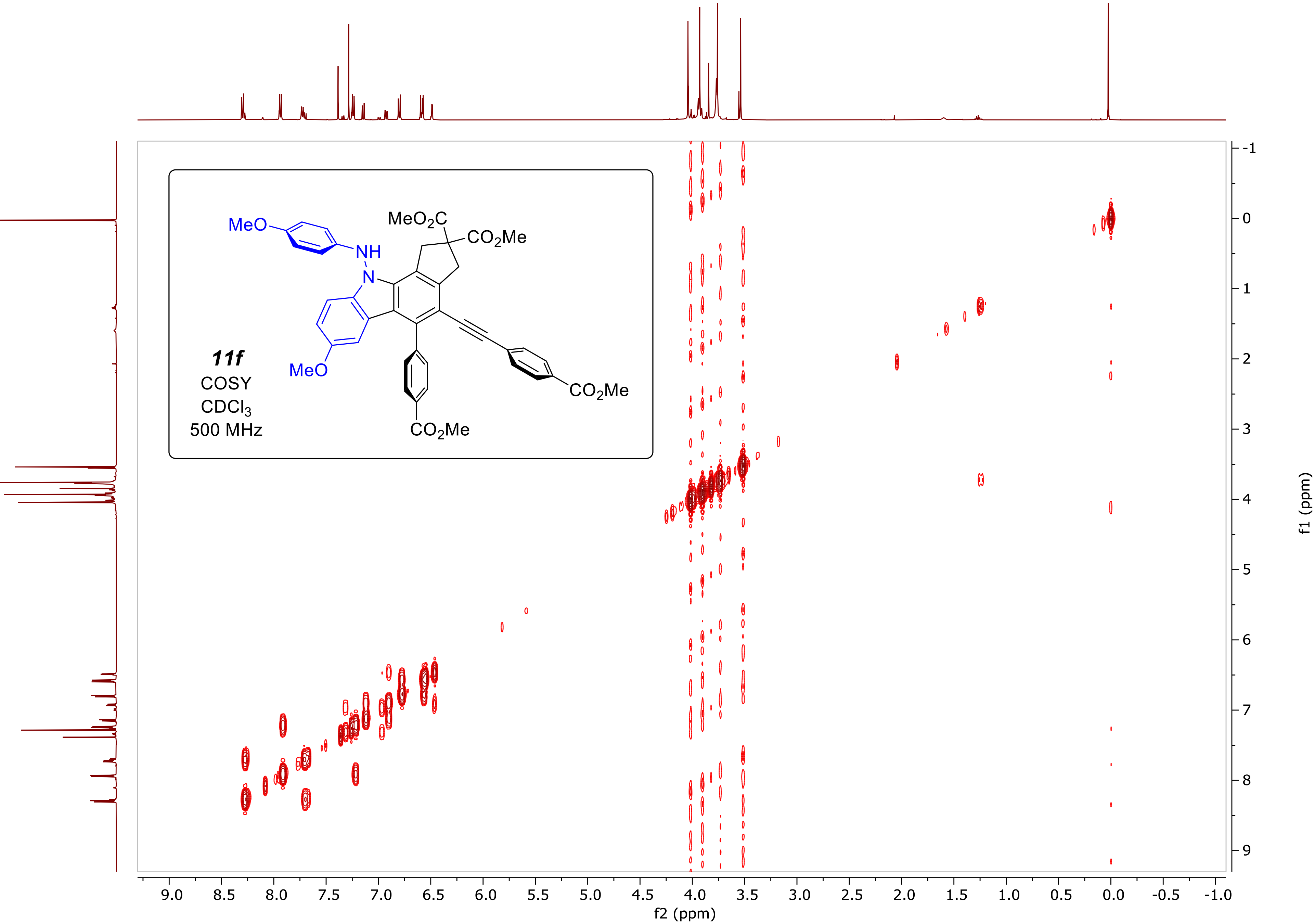
MeO
NH
N
MeO
MeO2C
CO2Me
CO2Me
CO2Me
11f
COSY
CDCl3
500 MHz
f2 (ppm)
f1 (ppm)

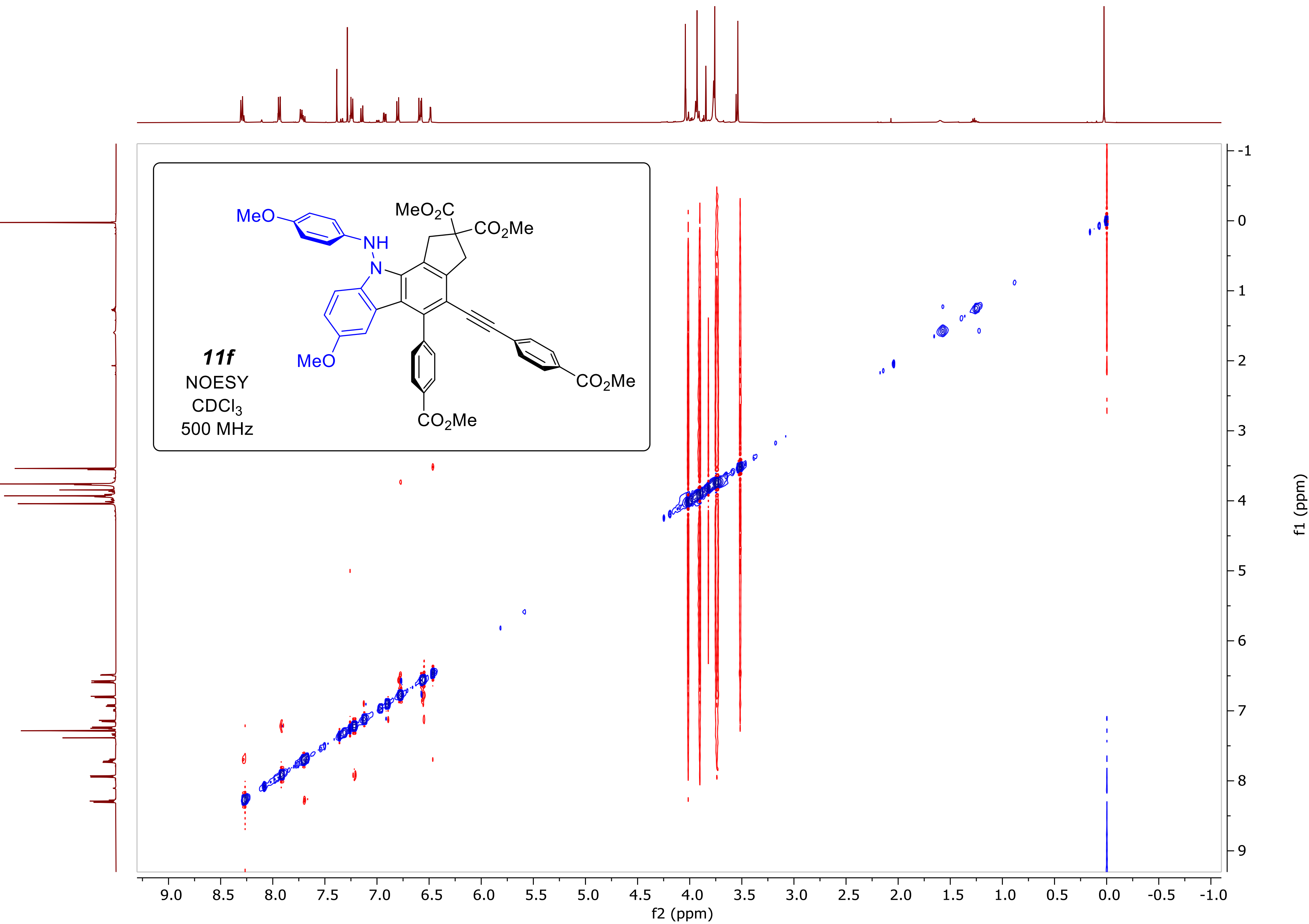
MeO
NH
N
MeO
MeO$_2$C
CO$_2$Me
CO$_2$Me
CO$_2$Me
11f
NOESY
CDCl$_3$
500 MHz
f2 (ppm)
f1 (ppm)

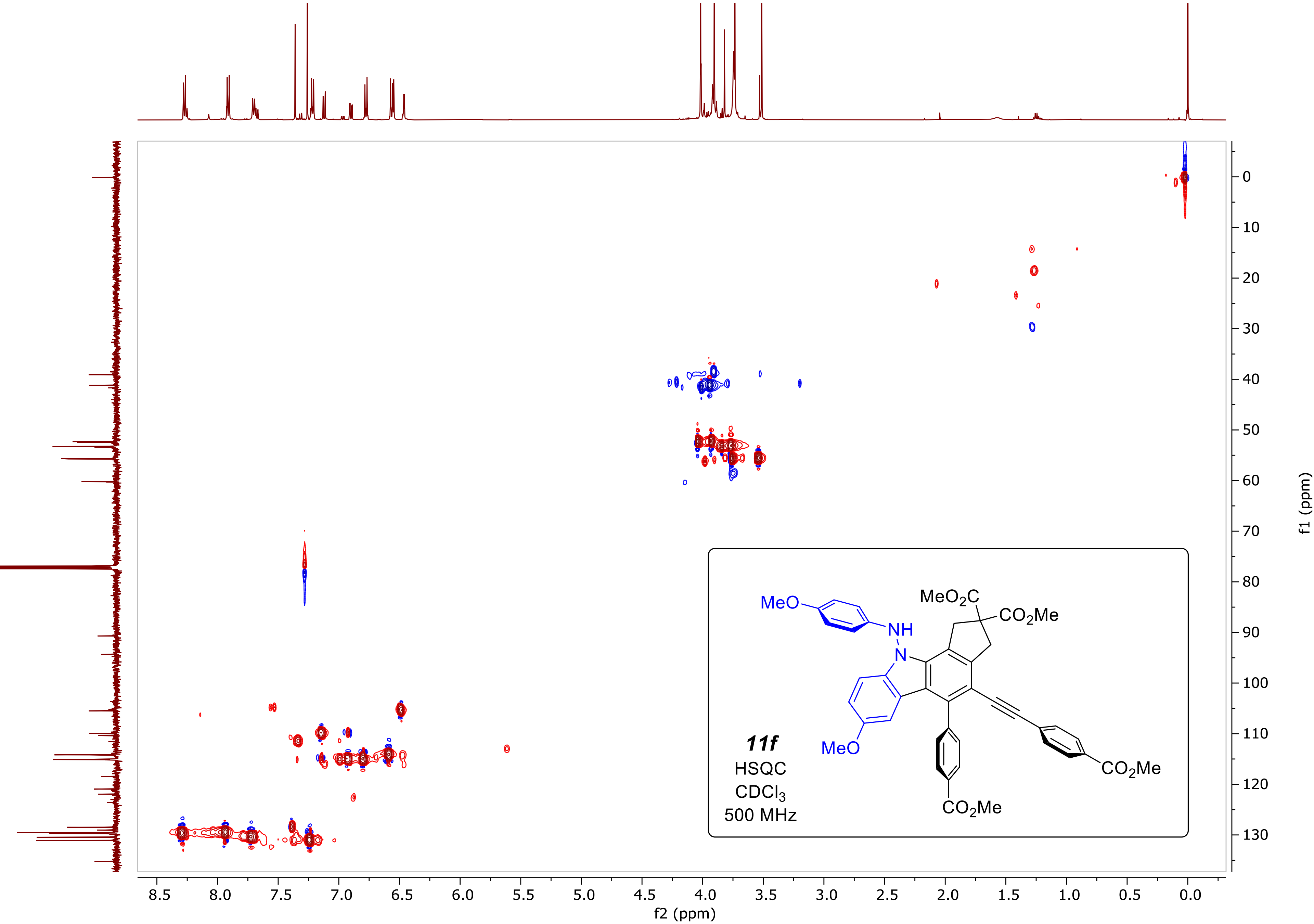

MeO
NH
N
MeO
MeO2C
CO2Me
CO2Me
CO2Me
11f
HSQC
CDCl3
500 MHz
f2 (ppm)
f1 (ppm)

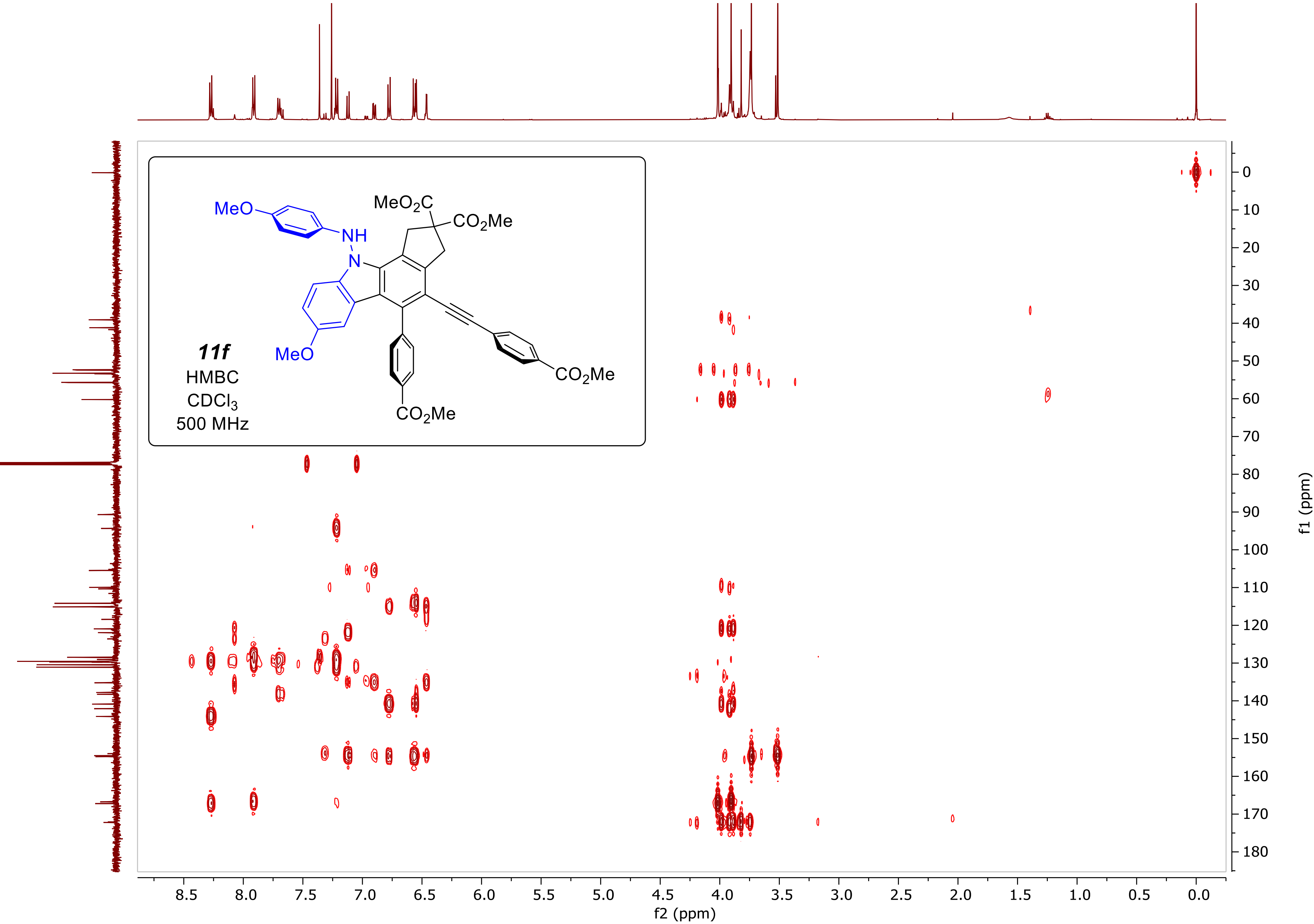
MeO
NH
N
MeO
MeO$_2$C
CO$_2$Me
CO$_2$Me
CO$_2$Me
11f
HMBC
CDCl$_3$
500 MHz
f2 (ppm)
f1 (ppm)

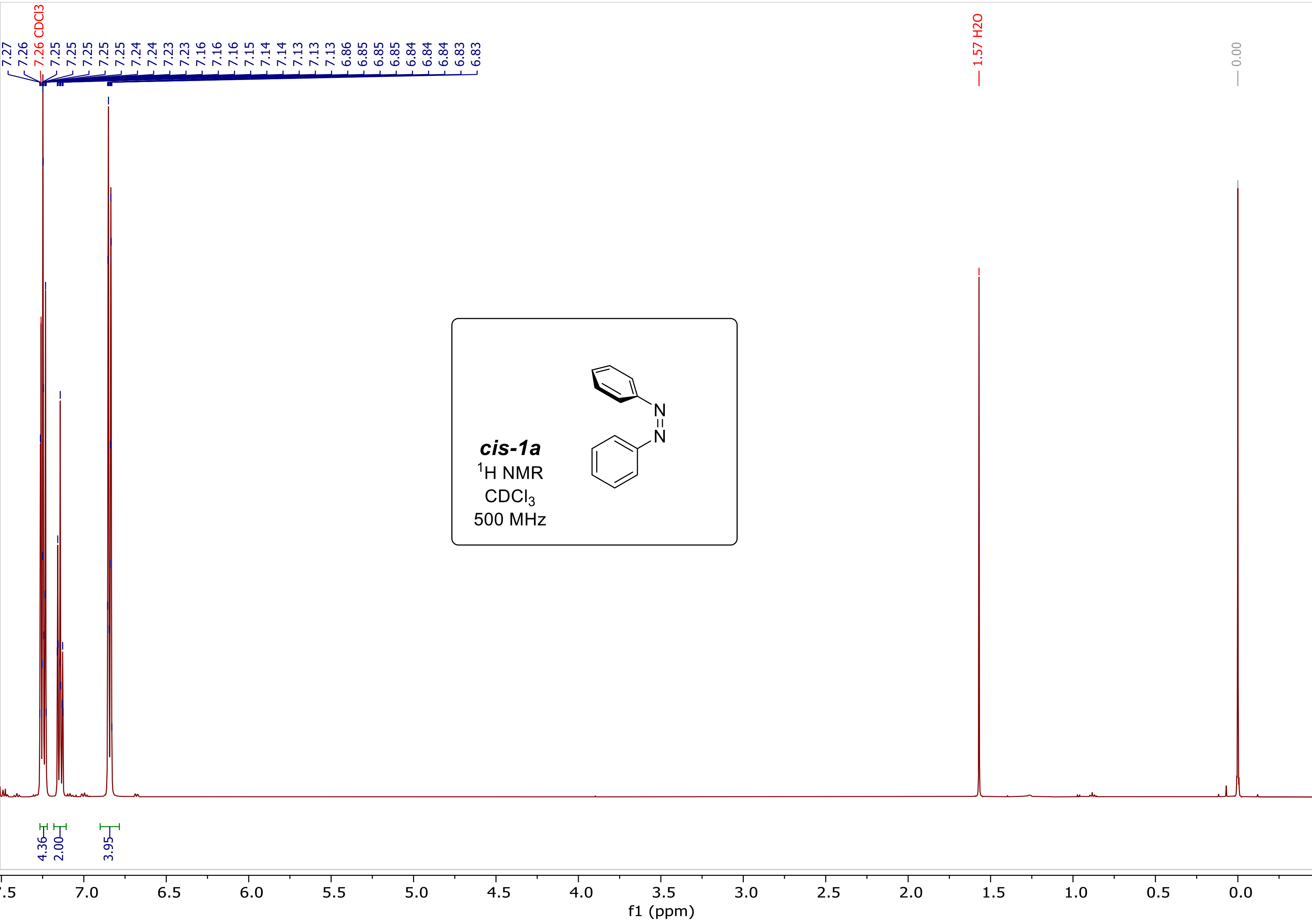
7.27
7.26
7.26 CDCl3
7.25
7.25
7.25
7.25
7.25
7.24
7.24
7.23
7.23
7.16
7.16
7.16
7.15
7.14
7.14
7.13
7.13
7.13
6.86
6.85
6.85
6.85
6.84
6.84
6.84
6.83
6.83
1.57 H2O
0.00
cis-1a
1H NMR
CDCl3
500 MHz
N
N
4.36
2.00
3.95
7.5
7.0
6.5
6.0
5.5
5.0
4.5
4.0
3.5
3.0
2.5
2.0
1.5
1.0
0.5
0.0
f1 (ppm)

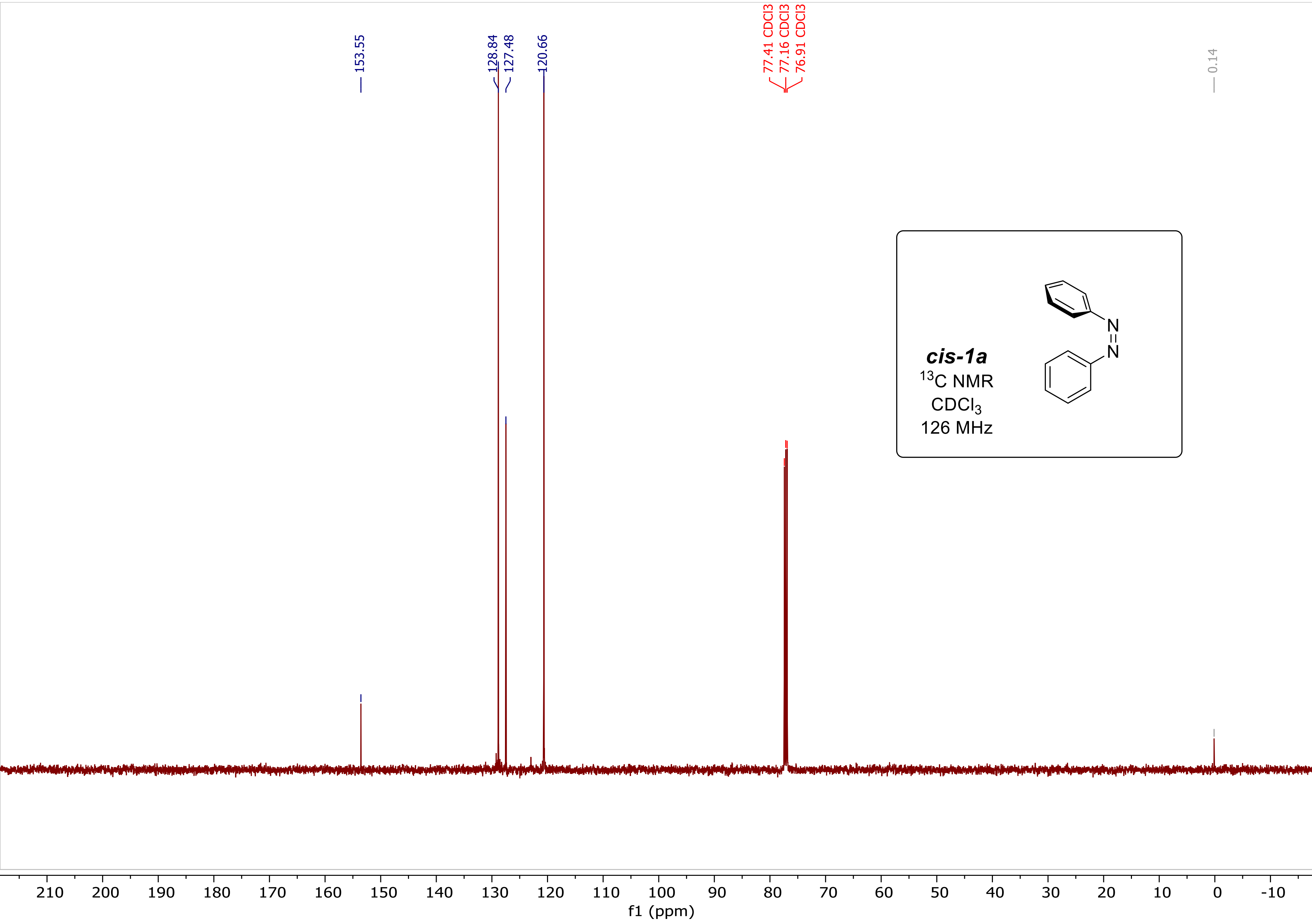
153.55
128.84
127.48
120.66
77.41 CDCl3
77.16 CDCl3
76.91 CDCl3
0.14
cis-1a
13C NMR
CDCl3
126 MHz
N
N
210
200
190
180
170
160
150
140
130
120
110
100
90
80
70
60
50
40
30
20
10
0
-10
f1 (ppm)

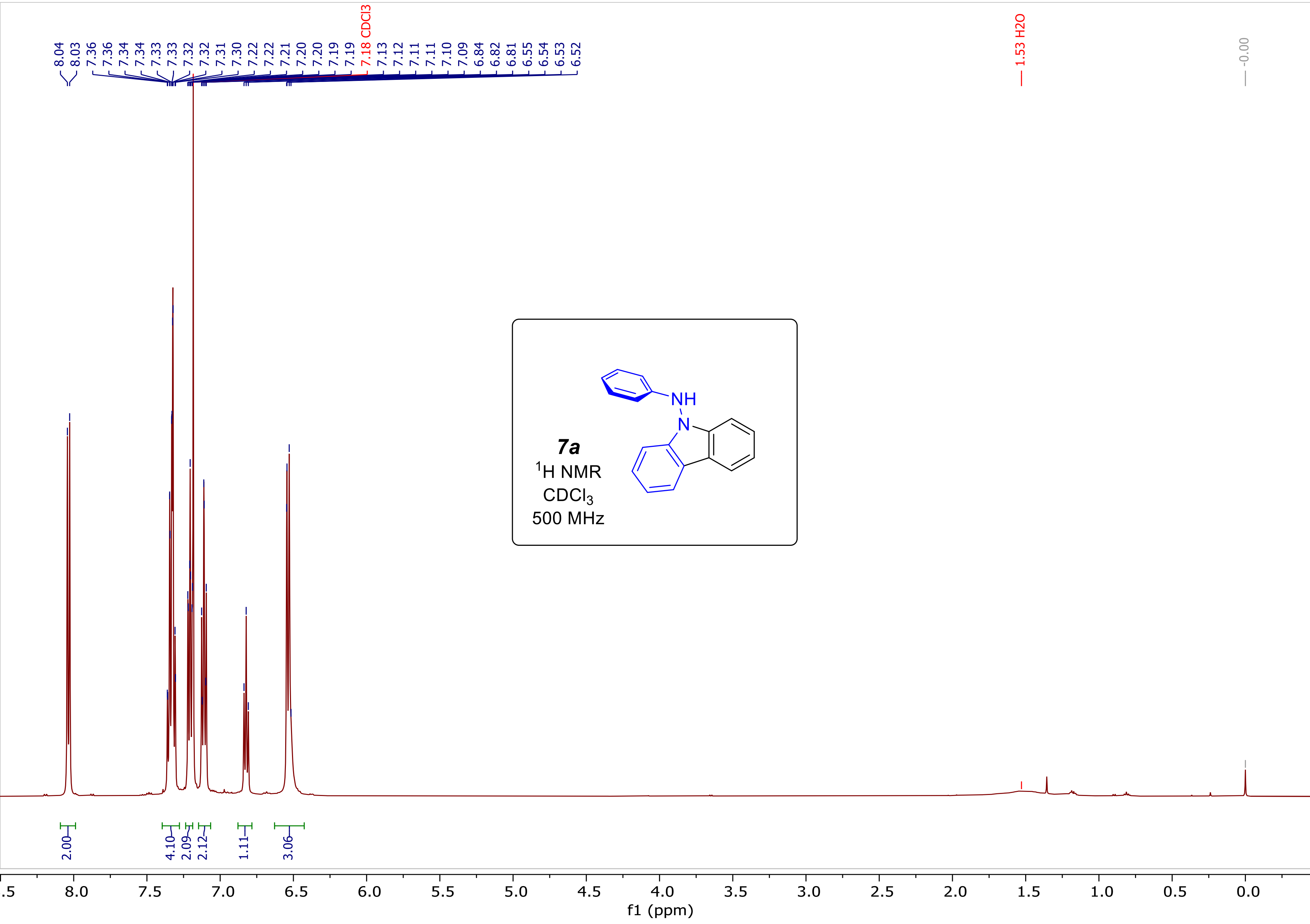
8.04
8.03
7.36
7.36
7.34
7.34
7.33
7.33
7.32
7.32
7.31
7.30
7.22
7.22
7.21
7.20
7.20
7.19
7.19
7.18 CDCl3
7.13
7.12
7.11
7.11
7.10
7.09
6.84
6.82
6.81
6.55
6.54
6.53
6.52
1.53 H2O
-0.00
NH
N
7a
1H NMR
CDCl3
500 MHz
2.00
4.10
2.09
2.12
1.11
3.06
f1 (ppm)

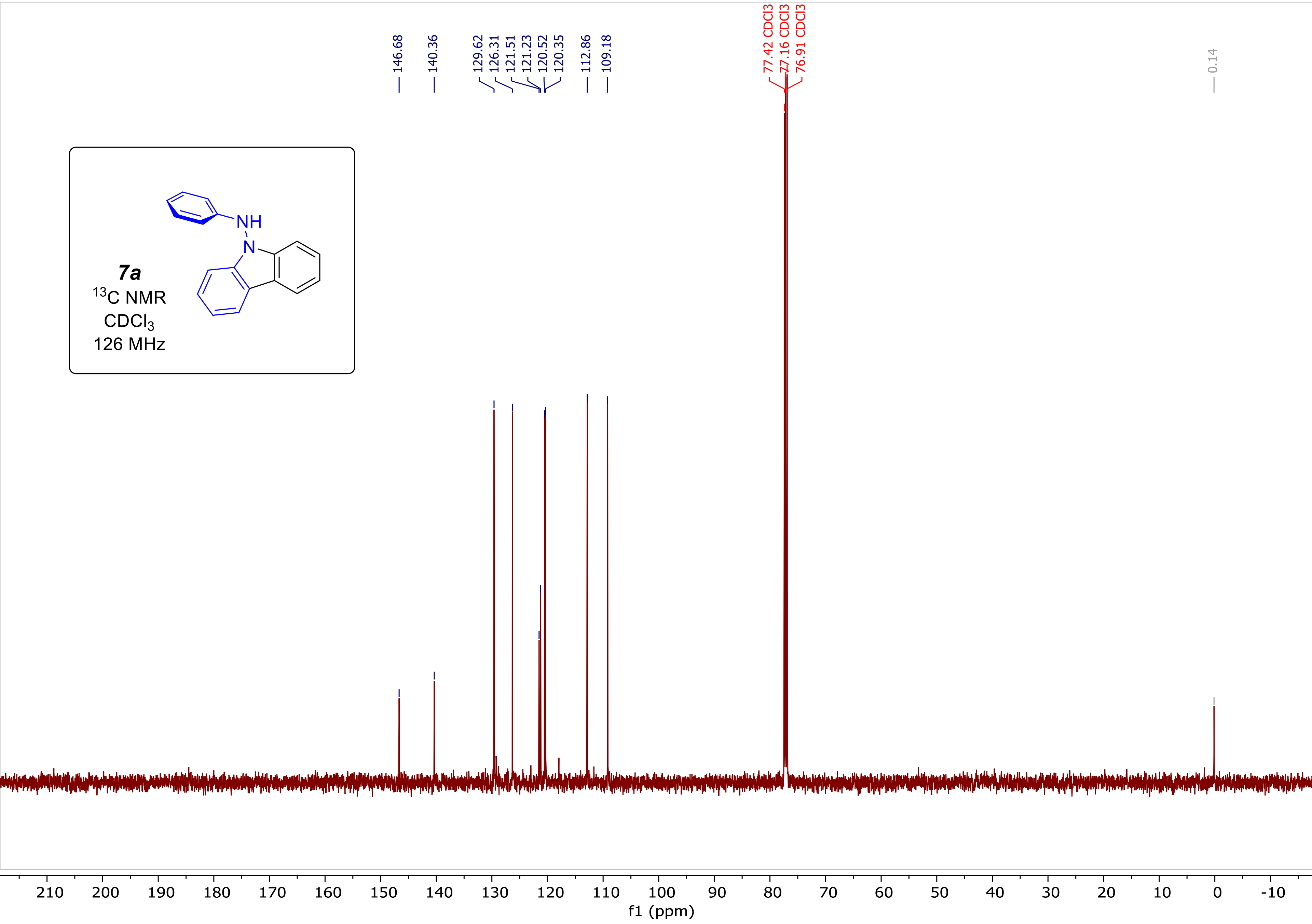

146.68
140.36
129.62
126.31
121.51
121.23
120.52
120.35
112.86
109.18
77.42 CDCl3
77.16 CDCl3
76.91 CDCl3
0.14
NH
N
7a
^{13}C NMR
$CDCl_3$
126 MHz
210
200
190
180
170
160
150
140
130
120
110
100
90
80
70
60
50
40
30
20
10
0
-10
f1 (ppm)

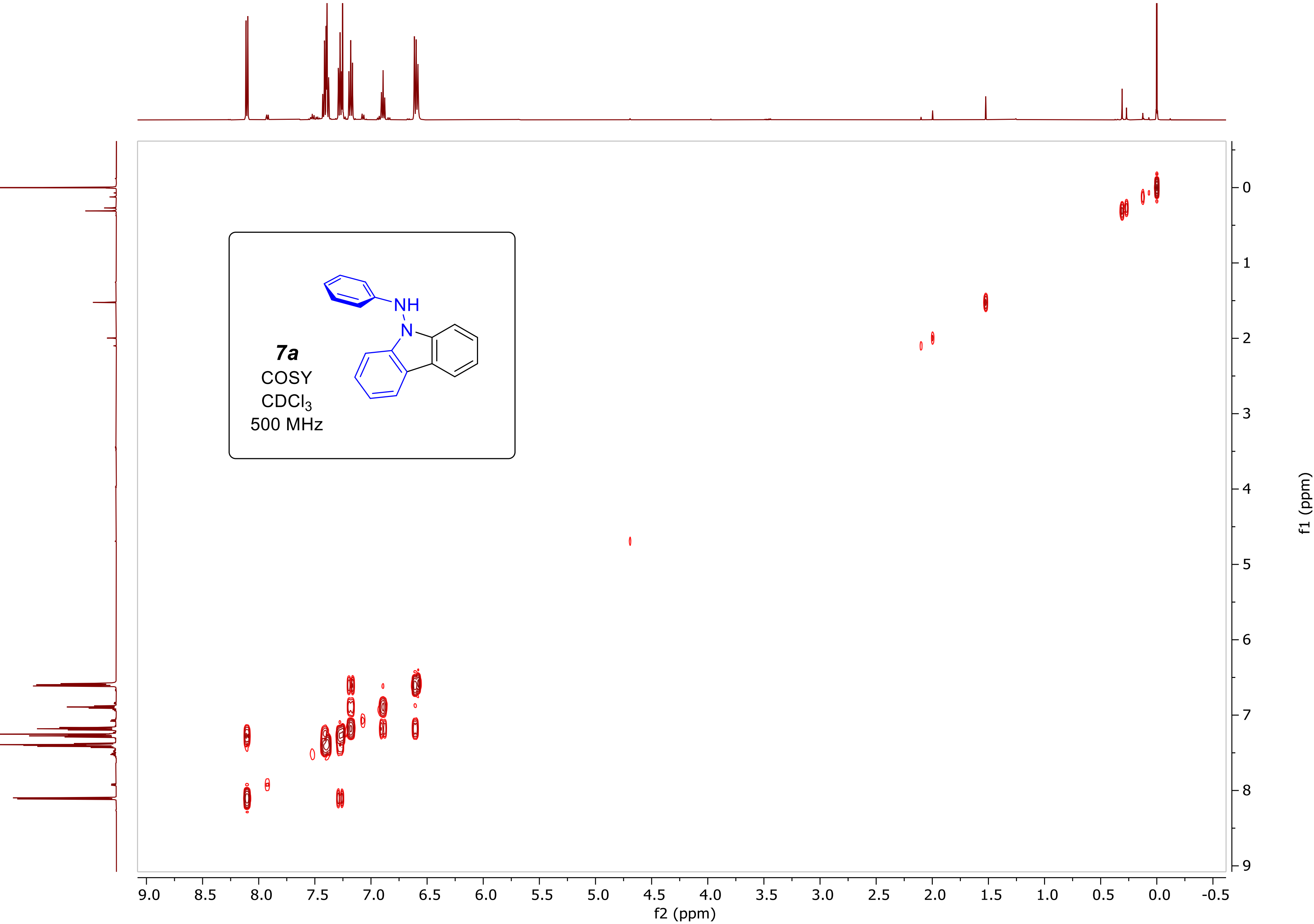
7a
COSY
$CDCl_3$
500 MHz
NH
N
f2 (ppm)
f1 (ppm)
9.0 8.5 8.0 7.5 7.0 6.5 6.0 5.5 5.0 4.5 4.0 3.5 3.0 2.5 2.0 1.5 1.0 0.5 0.0 -0.5
0 1 2 3 4 5 6 7 8 9

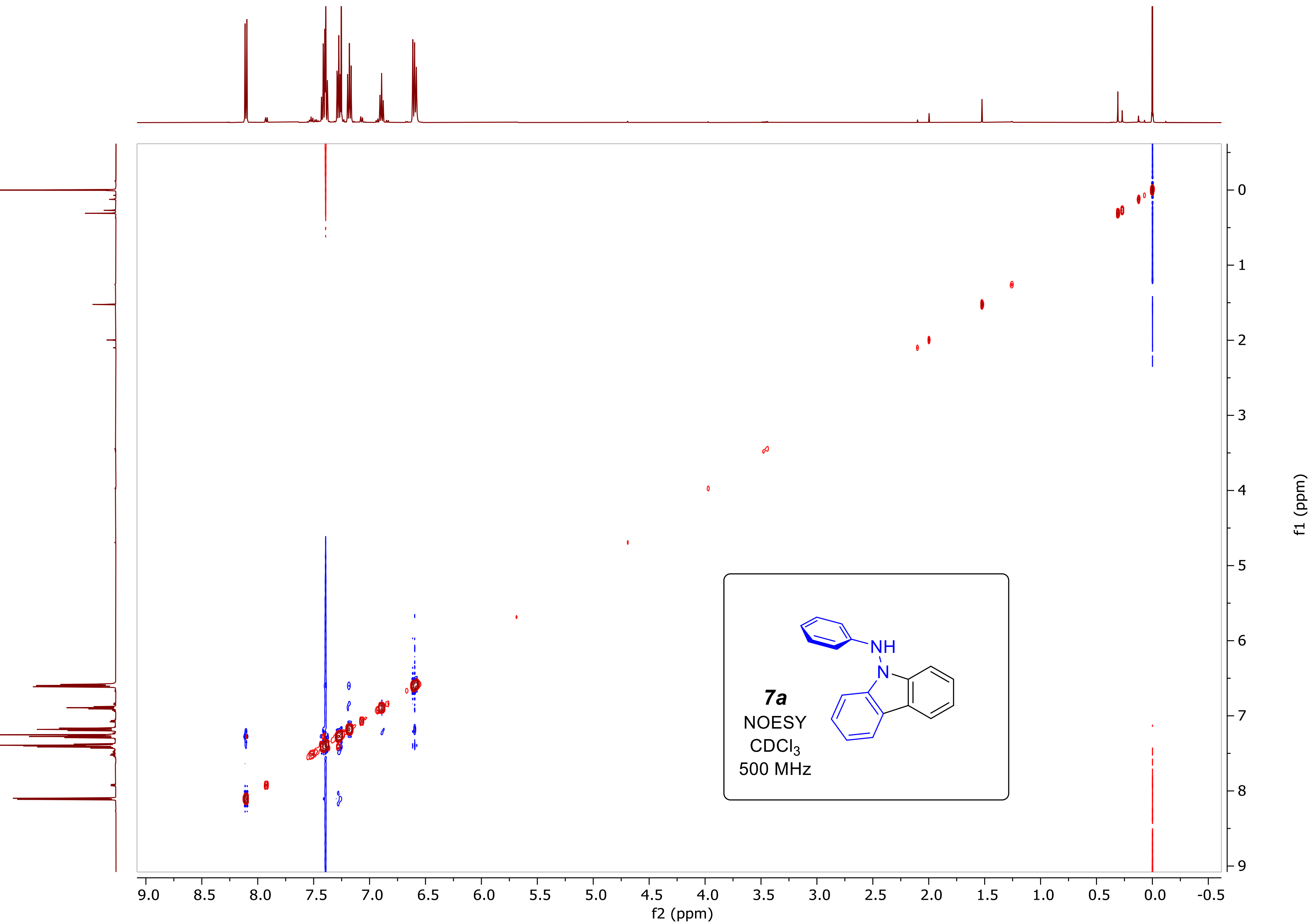
7a
NOESY
$CDCl_3$
500 MHz
NH
N
f2 (ppm)
f1 (ppm)

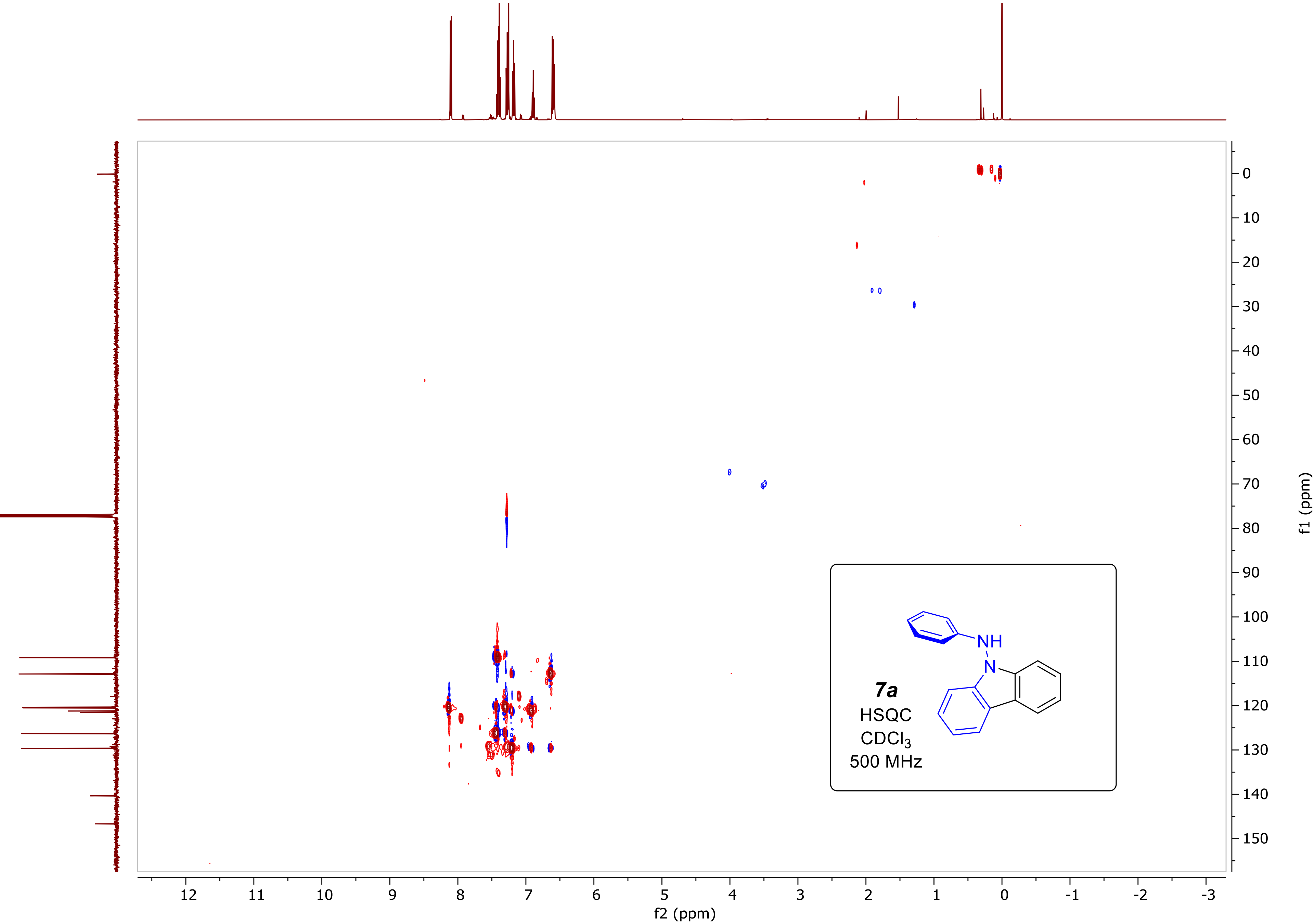
0
10
20
30
40
50
60
70
80
90
100
110
120
130
140
150
f1 (ppm)
12
11
10
9
8
7
6
5
4
3
2
1
0
-1
-2
-3
f2 (ppm)
NH
N
7a
HSQC
CDCl3
500 MHz

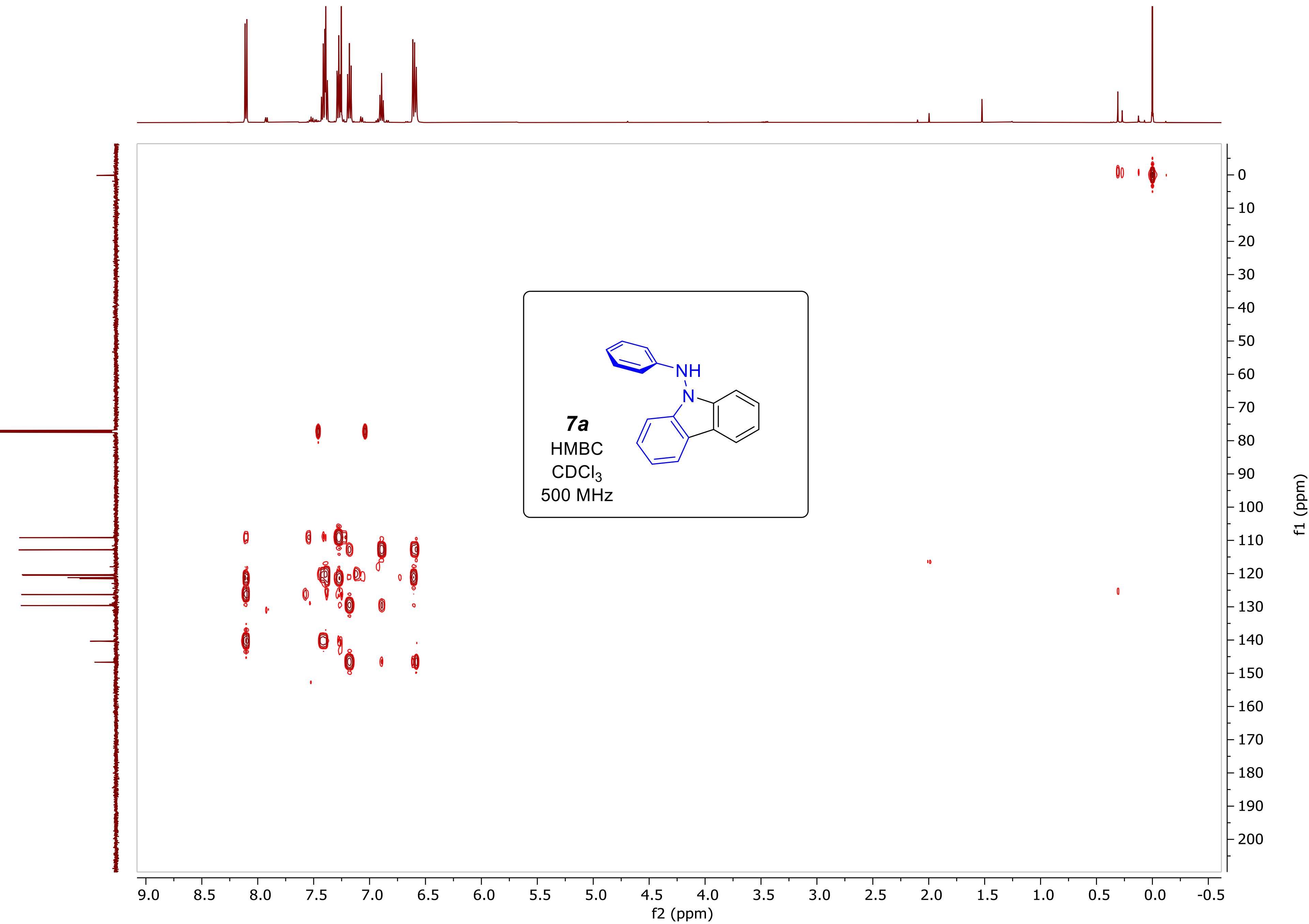
NH
N
7a
HMBC
CDCl3
500 MHz
f2 (ppm)
f1 (ppm)

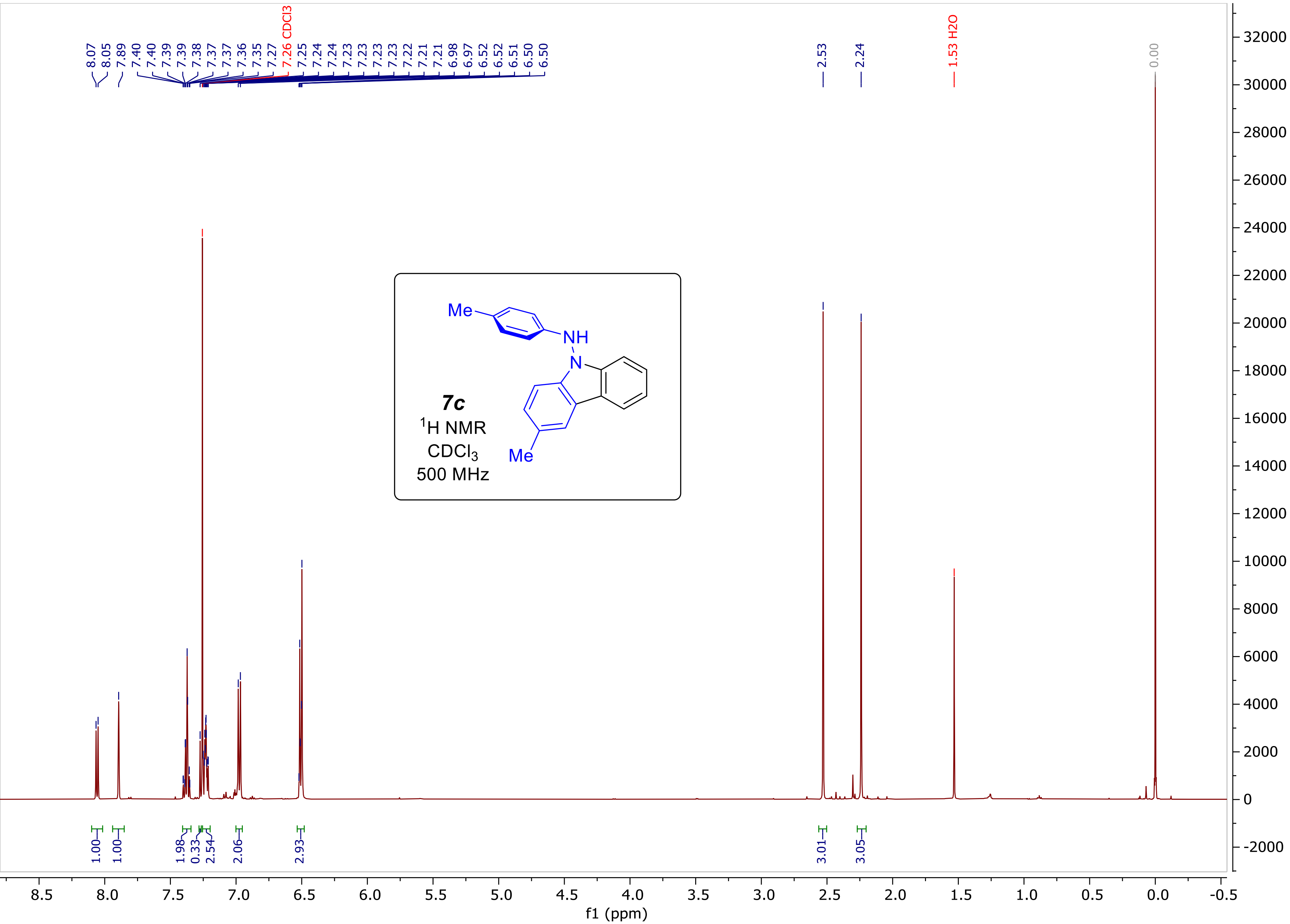
7c
¹H NMR
CDCl₃
500 MHz
Me
NH
N
Me
8.07 8.05 7.89 7.40 7.40 7.39 7.39 7.38 7.37 7.37 7.36 7.35 7.27 7.26 CDCl3 7.25 7.24 7.24 7.23 7.23 7.23 7.23 7.22 7.21 7.21 6.98 6.97 6.52 6.52 6.51 6.50 6.50
2.53 2.24 1.53 H2O 0.00
1.00 1.00 1.98 0.33 2.54 2.06 2.93 3.01 3.05
f1 (ppm)

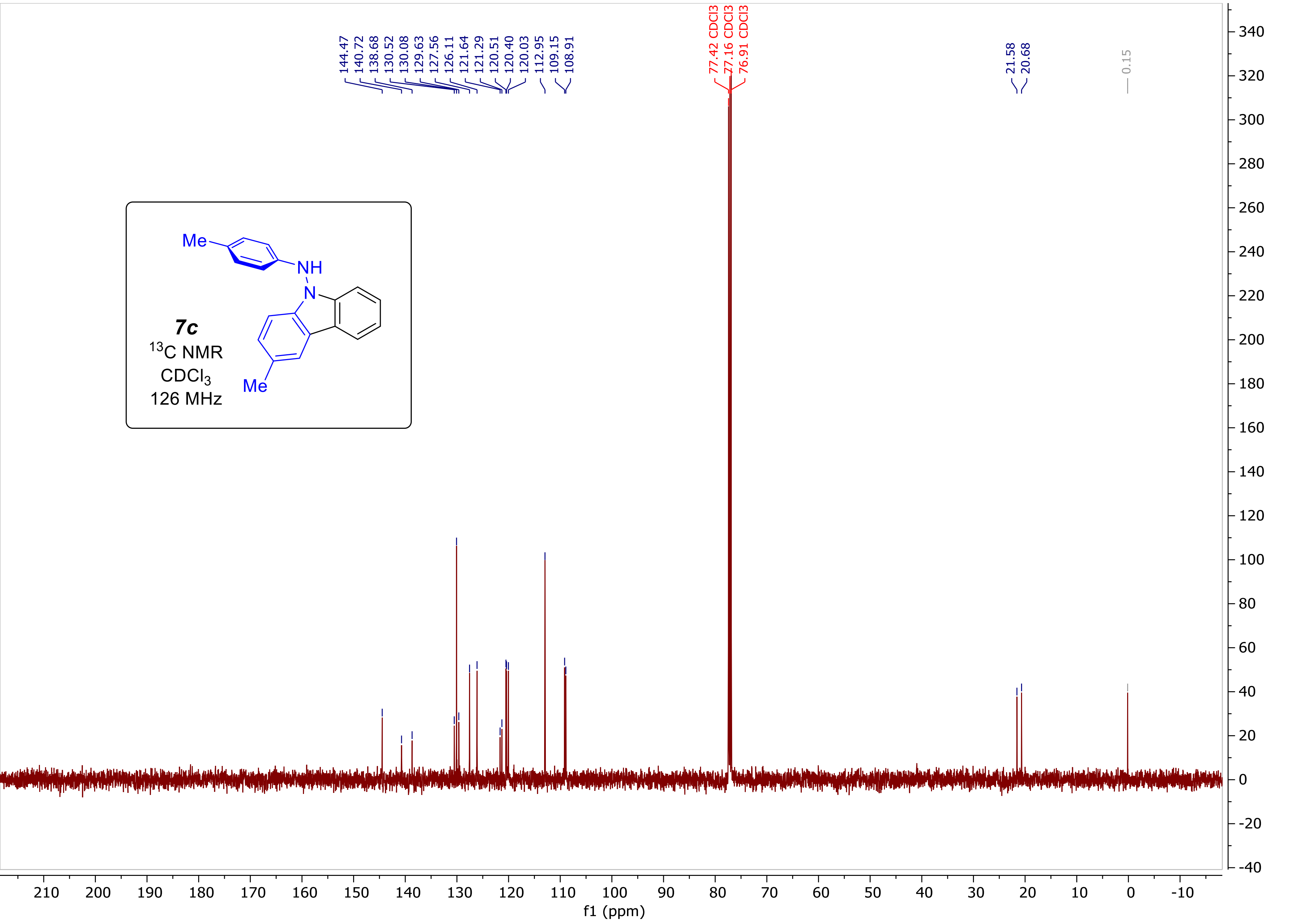
144.47
140.72
138.68
130.52
130.08
129.63
127.56
126.11
121.64
121.29
120.51
120.40
120.03
112.95
109.15
108.91
77.42 CDCl3
77.16 CDCl3
76.91 CDCl3
21.58
20.68
0.15
Me
NH
N
Me
7c
13C NMR
CDCl3
126 MHz
f1 (ppm)

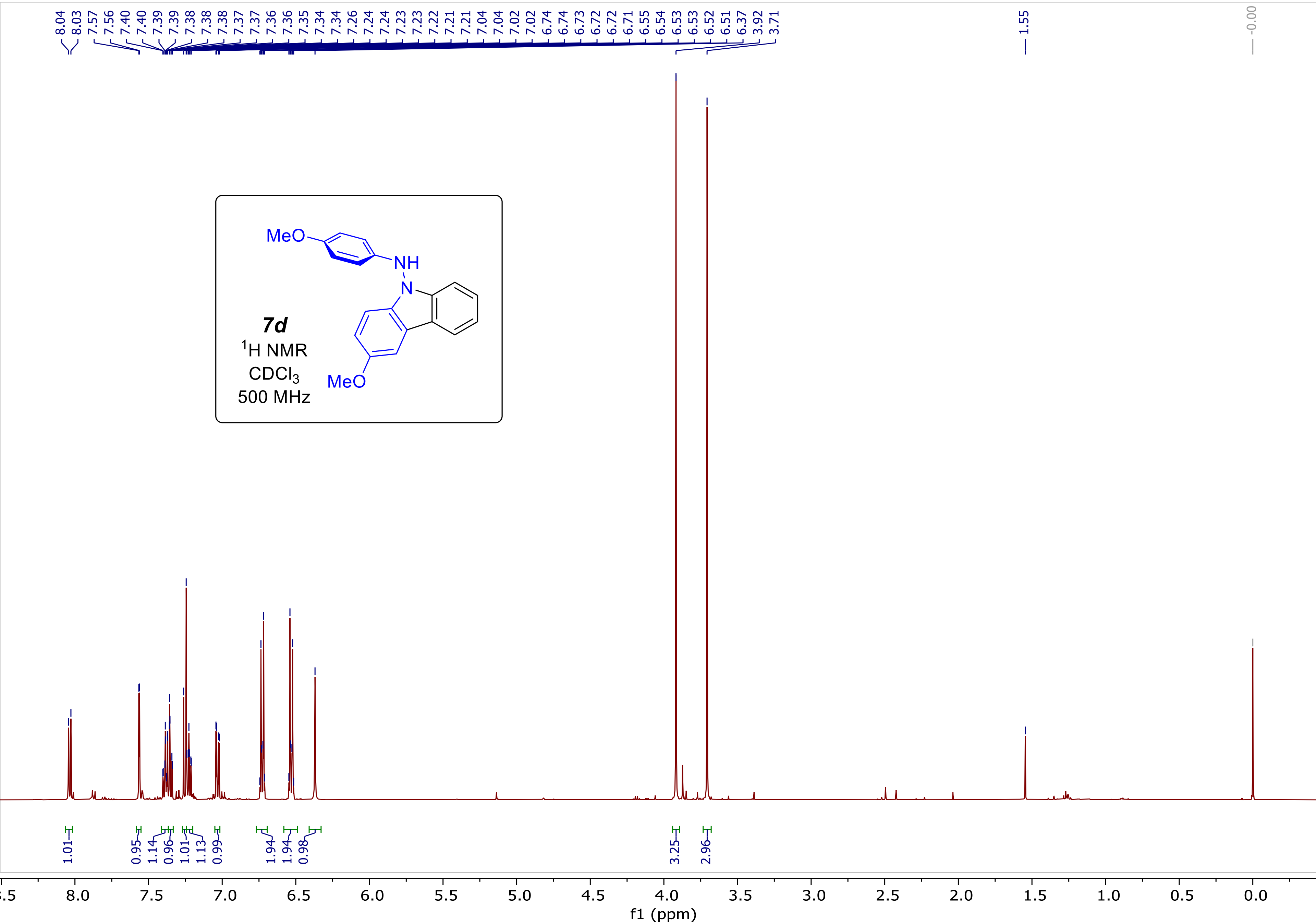
7d
^{1}H NMR
$CDCl_3$
500 MHz
MeO
NH
N
MeO
f1 (ppm)

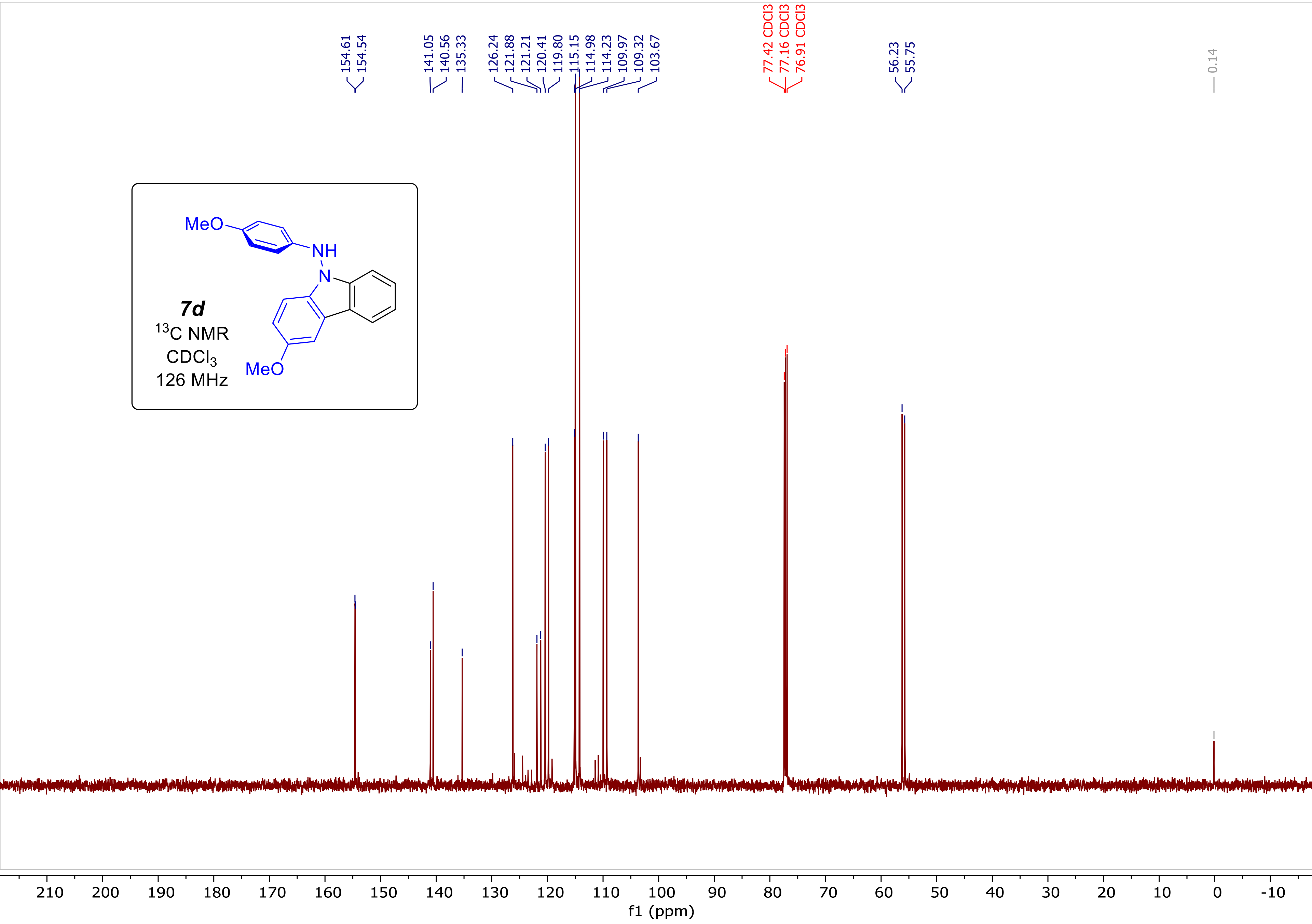
154.61
154.54
141.05
140.56
135.33
126.24
121.88
121.21
120.41
119.80
115.15
114.98
114.23
109.97
109.32
103.67
77.42 CDCl3
77.16 CDCl3
76.91 CDCl3
56.23
55.75
0.14
MeO
NH
N
MeO
7d
^{13}C NMR
$CDCl_3$
126 MHz
210 200 190 180 170 160 150 140 130 120 110 100 90 80 70 60 50 40 30 20 10 0 -10
f1 (ppm)

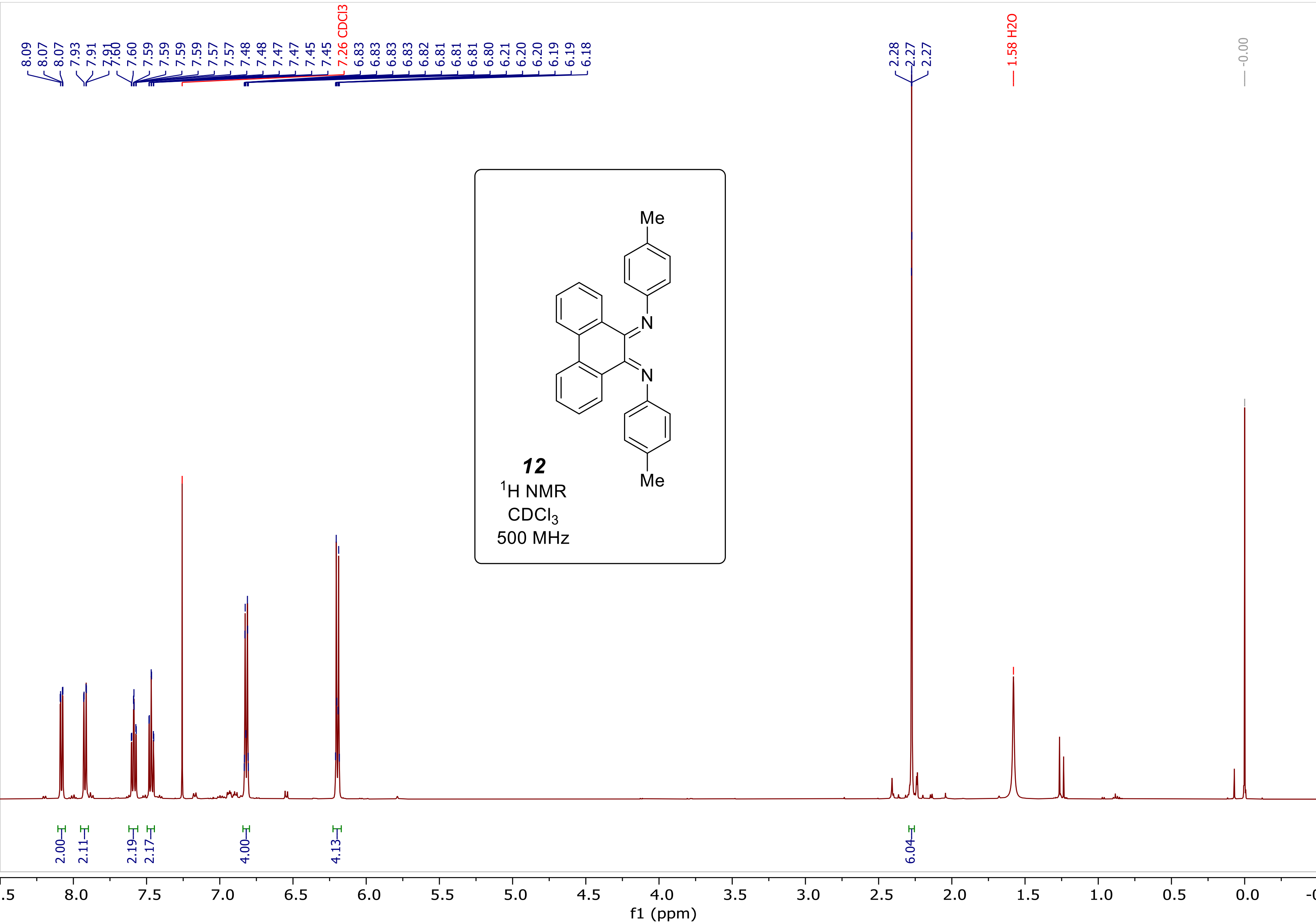
8.09
8.07
8.07
7.93
7.91
7.91
7.60
7.60
7.59
7.59
7.59
7.59
7.57
7.57
7.48
7.48
7.47
7.47
7.45
7.45
7.26 CDCl3
6.83
6.83
6.83
6.83
6.82
6.81
6.81
6.81
6.80
6.21
6.20
6.20
6.19
6.19
6.18
2.28
2.27
2.27
1.58 H2O
-0.00
Me
N
N
Me
12
1H NMR
CDCl3
500 MHz
2.00
2.11
2.19
2.17
4.00
4.13
6.04
8.5
8.0
7.5
7.0
6.5
6.0
5.5
5.0
4.5
4.0
3.5
3.0
2.5
2.0
1.5
1.0
0.5
0.0
f1 (ppm)

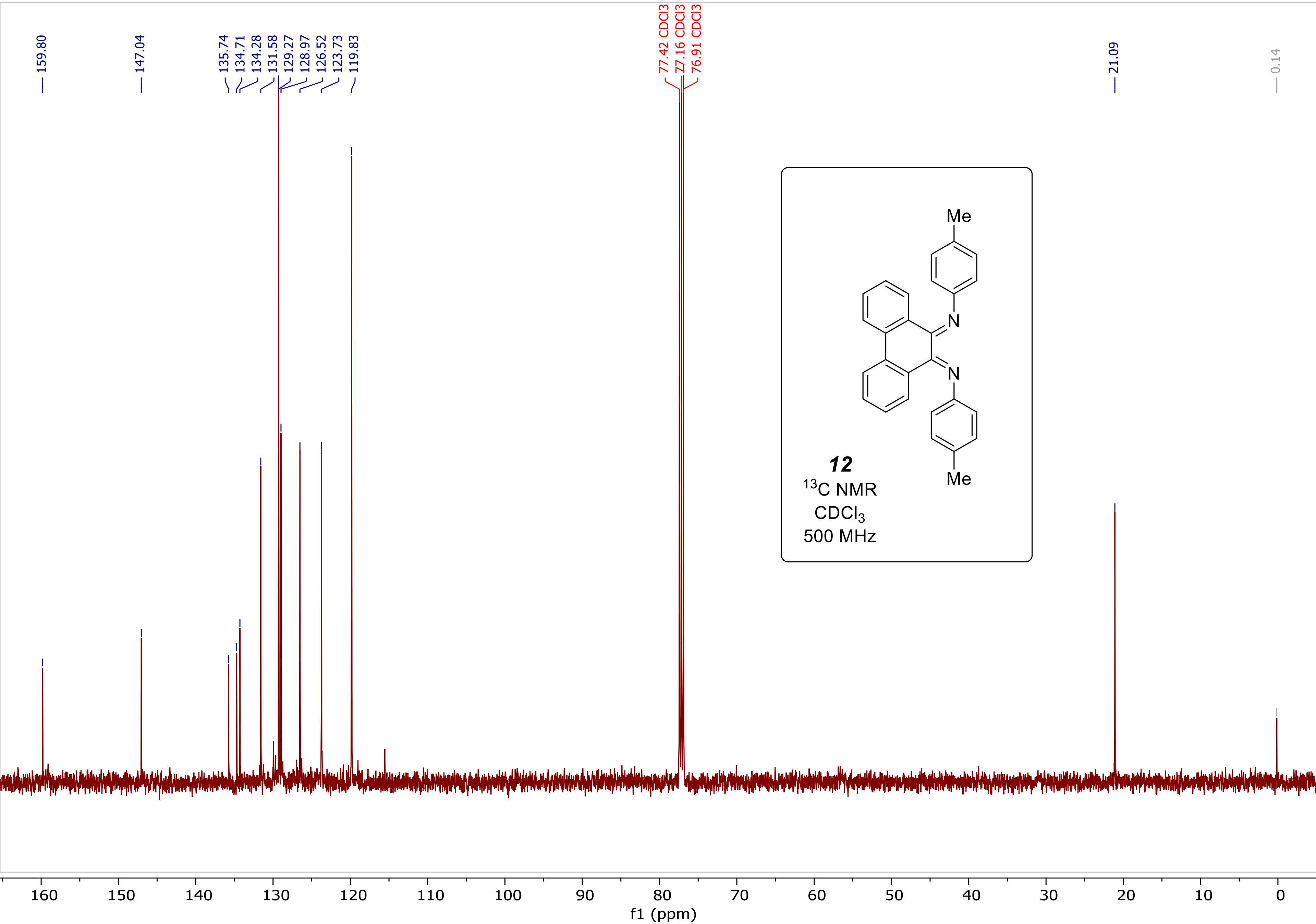
159.80
147.04
135.74
134.71
134.28
131.58
129.27
128.97
126.52
123.73
119.83
77.42 CDCl3
77.16 CDCl3
76.91 CDCl3
21.09
0.14
Me
N
N
Me
12
13C NMR
CDCl3
500 MHz
160
150
140
130
120
110
100
90
80
70
60
50
40
30
20
10
0
f1 (ppm)

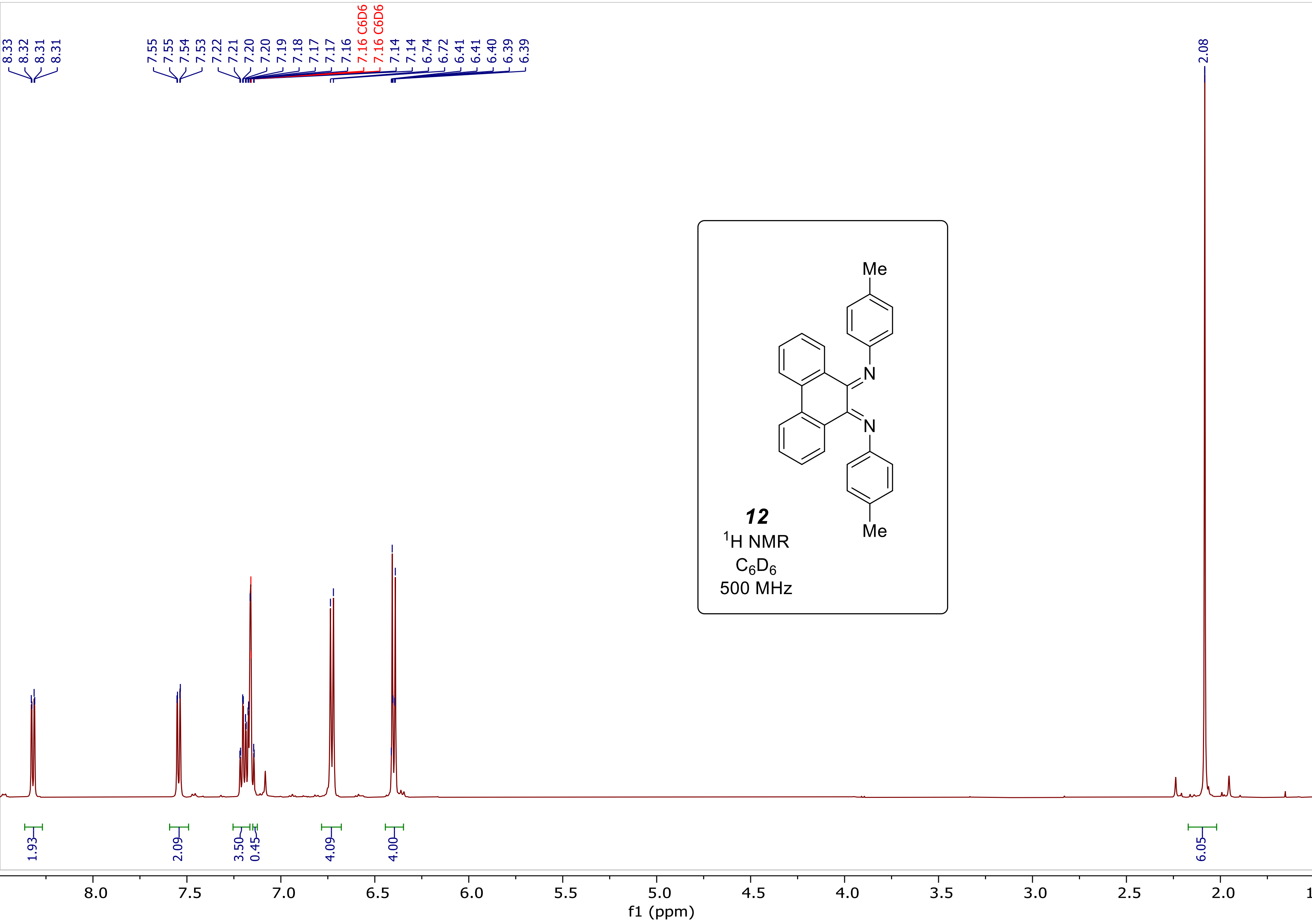
8.33
8.32
8.31
8.31
7.55
7.55
7.54
7.53
7.22
7.21
7.20
7.20
7.19
7.18
7.17
7.17
7.16
7.16 C6D6
7.16 C6D6
7.14
7.14
6.74
6.72
6.41
6.41
6.40
6.39
6.39
2.08
Me
N
N
Me
12
1H NMR
C6D6
500 MHz
1.93
2.09
3.50
0.45
4.09
4.00
6.05
8.0
7.5
7.0
6.5
6.0
5.5
5.0
4.5
4.0
3.5
3.0
2.5
2.0
f1 (ppm)

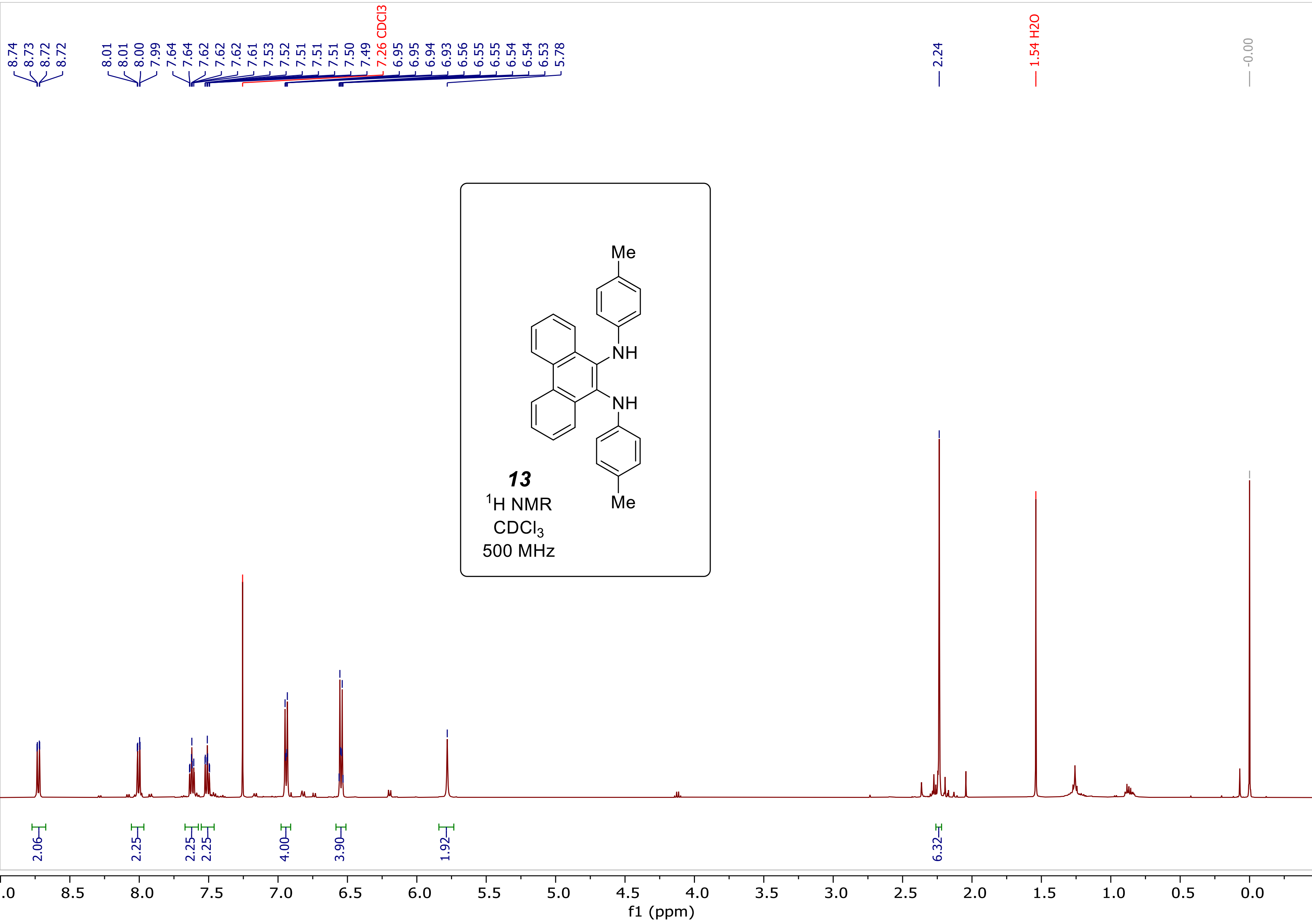
8.74
8.73
8.72
8.72
8.01
8.01
8.00
7.99
7.64
7.64
7.62
7.62
7.62
7.61
7.53
7.52
7.51
7.51
7.51
7.50
7.49
7.26 CDCl3
6.95
6.95
6.94
6.93
6.56
6.55
6.55
6.54
6.54
6.53
5.78
2.24
1.54 H2O
-0.00
Me
NH
NH
Me
13
1H NMR
CDCl3
500 MHz
2.06
2.25
2.25
2.25
4.00
3.90
1.92
6.32
f1 (ppm)

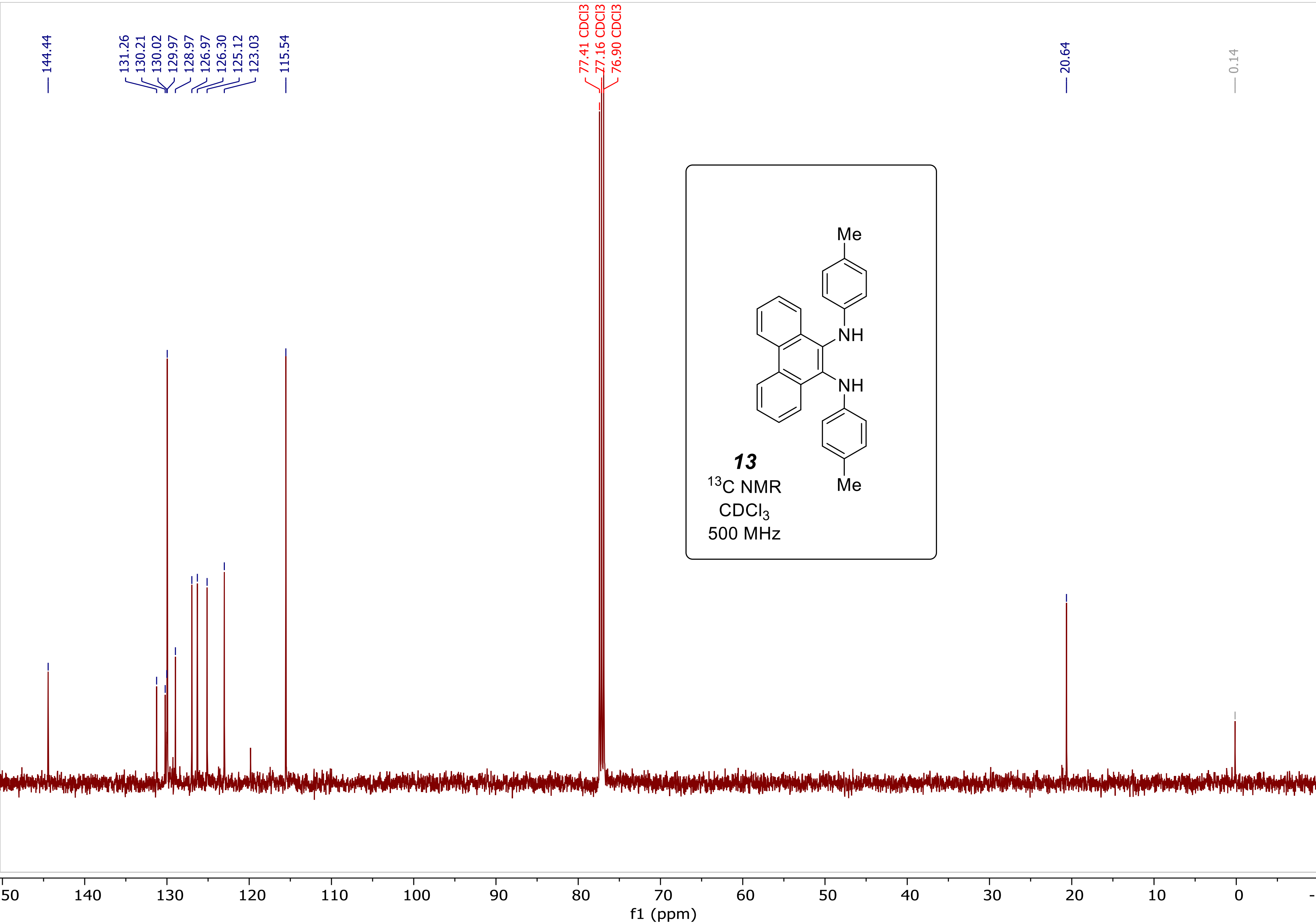
144.44
131.26
130.21
130.02
129.97
128.97
126.97
126.30
125.12
123.03
115.54
77.41 CDCl3
77.16 CDCl3
76.90 CDCl3
20.64
0.14
Me
NH
NH
Me
13
13C NMR
CDCl3
500 MHz
150
140
130
120
110
100
90
80
70
60
50
40
30
20
10
0
f1 (ppm)

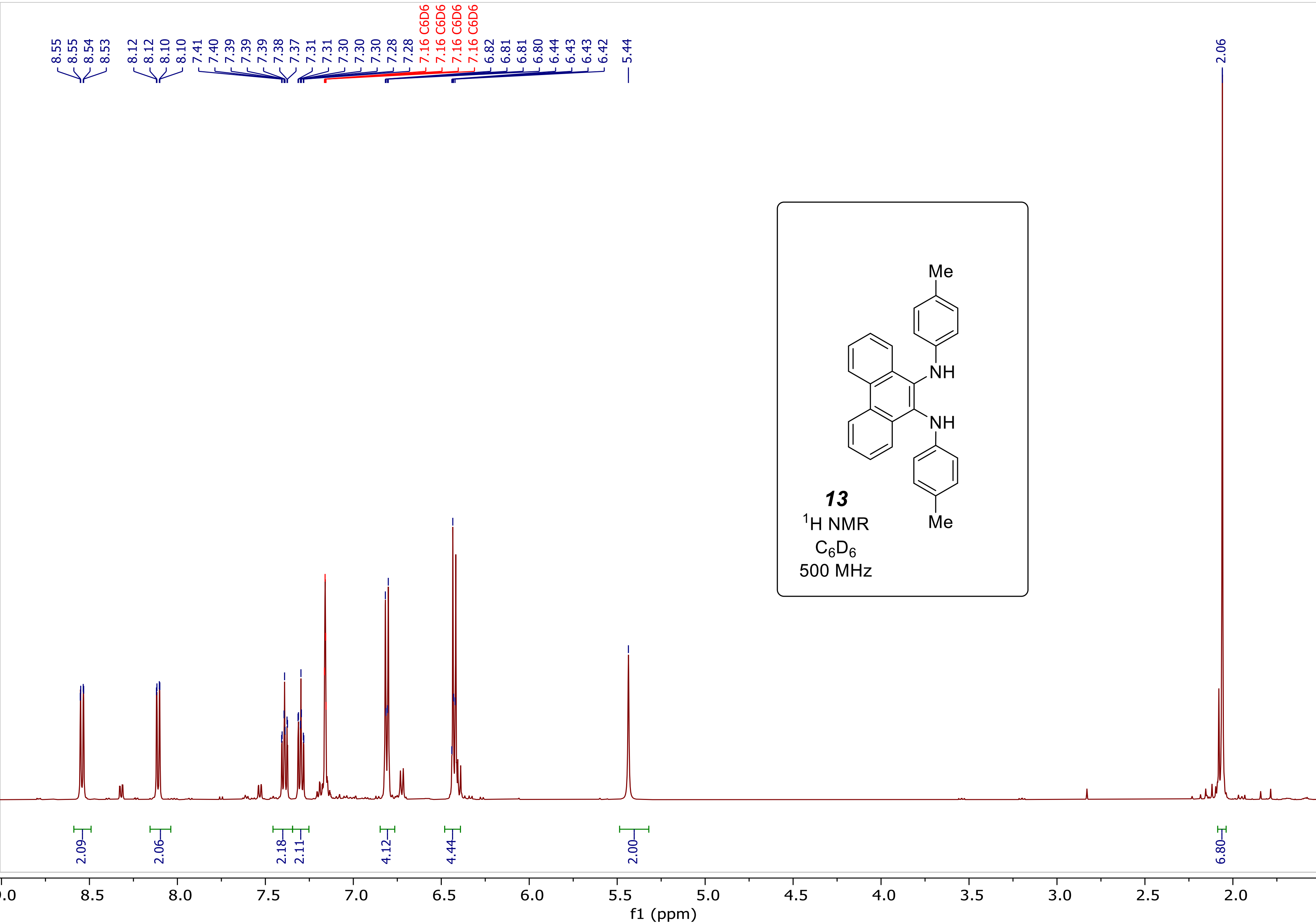
8.55 8.55 8.54 8.53 8.12 8.12 8.10 8.10 7.41 7.40 7.39 7.39 7.39 7.38 7.37 7.31 7.31 7.30 7.30 7.30 7.28 7.28 7.16 C6D6 7.16 C6D6 7.16 C6D6 7.16 C6D6 6.82 6.81 6.81 6.80 6.44 6.43 6.43 6.42 5.44 2.06
Me
NH
NH
Me
13
1H NMR
C6D6
500 MHz
2.09 2.06 2.18 2.11 4.12 4.44 2.00 6.80
9.0 8.5 8.0 7.5 7.0 6.5 6.0 5.5 5.0 4.5 4.0 3.5 3.0 2.5 2.0
f1 (ppm)

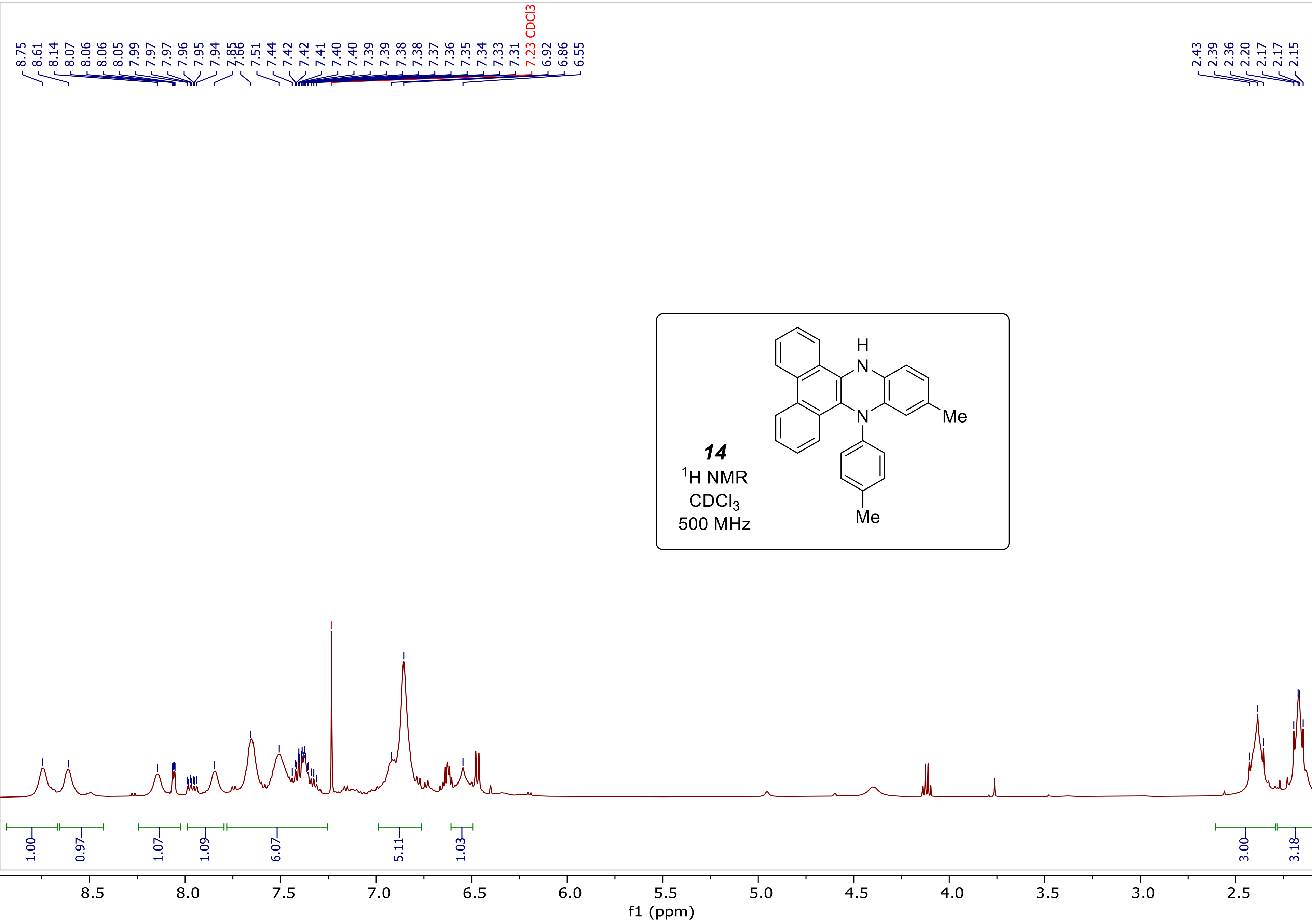
8.75
8.61
8.14
8.07
8.06
8.06
8.05
7.99
7.97
7.97
7.96
7.95
7.94
7.85
7.66
7.51
7.44
7.42
7.42
7.41
7.40
7.40
7.39
7.39
7.38
7.38
7.37
7.36
7.35
7.34
7.33
7.31
7.23 CDCl3
6.92
6.86
6.55
2.43
2.39
2.36
2.20
2.17
2.17
2.15
H
N
N
Me
Me
14
1H NMR
CDCl3
500 MHz
1.00
0.97
1.07
1.09
6.07
5.11
1.03
3.00
3.18
8.5
8.0
7.5
7.0
6.5
6.0
5.5
5.0
4.5
4.0
3.5
3.0
2.5
f1 (ppm)

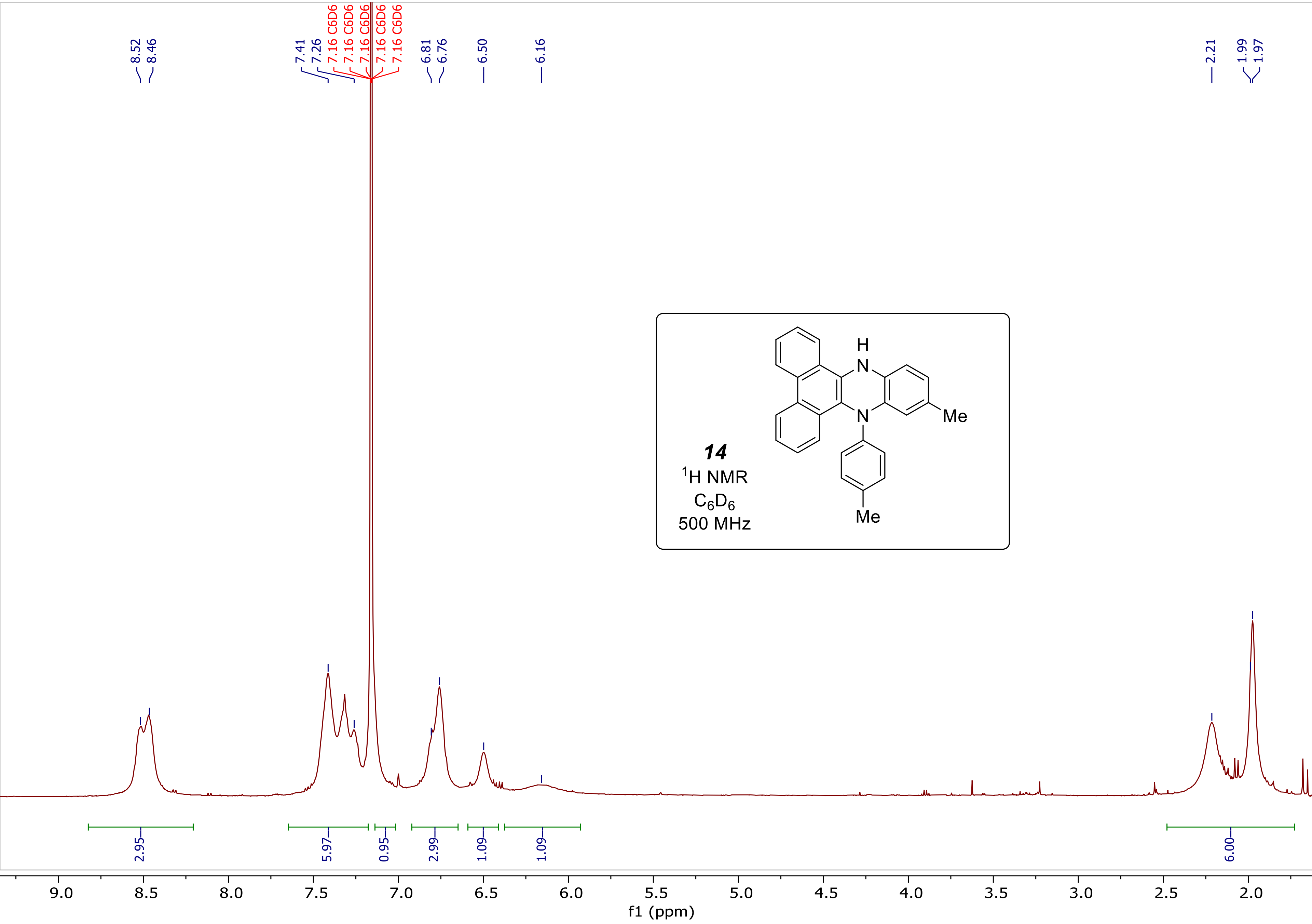
8.52
8.46
7.41
7.26
7.16 C6D6
7.16 C6D6
7.16 C6D6
7.16 C6D6
7.16 C6D6
6.81
6.76
6.50
6.16
2.21
1.99
1.97
H
N
N
Me
Me
14
1H NMR
C6D6
500 MHz
2.95
5.97
0.95
2.99
1.09
1.09
6.00
9.0
8.5
8.0
7.5
7.0
6.5
6.0
5.5
5.0
4.5
4.0
3.5
3.0
2.5
2.0
f1 (ppm)
